# Gene expression profiling in the Cynomolgus macaque *Macaca fascicularis *shows variation within the normal birth range

**DOI:** 10.1186/1471-2164-12-509

**Published:** 2011-10-16

**Authors:** Bright Starling Emerald, Keefe Chng, Shinya Masuda, Deborah M Sloboda, Mark H Vickers, Ravi Kambadur, Peter D Gluckman

**Affiliations:** 1 Growth, Development and Metabolism Programme, Singapore Institute for Clinical Sciences, Brenner Centre for Molecular Medicine, 30 Medical Drive, Singapore; 2 Liggins Institute and the National Research Centre for Growth and Development, The University of Auckland, Private Bag 92019, Auckland 1142, New Zealand; 3 Division of Molecular Genetics & Cell Biology, School of Biological Sciences, Nanyang Technological University, Singapore, Singapore; 4 Department of Anatomy, Faculty of medicine and Health Sciences, UAE University, Tawam Medical Campus, PO BOX 17666, Al ain, UAE

## Abstract

**Background:**

Although an adverse early-life environment has been linked to an increased risk of developing the metabolic syndrome, the molecular mechanisms underlying altered disease susceptibility as well as their relevance to humans are largely unknown. Importantly, emerging evidence suggests that these effects operate within the normal range of birth weights and involve mechanisms of developmental palsticity rather than pathology.

**Method:**

To explore this further, we utilised a non-human primate model *Macaca fascicularis *(Cynomolgus macaque) which shares with humans the same progressive history of the metabolic syndrome. Using microarray we compared tissues from neonates in the average birth weight (50-75^th ^centile) to those of lower birth weight (5-25^th ^centile) and studied the effect of different growth trajectories within the normal range on gene expression levels in the umbilical cord, neonatal liver and skeletal muscle.

**Results:**

We identified 1973 genes which were differentially expressed in the three tissue types between average and low birth weight animals (P < 0.05). Gene ontology analysis identified that these genes were involved in metabolic processes including cellular lipid metabolism, cellular biosynthesis, cellular macromolecule synthesis, cellular nitrogen metabolism, cellular carbohydrate metabolism, cellular catabolism, nucleotide and nucleic acid metabolism, regulation of molecular functions, biological adhesion and development.

**Conclusion:**

These differences in gene expression levels between animals in the upper and lower percentiles of the normal birth weight range may point towards early life metabolic adaptations that in later life result in differences in disease risk.

## Background

Clinical, experimental and epidemiological studies have highlighted a link between the early-life environment and the health and well-being of offspring in later life. An adverse maternal environment has been linked to an increased risk of developing metabolic and cardiovascular disorders including type 2 diabetes, obesity, hyperlipidemia, insulin resistance and hypertension [1-7]. An important feature of these studies is that these relationships exist within the normative birth range and do not depend on extremes of birth weight. This has led to the proposal that later life disease risk is the result of maladaptive consequences of plastic mechanisms which would normally be adaptive [8,9].

It is proposed that developmental plasticity determines the trajectory of development through epigenetic processes such that the fetus attempts to match its later phenotype to the environment [10]. It has been proposed that low birth weight is a marker of a poor early life nutritional environment [11] and thus a smaller fetus is more likely to develop a metabolic capacity appropriate for a low nutrient postnatal environment. But, if faced with a high nutrient environment it is more likely to become obese and insulin resistant [12]. Although, epigenetic processes have been increasingly implicated largely from rodent studies involving nutritional manipulation of the dam [13,14] the molecular mechanisms underlying altered disease susceptibility are largely unknown. There is also some evidence that these developmental trajectories, and associated long-term gene expression and epigenetic changes can be reversed by the administration of the adipokine leptin to the neonatal rat although the concentrations used were higher than physiological levels [10,12,15,16]. These data suggest that a better understanding of the molecular events associated with impaired early life development may help in designing future intervention strategies.

To identify the possible molecular pathways associated with variations in the fetal environment, we have utilised a non-human primate (NHP) model, the *Macaca fascicularis *(Cynomolgus macaque) to elucidate whether variations within the normal birth weight range are associated with differential gene expressions patterns. Cynomolgus macaques share with humans the same progressive history of the metabolic syndrome [17] which makes this model directly relevant to humans and importantly, Cynomolgus macaque is a monotocous species in which spontaneous variation in fetal growth rather than experimental manipulation can be investigated. This study therefore we have investigated the effect of spontaneous lower birth weight on gene expression in key tissues (umbilical cord, hepatic tissue and skeletal muscle) from female Cynomolgus macaque neonates.

## Methods

### Collection of Umblical cords

Sixty-five pregnant Cynomolgus macaque dams, sired naturally by one male, were monitored prior to delivery at the Vietnam Primate Breeding and Development Corporation. After birth, dams were sedated (ketamine-HCl; 7 mg/kg) to facilitate collection of the umbilical cord. The cords were collected and immediately snap-frozen in liquid nitrogen and stored at -80°C for later analyses. Neonates were weighed at birth and promptly returned to the dams. All animal procedures were approved by Nafovanny, subsidiary of the Ministry of Forestry, Vietnam, and performed in accordance with the guidelines set by the national advisory committee for laboratory animal research (NACLAR) of Singapore.

### Collection of hepatic and skeletal muscle samples

The normative birth range was assessed from these 65 pregnancies and 8 neonates were selected based on their birth weights to comprise 2 groups: 1) ***lower birth weight group (LBW)***; n = 4 classified as those that were within the 5^th ^to 25^th ^birth weight percentile, birth weight range 299-317 g and 2) ***average birth weight group (ABW); ***n = 4 classified as those that were within the 50^th ^to 75^th ^birth weight percentile, birth weight range 358-398 g. The normal gestation of Cynomolgus macaque is approximately 155-170 days [18]. We have estimated the gestational age based on early ultrasound measurements (greatest length of the embryo at the time of pregnancy detection) and used those pregnancies where fetuses were within normal distribution for full term Cynomolgus macaques [18]. On postnatal day 5, neonates were sedated with an intramuscular injection of ketamine-HCl (15 mg/kg), and exsanguinated under anesthesia. Liver and skeletal muscle (biceps femoris) were collected and immediately snap frozen in liquid nitrogen and stored at -80°C for later analyses

### Preparation of Total RNA

Total RNA was isolated from umbilical cords and neonatal tissues using TRIzol reagent according to the manufacturer's instructions (Invitrogen). RNA integrity was confirmed by bio analyzer 2100 (Agilent Technologies, Santa Clara, USA). An RNA Integrity Number (RIN) value of 7.5 above was considered acceptable and used in further experiments.

### Microarray analysis

For microarray analysis, RNA from 6 groups of samples (Cord: ABW and LBW; Liver: ABW and LBW; skeletal muscle: ABW and LBW) were labeled using QuickAmp 1-color labeling kit (Agilent Technologies) according to manufacturer's protocol. The Cy3 labeled cRNA were subsequently hybridized to Agilent Rhesus Macaque (G2519F-015421) Gene Expression microarray. The Rhesus macaque gene expression microarray used in this study represented 43,803 Rhesus monkey probes synthesized as 60-mers spotted using the Agilent SurePrint technology (Agilent Technologies). The microarrays were scanned with Agilent High resolution Scanner and the images were feature extracted using FE software version 10.5 (Agilent Technologies).

Data analysis was performed using Genespring GX ver10 (Agilent Technologies). The raw signal intensity from each samples is global normalized to 75th percentile and base-line transformed. Probes flagged with present call in at least 75% of the samples in any of the 6 groups were used for subsequent data analysis.

Two-way ANOVA was performed with p value cut-off at 0.05 to identify genes that are differentially expressed in the tissue type and birth weight. Due to limited annotation of Rhesus Monkey genome, the microarray probes are annotated against human genes and ontology. For mapping against the human genome, the probes from the monkey microarray were re-annotated using Agilent eARRAY. The probe sequences were aligned to human genome (hg18) using BLAST based algorithm and the associated human annotation was extracted from the database.

To study the gene expression profile of the birth weight in each of the tissue type, Welch T-Test with p-value cut off of 0.05 and fold change of 1.5X was performed between the ABW and LBW samples in each of the tissue groups.

Complete microarray data is available at the Gene Expression Omnibus (GEO) database under the accession number GSE32069.

### Network Analysis

The microarray data was imported into Pathway Studio version 7 (Ariadne Genomics, Rockville, USA) for network analysis. Gene Set Enrichment Analysis (GSEA) was performed on ABW *vs *LBW in the respective tissue using Kolmogorov-Smirnov algorithm with p-value cut-off at 0.05. In addition, Network Enrichment Analysis (NEA) was performed to identify expression hub of the treatment. Sub network was generated by connecting entities to their expression target network using the Resnet 7 Mammalian database.

### Quantitative RT-PCR

Five up-regulated and two down regulated genes were selected for verification using qRT-PCR. The primers were designed using the Cynomolgus if available or Rhesus macaque sequences using the primer 3 software if not available [19]. The gene symbols and the primers are listed in Table 1.

**Table 1:** Sequences of primers used for qRT-PCR:

PASK F 5' CTACTCCGGGAGCTGCTATC 3'
PASK R5' AGCAGCAGAACAGAGGTGTG 3'



116936 F5' GCACATCTGCCTGAAGTGAA 3'

116936 R5' GAGCAGCTTGTCCAGGAAGT 3'



ADK F5' TGGTGGCTCTACCCAGAACT 3'

ADK R5' CATCTACATGGGCTTCAGCA 3'



ELMO F5' AGCTCTGTGTGGCTTGGTTT 3'

ELMO R5' CGGTGTGAATAACGGAGTCCT 3'



SIX1 F5' GTTTAAGAACCGGAGGCAAA 3'

SIX1 R5' GGAGAGAGTTGGTTCTGCTTG 3'



SLC12A9 F5' GGCTTCAACAGCAGTACCCT 3'

SLC12A9 R5' AAGAGGACAGCAAAGACGCT 3'



RBL1 F5' TAGCCTGACCAACATGGAGA 3'

RBL1 R5' GTTCAAGCAATTCTGCCTCA 3'



Uni18SrRNA F5' AGTCCCTGCCCTTTGTACACA 3'

Uni18SrRNA R5' GATCCGAGGGCCTCACTAAAC 3'

We used skeletal muscle RNA to verify the array. Briefly, total RNA was extracted as mentioned above from four ABW and four LBW neonates and were reverse transcribed using Applied Biosystem's high-capacity cDNA reverse transcription kit using 1 μg of total RNA in a reaction volume of 20 μl. The PCR reactions were carried out in 25 μl of SYBR Green Master Mix with 200 ng of cDNA using 7500 real time PCR system (Applied Biosystems, CA, USA). The comparative Ct method was used to calculate the relative gene expression [20]. 18S RNA was used as the internal control which was validated using the method described in Schmittgen and Livak [20] and found to be stable and consistent.

## Results

### Microarray analysis

**Figure 1:**
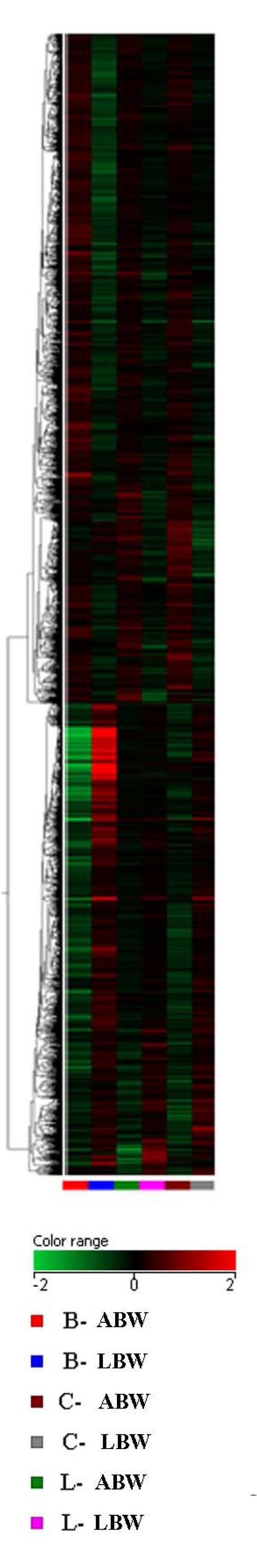
**Hierarchical clustergram of 1973 genes (1141 up regulated in LBW and 832 down regulated in LBW) identified by ANOVA (P < 0.05) in all the three tissues (umbilical cord (C), liver (L) and skeletal muscle (B) analyzed**. The relative expression is reflected by the intensity of the color (Green = down regulated, red = up regulated)

Two-way factorial ANOVA identified 1973 genes which were differentially expressed in the three tissue types between ABW and LBW neonates (P < 0.05). Of these, 1141 genes were up regulated in the LBW samples while 832 genes were down-regulated in the LBW samples compared to ABW (Figure 1).

**Figure 2:**
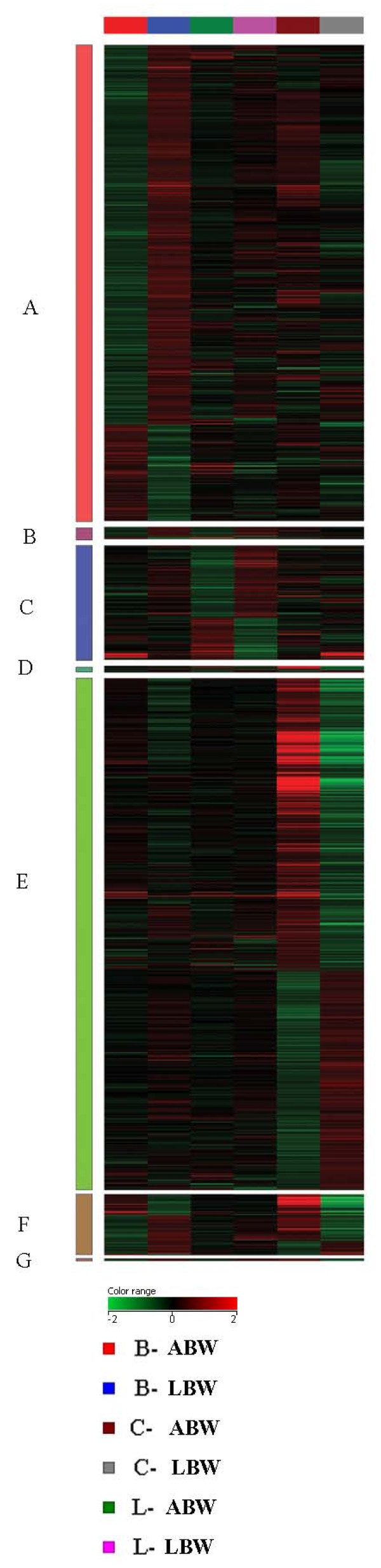
**Heat map depicting the differentially expressed genes in skeletal muscle (850 genes), liver (210 genes) and cord (891 genes) (P < 0.05, >= 1.5 fold difference)**. **A**. 733 genes (584 genes up regulated in LBW skeletal muscle, 149 genes down regulated in LBW skeletal muscle), **B**. 19 genes (16 genes up regulated in LBW skeletal muscle and 15 genes up regulated in LBW liver, 3 genes down regulated in LBW skeletal muscle and 4 genes down regulated in liver). **C **182 genes (115 genes up regulated in LBW liver and 67 genes down regulated in LBW liver). **D **5 genes (2 genes up regulated in LBW cords and 3 genes up regulated LBW liver, 3 genes down regulated in LBW cords and 2 genes down regulated in LBW liver). **E**. 788 genes (338 genes up regulated in LBW cord and 450 genes down regulated LBW cord). **F**. 94 genes (60 genes up regulated in LBW skeletal muscle and 22 up regulated in LBW cord, 34 genes down regulated in LBW skeletal muscle and 72 genes down regulated in LBW cord. **G**. 4 genes (4 genes up regulated in LBW skeletal muscle and liver, 4 genes down regulated in LBW cord).

**Figure 3:**
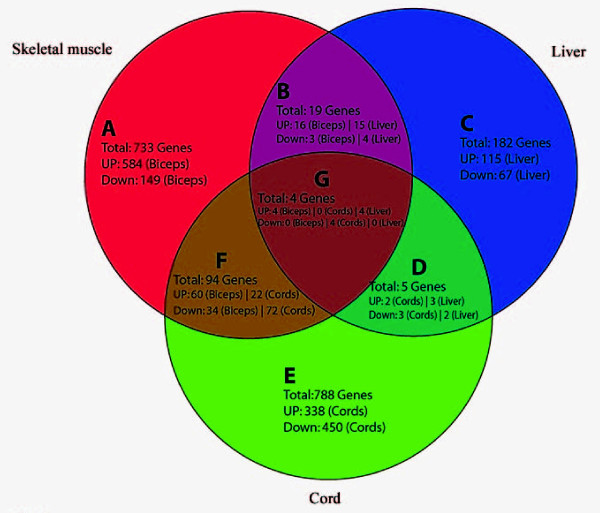
**Venn diagram depicting the differentially expressed genes in skeletal muscle (850 genes), liver (210 genes) and cord (891 genes) t-test unpaired unequal variance, LBW *vs *ABW, p < 0.05, >= 1.5 fold difference**.

Gene expression profiles of umbilical cord, liver and skeletal muscle were also compared using Welch-t-test. There were 250 genes significantly and differently expressed in the liver, 850 genes significantly and differently expressed in the skeletal muscle and 891 genes significantly and differently expressed in cord samples (P < 0.05, >1.5 fold difference) (Figure 2, 3). The top 50 genes whose gene expression levels changed in each tissue based on their fold difference based on Welch T-test are given in Tables 2, 3, 4.

**Table 2:** List of top 50 genes significantly differentially regulated in the skeletal muscle based on fold difference (t-test).

Gene Symbol	2Way ANOVA P value	Fold Change in Skeletal muscle, Welch t-test	Regulation in LBW	Fold change Liver, Welch t-test	Regulation in LBW	Fold change in Cords, Welch t-test	Regulation in LBW	Gene name
XM_116936	0.017913306	10.054285	up	1.080386	up	2.185324	up	PREDICTED: Homo sapiens similar to RIKEN cDNA 4832428D23 gene (LOC196541) mRNA [XM_116936]

XM_056254	0.021895792	9.569201	up	2.09208	down	1.391897	up	PREDICTED: Homo sapiens heparan sulfate (glucosamine) 3-O-sulfotransferase 4 (HS3ST4) mRNA [XM_056254]
C5orf23	0.01475329	6.0808635	up	1.072585	up	1.301361	up	Homo sapiens chromosome 5 open reading frame 23 (C5orf23), mRNA [NM_024563]

PASK	0.03426426	5.977056	up	2.995584	up	19.17474	up	Homo sapiens PAS domain containing serine/threonine kinase (PASK), mRNA [NM_015148]
WDR8	0.004681234	5.6804295	down	2.978104	down	8.061375	down	Homo sapiens WD repeat domain 8 (WDR8), mRNA [NM_017818]

ADK	1.63E-04	5.639505	down	1.368648	down	9.386349	down	Homo sapiens adenosine kinase (ADK), transcript variant ADK-short, mRNA [NM_001123]
APOBEC3G	0.040698703	4.8567457	down	1.669566	down	1.105857	down	Homo sapiens apolipoprotein B mRNA editing enzyme, catalytic polypeptide-like 3G (APOBEC3G), mRNA [NM_021822]

CCL2	0.002121243	4.757898	down	1.287543	down	2.895307	down	Homo sapiens chemokine (C-C motif) ligand 2 (CCL2), mRNA [NM_002982]
A_01_P013390	0.029869065	4.6248507	down	1.099004	down	1.527467	down	

BC044226	0.04677891	4.3998837	down	1.966762	down	1.502299	down	Homo sapiens myosin binding protein H, mRNA [BC044226]
CCL11	0.008992519	4.050988	down	3.178148	down	1.002582	up	Homo sapiens chemokine (C-C motif) ligand 11 (CCL11), mRNA [NM_002986]

POU4F3	0.02459994	3.8642302	up	1.615214	up	1.991698	down	Homo sapiens POU domain, class 4, transcription factor 3 (POU4F3), mRNA [NM_002700]
XR_011794	0.010519872	3.7855186	up	3.637105	up	2.76764	up	PREDICTED: Macaca mulatta similar to poly(A)-specific ribonuclease (PARN)-like domain containing 1 (LOC707835), mRNA [XR_011794]

H1FOO	0.011109326	3.7804163	up	1.404401	up	1.449255	down	Homo sapiens H1 histone family, member O, oocyte-specific (H1FOO), mRNA [NM_153833]
C17orf75	0.001362531	3.7583363	down	3.447702	down	1.443745	down	Homo sapiens chromosome 17 open reading frame 75 (C17orf75), mRNA [NM_022344]

RNF216	0.001650785	3.7307158	down	1.504036	down	6.793567	down	Homo sapiens TRIAD3 protein (TRIAD3), transcript variant 1, mRNA [NM_207111]
GTSF1	0.017040279	3.6632807	up	4.653995	up	2.487166	up	Homo sapiens family with sequence similarity 112, member B (FAM112B), mRNA [NM_144594]

C13orf33	0.006214988	3.5870998	down	2.664488	down	1.061963	up	Homo sapiens chromosome 13 open reading frame 33 (C13orf33), mRNA [NM_032849]
CA2	0.033340864	3.5736616	down	3.483002	down	1.194329	up	Homo sapiens carbonic anhydrase II (CA2), mRNA [NM_000067]

CO645773	0.03704907	3.5384166	down	2.022003	down	1.474969	down	ILLUMIGEN_MCQ_30118 Katze_MMPB Macaca mulatta cDNA clone IBIUW:22572 5' similar to Bases 1 to 42 highly similar to human RARRES3 (Hs.17466), mRNA sequence [CO645773]
MYST3	0.013409907	3.3029778	up	2.827006	up	1.403819	up	Homo sapiens MYST histone acetyltransferase (monocytic leukemia) 3 (MYST3), mRNA [NM_006766]

NM_000977	0.008561992	3.2655115	up	2.138359	up	2.014805	up	Homo sapiens ribosomal protein L13 (RPL13), transcript variant 1, mRNA. [NM_000977]
XR_014265	0.001382546	3.2319098	down	1.270026	down	2.168579	down	PREDICTED: Macaca mulatta hypothetical protein LOC716045 (LOC716045), mRNA [XR_014265]

NM_001004685	0.043442905	3.1747854	up	1.353824	up	1.407273	down	Homo sapiens olfactory receptor, family 2, subfamily F, member 2 (OR2F2), mRNA. [NM_001004685]
XR_014204	0.029225934	3.167453	down	3.849135	down	1.084816	up	PREDICTED: Macaca mulatta hypothetical protein LOC719546 (LOC719546), mRNA [XR_014204]

DARS	3.99E-05	3.1527941	down	1.10567	down	12.63266	down	Homo sapiens aspartyl-tRNA synthetase (DARS), mRNA [NM_001349]
GYLTL1B	9.45E-04	3.143812	up	1.169517	up	3.19979	up	Homo sapiens glycosyltransferase-like 1B (GYLTL1B), mRNA [NM_152312]

CNNM2	1.94E-04	3.1423542	up	1.00608	down	1.785853	up	Homo sapiens cyclin M2 (CNNM2), transcript variant 1, mRNA [NM_017649]
C20orf26	0.023210809	3.1168563	up	1.353857	up	1.414684	up	Homo sapiens chromosome 20 open reading frame 26 (C20orf26), mRNA [NM_015585]

SLC26A9	0.042099766	3.0884879	up	2.137558	up	1.776504	up	Homo sapiens solute carrier family 26, member 9 (SLC26A9), transcript variant 1, mRNA [NM_052934]
SEC14L3	0.025527291	3.0402198	up	1.787294	up	1.547541	down	Homo sapiens SEC14-like 3 (S. cerevisiae) (SEC14L3), mRNA [NM_174975]

TMEM20	0.001055841	2.9346027	down	1.821723	down	1.558415	down	Homo sapiens transmembrane protein 20 (TMEM20), mRNA [NM_153226]
UHRF1	0.042854026	2.9323897	down	1.272487	down	1.455897	down	Homo sapiens ubiquitin-like, containing PHD and RING finger domains, 1 (UHRF1), transcript variant 2, mRNA [NM_013282]

CST9L	0.008572864	2.9286344	up	1.46455	up	1.695363	down	Homo sapiens cystatin 9-like (mouse) (CST9L), mRNA [NM_080610]
C2	0.005425731	2.906241	down	1.470166	down	1.228973	up	Homo sapiens complement component 2 (C2), mRNA [NM_000063]

C7orf62	0.03373279	2.8281868	up	1.466387	up	1.326441	down	Homo sapiens hypothetical protein MGC26647 (MGC26647), mRNA [NM_152706]
HOXB13	0.008404698	2.822795	up	1.530259	up	1.061908	down	Homo sapiens homeobox B13 (HOXB13), mRNA [NM_006361]

ANLN	0.026928915	2.8110342	down	1.205709	down	1.210447	down	Homo sapiens anillin, actin binding protein (ANLN), mRNA [NM_018685]
TMEM45B	0.02836952	2.789488	up	1.108079	up	1.434473	up	Homo sapiens transmembrane protein 45B (TMEM45B), mRNA [NM_138788]

COL8A2	0.029133584	2.7760508	down	1.111961	down	2.334076	down	Homo sapiens collagen, type VIII, alpha 2 (COL8A2), mRNA [NM_005202]
IL15RA	3.56E-05	2.7710447	down	2.542192	down	5.776398	down	Homo sapiens interleukin 15 receptor, alpha (IL15RA), transcript variant 2, mRNA [NM_172200]

AY937248	0.002621024	2.7557867	up	1.074139	down	2.046131	up	Macaca mulatta placental protein 14 mRNA, complete cds [AY937248]
SCN3B	0.00199475	2.7342772	up	1.566765	up	1.26591	up	Homo sapiens sodium channel, voltage-gated, type III, beta (SCN3B), transcript variant 1, mRNA [NM_018400]

TRIM6	0.005424626	2.7181938	up	1.451755	up	1.960095	up	Homo sapiens tripartite motif-containing 6 (TRIM6), transcript variant 1, mRNA [NM_001003818]
TNS4	0.041628703	2.7158492	up	1.315606	up	1.297834	down	Homo sapiens tensin 4 (TNS4), mRNA [NM_032865]

PDE1C	0.026903268	2.6685586	up	1.596604	up	1.15087	down	Homo sapiens phosphodiesterase 1C, calmodulin-dependent 70kDa (PDE1C), mRNA [NM_005020]
S100A4	0.01836204	2.656307	down	1.047433	down	2.01034	down	Homo sapiens S100 calcium binding protein A4 (S100A4), transcript variant 1, mRNA [NM_002961]

AADACL1	0.017924123	2.6216743	down	1.119603	down	1.47176	down	Homo sapiens arylacetamide deacetylase-like 1 (AADACL1), mRNA [NM_020792]
XR_011345	0.029255124	2.6165082	up	1.450092	up	1.492756	down	PREDICTED: Macaca mulatta similar to otoancorin isoform 1 (LOC699600), mRNA [XR_011345]

FN1	0.02028952	2.6025481	down	1.05622	down	1.051482	down	Homo sapiens fibronectin 1 (FN1), transcript variant 1, mRNA [NM_212482]
SP7	3.05E-04	2.5985072	up	1.616763	up	1.088733	down	Homo sapiens Sp7 transcription factor (SP7), mRNA [NM_152860]

**Table 3:** List of top 50 genes significantly differentially regulated in liver based on fold difference (t-test).

Gene Symbol	2Way ANOVA P value	Fold Change in Liver Welch t-test	Regulation in LBW	Fold change skeletal tissue, Welch t-test	Regulation in LBW	Fold change in Cords, Welch t-test	Regulation in LBW	Gene name
ELMOD1	0.011425177	25.931845	down	1.737314	down	2.051013	up	Homo sapiens ELMO/CED-12 domain containing 1 (ELMOD1), mRNA [NM_018712]

RBBP9	0.026729036	11.012514	down	1.47388	down	5.131376	down	Homo sapiens retinoblastoma binding protein 9 (RBBP9), mRNA [NM_006606]
MMP25	0.001325307	10.996225	down	2.384582	down	1.436251	down	Homo sapiens matrix metallopeptidase 25 (MMP25), mRNA [NM_022468]

C5orf46	0.047762383	10.762607	down	1.234639	up	1.096919	up	Homo sapiens similar to AVLV472 (MGC23985), mRNA [NM_206966]
GOLSYN	2.29E-05	6.13113	up	1.05566	up	1.458383	up	Homo sapiens hypothetical protein FLJ20366 (FLJ20366), mRNA [NM_017786]

LCN15	0.005291665	5.3478875	up	1.891747	up	2.217465	up	Homo sapiens MSFL2541 (UNQ2541), mRNA [NM_203347]
CCDC146	0.030661521	5.281487	up	1.06658	up	1.360575	up	Homo sapiens KIAA1505 protein (KIAA1505), mRNA [NM_020879]

AK094929	0.001118242	5.262121	up	1.690864	up	1.110624	up	Homo sapiens cDNA FLJ37610 fis, clone BRCOC2011398. [AK094929]
SORCS3	0.00328917	5.0026994	up	2.266284	up	2.921157	up	Homo sapiens sortilin-related VPS10 domain containing receptor 3 (SORCS3), mRNA [NM_014978]

COL4A4	0.01571377	4.897748	down	1.548386	down	1.059327	down	Homo sapiens collagen, type IV, alpha 4 (COL4A4), mRNA [NM_000092]
IL1R2	0.004347455	4.777918	down	1.8715	down	1.690538	down	Homo sapiens interleukin 1 receptor, type II (IL1R2), transcript variant 1, mRNA [NM_004633]

CD200R1	0.04967409	4.7169123	down	1.052303	up	1.319852	down	Homo sapiens CD200 receptor 1 (CD200R1), transcript variant 1, mRNA [NM_138806]
GTSF1	0.017040279	4.653995	up	3.663281	up	2.487166	up	Homo sapiens family with sequence similarity 112, member B (FAM112B), mRNA [NM_144594]

SNAI1	0.005038617	4.582946	down	1.534132	down	1.847129	down	Homo sapiens snail homolog 1 (Drosophila) (SNAI1), mRNA [NM_005985]
USH1C	0.00505742	4.3276477	up	1.595945	up	1.465964	up	Homo sapiens Usher syndrome 1C (autosomal recessive, severe) (USH1C), transcript variant 1, mRNA [NM_005709]

ALLC	0.006709512	4.060955	up	1.861818	up	2.766094	up	Homo sapiens allantoicase (ALLC), transcript variant 1, mRNA [NM_018436]
CXCL3	0.04903758	3.9091544	down	1.096309	down	1.172056	up	Homo sapiens chemokine (C-X-C motif) ligand 3 (CXCL3), mRNA [NM_002090]

XR_014204	0.029225934	3.8491352	down	3.167453	down	1.084816	up	PREDICTED: Macaca mulatta hypothetical protein LOC719546 (LOC719546), mRNA [XR_014204]
XR_011794	0.010519872	3.637105	up	3.785519	up	2.76764	up	PREDICTED: Macaca mulatta similar to poly(A)-specific ribonuclease (PARN)-like domain containing 1 (LOC707835), mRNA [XR_011794]

NMNAT2	0.045685206	3.6255727	up	2.521069	down	2.189285	up	Homo sapiens nicotinamide nucleotide adenylyltransferase 2 (NMNAT2), transcript variant 1, mRNA [NM_015039]
SLC39A8	0.041505713	3.6139402	down	1.366996	down	1.390711	down	Homo sapiens solute carrier family 39 (zinc transporter), member 8 (SLC39A8), mRNA [NM_022154]

CA2	0.033340864	3.4830022	down	3.573662	down	1.194329	up	Homo sapiens carbonic anhydrase II (CA2), mRNA [NM_000067]
NTRK3	0.003890598	3.4711208	up	1.593136	up	1.043792	down	Homo sapiens neurotrophic tyrosine kinase, receptor, type 3 (NTRK3), transcript variant 1, mRNA [NM_001012338]

C17orf75	0.001362531	3.4477015	down	3.758336	down	1.443745	down	Homo sapiens chromosome 17 open reading frame 75 (C17orf75), mRNA [NM_022344]
CXCL1	0.006956845	3.392382	down	1.063934	up	4.611126	down	Homo sapiens chemokine (C-X-C motif) ligand 1 (melanoma growth stimulating activity, alpha) (CXCL1), mRNA [NM_001511]

NM_198692	0.03194352	3.3605232	down	1.343767	up	1.654617	down	Homo sapiens keratin associated protein 10-11 (KRTAP10-11), mRNA. [NM_198692]
SHANK2	0.023280138	3.3547363	up	1.255762	up	1.189415	down	Homo sapiens SH3 and multiple ankyrin repeat domains 2 (SHANK2), transcript variant 1, mRNA [NM_012309]

CCDC81	0.004982437	3.2717588	down	1.533572	down	4.912844	down	Homo sapiens coiled-coil domain containing 81 (CCDC81), mRNA [NM_021827]
CCL11	0.008992519	3.1781478	down	4.050988	down	1.002582	up	Homo sapiens chemokine (C-C motif) ligand 11 (CCL11), mRNA [NM_002986]

CO647386	0.020776557	3.1651435	down	1.030158	down	1.631509	down	ILLUMIGEN_MCQ_40418 Katze_MMPB2 Macaca mulatta cDNA clone IBIUW:21432 5' similar to Bases 185 to 778 highly similar to human CXCL2 (Hs.75765), mRNA sequence [CO647386]
GPR98	0.024752488	3.1570897	down	1.10821	down	3.919889	down	Homo sapiens G protein-coupled receptor 98 (GPR98), transcript variant 1, mRNA [NM_032119]

TMEM59L	5.34E-04	3.137047	up	1.57364	up	1.407988	up	Homo sapiens transmembrane protein 59-like (TMEM59L), mRNA [NM_012109]
UGT1A6	0.040070046	3.1353552	down	2.066639	down	1.399845	down	Homo sapiens UDP glucuronosyltransferase 1 family, polypeptide A6 (UGT1A6), transcript variant 1, mRNA [NM_001072]

KCNE2	7.02E-04	3.1135461	up	1.972823	up	1.170852	down	Homo sapiens potassium voltage-gated channel, Isk-related family, member 2 (KCNE2), mRNA [NM_172201]
XR_012376	0.022554293	3.0699794	down	1.9303	down	1.439485	down	PREDICTED: Macaca mulatta hypothetical protein LOC710335 (LOC710335), mRNA [XR_012376]

RXFP1	0.002097808	3.0400152	up	1.931833	up	1.276558	up	Homo sapiens relaxin/insulin-like family peptide receptor 1 (RXFP1), mRNA [NM_021634]
ITGBL1	0.02861261	3.0041916	down	2.563332	down	1.243521	down	Homo sapiens integrin, beta-like 1 (with EGF-like repeat domains) (ITGBL1), mRNA [NM_004791]

PASK	0.03426426	2.995584	up	5.977056	up	19.17474	up	Homo sapiens PAS domain containing serine/threonine kinase (PASK), mRNA [NM_015148]
DEFB1	0.004949556	2.9797235	down	1.065357	up	2.807533	down	Homo sapiens defensin, beta 1 (DEFB1), mRNA [NM_005218]

WDR8	0.004681234	2.9781044	down	5.68043	down	8.061375	down	Homo sapiens WD repeat domain 8 (WDR8), mRNA [NM_017818]
RIMS4	0.04112827	2.9677074	up	1.724443	up	1.024011	down	Homo sapiens regulating synaptic membrane exocytosis 4 (RIMS4), mRNA [NM_182970]

PDGFRL	0.011396675	2.9371088	down	1.21306	down	1.55284	down	Homo sapiens platelet-derived growth factor receptor-like (PDGFRL), mRNA [NM_006207]
TNRC4	0.005930619	2.9251838	up	1.208589	up	1.396738	up	Homo sapiens trinucleotide repeat containing 4 (TNRC4), mRNA [NM_007185]

UGT2B11	0.02518766	2.9193914	down	1.09908	down	2.958316	down	Homo sapiens UDP glucuronosyltransferase 2 family, polypeptide B11 (UGT2B11), mRNA [NM_001073]
FLT3LG	0.029793594	2.8908129	up	1.037797	down	1.527226	up	Homo sapiens fms-related tyrosine kinase 3 ligand (FLT3LG), mRNA [NM_001459]

IP6K3	0.03900314	2.8685477	up	1.471319	up	1.39455	down	Homo sapiens inositol hexaphosphate kinase 3 (IHPK3), mRNA [NM_054111]
ST6GALNAC1	0.03669531	2.8622296	down	1.408642	down	2.126189	down	Homo sapiens ST6 (alpha-N-acetyl-neuraminyl-2,3-beta-galactosyl-1,3)-N-acetylgalactosaminide alpha-2,6-sialyltransferase 1 (ST6GALNAC1), mRNA [NM_018414]

EFNA4	0.007086268	2.843187	down	1.084758	up	2.766163	down	Homo sapiens ephrin-A4 (EFNA4), transcript variant 1, mRNA [NM_005227]
MYST3	0.013409907	2.827006	up	3.302978	up	1.403819	up	Homo sapiens MYST histone acetyltransferase (monocytic leukemia) 3 (MYST3), mRNA [NM_006766]

XM_370715	0.001614059	2.7565975	up	1.561267	up	2.382949	up	PREDICTED: Homo sapiens similar to hypothetical protein MGC48915 (LOC387911), mRNA [XM_370715]
NM_031436	0.035783853	2.6903787	up	1.740635	up	1.698687	up	Homo sapiens aldo-keto reductase family 1, member C-like 2 (AKR1CL2), mRNA. [NM_031436]

**Table 4:** Top 50 genes significantly differentially regulated in cord based on fold difference (t-test).

Gene Symbol	2Way ANOVA P value	Fold Change in cord, Welch t-test	Regulation in LBW	Fold change skeletal tissue, Welch t-test	Regulation in LBW	Fold change in liver,	Regulation in LBW	Gene name
ATP5F1	0.003504217	29.998667	down	1.850386	down	1.153583	down	Homo sapiens ATP synthase, H+ transporting, mitochondrial F0 complex, subunit B1 (ATP5F1), nuclear gene encoding mitochondrial protein, mRNA [NM_001688]

EHHADH	3.25E-05	29.713446	down	1.004504	up	1.027032	down	Homo sapiens enoyl-Coenzyme A, hydratase/3-hydroxyacyl Coenzyme A dehydrogenase (EHHADH), mRNA [NM_001966]
CNPY2	6.12E-05	24.275322	down	2.033511	down	1.345912	down	Homo sapiens transmembrane protein 4 (TMEM4), mRNA [NM_014255]

IMMP1L	5.31E-04	21.21959	down	1.067236	down	1.052987	up	Homo sapiens IMP1 inner mitochondrial membrane peptidase-like (S. cerevisiae) (IMMP1L), mRNA [NM_144981]
GNL2	0.008845676	19.523186	down	1.389994	down	1.352022	up	Homo sapiens guanine nucleotide binding protein-like 2 (nucleolar) (GNL2), mRNA [NM_013285]

HRSP12	0.010644738	19.35189	down	1.121147	up	1.013192	up	Homo sapiens heat-responsive protein 12 (HRSP12), mRNA [NM_005836]
CN643639	2.57E-05	19.177528	down	1.710693	down	1.132373	up	ILLUMIGEN_MCQ_8235 Katze_MMBR Macaca mulatta cDNA clone IBIUW:3333 5' similar to Bases 1 to 682 highly similar to human Unigene Hs.513885, mRNA sequence [CN643639]

PASK	0.03426426	19.17474	up	5.977056	up	2.995584	up	Homo sapiens PAS domain containing serine/threonine kinase (PASK), mRNA [NM_015148]
NDUFC1	1.93E-04	18.786453	down	1.418954	down	1.095977	up	Homo sapiens NADH dehydrogenase (ubiquinone) 1, subcomplex unknown, 1, 6 kDa (NDUFC1), mRNA [NM_002494]

BNIP3L	3.67E-05	18.46857	down	2.523903	down	1.123252	down	Homo sapiens BCL2/adenovirus E1B 19 kDa interacting protein 3-like (BNIP3L), mRNA [NM_004331]
GALE	0.004030123	18.304993	down	1.591229	up	1.378757	down	Homo sapiens UDP-galactose-4-epimerase (GALE), transcript variant 1, mRNA [NM_000403]

XM_371837	2.06E-06	18.251152	down	1.76245	down	1.15429	down	PREDICTED: Homo sapiens similar to oxidoreductase UCPA (LOC389416), mRNA [XM_371837]
OSTCL	9.90E-05	17.57389	down	1.173702	down	1.075778	down	Homo sapiens similar to RIKEN cDNA 2310008M10 (LOC202459), mRNA [NM_145303]

WDR75	7.83E-05	17.02899	down	2.353145	down	1.037028	down	Homo sapiens WD repeat domain 75 (WDR75), mRNA [NM_032168]
SMPDL3A	1.25E-05	16.127317	down	1.290077	down	1.780315	down	Homo sapiens sphingomyelin phosphodiesterase, acid-like 3A (SMPDL3A), mRNA [NM_006714]

LIN7C	6.29E-04	15.929515	down	1.429568	down	1.006664	down	Homo sapiens lin-7 homolog C (C. elegans) (LIN7C), mRNA [NM_018362]
COX7B	8.03E-06	15.9135065	down	1.283232	down	1.138854	up	Homo sapiens cytochrome c oxidase subunit VIIb (COX7B), nuclear gene encoding mitochondrial protein, mRNA [NM_001866]

MRPL1	1.35E-06	15.901582	down	1.776047	down	1.05264	up	Homo sapiens mitochondrial ribosomal protein L1 (MRPL1), nuclear gene encoding mitochondrial protein, mRNA [NM_020236]
NPM3	3.58E-04	15.357531	down	1.39378	down	1.314134	up	Homo sapiens nucleophosmin/nucleoplasmin, 3 (NPM3), mRNA [NM_006993]

RNF126	4.43E-04	15.14725	down	1.165075	down	1.084156	down	Homo sapiens ring finger protein 126 (RNF126), transcript variant 2, mRNA [NM_194460]
ATG4C	1.86E-06	14.422242	down	1.01449	down	1.571663	down	Homo sapiens ATG4 autophagy related 4 homolog C (S. cerevisiae) (ATG4C), transcript variant 7, mRNA [NM_032852]

ACMSD	2.00E-04	14.3137	down	1.121731	up	1.098467	down	Homo sapiens aminocarboxymuconate semialdehyde decarboxylase (ACMSD), mRNA [NM_138326]
WASF3	0.0026955	14.211387	down	1.134297	down	1.574282	up	Homo sapiens WAS protein family, member 3 (WASF3), mRNA [NM_006646]

F13B	1.26E-05	13.94526	down	1.052562	down	1.02993	down	Homo sapiens coagulation factor XIII, B polypeptide (F13B), mRNA [NM_001994]
HNRNPA1L2	0.007305239	13.879195	down	1.288451	up	1.062584	down	Homo sapiens heterogeneous nuclear ribonucleoprotein A1-like (LOC144983), transcript variant 1, mRNA [NM_001011724]

XR_011737	0.001940294	13.592515	down	1.736661	down	1.168041	up	PREDICTED: Macaca mulatta similar to transcription factor B2, mitochondrial (LOC710669), mRNA [XR_011737]
CP	1.42E-04	13.285389	down	1.122256	down	1.274657	down	Homo sapiens ceruloplasmin (ferroxidase) (CP), mRNA [NM_000096]

TOLLIP	1.59E-04	12.683504	down	1.156986	down	1.00844	down	Homo sapiens toll interacting protein (TOLLIP), mRNA [NM_019009]
DARS	3.99E-05	12.632657	down	3.152794	down	1.10567	down	Homo sapiens aspartyl-tRNA synthetase (DARS), mRNA [NM_001349]

CKLF	4.24E-04	12.476327	down	1.130294	down	1.064736	down	Homo sapiens chemokine-like factor (CKLF), transcript variant 1, mRNA [NM_016951]
STT3B	1.13E-04	12.457355	down	2.027118	down	1.030927	down	Homo sapiens STT3, subunit of the oligosaccharyltransferase complex, homolog B (S. cerevisiae) (STT3B), mRNA [NM_178862]

TXNDC11	4.40E-05	12.377014	down	1.525444	down	1.011564	down	Homo sapiens thioredoxin domain containing 11 (TXNDC11), mRNA [NM_015914]
CR603105	9.80E-04	12.209639	down	1.284419	down	1.155491	down	full-length cDNA clone CS0DF006YN22 of Fetal brain of Homo sapiens (human) [CR603105]

XR_010672	5.09E-05	11.634537	down	1.602301	down	1.059423	up	PREDICTED: Macaca mulatta similar to Molybdenum cofactor synthesis protein 2 large subunit (Molybdopterin synthase large subunit) (MPT synthase large subunit) (MOCS2B) (MOCO1-B) (LOC703049), mRNA [XR_010672]
GDE1	0.03917646	11.536806	down	1.190901	up	1.20255	up	Homo sapiens membrane interacting protein of RGS16 (MIR16), mRNA [NM_016641]

CFHR2	2.07E-04	11.37657	down	1.208862	down	1.032327	down	Homo sapiens complement factor H-related 2 (CFHR2), mRNA [NM_005666]
RPL30	0.03067462	11.374878	down	1.173423	up	1.096937	up	Homo sapiens ribosomal protein L30 (RPL30), mRNA [NM_000989]

XM_495885	0.002429009	11.322625	down	1.165255	down	1.156577	down	PREDICTED: Homo sapiens similar to ribosomal protein S12 (LOC440055), mRNA [XM_495885]
NDUFB1	2.13E-04	11.293429	down	1.404433	down	1.277028	up	Homo sapiens NADH dehydrogenase (ubiquinone) 1 beta subcomplex, 1, 7 kDa (NDUFB1), mRNA [NM_004545]

NM_032807	2.89E-04	11.240869	down	1.075345	down	1.014724	down	Homo sapiens F-box protein, helicase, 18 (FBXO18), transcript variant 1, mRNA. [NM_032807]
CSGALNACT 2	1.34E-05	11.216295	down	1.714551	down	1.080183	down	Homo sapiens chondroitin sulfate GalNAcT-2 (GALNACT-2), mRNA [NM_018590]

NM_022333	0.002598488	10.94874	down	1.130622	up	1.014866	down	Homo sapiens TIA1 cytotoxic granule-associated RNA binding protein-like 1 (TIAL1), ranscript variant 2, mRNA [NM_022333]
NM_032807	0.001172931	10.342792	down	1.522541	down	1.139832	down	Homo sapiens F-box protein, helicase, 18 (FBXO18), transcript variant 1, mRNA. [NM_032807]

AAMP	2.62E-05	10.171815	down	1.560637	down	1.085371	down	Homo sapiens angio-associated, migratory cell protein (AAMP), mRNA [NM_001087]
ESF1	1.02E-07	9.745275	down	1.620818	down	1.008674	down	Homo sapiens ESF1, nucleolar pre-rRNA processing protein, homolog (S. cerevisiae) (ESF1), mRNA [NM_016649]

DOCK7	0.01799708	9.74066	down	1.174452	up	1.171709	up	Homo sapiens dedicator of cytokinesis 7 (DOCK7), mRNA [NM_033407]
DDX3Y	4.78E-06	9.687978	down	1.356089	down	1.134329	down	Homo sapiens DEAD (Asp-Glu-Ala-Asp) box polypeptide 3, Y-linked (DDX3Y), mRNA [NM_004660]

XIAP	3.65E-05	9.587557	down	1.882389	down	1.280735	up	Homo sapiens baculoviral IAP repeat-containing 4 (BIRC4), mRNA [NM_001167]
TRPC4AP	9.51E-05	9.4549885	down	1.340843	down	1.07043	up	Homo sapiens transient receptor potential cation channel, subfamily C, member 4 associated protein (TRPC4AP), transcript variant 1, mRNA [NM_015638]

ADK	1.63E-04	9.386349	down	5.639505	down	1.368648	down	Homo sapiens adenosine kinase (ADK), transcript variant ADK-short, mRNA [NM_001123]
NM_0010022 92	3.92E-04	9.258961	down	1.016391	up	1.220083	down	Homo sapiens chromosome 1 open reading frame 139 (C1orf139), transcript variant 2, mRNA. [NM_001002292]

Of the 250 genes which were differently expressed in the liver, 182 genes were unique to the liver (115 genes up regulated in the LBW group and 67 genes down-regulated in the LBW group). There were 19 genes which were significantly and differently expressed between liver and skeletal muscle (16 up regulated in LBW skeletal muscle and 15 genes up regulated in liver and 3 down-regulated in the LBW skeletal muscle and 4 down regulated in LBW liver). There were 5 genes which are significantly and differently expressed between liver and cord (3 up regulated in LBW liver and 2 genes up regulated in LBW cord and 2 down regulated in LBW liver and 3 down regulated in the cord).

Of the 850 genes significantly and differently expressed in skeletal muscle, 733 genes were specific to the skeletal muscle; i.e. showed altered regulation only in the skeletal muscle (584 genes up regulated in the LBW samples and 149 genes down regulated in the LBW group). There were 94 genes which were significantly and differently expressed between skeletal muscle and cord (60 up regulated in LBW skeletal muscle and 22 genes up regulated in LBW cord and 34 genes down regulated in the LBW skeletal muscle and 72 genes down regulated in the LBW cord).

Of the 891 genes significantly and differently expressed in umbilical cord; 788 genes showed altered regulation only in umbilical cord (338 genes up regulated and 450 genes down regulated in LBW group). There were 4 genes which are significantly and differently expressed between liver, skeletal muscle and umbilical cord (4 genes up regulated in the LBW skeletal muscle and liver while the same four genes were down regulated in LBW cord) Figure 2, 3.

### Functional classification of genes

Gene ontology was used to classify genes based on functional significance. The main component of the Gene Ontology (GO) annotation taken into consideration was metabolic function. Genes were classified into fifteen functional categories: Cellular lipid metabolic process (43 genes), Cellular biosynthetic process (95 genes), Cellular macromolecule synthesis (222 genes), Cellular nitrogen compound metabolic process (10 genes), Cellular carbohydrate metabolic process (6 genes), Cellular catabolic process (9 genes), Nucelobase, Nucleoside, nucleotide and nucleic acid metabolic process (216 genes), Other cellular metabolic process (29 genes), Other metabolic process (36 genes), Transport (141 genes), Regulation of molecular functions (28 genes), Biological adhesion (27 genes), Developmental process (74 genes) Other biological processes (252 genes) and Non classified (795) with p-value of (p > 0.05). The genes enriched for each GO term were further classified into the number of genes up regulated or down regulated in each tissue with a fold difference of 1.5 or greater (Table 5) (Additional file 1).

**Table 5:** Gene ontology classification to group genes using Genespring ver10 (Agilent Tech, Santa Clara) of similar functional families.

	Skeletal muscle		Liver		Cord	
	
	Up regulated	Down regulated	Up regulated	Down regulated	Up regulated	Down regulated
	
Cellular lipid metabolic process	15	6	4	4	13	11
Cellular biosynthesis process	8	14	6	4	16	40

Cellular macromolecule synthesis	21	32	19	14	45	63

Cellular nitrogen metabolic process	4	1	4	0	7	1

Cellular carbohydrate metabolic process	2	0	0	0	2	1

Cellular catabolic process	1	2	0	0	2	1

Nucleobase, Nucleoside, nucleotide and nucleic acid metabolic process	19	17	15	4	66	43

Other cellular metabolic process	3	2	1	3	3	15

Other metabolic process	5	7	5	2	9	11

Transport	30	17	21	14	39	26

Regulation of molecular functions	4	2	1	1	12	8

Biological adhesion	6	8	4	5	8	10

Developmental process	19	11	7	4	20	16

Other biological process	38	38	25	19	65	59

### Quantitative RT-PCR

To validate the micoarray results we carried out quantitative RT-PCR (qRT-PCR) using the same RNA samples in the microarray analysis. We selected seven genes (a novel gene XM_116936, PAS domain containing serine/threonine kinase (PASK), Adenisine kinase (ADK) transcript variant ADK-short, ELMO/CED-12 domain containing 1 (ELMOD1), Sine oculins homeobox homolog 1 (SIX1) Retinoblastoma like-1 (RBL1) and Solute carrier family 12 (potassium/chloride transporters) member 9 (SLC12A9) for this validation of the array using skeletal muscle cDNA. Of these 5 genes were significantly up regulated in the array and 2 genes were significantly down regulated in the array. Our results from the qPCR complement our results from the microarray (Table 6). The fold differences along with the values which derived from the microarray are presented in Table 6.

**Table 6:** Verification of seven genes from the microarray using Real-time RT-PCR analysis in skeletal muscle.

Gene Symbol and description	2 way ANOVA p-value (Birth weight)	Microarray Fold change (t-Test)	Regulation in LBW	qPCR-Fold Change
XM_116936	0.017913306	10.054285	Up regulated	
PREDICTED: *Homo sapiens *similar to RIKEN cDNA 4832428D23 gene				4.780893

PASK:	0.03426426	5.977056	Up regulated	
*Homo sapiens *PAS domain containing serine/threonine kinase				14.55481
ADK:	1.63E-04	-5.639505	Down regulated	
*Homo sapiens *adenosine kinase, (transcript variant ADK-short)				-3.57235

ELMOD1:				
*Homo sapiens *ELMO/CED-12 domain containing 1.	0.011425177	-1.7373136	Down regulated	-1.71015
SIX1:				
*Homo sapiens *sine oculis homeobox homolog 1 (*Drosophila*).	0.045728132	1.1253903	Up in low birth weight	1.246574

RBL1:				
*Homo sapiens *retinoblastoma-like 1 (p107), (transcript variant 1)	0.011705314	1.5320477	Up regulated	3.023726
SLC12A9:				
*Homo sapiens *solute carrier family 12 (potassium/chloride transporters), member 9.	1.31E-05	1.3670695	Up regulated	1.694303

## Discussion

In the present study, we have identified genes involved in key metabolic signaling pathways in three tissue types in a non-human primate model, that were differentially expressed according to the birth weight of the animal. Importantly, this differential expression was across the normal birth weight spectrum and therefore likely to represent adaptive pathways that the fetuses uses to predict its postnatal environment. The identification of novel signaling pathways that appear to be regulated by the early life environment is a key step in designing future experimental paradigms to understand the association between birth weight and disease risk. Metabolic disease particularly, has been strongly with early life adversity [21,22]. Our data begin to shed light on the key signaling pathways that are vulnerable to subtle changes in the early life environment.

The strength of our study, despite its small size, is that we have focused on infants whose growth was not experimentally manipulated but lay within normal birth weight range. Many experimental models have manipulated pregnancy in an effort to produce fetal growth restriction. Such studies have shown that offspring which are born growth restricted catch-up in growth with their normally nourished counterparts and in adulthood are obese, hypertensive, hyperinsulinemic, leptin resistant and display sedentary behavior [23-25]. Investigations into underlying mechanisms and the determination of gene expression levels that may explain these altered phenotypes have produced conflicting results [26-28] which may reflect variations in the model systems used and the gender of the animals used [28]. Taken together, although these studies have established the link between early life nutritional adversity to later pathophysiology, there are limitations in the interpretation of rodent studies in development as applied to humans.

In the present study we aimed to study the molecular associates of growth variation within the normative range and exclude pathology. This is because the growing literature on developmental outcomes highlights that the importance of variation in risk is associated with non-pathological developmental environments. Accordingly we studied relatively small infants born between the 5^th ^and 25^th ^centile but excluded the smallest neonates, which may reflect obstetrical abnormalities. These infants were compared to infants in the middle of the normative range (50^th^-75^th ^centile) and accordingly we excluded infants who may have had macrosomia as a result of the mother's being over-nourished by being maintained in captivity. Thus we are confident that we have excluded pathological influences and demonstrated that within the normative range patterns of gene expression may vary considerably with variable birth weights. Indeed we found a number of genes with more than a 10 fold shift in expression levels. There are important implications to this observation. Historically, experimental and epidemiological focus has been on the extremes of birth weight (either small for gestational age or large for gestational age) and there has been little focus within the normal birth weight range continuum. What is evident from the present study is that relatively small changes in the birth phenotype may be associated with profound changes in molecular physiology. In turn this also suggests the presence of highly evolved processes by which the fetus can adjust its development in response to subtle cues from mother [29].

The Cynomolgus, as in the human, has monotocous pregnancies with haemochorial placentae; they have omnivorous diets and monogastric digestion. They also share with humans the same progressive history of the metabolic syndrome [17]. We have identified alterations in the levels and expression patterns of a number of genes involved in different metabolic processes including cellular lipid metabolism, cellular biosynthesis, cellular macromolecule synthesis, cellular nitrogen metabolism, cellular carbohydrate metabolism, cellular catabolism, nucleotide and nucleic acid metabolism, biological adhesion and development.Recently, transcriptional profiling in rats subjected to gestational under nutrition was performed in young adult male rats where 249 genes were shown to be differentially expressed in the liver [28]. We compared the genes which are significantly altered in our array with those identified in the rat array study and have identified twelve similar or closely related genes from those identified in the rat: Tribbles homolog 2 (Trib2); 3-hydroxyanthanillate dioxygenase (Haao), transmembrane serine protease 6 (Tmmprss6); Thioredoxin domain containing10 (Txndc10); tralation initiation factor 4A3 (Eifa3); Ribosomal protein L31 (Rpl31); Danse 1-like 2 (Dnase1l2); Quinolate phosphoribosyl transferase (Qprt); general transcription factor IIa 2 (Gtf2a3); General transcription factor II H 3 [28]. Only four of these genes (Trib2, Trip10, EIF4A3 and Dnase1l2) were altered in the same direction in the livers of LBW macaques as in the rat array. We have also compared our observations with those identified from LBW term placentas by McCarthy and colleagues [30] and found similarities in expression changes in the genes Procollagen-lysine, 2-oxoglutarate 5-dioygense (PLOD2); Soluble interleukin-1 receptor accessory protein (IL1RAP); Solute carrier family 2 (facilitated glucose transporter) member3 (SLC2A3); Myosin V1(MYH6); Ribosomal protein S6(RPS6) and Latexin (LXN). The Tribbles homolog 2 (Trib2) is also increased in the LBW infants and suggests another possible way in way insulin/IGF-1 signalling might be impaired during development. Tribbles belongs to a family of kinase-like proteins and are reported to be negative regulators of Akt, the principle target of insulin signaling [31,32].

Studies using animal models such as rodents to understand the developmental origins of metabolic diseases have shown that epigenetic changes in genes correlates with expression changes including metabolic enzymes such as PEPCK, transcriptional factors such as PPARα which regulate fat metabolism and factors associated with insulin action (e.g. PI3 kinase, PKC-ζ), the key regulatory genes which are responsible for bringing these changes are not known [15,33]. One aim in our study was to identify whether there were shifts within the expression of key early regulators and from the array we have identified one such key regulatory gene the PAS Kinase (PASK), an evolutionarily conserved PAS domain containing serine/threonine kinase whose expression is altered as a result of adverse early developmental conditions. PAS domains are evolutionarily conserved and appear from archaea, bacteria to eukaryotes and are present in many signaling proteins where they act as signal sensor domain [34]. The PASK gene, whereby expression was up regulated in the LBW animals, has been shown to be a metabolic sensor based on mice knock-out studies; mice lacking PASK are resistant to high-fat induced obesity, hepatic steatosis and are resistant to insulin [35].

## Conclusions

In summary, this paper has identified significant variation in gene expression in multiple tissues in primate newborns of different growth trajectories but within the normative range of birth size. Further detailed analyses may improve our understanding of how alterations in such genes due to adverse early life environment predisposes towards metabolic syndrome. These data give strength to the hypothesis that developmental plasticity operating within the normative range of birth weights can influence metabolic and other physiological systems in a way that might have later health consequences. It emphasizes that the concept of developmental programming need not involve pathological changes in growth trajectories to have molecular and presumably functional consequences.

## Competing interests

The authors declare that they have no competing interests.

## Authors' contributions

BSE, RK and PDG conceived the study. KC, DMS and MHV coordinated and collected the animal samples. BSE and SM undertook the molecular biology. BSE and PDG wrote the manuscript. BSE, KC, DMS, SM, MHV, RK and PDG reviewed/edited manuscript. All authors read and approved the final manuscript.

## Supplementary Material

Additional file 1 **Genes which were differentially regulated between the ABW and LBW groups classified based on GO terms**. Genes with a ≥ 1.5 fold change at least in one tissue with a p value of ≥ 0.5 identified from the array with the GO terms Click here for file
