# Supplementary material for: Gene expression profiling in the Cynomolgus macaque Macaca fascicularis shows variation within the normal birth range
Source: BMC Genomics. 2011 Oct 16;12:509. doi: 10.1186/1471-2164-12-509 (PMC3210194; doi:10.1186/1471-2164-12-509)
Supplement: Additional file 1 — Genes which were differentially regulated between the ABW and LBW groups classified based on GO terms. Genes with a ≥ 1.5 fold change at least in one tissue with a p value of ≥ 0.5 identified from the array with the GO terms [file 1471-2164-12-509-S1.DOC]

| Gene Symbol | Description | Genbank Accession | 2Way ANOVA p-value -LBW | Fold Change t-test (in LBW) | | |
| --- | --- | --- | --- | --- | --- | --- |
|  |  |  |  | Skeletal muscle | LIVER | CORDS |
| **METABOLIC PROCESS** | |  |  |  |  |  |
| **Cellular Lipid Metabolic Process** |  |  |  |  |  |  |
| SELI | selenoprotein I (SELI) | NM_033505 | 0.04013 | -1.26 | -1.01 | -1.76 |
| PIGX | phosphatidylinositol glycan anchor biosynthesis, class X (PIGX) | NM_017861 | 0.01508 | -1.23 | -1.16 | -1.57 |
| PIGP | phosphatidylinositol glycan anchor biosynthesis, class P (PIGP), transcript variant 2 | NM_153682 | 0.02289 | -1.11 | -1.27 | -1.34 |
| SMPDL3A | sphingomyelin phosphodiesterase, acid-like 3A (SMPDL3A) | NM_006714 | 0.00001 | -1.29 | -1.78 | -16.13 |
| DPM1 | dolichyl-phosphate mannosyltransferase polypeptide 1, catalytic subunit (DPM1) | NM_003859 | 0.00348 | -1.40 | -1.11 | -1.44 |
| UGT2B11 | UDP glucuronosyltransferase 2 family, polypeptide B11 (UGT2B11) | NM_001073 | 0.02519 | -1.10 | -2.92 | -2.96 |
| PECR | peroxisomal trans-2-enoyl-CoA reductase (PECR) | NM_018441 | 0.00019 | -1.55 | -1.01 | -6.20 |
| LIPC | lipase, hepatic (LIPC) | NM_000236 | 0.02210 | -2.36 | -1.56 | -2.75 |
| PIGP | phosphatidylinositol glycan anchor biosynthesis, class P (PIGP), transcript variant 2 | NM_153682 | 0.03427 | -1.12 | -1.24 | -1.31 |
| ELOVL3 | elongation of very long chain fatty acids (FEN1/Elo2, SUR4/Elo3, yeast)-like 3 (ELOVL3) | NM_152310 | 0.00413 | -2.44 | -2.43 | -2.02 |
| EHHADH | enoyl-Coenzyme A, hydratase/3-hydroxyacyl Coenzyme A dehydrogenase (EHHADH) | NM_001966 | 0.00003 | 1.00 | -1.03 | -29.71 |
| ADH5 | alcohol dehydrogenase 5 (class III), chi polypeptide (ADH5) | NM_000671 | 0.00687 | -1.80 | 1.05 | -2.33 |
| CHPT1 | choline phosphotransferase 1 (CHPT1) | NM_020244 | 0.00001 | -1.55 | 1.05 | -8.14 |
| PIGK | phosphatidylinositol glycan anchor biosynthesis, class K (PIGK) | NM_005482 | 0.00526 | -1.54 | 1.00 | -2.36 |
| IP6K3 | inositol hexaphosphate kinase 3 (IHPK3) | NM_054111 | 0.03900 | 1.47 | 2.87 | -1.39 |
| SLC27A1 | solute carrier family 27 (fatty acid transporter), member 1 (SLC27A1) | NM_198580 | 0.03701 | 1.62 | -1.01 | 1.35 |
| PI4KB | phosphatidylinositol 4-kinase, catalytic, beta polypeptide (PIK4CB) | NM_002651 | 0.01993 | -1.01 | 1.19 | 1.54 |
| HSD3B7 | hydroxy-delta-5-steroid dehydrogenase, 3 beta- and steroid delta-isomerase 7 (HSD3B7) | NM_025193 | 0.02373 | 1.48 | 1.37 | 2.08 |
| STAT5A | signal transducer and activator of transcription 5A (STAT5A) | NM_003152 | 0.00521 | 1.48 | 1.15 | 1.29 |
| PLA2G15 | lysophospholipase 3 (lysosomal phospholipase A2) (LYPLA3) | NM_012320 | 0.02715 | 1.36 | 1.03 | 1.33 |
| CYP21A2 | cytochrome P450, family 21, subfamily A, polypeptide 2 (CYP21A2) | NM_000500 | 0.01313 | 2.26 | 1.26 | 1.17 |
| SHH | sonic hedgehog homolog (Drosophila) (SHH) | NM_000193 | 0.00498 | 1.50 | 1.15 | 1.44 |
| LEPR | leptin receptor (LEPR), transcript variant 2 | NM_001003679 | 0.02923 | 1.25 | 2.06 | 1.22 |
| PLCB3 | phospholipase C, beta 3 (phosphatidylinositol-specific) (PLCB3) | NM_000932 | 0.00418 | 1.60 | 1.20 | 1.57 |
| ST6GALNAC6 | ST6 (alpha-N-acetyl-neuraminyl-2,3-beta-galactosyl-1,3)-N-acetylgalactosaminide alpha-2,6-sialyltransferase 6 (ST6GALNAC6) | NM_013443 | 0.03583 | 1.75 | 1.11 | 1.41 |
| ECHS1 | enoyl Coenzyme A hydratase, short chain, 1, mitochondrial (ECHS1), nuclear gene encoding mitochondrial protein | NM_004092 | 0.00915 | 1.45 | 1.34 | 1.12 |
| SPHK2 | sphingosine kinase 2 (SPHK2) | NM_020126 | 0.02176 | 1.19 | 1.16 | 2.13 |
| FASN | fatty acid synthase (FASN) | NM_004104 | 0.02721 | 1.17 | 1.17 | 1.60 |
| SCAP | SREBF chaperone (SCAP) | NM_012235 | 0.01836 | 1.39 | 1.15 | 1.51 |
| RNPEP | arginyl aminopeptidase (aminopeptidase B) (RNPEP) | NM_020216 | 0.01955 | 1.39 | 1.38 | 1.29 |
| AGPAT1 | 1-acylglycerol-3-phosphate O-acyltransferase 1 (lysophosphatidic acid acyltransferase, alpha) (AGPAT1), transcript variant 1 | NM_006411 | 0.01401 | 1.27 | 1.26 | 1.21 |
| SOAT2 | sterol O-acyltransferase 2 (SOAT2) | NM_003578 | 0.00238 | 1.97 | 1.70 | 1.09 |
| FAAH | fatty acid amide hydrolase (FAAH) | NM_001441 | 0.02126 | 1.01 | 1.43 | 1.97 |
| PIGZ | phosphatidylinositol glycan anchor biosynthesis, class Z (PIGZ) | NM_025163 | 0.02283 | 1.40 | 1.96 | 1.62 |
| CPT1C | carnitine palmitoyltransferase 1C (CPT1C) | NM_152359 | 0.00023 | 1.54 | 1.37 | 1.90 |
| PERLD1 | per1-like domain containing 1 (PERLD1) | NM_033419 | 0.00491 | 1.09 | 1.19 | 1.48 |
| PLCD1 | phospholipase C, delta 1 (PLCD1) | NM_006225 | 0.04107 | 1.11 | 1.01 | 1.51 |
| CLN6 | ceroid-lipofuscinosis, neuronal 6, late infantile, variant (CLN6) | NM_017882 | 0.02853 | 1.63 | 1.33 | 1.09 |
| EPHX2 | epoxide hydrolase 2, cytoplasmic (EPHX2) | NM_001979 | 0.03870 | 1.74 | 1.20 | 1.25 |
| OSBPL2 | oxysterol binding protein-like 2 (OSBPL2), transcript variant 2 | NM_144498 | 0.00626 | 1.41 | 1.18 | 1.56 |
| TAZ | tafazzin (cardiomyopathy, dilated 3A (X-linked); endocardial fibroelastosis 2; Barth syndrome) (TAZ), transcript variant 1 | NM_000116 | 0.00895 | 1.16 | 1.18 | 1.24 |
| PLCG1 | phospholipase C, gamma 1 (PLCG1), transcript variant 1 | NM_002660 | 0.00140 | 1.31 | 1.16 | 1.76 |
| FADS3 | fatty acid desaturase 3 (FADS3) | NM_021727 | 0.01173 | 1.63 | 1.06 | 1.80 |
| **Cellular Biosynthesis Process** | |  |  |  |  |  |
| CDO1 | cysteine dioxygenase, type I (CDO1) | NM_001801 | 0.00744 | -1.93 | -1.91 | -1.87 |
| MRPL16 | mitochondrial ribosomal protein L16 (MRPL16), nuclear gene encoding mitochondrial protein | NM_017840 | 0.01215 | -1.09 | -1.01 | -1.27 |
| RPL17 | ribosomal protein L17 (RPL17), transcript variant 1 | NM_000985 | 0.00019 | -1.09 | -1.09 | -4.77 |
| CPOX | coproporphyrinogen oxidase (CPOX) | NM_000097 | 0.01090 | -1.83 | -1.47 | -1.55 |
| LIAS | lipoic acid synthetase (LIAS), nuclear gene encoding mitochondrial protein, transcript variant 1 | NM_006859 | 0.04067 | -1.29 | -1.05 | -1.52 |
| MRPL32 | mitochondrial ribosomal protein L32 (MRPL32), nuclear gene encoding mitochondrial protein | NM_031903 | 0.00163 | -1.60 | -1.53 | -4.02 |
| GTF3C6 | chromosome 6 open reading frame 51 (C6orf51) | NM_138408 | 0.01174 | -1.14 | -1.07 | -1.52 |
| MRPL47 | mitochondrial ribosomal protein L47 (MRPL47), nuclear gene encoding mitochondrial protein, transcript variant 1 | NM_020409 | 0.02698 | -1.20 | -1.06 | -2.25 |
| RPS3A | ribosomal protein S3A (RPS3A) | NM_001006 | 0.03238 | -1.07 | -1.20 | -7.27 |
| RPL17 | ribosomal protein L17 (RPL17), transcript variant 1 | NM_000985 | 0.03152 | -1.01 | -1.19 | -2.28 |
| DARS | aspartyl-tRNA synthetase (DARS) | NM_001349 | 0.00004 | -3.15 | -1.11 | -12.63 |
| SEPSECS | soluble liver antigen/liver pancreas antigen (SLA/LP), transcript variant 1 | NM_016955 | 0.04468 | -1.15 | -1.09 | -1.14 |
| GCH1 | GTP cyclohydrolase 1 (dopa-responsive dystonia) (GCH1), transcript variant 1 | NM_000161 | 0.03241 | -1.37 | -1.54 | -1.35 |
| DSE | dermatan sulfate epimerase (DSE), transcript variant 1 | NM_013352 | 0.02913 | -1.88 | -1.49 | -1.17 |
| AMD1 | adenosylmethionine decarboxylase 1 (AMD1), transcript variant 1 | NM_001634 | 0.00950 | -1.56 | -1.11 | -1.63 |
| ALDH18A1 | aldehyde dehydrogenase 18 family, member A1 (ALDH18A1), nuclear gene encoding mitochondrial protein, transcript variant 1 | NM_002860 | 0.01477 | -1.14 | -1.22 | -1.33 |
| NR3C1 | nuclear receptor subfamily 3, group C, member 1 (glucocorticoid receptor) (NR3C1), transcript variant 5 | NM_000176 | 0.02426 | -1.21 | -1.08 | -1.72 |
| PRIM1 | primase, polypeptide 1, 49kDa (PRIM1) | NM_000946 | 0.00104 | -1.13 | -1.24 | -2.51 |
| MRPS30 | mitochondrial ribosomal protein S30 (MRPS30), nuclear gene encoding mitochondrial protein | NM_016640 | 0.03625 | -1.69 | -1.11 | -1.16 |
| MED30 | thyroid hormone receptor associated protein 6 (THRAP6) | NM_080651 | 0.00179 | -1.19 | -1.21 | -2.48 |
| DARS2 | aspartyl-tRNA synthetase 2, mitochondrial (DARS2) | NM_018122 | 0.01357 | -1.36 | -1.17 | -1.20 |
| MRPS10 | mitochondrial ribosomal protein S10 (MRPS10), nuclear gene encoding mitochondrial protein | NM_018141 | 0.00123 | -1.35 | -1.03 | -1.50 |
| COPS5 | COP9 constitutive photomorphogenic homolog subunit 5 (Arabidopsis) (COPS5) | NM_006837 | 0.00891 | -1.21 | -1.02 | -1.51 |
| GFM1 | G elongation factor, mitochondrial 1 (GFM1), nuclear gene encoding mitochondrial protein | NM_024996 | 0.01757 | -1.49 | -1.09 | -1.34 |
| GTF2H3 | general transcription factor IIH, polypeptide 3, 34kDa (GTF2H3) | NM_001516 | 0.00992 | -1.50 | -1.01 | -1.78 |
| EIF4E | eukaryotic translation initiation factor 4E (EIF4E) | NM_001968 | 0.01186 | -1.73 | -1.18 | -1.23 |
| MAT2B | methionine adenosyltransferase II, beta (MAT2B), transcript variant 2 | NM_182796 | 0.00357 | -1.32 | -1.30 | -2.06 |
| PMM2 | phosphomannomutase 2 (PMM2) | NM_000303 | 0.04478 | -1.29 | -1.06 | -1.11 |
| RPS10 | ribosomal protein S10 (RPS10) | NM_001014 | 0.03580 | -1.04 | -1.15 | -1.64 |
| FUBP1 | far upstream element (FUSE) binding protein 1 (FUBP1) | NM_003902 | 0.00035 | -1.32 | -1.34 | -1.56 |
| RPL10L | ribosomal protein L10-like (RPL10L) | NM_080746 | 0.03909 | -1.05 | -1.05 | -5.66 |
| HIF1A | hypoxia-inducible factor 1, alpha subunit (basic helix-loop-helix transcription factor) (HIF1A), transcript variant 2 | NM_181054 | 0.04619 | -1.36 | -1.34 | -1.05 |
| RPS6 | ribosomal protein S6 (RPS6) | NM_001010 | 0.00472 | -1.10 | -1.12 | -3.33 |
| BMPR2 | bone morphogenetic protein receptor, type II (serine/threonine kinase) (BMPR2) | NM_001204 | 0.03033 | -1.38 | -1.15 | -1.46 |
| GTF2A2 | general transcription factor IIA, 2 (12kD subunit) (GTF2A2) | NM_004492 | 0.04831 | -1.04 | -1.01 | -1.43 |
| PTTG1 | pituitary tumor-transforming 1 (PTTG1) | NM_004219 | 0.02039 | -1.85 | -1.15 | -1.57 |
| CCNH | cyclin H (CCNH) | NM_001239 | 0.01499 | -1.42 | -1.04 | -1.63 |
| CSGALNACT2 | chondroitin sulfate GalNAcT-2 (GALNACT-2) | NM_018590 | 0.00001 | -1.71 | -1.08 | -11.22 |
| KYNU | kynureninase (L-kynurenine hydrolase) (KYNU), transcript variant 1 | NM_003937 | 0.00013 | 1.09 | -2.38 | -6.96 |
| MARS | methionyl-tRNA synthetase (MARS) | NM_004990 | 0.01862 | -1.24 | 1.00 | -1.68 |
| PSTK | chromosome 10 open reading frame 89 (C10orf89) | NM_153336 | 0.00014 | -1.23 | 1.01 | -5.49 |
| PPAT | phosphoribosyl pyrophosphate amidotransferase (PPAT) | NM_002703 | 0.04292 | -1.07 | 1.00 | -1.66 |
| THRAP3 | thyroid hormone receptor associated protein 3 (THRAP3) | NM_005119 | 0.00512 | -1.38 | 1.09 | -2.98 |
| POLR2C | polymerase (RNA) II (DNA directed) polypeptide C, 33kDa (POLR2C) | NM_032940 | 0.00025 | -1.47 | 1.04 | -2.90 |
| COQ6 | coenzyme Q6 homolog, monooxygenase (S. cerevisiae) (COQ6), transcript variant 1 | NM_182476 | 0.01303 | -1.18 | 1.24 | -1.95 |
| MRPL15 | mitochondrial ribosomal protein L15 (MRPL15), nuclear gene encoding mitochondrial protein | NM_014175 | 0.02976 | -1.26 | 1.03 | -1.74 |
| MRPL19 | mitochondrial ribosomal protein L19 (MRPL19), nuclear gene encoding mitochondrial protein | NM_014763 | 0.03391 | -1.32 | 1.04 | -1.22 |
| LGTN | ligatin (LGTN) | NM_006893 | 0.00569 | -1.09 | 1.39 | -7.99 |
| PSPH | phosphoserine phosphatase (PSPH) | NM_004577 | 0.00009 | -1.62 | 1.09 | -3.65 |
| RPS7 | ribosomal protein S7 (RPS7) | NM_001011 | 0.00007 | -1.13 | 1.01 | -5.45 |
| MRPL1 | mitochondrial ribosomal protein L1 (MRPL1), nuclear gene encoding mitochondrial protein | NM_020236 | 0.00000 | -1.78 | 1.05 | -15.90 |
| NPM3 | nucleophosmin/nucleoplasmin, 3 (NPM3) | NM_006993 | 0.00036 | -1.39 | 1.31 | -15.36 |
| EXT1 | exostoses (multiple) 1 (EXT1) | NM_000127 | 0.00038 | -1.37 | 1.11 | -3.09 |
| RPL30 | ribosomal protein L30 (RPL30) | NM_000989 | 0.03067 | 1.17 | 1.10 | -11.37 |
| PPCDC | phosphopantothenoylcysteine decarboxylase (PPCDC) | NM_021823 | 0.04747 | 1.98 | 1.11 | -1.13 |
| MLLT1 | myeloid/lymphoid or mixed-lineage leukemia (trithorax homolog, Drosophila); translocated to, 1 (MLLT1) | NM_005934 | 0.03810 | 1.11 | -1.01 | 1.88 |
| NMNAT2 | nicotinamide nucleotide adenylyltransferase 2 (NMNAT2), transcript variant 1 | NM_015039 | 0.04569 | -2.52 | 3.63 | 2.19 |
| GTF3C1 | general transcription factor IIIC, polypeptide 1, alpha 220kDa (GTF3C1) | NM_001520 | 0.02332 | -1.02 | 1.20 | 1.30 |
| HAAO | 3-hydroxyanthranilate 3,4-dioxygenase (HAAO) | NM_012205 | 0.03975 | -1.04 | 1.45 | 1.63 |
| PC | pyruvate carboxylase (PC), nuclear gene encoding mitochondrial protein, transcript variant 1 | NM_000920 | 0.01504 | -1.03 | 1.77 | 1.45 |
| MTHFSD | methenyltetrahydrofolate synthetase domain containing (MTHFSD) | NM_022764 | 0.03445 | -1.00 | 1.22 | 1.64 |
| AARSD1 | alanyl-tRNA synthetase domain containing 1 (AARSD1) | NM_025267 | 0.00066 | 1.32 | 1.36 | 1.40 |
| CDK9 | cyclin-dependent kinase 9 (CDC2-related kinase) (CDK9) | NM_001261 | 0.00637 | 1.50 | 1.54 | 1.70 |
| BCAT2 | branched chain aminotransferase 2, mitochondrial (BCAT2) | NM_001190 | 0.04320 | 1.32 | 1.15 | 1.07 |
| TNIP1 | TNFAIP3 interacting protein 1 (TNIP1) | NM_006058 | 0.01449 | 1.41 | 1.01 | 1.46 |
| MED12 | mediator of RNA polymerase II transcription, subunit 12 homolog (S. cerevisiae) (MED12) | NM_005120 | 0.00405 | 1.27 | 1.20 | 1.31 |
| FOXO4 | myeloid/lymphoid or mixed-lineage leukemia (trithorax homolog, Drosophila); translocated to, 7 (MLLT7) | NM_005938 | 0.02019 | 1.11 | 1.21 | 1.72 |
| QPRT | quinolinate phosphoribosyltransferase (nicotinate-nucleotide pyrophosphorylase (carboxylating)) (QPRT) | NM_014298 | 0.00154 | 1.20 | 1.43 | 1.72 |
| GAMT | guanidinoacetate N-methyltransferase (GAMT), transcript variant 2 | NM_138924 | 0.01572 | 1.07 | 1.36 | 1.38 |
| PSMC5 | proteasome (prosome, macropain) 26S subunit, ATPase, 5 (PSMC5) | NM_002805 | 0.01361 | 1.04 | 1.19 | 1.40 |
| EIF2B1 | eukaryotic translation initiation factor 2B, subunit 1 alpha, 26kDa (EIF2B1) | NM_001414 | 0.02494 | 1.03 | 1.26 | 1.22 |
| ASMTL | acetylserotonin O-methyltransferase-like (ASMTL) | NM_004192 | 0.01114 | 1.24 | 1.15 | 1.45 |
| MRPS6 | mitochondrial ribosomal protein S6 (MRPS6), nuclear gene encoding mitochondrial protein | NM_032476 | 0.00679 | 1.58 | 1.24 | 1.06 |
| QDPR | quinoid dihydropteridine reductase (QDPR) | NM_000320 | 0.03993 | 1.20 | 1.51 | 1.10 |
| TCEB2 | transcription elongation factor B (SIII), polypeptide 2 (18kDa, elongin B) (TCEB2), transcript variant 1 | NM_007108 | 0.01692 | 1.35 | 1.34 | 1.16 |
| BHMT2 | betaine-homocysteine methyltransferase 2 (BHMT2) | NM_017614 | 0.00073 | 1.99 | 1.38 | 2.38 |
| GGT7 | gamma-glutamyltransferase-like 3 (GGTL3) | NM_178026 | 0.03586 | 1.17 | 1.28 | 1.45 |
| NECAB3 | amyloid beta (A4) precursor protein-binding, family A, member 2 binding protein (APBA2BP), transcript variant 2 | NM_031232 | 0.00006 | 1.10 | 1.18 | 2.58 |
| HS6ST1 | heparan sulfate 6-O-sulfotransferase 1 (HS6ST1) | NM_004807 | 0.03730 | 1.38 | 1.21 | 1.87 |
| DEAF1 | deformed epidermal autoregulatory factor 1 (Drosophila) (DEAF1) | NM_021008 | 0.00096 | 1.28 | 1.08 | 1.56 |
| HTT | huntingtin (Huntington disease) (HD) | NM_002111 | 0.02473 | 1.35 | 1.26 | 2.08 |
| NDST1 | N-deacetylase/N-sulfotransferase (heparan glucosaminyl) 1 (NDST1) | NM_001543 | 0.02213 | 1.45 | 1.02 | 1.44 |
| DMD | dystrophin (muscular dystrophy, Duchenne and Becker types) (DMD), transcript variant Dp427p2 | NM_004010 | 0.01784 | 1.29 | 1.47 | 1.32 |
| RPL10 | ribosomal protein L10 (RPL10) | NM_006013 | 0.02253 | 1.56 | 1.26 | 1.01 |
| NFRKB | nuclear factor related to kappaB binding protein (NFRKB) | NM_006165 | 0.01765 | 1.28 | 1.05 | 1.39 |
| ADI1 | acireductone dioxygenase 1 (ADI1) | NM_018269 | 0.04041 | 1.99 | 1.37 | 1.27 |
| ALDH4A1 | aldehyde dehydrogenase 4 family, member A1 (ALDH4A1), nuclear gene encoding mitochondrial protein, transcript variant P5CDhL | NM_003748 | 0.04015 | 1.36 | 1.01 | 1.71 |
| EIF3G | eukaryotic translation initiation factor 3, subunit 4 delta, 44kDa (EIF3S4) | NM_003755 | 0.02879 | 1.30 | 1.12 | 1.34 |
| GGT1 | gamma-glutamyltransferase 1 (GGT1), transcript variant 3 | NM_013430 | 0.02327 | 1.69 | 1.21 | 1.15 |
| EIF3G | eukaryotic translation initiation factor 3, subunit 4 delta, 44kDa (EIF3S4) | NM_003755 | 0.02266 | 1.44 | 1.08 | 1.25 |
| ADC | arginine decarboxylase (ADC) | NM_052998 | 0.03035 | 1.00 | 2.35 | 1.35 |
| NADSYN1 | NAD synthetase 1 (NADSYN1) | NM_018161 | 0.01135 | 1.27 | 1.40 | 1.33 |
| ABTB1 | ankyrin repeat and BTB (POZ) domain containing 1 (ABTB1), transcript variant 2 | NM_172027 | 0.02553 | 1.44 | 1.12 | 1.74 |
| NAGS | N-acetylglutamate synthase (NAGS) | NM_153006 | 0.00027 | 2.17 | 1.81 | 2.68 |
| SNAPC4 | small nuclear RNA activating complex, polypeptide 4, 190kDa (SNAPC4) | NM_003086 | 0.01740 | 1.27 | 1.22 | 1.53 |
| **Cellular Macromolecule Synthesis** | |  |  |  |  |  |
| BUB1B | BUB1 budding uninhibited by benzimidazoles 1 homolog beta (yeast) (BUB1B) | NM_001211 | 0.02662 | -2.09 | -1.91 | -1.22 |
| MAP2K1 | mitogen-activated protein kinase kinase 1 (MAP2K1) | NM_002755 | 0.04911 | -1.14 | -1.14 | -1.12 |
| GLRX2 | glutaredoxin 2 (GLRX2), transcript variant 1 | NM_016066 | 0.00710 | -1.67 | -1.19 | -1.81 |
| ANAPC10 | anaphase promoting complex subunit 10 (ANAPC10) | NM_014885 | 0.04949 | -1.18 | -1.04 | -1.42 |
| DPH5 | DPH5 homolog (S. cerevisiae) (DPH5), transcript variant 2 | NM_015958 | 0.01917 | -1.47 | -1.13 | -1.34 |
| CFH | complement factor H (CFH), transcript variant 1 | NM_000186 | 0.04685 | -1.71 | -1.06 | -1.64 |
| DUSP11 | dual specificity phosphatase 11 (RNA/RNP complex 1-interacting) (DUSP11) | NM_003584 | 0.04306 | -1.07 | -1.46 | -1.49 |
| FN1 | fibronectin 1 (FN1), transcript variant 1 | NM_212482 | 0.02029 | -2.60 | -1.06 | -1.05 |
| PGK1 | phosphoglycerate kinase 1 (PGK1) | NM_000291 | 0.00990 | -1.55 | -1.20 | -1.69 |
| CAMK1 | calcium/calmodulin-dependent protein kinase I (CAMK1) | NM_003656 | 0.03578 | -1.31 | -1.22 | -1.54 |
| HIPK1 | homeodomain interacting protein kinase 1 (HIPK1), transcript variant 1 | NM_198268 | 0.00503 | -1.24 | -1.16 | -1.39 |
| TGFA | transforming growth factor, alpha (TGFA) | NM_003236 | 0.03580 | -1.40 | -1.86 | -1.29 |
| ST6GALNAC1 | ST6 (alpha-N-acetyl-neuraminyl-2,3-beta-galactosyl-1,3)-N-acetylgalactosaminide alpha-2,6-sialyltransferase 1 (ST6GALNAC1) | NM_018414 | 0.03670 | -1.41 | -2.86 | -2.13 |
| FUT8 | fucosyltransferase 8 (alpha (1,6) fucosyltransferase) (FUT8), transcript variant 2 | NM_178154 | 0.04590 | -1.01 | -1.30 | -1.51 |
| GALNT9 | UDP-N-acetyl-alpha-D-galactosamine:polypeptide N-acetylgalactosaminyltransferase 9 (GalNAc-T9) (GALNT9) | NM_021808 | 0.03179 | -1.28 | -1.91 | -1.47 |
| FKBP14 | FK506 binding protein 14, 22 kDa (FKBP14) | NM_017946 | 0.00859 | -1.25 | -1.06 | -1.94 |
| UBE2NL | ubiquitin-conjugating enzyme E2N-like (UBE2NL) | NM_001012989 | 0.00606 | -1.77 | -1.11 | -1.98 |
| CSNK1A1 | casein kinase 1, alpha 1 (CSNK1A1), transcript variant 2 | NM_001892 | 0.01893 | -1.25 | -1.15 | -1.39 |
| ZYG11B | zyg-11 homolog B (C. elegans) (ZYG11B) | NM_024646 | 0.04132 | -1.43 | -1.22 | -1.23 |
| CCNB1 | cyclin B1 (CCNB1) | NM_031966 | 0.00414 | -2.13 | -1.78 | -1.16 |
| PSMC6 | proteasome (prosome, macropain) 26S subunit, ATPase, 6 (PSMC6) | NM_002806 | 0.00725 | -1.45 | -1.25 | -2.70 |
| STT3B | STT3, subunit of the oligosaccharyltransferase complex, homolog B (S. cerevisiae) (STT3B) | NM_178862 | 0.00011 | -2.03 | -1.03 | -12.46 |
| LIMK1 | LIM domain kinase 1 (LIMK1) | NM_002314 | 0.02443 | -1.52 | -1.35 | -1.07 |
| C4BPB | complement component 4 binding protein, beta (C4BPB), transcript variant 1 | NM_000716 | 0.02105 | -1.23 | -1.13 | -7.87 |
| PTPN4 | protein tyrosine phosphatase, non-receptor type 4 (megakaryocyte) (PTPN4) | NM_002830 | 0.00140 | -1.17 | -1.09 | -1.79 |
| SEC11C | SEC11 homolog C (S. cerevisiae) (SEC11C) | NM_033280 | 0.04764 | -1.37 | -1.08 | -1.52 |
| DPH3 | DPH3, KTI11 homolog (S. cerevisiae) (DPH3), transcript variant 1 | NM_206831 | 0.00154 | -1.34 | -1.30 | -1.58 |
| WDSUB1 | WD repeat, sterile alpha motif and U-box domain containing 1 (WDSUB1) | NM_152528 | 0.00207 | -1.43 | -1.24 | -2.01 |
| RPA3 | replication protein A3, 14kDa (RPA3) | NM_002947 | 0.02529 | -1.13 | -1.16 | -1.83 |
| SPCS2 | signal peptidase complex subunit 2 homolog (S. cerevisiae) (SPCS2) | NM_014752 | 0.00894 | -1.17 | -1.05 | -1.49 |
| CTSL1 | cathepsin L1 (CTSL1), transcript variant 1 | NM_001912 | 0.00000 | -1.46 | -1.20 | -7.29 |
| LPAR1 | endothelial differentiation, lysophosphatidic acid G-protein-coupled receptor, 2 (EDG2), transcript variant 2 | NM_057159 | 0.00380 | -1.41 | -1.24 | -2.43 |
| ATG3 | ATG3 autophagy related 3 homolog (S. cerevisiae) (ATG3) | NM_022488 | 0.02635 | -1.10 | -1.00 | -1.93 |
| NEK2 | NIMA (never in mitosis gene a)-related kinase 2 (NEK2) | NM_002497 | 0.01089 | -1.84 | -1.39 | -1.83 |
| VCPIP1 | valosin containing protein (p97)/p47 complex interacting protein 1 (VCPIP1) | NM_025054 | 0.04356 | -1.32 | -1.16 | -1.10 |
| LMAN1 | lectin, mannose-binding, 1 (LMAN1) | NM_005570 | 0.00308 | -1.45 | -1.11 | -1.51 |
| CCL2 | chemokine (C-C motif) ligand 2 (CCL2) | NM_002982 | 0.00212 | -4.76 | -1.29 | -2.90 |
| UBE2A | ubiquitin-conjugating enzyme E2A (RAD6 homolog) (UBE2A), transcript variant 1 | NM_003336 | 0.04429 | -1.15 | -1.03 | -1.31 |
| DNAJC10 | DnaJ (Hsp40) homolog, subfamily C, member 10 (DNAJC10) | NM_018981 | 0.00456 | -1.30 | -1.02 | -1.71 |
| NEK6 | NIMA (never in mitosis gene a)-related kinase 6 (NEK6) | NM_014397 | 0.00377 | -1.16 | -1.57 | -1.41 |
| NGLY1 | N-glycanase 1 (NGLY1) | NM_018297 | 0.00694 | -1.20 | -1.11 | -1.40 |
| LDHA | lactate dehydrogenase A (LDHA) | NM_005566 | 0.00826 | -2.05 | -1.23 | -1.50 |
| ANXA1 | annexin A1 (ANXA1) | NM_000700 | 0.00388 | -2.09 | -1.22 | -1.83 |
| CDC2 | cell division cycle 2, G1 to S and G2 to M (CDC2), transcript variant 1 | NM_001786 | 0.01615 | -1.59 | -1.48 | -3.09 |
| UCHL3 | ubiquitin carboxyl-terminal esterase L3 (ubiquitin thiolesterase) (UCHL3) | NM_006002 | 0.00130 | -1.52 | -1.26 | -1.63 |
| UGCGL2 | UDP-glucose ceramide glucosyltransferase-like 2 (UGCGL2) | NM_020121 | 0.00152 | -1.21 | -1.15 | -4.36 |
| TALDO1 | transaldolase 1 (TALDO1) | NM_006755 | 0.00000 | -1.26 | -1.16 | -7.25 |
| MET | met proto-oncogene (hepatocyte growth factor receptor) (MET) | NM_000245 | 0.01290 | -1.13 | -1.41 | -1.36 |
| FBXO15 | F-box protein 15 (FBXO15) | NM_152676 | 0.00646 | -2.31 | -1.40 | -1.42 |
| GMFB | glia maturation factor, beta (GMFB) | NM_004124 | 0.01276 | -1.03 | -1.13 | -1.81 |
| UHRF1 | ubiquitin-like, containing PHD and RING finger domains, 1 (UHRF1), transcript variant 2 | NM_013282 | 0.04285 | -2.93 | -1.27 | -1.46 |
| GLRX2 | glutaredoxin 2 (GLRX2), transcript variant 1 | NM_016066 | 0.01288 | -1.59 | -1.21 | -1.63 |
| CTSA | cathepsin A (CTSA) | NM_000308 | 0.01883 | -1.44 | -1.50 | -1.08 |
| RABGGTB | Rab geranylgeranyltransferase, beta subunit (RABGGTB) | NM_004582 | 0.03320 | -1.47 | -1.03 | -1.91 |
| Sep15 | 15 kDa selenoprotein (SEP15), transcript variant 1 | NM_004261 | 0.01014 | -1.35 | -1.06 | -1.58 |
| MMP25 | matrix metallopeptidase 25 (MMP25) | NM_022468 | 0.00133 | -2.38 | -11.00 | -1.44 |
| PRCP | prolylcarboxypeptidase (angiotensinase C) (PRCP), transcript variant 2 | NM_199418 | 0.00507 | -1.26 | -1.73 | -1.49 |
| LATS1 | LATS, large tumor suppressor, homolog 1 (Drosophila) (LATS1) | NM_004690 | 0.00166 | -1.45 | -1.29 | -1.84 |
| RNF128 | ring finger protein 128 (RNF128), transcript variant 1 | NM_194463 | 0.00082 | -1.64 | -1.99 | -4.81 |
| PSMA1 | proteasome (prosome, macropain) subunit, alpha type, 1 (PSMA1), transcript variant 1 | NM_148976 | 0.01299 | -1.25 | -1.20 | -1.49 |
| UEVLD | UEV and lactate/malate dehyrogenase domains (UEVLD), transcript variant 2 | NM_018314 | 0.00171 | -1.40 | -1.06 | -1.36 |
| FKBP11 | FK506 binding protein 11, 19 kDa (FKBP11) | NM_016594 | 0.01455 | -1.81 | -1.23 | -1.49 |
| SENP7 | SUMO1/sentrin specific peptidase 7 (SENP7), transcript variant 1 | NM_020654 | 0.01247 | -1.46 | -1.30 | -3.08 |
| SEC11A | SEC11 homolog A (S. cerevisiae) (SEC11A) | NM_014300 | 0.01376 | -1.19 | -1.09 | -1.79 |
| PPP3CA | protein phosphatase 3 (formerly 2B), catalytic subunit, alpha isoform (PPP3CA) | NM_000944 | 0.02224 | -1.21 | -1.05 | -1.64 |
| CYCS | cytochrome c, somatic (CYCS), nuclear gene encoding mitochondrial protein | NM_018947 | 0.04013 | -1.77 | -1.03 | -1.56 |
| UBE2D1 | ubiquitin-conjugating enzyme E2D 1 (UBC4/5 homolog, yeast) (UBE2D1) | NM_003338 | 0.03951 | -1.70 | -1.20 | -1.23 |
| PLOD2 | procollagen-lysine, 2-oxoglutarate 5-dioxygenase 2 (PLOD2), transcript variant 1 | NM_182943 | 0.01431 | -1.91 | -1.46 | -1.23 |
| NSMCE2 | non-SMC element 2, MMS21 homolog (S. cerevisiae) (NSMCE2) | NM_173685 | 0.00022 | -1.10 | -1.06 | -3.23 |
| RNF216 | TRIAD3 protein (TRIAD3), transcript variant 1 | NM_207111 | 0.00165 | -3.73 | -1.50 | -6.79 |
| UCHL5 | ubiquitin carboxyl-terminal hydrolase L5 (UCHL5) | NM_015984 | 0.00059 | -1.43 | -1.31 | -1.54 |
| ADAM17 | ADAM metallopeptidase domain 17 (tumor necrosis factor, alpha, converting enzyme) (ADAM17) | NM_003183 | 0.02485 | -1.06 | -1.32 | -1.34 |
| NARG1 | NMDA receptor regulated 1 (NARG1) | NM_057175 | 0.00206 | -1.44 | -1.05 | -1.39 |
| ATG4C | ATG4 autophagy related 4 homolog C (S. cerevisiae) (ATG4C), transcript variant 7 | NM_032852 | 0.00000 | -1.01 | -1.57 | -14.42 |
| PPM1D | protein phosphatase 1D magnesium-dependent, delta isoform (PPM1D) | NM_003620 | 0.02902 | 1.08 | -1.12 | -1.64 |
| CSNK1A1L | casein kinase 1, alpha 1-like (CSNK1A1L) | NM_145203 | 0.00808 | 1.03 | -1.19 | -1.79 |
| LVRN | laeverin (FLJ90650) | NM_173800 | 0.03429 | 1.32 | -1.15 | -3.16 |
| RFWD2 | ring finger and WD repeat domain 2 (RFWD2), transcript variant 1 | NM_022457 | 0.04857 | 1.02 | -1.01 | -1.30 |
| MMP1 | matrix metallopeptidase 1 (interstitial collagenase) (MMP1) | NM_002421 | 0.01962 | 1.04 | -2.64 | -1.29 |
| EIF4A3 | eukaryotic translation initiation factor 4A, isoform 3 (EIF4A3) | NM_014740 | 0.02635 | -1.29 | 1.25 | -4.47 |
| PFDN4 | prefoldin subunit 4 (PFDN4) | NM_002623 | 0.03253 | -1.18 | 1.07 | -2.33 |
| RP2 | retinitis pigmentosa 2 (X-linked recessive) (RP2) | NM_006915 | 0.00011 | -2.05 | 1.11 | -9.21 |
| NAE1 | amyloid beta precursor protein binding protein 1 (APPBP1), transcript variant 1 | NM_003905 | 0.00505 | -1.13 | 1.08 | -1.69 |
| PSMD14 | proteasome (prosome, macropain) 26S subunit, non-ATPase, 14 (PSMD14) | NM_005805 | 0.03188 | -1.31 | 1.04 | -1.66 |
| XIAP | baculoviral IAP repeat-containing 4 (BIRC4) | NM_001167 | 0.00004 | -1.88 | 1.28 | -9.59 |
| PARP4 | poly (ADP-ribose) polymerase family, member 4 (PARP4) | NM_006437 | 0.01348 | -1.79 | 1.14 | -6.57 |
| WNK2 | WNK lysine deficient protein kinase 2 (WNK2) | NM_006648 | 0.00452 | -1.99 | 1.12 | -4.30 |
| ATG10 | ATG10 autophagy related 10 homolog (S. cerevisiae) (ATG10) | NM_031482 | 0.04906 | -1.44 | 1.10 | -1.61 |
| PPP1R2 | protein phosphatase 1, regulatory (inhibitor) subunit 2 (PPP1R2) | NM_006241 | 0.00611 | -1.40 | 1.01 | -1.85 |
| PPP1CB | protein phosphatase 1, catalytic subunit, beta isoform (PPP1CB), transcript variant 1 | NM_002709 | 0.01105 | -1.13 | 1.01 | -1.82 |
| BAG2 | BCL2-associated athanogene 2 (BAG2) | NM_004282 | 0.03387 | -1.39 | 1.01 | -2.40 |
| IMMP1L | IMP1 inner mitochondrial membrane peptidase-like (S. cerevisiae) (IMMP1L) | NM_144981 | 0.00053 | -1.07 | 1.05 | -21.22 |
| SLC35A1 | solute carrier family 35 (CMP-sialic acid transporter), member A1 (SLC35A1) | NM_006416 | 0.00025 | -1.90 | 1.03 | -4.17 |
| UBTD2 | ubiquitin domain containing 2 (UBTD2) | NM_152277 | 0.04344 | -1.20 | 1.00 | -1.50 |
| NGF | nerve growth factor, beta polypeptide (NGFB) | NM_002506 | 0.04477 | -1.24 | 1.09 | -5.37 |
| RABGEF1 | RAB guanine nucleotide exchange factor (GEF) 1 (RABGEF1) | NM_014504 | 0.01959 | -1.31 | 1.00 | -1.58 |
| PTCH1 | patched homolog 1 (Drosophila) (PTCH1) | NM_000264 | 0.02086 | 1.87 | 1.22 | -1.06 |
| TESSP2 | testis serine protease 2 (TESSP2) | NM_182702 | 0.00391 | 2.03 | 1.74 | -1.05 |
| DYRK4 | dual-specificity tyrosine-(Y)-phosphorylation regulated kinase 4 (DYRK4) | NM_003845 | 0.01346 | 2.33 | 1.26 | -1.07 |
| DNAJC4 | DnaJ (Hsp40) homolog, subfamily C, member 4 (DNAJC4) | NM_005528 | 0.02045 | 1.75 | 1.51 | -1.05 |
| NTRK3 | neurotrophic tyrosine kinase, receptor, type 3 (NTRK3), transcript variant 1 | NM_001012338 | 0.00389 | 1.59 | 3.47 | -1.04 |
| DNAJB6 | DnaJ (Hsp40) homolog, subfamily B, member 6 (DNAJB6), transcript variant 1 | NM_058246 | 0.00525 | 1.39 | 1.50 | -1.02 |
| CCL11 | chemokine (C-C motif) ligand 11 (CCL11) | NM_002986 | 0.00899 | -4.05 | -3.18 | 1.00 |
| C2 | complement component 2 (C2) | NM_000063 | 0.00543 | -2.91 | -1.47 | 1.23 |
| PPM1F | protein phosphatase 1F (PP2C domain containing) (PPM1F) | NM_014634 | 0.01436 | -1.03 | -1.01 | 1.58 |
| TYK2 | tyrosine kinase 2 (TYK2) | NM_003331 | 0.01657 | 1.01 | -1.00 | 2.02 |
| PAM | peptidylglycine alpha-amidating monooxygenase (PAM), transcript variant 1 | NM_000919 | 0.01594 | 1.60 | -1.05 | 1.31 |
| TGM2 | transglutaminase 2 (C polypeptide, protein-glutamine-gamma-glutamyltransferase) (TGM2), transcript variant 2 | NM_198951 | 0.02426 | 1.84 | -1.31 | 1.77 |
| PTPN18 | protein tyrosine phosphatase, non-receptor type 18 (brain-derived) (PTPN18) | NM_014369 | 0.00761 | 1.46 | -1.01 | 2.12 |
| ADAM8 | ADAM metallopeptidase domain 8 (ADAM8) | NM_001109 | 0.02707 | 1.21 | -1.07 | 2.50 |
| MADD | MAP-kinase activating death domain (MADD), transcript variant 4 | NM_003682 | 0.03654 | -1.02 | 1.20 | 1.69 |
| E4F1 | E4F transcription factor 1 (E4F1) | NM_004424 | 0.03692 | -1.03 | 1.16 | 1.43 |
| SMURF1 | SMAD specific E3 ubiquitin protein ligase 1 (SMURF1), transcript variant 1 | NM_020429 | 0.01650 | -1.07 | 1.07 | 2.14 |
| STRADA | protein kinase LYK5 (LYK5), transcript variant 3 | NM_153335 | 0.04555 | -1.00 | 1.07 | 1.49 |
| LRPAP1 | low density lipoprotein receptor-related protein associated protein 1 (LRPAP1) | NM_002337 | 0.04717 | -1.04 | 1.16 | 1.31 |
| PDK2 | pyruvate dehydrogenase kinase, isozyme 2 (PDK2) | NM_002611 | 0.01009 | -1.01 | 1.22 | 1.56 |
| UBE2D4 | ubiquitin-conjugating enzyme E2D 4 (putative) (UBE2D4) | NM_015983 | 0.02630 | -1.03 | 1.16 | 1.21 |
| USP11 | ubiquitin specific peptidase 11 (USP11) | NM_004651 | 0.04313 | 1.18 | 1.15 | 1.17 |
| MBD3 | methyl-CpG binding domain protein 3 (MBD3) | NM_003926 | 0.03267 | 1.17 | 1.14 | 1.51 |
| GAK | cyclin G associated kinase (GAK) | NM_005255 | 0.00453 | 1.16 | 1.11 | 1.54 |
| SMG7 | Smg-7 homolog, nonsense mediated mRNA decay factor (C. elegans) (SMG7), transcript variant 1 | NM_173156 | 0.01670 | 1.19 | 1.19 | 1.31 |
| MAST2 | microtubule associated serine/threonine kinase 2 (MAST2) | NM_015112 | 0.00394 | 1.33 | 1.66 | 2.00 |
| DBNL | drebrin-like (DBNL), transcript variant 1 | NM_014063 | 0.00528 | 1.11 | 1.18 | 1.71 |
| ADAM32 | ADAM metallopeptidase domain 32 (ADAM32) | NM_145004 | 0.00041 | 2.11 | 1.92 | 1.58 |
| FGFR1 | fibroblast growth factor receptor 1 (fms-related tyrosine kinase 2, Pfeiffer syndrome) (FGFR1), transcript variant 5 | NM_023107 | 0.00234 | 1.32 | 1.41 | 1.54 |
| NTHL1 | nth endonuclease III-like 1 (E. coli) (NTHL1) | NM_002528 | 0.00579 | 1.45 | 1.41 | 1.28 |
| DUSP18 | dual specificity phosphatase 18 (DUSP18) | NM_152511 | 0.02270 | 1.06 | 1.08 | 2.13 |
| LRRC41 | leucine rich repeat containing 41 (LRRC41) | NM_006369 | 0.03922 | 1.07 | 1.10 | 1.28 |
| ADAMTS13 | ADAM metallopeptidase with thrombospondin type 1 motif, 13 (ADAMTS13), transcript variant 1 | NM_139025 | 0.00496 | 1.35 | 1.46 | 2.36 |
| USP34 | ubiquitin specific peptidase 34 (USP34) | NM_014709 | 0.02400 | 1.09 | 1.16 | 1.38 |
| TRIB2 | tribbles homolog 2 (Drosophila) (TRIB2) | NM_021643 | 0.03882 | 1.12 | 1.23 | 1.38 |
| NMT1 | N-myristoyltransferase 1 (NMT1) | NM_021079 | 0.00770 | 1.24 | 1.20 | 1.39 |
| ZFP36L1 | zinc finger protein 36, C3H type-like 1 (ZFP36L1) | NM_004926 | 0.00362 | 1.28 | 1.01 | 1.90 |
| PRPF19 | PRP19/PSO4 pre-mRNA processing factor 19 homolog (S. cerevisiae) (PRPF19) | NM_014502 | 0.02173 | 1.37 | 1.15 | 1.11 |
| AGA | aspartylglucosaminidase (AGA) | NM_000027 | 0.04974 | 1.40 | 1.12 | 1.14 |
| LTK | leukocyte tyrosine kinase (LTK), transcript variant 1 | NM_002344 | 0.00579 | 1.29 | 1.16 | 1.55 |
| UBOX5 | U-box domain containing 5 (UBOX5), transcript variant 1 | NM_014948 | 0.00439 | 1.50 | 1.54 | 1.27 |
| ILKAP | integrin-linked kinase-associated serine/threonine phosphatase 2C (ILKAP) | NM_030768 | 0.00039 | 1.32 | 1.26 | 1.20 |
| S1PR2 | endothelial differentiation, sphingolipid G-protein-coupled receptor, 5 (EDG5) | NM_004230 | 0.02093 | 1.55 | 1.39 | 1.14 |
| TMPRSS6 | transmembrane protease, serine 6 (TMPRSS6) | NM_153609 | 0.04003 | 1.17 | 1.29 | 1.44 |
| TLK2 | tousled-like kinase 2 (TLK2) | NM_006852 | 0.01577 | 1.29 | 1.12 | 1.19 |
| TAOK2 | TAO kinase 2 (TAOK2), transcript variant 2 | NM_016151 | 0.04793 | 1.13 | 1.16 | 1.16 |
| WSB1 | WD repeat and SOCS box-containing 1 (WSB1), transcript variant 1 | NM_015626 | 0.02409 | 1.19 | 1.33 | 1.43 |
| DNAJB12 | DnaJ (Hsp40) homolog, subfamily B, member 12 (DNAJB12), transcript variant 2 | NM_017626 | 0.02700 | 1.24 | 1.09 | 1.33 |
| CCT6A | chaperonin containing TCP1, subunit 6A (zeta 1) (CCT6A), transcript variant 1 | NM_001762 | 0.04972 | 1.07 | 1.18 | 1.44 |
| ZC3HC1 | zinc finger, C3HC-type containing 1 (ZC3HC1) | NM_016478 | 0.01575 | 1.06 | 1.16 | 1.32 |
| NRD1 | nardilysin (N-arginine dibasic convertase) (NRD1) | NM_002525 | 0.01293 | 1.08 | 1.14 | 1.16 |
| RBM14 | RNA binding motif protein 14 (RBM14) | NM_006328 | 0.01424 | 1.01 | 1.23 | 1.69 |
| GRK5 | G protein-coupled receptor kinase 5 (GRK5) | NM_005308 | 0.03852 | 1.24 | 1.14 | 1.40 |
| DET1 | de-etiolated homolog 1 (Arabidopsis) (DET1) | NM_017996 | 0.02941 | 1.00 | 1.31 | 1.03 |
| RBM8A | RNA binding motif protein 8A (RBM8A) | NM_005105 | 0.01583 | 1.38 | 1.22 | 1.24 |
| TYSND1 | trypsin domain containing 1 (TYSND1), transcript variant 1 | NM_173555 | 0.03955 | 1.46 | 1.24 | 1.36 |
| GNA11 | guanine nucleotide binding protein (G protein), alpha 11 (Gq class) (GNA11) | NM_002067 | 0.00605 | 1.14 | 1.08 | 2.25 |
| SPSB2 | splA/ryanodine receptor domain and SOCS box containing 2 (SPSB2) | NM_032641 | 0.04608 | 1.02 | 1.23 | 1.22 |
| TOR1A | torsin family 1, member A (torsin A) (TOR1A) | NM_000113 | 0.02638 | 1.13 | 1.02 | 1.33 |
| TMUB2 | transmembrane and ubiquitin-like domain containing 2 (TMUB2), transcript variant 2 | NM_177441 | 0.03699 | 1.08 | 1.00 | 1.34 |
| NSD1 | nuclear receptor binding SET domain protein 1 (NSD1), transcript variant 2 | NM_022455 | 0.01055 | 1.08 | 1.19 | 1.60 |
| ASB6 | ankyrin repeat and SOCS box-containing 6 (ASB6), transcript variant 1 | NM_017873 | 0.00658 | 1.43 | 1.16 | 1.29 |
| STK11 | serine/threonine kinase 11 (STK11) | NM_000455 | 0.00071 | 1.46 | 1.32 | 1.39 |
| TMUB2 | transmembrane and ubiquitin-like domain containing 2 (TMUB2), transcript variant 2 | NM_177441 | 0.02541 | 1.08 | 1.01 | 1.33 |
| BRAP | BRCA1 associated protein (BRAP) | NM_006768 | 0.01382 | 1.26 | 1.09 | 1.17 |
| EHMT1 | euchromatic histone-lysine N-methyltransferase 1 (EHMT1) | NM_024757 | 0.02747 | 1.25 | 1.17 | 1.23 |
| TAOK2 | TAO kinase 2 (TAOK2), transcript variant 1 | NM_004783 | 0.02308 | 1.15 | 1.31 | 1.39 |
| DULLARD | dullard homolog (Xenopus laevis) (DULLARD) | NM_015343 | 0.04747 | 1.41 | 1.10 | 1.16 |
| TYRO3 | TYRO3 protein tyrosine kinase (TYRO3) | NM_006293 | 0.01027 | 1.27 | 1.51 | 1.28 |
| TMPRSS5 | transmembrane protease, serine 5 (spinesin) (TMPRSS5) | NM_030770 | 0.01230 | 1.16 | 1.60 | 2.05 |
| LONP1 | lon peptidase 1, mitochondrial (LONP1), nuclear gene encoding mitochondrial protein | NM_004793 | 0.01912 | 1.18 | 1.09 | 1.28 |
| PPM1G | protein phosphatase 1G (formerly 2C), magnesium-dependent, gamma isoform (PPM1G), transcript variant 1 | NM_177983 | 0.04791 | 1.17 | 1.17 | 1.18 |
| KAT2A | GCN5 general control of amino-acid synthesis 5-like 2 (yeast) (GCN5L2) | NM_021078 | 0.01841 | 1.25 | 1.34 | 2.55 |
| CRY2 | cryptochrome 2 (photolyase-like) (CRY2) | NM_021117 | 0.02213 | 1.45 | 1.41 | 1.34 |
| MAP2K5 | mitogen-activated protein kinase kinase 5 (MAP2K5), transcript variant B | NM_002757 | 0.04484 | 1.34 | 1.07 | 1.24 |
| CHFR | checkpoint with forkhead and ring finger domains (CHFR) | NM_018223 | 0.03282 | 1.14 | 1.09 | 1.71 |
| FLJ25006 | hypothetical protein FLJ25006 (FLJ25006) | NM_144610 | 0.04139 | 1.22 | 1.39 | 1.65 |
| MYST3 | MYST histone acetyltransferase (monocytic leukemia) 3 (MYST3) | NM_006766 | 0.01341 | 3.30 | 2.83 | 1.40 |
| AGBL5 | hypothetical protein FLJ21839 (FLJ21839), transcript variant 1 | NM_021831 | 0.00135 | 1.59 | 1.65 | 1.33 |
| MYLIP | myosin regulatory light chain interacting protein (MYLIP) | NM_013262 | 0.02335 | 1.72 | 1.72 | 1.19 |
| CTSF | cathepsin F (CTSF) | NM_003793 | 0.00537 | 1.37 | 1.16 | 1.21 |
| GLYCTK | glycerate kinase (GLYCTK) | NM_145262 | 0.02879 | 1.12 | 1.01 | 2.04 |
| PICK1 | protein interacting with PRKCA 1 (PICK1), transcript variant 1 | NM_012407 | 0.03367 | 1.14 | 1.05 | 1.75 |
| SUV39H1 | suppressor of variegation 3-9 homolog 1 (Drosophila) (SUV39H1) | NM_003173 | 0.00859 | 1.41 | 1.08 | 1.08 |
| QSOX1 | quiescin Q6 (QSCN6), transcript variant 1 | NM_002826 | 0.00007 | 1.67 | 1.44 | 1.47 |
| APOA5 | apolipoprotein A-V (APOA5) | NM_052968 | 0.04517 | 1.74 | 1.70 | 1.69 |
| FBXL8 | F-box and leucine-rich repeat protein 8 (FBXL8) | NM_018378 | 0.00697 | 2.13 | 1.12 | 1.25 |
| SIRT6 | sirtuin (silent mating type information regulation 2 homolog) 6 (S. cerevisiae) (SIRT6) | NM_016539 | 0.00086 | 2.41 | 1.68 | 3.08 |
| DNAJC7 | DnaJ (Hsp40) homolog, subfamily C, member 7 (DNAJC7) | NM_003315 | 0.03109 | 1.16 | 1.17 | 1.18 |
| TEX14 | testis expressed 14 (TEX14), transcript variant 2 | NM_031272 | 0.02004 | 1.55 | 1.46 | 1.33 |
| STRADA | protein kinase LYK5 (LYK5), transcript variant 3 | NM_153335 | 0.04120 | 1.06 | 1.06 | 1.48 |
| ANAPC4 | anaphase promoting complex subunit 4 (ANAPC4) | NM_013367 | 0.04692 | 1.36 | 1.19 | 1.21 |
| TNK2 | tyrosine kinase, non-receptor, 2 (TNK2), transcript variant 2 | NM_001010938 | 0.00244 | 1.30 | 1.20 | 2.83 |
| IFT172 | intraflagellar transport 172 homolog (Chlamydomonas) (IFT172) | NM_015662 | 0.04998 | 1.49 | 1.14 | 1.26 |
| TRRAP | transformation/transcription domain-associated protein (TRRAP) | NM_003496 | 0.00126 | 1.26 | 1.09 | 1.77 |
| DNASE1L2 | deoxyribonuclease I-like 2 (DNASE1L2) | NM_001374 | 0.00222 | 1.38 | 1.61 | 2.09 |
| SMG6 | Smg-6 homolog, nonsense mediated mRNA decay factor (C. elegans) (SMG6) | NM_017575 | 0.03440 | 1.27 | 1.02 | 1.35 |
| RPIA | ribose 5-phosphate isomerase A (ribose 5-phosphate epimerase) (RPIA) | NM_144563 | 0.04179 | 1.33 | 1.03 | 1.36 |
| NPEPL1 | aminopeptidase-like 1 (NPEPL1) | NM_024663 | 0.03342 | 1.04 | 1.10 | 1.73 |
| NFYC | nuclear transcription factor Y, gamma (NFYC) | NM_014223 | 0.04020 | 1.07 | 1.28 | 1.25 |
| ADPRH | ADP-ribosylarginine hydrolase (ADPRH) | NM_001125 | 0.04907 | 1.11 | 1.37 | 1.47 |
| CSNK1E | casein kinase 1, epsilon (CSNK1E), transcript variant 1 | NM_152221 | 0.00896 | 1.13 | 1.04 | 1.74 |
| TRIM11 | tripartite motif-containing 11 (TRIM11) | NM_145214 | 0.03841 | 1.25 | 1.09 | 1.21 |
| PASK | PAS domain containing serine/threonine kinase (PASK) | NM_015148 | 0.03426 | 5.98 | 3.00 | 19.17 |
| DSTYK | receptor interacting protein kinase 5 (RIPK5), transcript variant 1 | NM_015375 | 0.02397 | 1.39 | 1.08 | 1.24 |
| DAXX | death-associated protein 6 (DAXX) | NM_001350 | 0.01882 | 1.08 | 1.02 | 1.42 |
| PPIE | peptidylprolyl isomerase E (cyclophilin E) (PPIE), transcript variant 2 | NM_203456 | 0.00446 | 1.48 | 1.20 | 2.08 |
| MAP2K7 | mitogen-activated protein kinase kinase 7 (MAP2K7) | NM_145185 | 0.01397 | 1.34 | 1.10 | 1.38 |
| TTLL4 | tubulin tyrosine ligase-like family, member 4 (TTLL4) | NM_014640 | 0.00379 | 1.14 | 1.18 | 1.55 |
| PRMT1 | protein arginine methyltransferase 1 (PRMT1), transcript variant 2 | NM_198319 | 0.01425 | 1.20 | 1.34 | 1.04 |
| CREBBP | CREB binding protein (Rubinstein-Taybi syndrome) (CREBBP), transcript variant 1 | NM_004380 | 0.01338 | 1.24 | 1.10 | 1.19 |
| HDAC1 | histone deacetylase 1 (HDAC1) | NM_004964 | 0.02670 | 1.20 | 1.06 | 1.21 |
| OGDHL | oxoglutarate dehydrogenase-like (OGDHL) | NM_018245 | 0.04324 | 2.02 | 1.35 | 1.98 |
| DPEP2 | dipeptidase 2 (DPEP2) | NM_022355 | 0.00227 | 1.83 | 2.35 | 1.13 |
| SPG7 | spastic paraplegia 7, paraplegin (pure and complicated autosomal recessive) (SPG7), nuclear gene encoding mitochondrial protein, transcript variant 1 | NM_003119 | 0.00634 | 1.16 | 1.34 | 1.59 |
| ARIH2 | ariadne homolog 2 (Drosophila) (ARIH2) | NM_006321 | 0.03014 | 1.21 | 1.20 | 1.08 |
| PWP2 | PWP2 periodic tryptophan protein homolog (yeast) (PWP2) | NM_005049 | 0.02373 | 1.14 | 1.16 | 1.58 |
| VRK3 | vaccinia related kinase 3 (VRK3), transcript variant 1 | NM_016440 | 0.00635 | 1.13 | 1.11 | 1.20 |
| PAN2 | ubiquitin specific peptidase 52 (USP52) | NM_014871 | 0.00699 | 1.33 | 1.37 | 1.78 |
| ADAM33 | ADAM metallopeptidase domain 33 (ADAM33), transcript variant 1 | NM_025220 | 0.02606 | 1.24 | 1.43 | 2.21 |
| STK4 | serine/threonine kinase 4 (STK4) | NM_006282 | 0.00147 | 1.49 | 1.47 | 1.30 |
| RNF20 | ring finger protein 20 (RNF20) | NM_019592 | 0.02094 | 1.21 | 1.21 | 1.30 |
| HGFAC | HGF activator (HGFAC) | NM_001528 | 0.00009 | 1.49 | 1.17 | 2.68 |
| CDK3 | cyclin-dependent kinase 3 (CDK3) | NM_001258 | 0.01819 | 1.15 | 1.91 | 2.07 |
| CNDP2 | CNDP dipeptidase 2 (metallopeptidase M20 family) (CNDP2) | NM_018235 | 0.04946 | 1.15 | 1.09 | 1.25 |
| PCSK4 | proprotein convertase subtilisin/kexin type 4 (PCSK4) | NM_017573 | 0.02027 | 1.42 | 1.79 | 2.07 |
| **Cellular Nitrogen Metabolic Process** | |  |  |  |  |  |
| HIBCH | 3-hydroxyisobutyryl-Coenzyme A hydrolase (HIBCH), transcript variant 1 | NM_014362 | 0.00004 | -1.91 | -1.09 | -5.96 |
| HNF4A | hepatocyte nuclear factor 4, alpha (HNF4A), transcript variant 3 | NM_178850 | 0.04053 | 1.84 | -1.03 | 2.01 |
| ALDH6A1 | aldehyde dehydrogenase 6 family, member A1 (ALDH6A1), nuclear gene encoding mitochondrial protein | NM_005589 | 0.02381 | 1.31 | 1.18 | 1.71 |
| AMT | aminomethyltransferase (AMT) | NM_000481 | 0.02509 | 1.06 | 1.58 | 1.15 |
| IDUA | iduronidase, alpha-L- (IDUA) | NM_000203 | 0.00096 | 1.29 | 1.19 | 1.68 |
| ALLC | allantoicase (ALLC), transcript variant 1 | NM_018436 | 0.00671 | 1.86 | 4.06 | 2.77 |
| TAT | tyrosine aminotransferase (TAT), nuclear gene encoding mitochondrial protein | NM_000353 | 0.00076 | 2.21 | 2.21 | 1.67 |
| TST | thiosulfate sulfurtransferase (rhodanese) (TST), nuclear gene encoding mitochondrial protein | NM_003312 | 0.00697 | 1.31 | 1.13 | 1.17 |
| CKB | creatine kinase, brain (CKB) | NM_001823 | 0.04835 | 1.54 | 1.80 | 2.00 |
| SHMT2 | serine hydroxymethyltransferase 2 (mitochondrial) (SHMT2) | NM_005412 | 0.03161 | 1.01 | 1.02 | 2.67 |
| **Cellular Carbohydrate Metabolic Process** | |  |  |  |  |  |
| EXTL2 | exostoses (multiple)-like 2 (EXTL2), transcript variant 1 | NM_001439 | 0.03129 | -1.36 | -1.42 | -1.24 |
| GNPDA2 | glucosamine-6-phosphate deaminase 2 (GNPDA2) | NM_138335 | 0.01769 | -1.45 | -1.04 | -1.31 |
| GALE | UDP-galactose-4-epimerase (GALE), transcript variant 1 | NM_000403 | 0.00403 | 1.59 | -1.38 | -18.30 |
| MAN2C1 | mannosidase, alpha, class 2C, member 1 (MAN2C1) | NM_006715 | 0.01551 | 1.23 | 1.19 | 1.81 |
| NISCH | nischarin (NISCH) | NM_007184 | 0.00269 | 1.36 | 1.14 | 2.33 |
| FN3K | fructosamine 3 kinase (FN3K) | NM_022158 | 0.03850 | 1.51 | 1.36 | 1.09 |
| **Cellular Catabolic Process** | |  |  |  |  |  |
| FAM176A | transmembrane protein 166 (TMEM166) | NM_032181 | 0.04038 | -1.89 | -1.48 | -1.27 |
| DERA | 2-deoxyribose-5-phosphate aldolase homolog (C. elegans) (DERA) | NM_015954 | 0.01738 | -1.50 | -1.02 | -1.52 |
| SDHD | succinate dehydrogenase complex, subunit D, integral membrane protein (SDHD), nuclear gene encoding mitochondrial protein | NM_003002 | 0.04416 | -1.38 | 1.01 | -1.15 |
| ACO2 | aconitase 2, mitochondrial (ACO2), nuclear gene encoding mitochondrial protein | NM_001098 | 0.04669 | -1.13 | 1.24 | 1.63 |
| ATG16L1 | ATG16 autophagy related 16-like 1 (S. cerevisiae) (ATG16L1), transcript variant 1 | NM_030803 | 0.02661 | 1.11 | 1.25 | 1.04 |
| RAB24 | RAB24, member RAS oncogene family (RAB24), transcript variant 2 | NM_130781 | 0.02101 | 1.08 | 1.17 | 1.61 |
| ATG9A | ATG9 autophagy related 9 homolog A (S. cerevisiae) (ATG9A), transcript variant 2 | NM_024085 | 0.03163 | 1.43 | 1.12 | 1.45 |
| ATG16L2 | ATG16 autophagy related 16-like 2 (S. cerevisiae) (ATG16L2) | NM_033388 | 0.00933 | 1.00 | 1.32 | 1.44 |
| TMEM150 | transmembrane protein 150 (TMEM150), transcript variant 2 | NM_153342 | 0.0125075 | 1.5075 | 1.227 | 1.0619 |
| **nucleobase, nucleoside, nucleotide and nucleic acid metabolic process** | |  |  |  |  |  |
| PHF10 | PHD finger protein 10 (PHF10), transcript variant 1 | NM_018288 | 0.03495 | -1.15 | -1.20 | -1.17 |
| LSM3 | LSM3 homolog, U6 small nuclear RNA associated (S. cerevisiae) (LSM3) | NM_014463 | 0.02155 | -1.24 | -1.27 | -1.82 |
| RRM2B | ribonucleotide reductase M2 B (TP53 inducible) (RRM2B) | NM_015713 | 0.01214 | -1.66 | -1.08 | -1.21 |
| TSEN15 | chromosome 1 open reading frame 19 (C1orf19) | NM_052965 | 0.00784 | -1.63 | -1.11 | -1.48 |
| ZNF177 | zinc finger protein 177 (ZNF177) | NM_003451 | 0.02917 | -2.10 | -1.73 | -1.12 |
| ZNF552 | zinc finger protein 552 (ZNF552) | NM_024762 | 0.00595 | -1.87 | -1.20 | -1.44 |
| GMEB1 | glucocorticoid modulatory element binding protein 1 (GMEB1), transcript variant 1 | NM_006582 | 0.00903 | -1.39 | -2.25 | -1.48 |
| ATP5F1 | ATP synthase, H+ transporting, mitochondrial F0 complex, subunit B1 (ATP5F1), nuclear gene encoding mitochondrial protein | NM_001688 | 0.00350 | -1.85 | -1.15 | -30.00 |
| ESF1 | ESF1, nucleolar pre-rRNA processing protein, homolog (S. cerevisiae) (ESF1) | NM_016649 | 0.00000 | -1.62 | -1.01 | -9.75 |
| PHF19 | PHD finger protein 19 (PHF19), transcript variant 1 | NM_015651 | 0.00015 | -1.08 | -1.14 | -4.59 |
| RPP38 | ribonuclease P/MRP 38kDa subunit (RPP38), transcript variant 1 | NM_183005 | 0.02130 | -1.06 | -1.07 | -2.53 |
| C1orf124 | chromosome 1 open reading frame 124 (C1orf124), transcript variant 2 | NM_001010984 | 0.03544 | -1.24 | -1.08 | -1.27 |
| ANKRD17 | ankyrin repeat domain 17 (ANKRD17), transcript variant 1 | NM_032217 | 0.01787 | -1.17 | -1.05 | -1.81 |
| MCTS1 | malignant T cell amplified sequence 1 (MCTS1) | NM_014060 | 0.04158 | -1.19 | -1.10 | -1.28 |
| C1orf124 | chromosome 1 open reading frame 124 (C1orf124), transcript variant 2 | NM_001010984 | 0.03355 | -1.26 | -1.16 | -1.17 |
| CSTF2 | cleavage stimulation factor, 3' pre-RNA, subunit 2, 64kDa (CSTF2) | NM_001325 | 0.01279 | -1.62 | -1.10 | -1.34 |
| ZNF684 | zinc finger protein 684 (ZNF684) | NM_152373 | 0.01011 | -2.36 | -1.42 | -1.47 |
| PABPC3 | poly(A) binding protein, cytoplasmic 3 (PABPC3) | NM_030979 | 0.00100 | -1.53 | -1.13 | -3.22 |
| DR1 | down-regulator of transcription 1, TBP-binding (negative cofactor 2) (DR1) | NM_001938 | 0.00472 | -1.76 | -1.01 | -2.11 |
| RPP38 | ribonuclease P/MRP 38kDa subunit (RPP38), transcript variant 1 | NM_183005 | 0.02239 | -1.25 | -1.07 | -2.75 |
| WDSOF1 | WD repeats and SOF1 domain containing (WDSOF1) | NM_015420 | 0.00596 | -1.20 | -1.07 | -1.54 |
| PHF5A | PHD finger protein 5A (PHF5A) | NM_032758 | 0.00845 | -1.08 | -1.01 | -2.16 |
| MED10 | mediator of RNA polymerase II transcription, subunit 10 homolog (NUT2, S. cerevisiae) (MED10) | NM_032286 | 0.00015 | -1.65 | -1.43 | -3.25 |
| GEMIN6 | gem (nuclear organelle) associated protein 6 (GEMIN6) | NM_024775 | 0.04631 | -1.18 | -1.18 | -1.27 |
| ATP6V1D | ATPase, H+ transporting, lysosomal 34kDa, V1 subunit D (ATP6V1D) | NM_015994 | 0.02525 | -1.15 | -1.00 | -1.62 |
| APOBEC3G | apolipoprotein B mRNA editing enzyme, catalytic polypeptide-like 3G (APOBEC3G) | NM_021822 | 0.04070 | -4.86 | -1.67 | -1.11 |
| SUPT3H | suppressor of Ty 3 homolog (S. cerevisiae) (SUPT3H), transcript variant 1 | NM_003599 | 0.00014 | -1.41 | -1.36 | -4.67 |
| SNRPC | small nuclear ribonucleoprotein polypeptide C (SNRPC) | NM_003093 | 0.03895 | -1.12 | -1.03 | -1.17 |
| PABPC1 | poly(A) binding protein, cytoplasmic 1 (PABPC1) | NM_002568 | 0.04347 | -1.27 | -1.23 | -1.75 |
| ENY2 | enhancer of yellow 2 homolog (Drosophila) (ENY2) | NM_020189 | 0.00038 | -1.39 | -1.18 | -4.26 |
| ADAD2 | testis nuclear RNA-binding protein-like (LOC161931) | NM_139174 | 0.04732 | -1.65 | -1.33 | -1.52 |
| BCCIP | BRCA2 and CDKN1A interacting protein (BCCIP), transcript variant B | NM_078468 | 0.00210 | -1.35 | -1.32 | -1.36 |
| SBDS | Shwachman-Bodian-Diamond syndrome (SBDS) | NM_016038 | 0.03975 | -1.33 | -1.04 | -1.35 |
| ADK | adenosine kinase (ADK), transcript variant ADK-short | NM_001123 | 0.00016 | -5.64 | -1.37 | -9.39 |
| TCEAL7 | transcription elongation factor A (SII)-like 7 (TCEAL7) | NM_152278 | 0.00028 | -1.38 | -1.31 | -6.30 |
| ZBTB11 | zinc finger and BTB domain containing 11 (ZBTB11) | NM_014415 | 0.00674 | -1.47 | -1.04 | -2.03 |
| BRWD1 | bromodomain and WD repeat domain containing 1 (BRWD1), transcript variant 3 | NM_001007246 | 0.04886 | -1.43 | -1.22 | -1.53 |
| AGGF1 | angiogenic factor with G patch and FHA domains 1 (AGGF1) | NM_018046 | 0.01780 | -1.23 | -1.05 | -1.40 |
| PCGF3 | polycomb group ring finger 3 (PCGF3) | NM_006315 | 0.04862 | -1.15 | -1.09 | -1.32 |
| C14orf156 | chromosome 14 open reading frame 156 (C14orf156) | NM_031210 | 0.02193 | -1.11 | -1.06 | -1.89 |
| ZNF415 | zinc finger protein 415 (ZNF415) | NM_018355 | 0.02841 | -1.45 | -1.20 | -1.73 |
| ATP5J | ATP synthase, H+ transporting, mitochondrial F0 complex, subunit F6 (ATP5J), nuclear gene encoding mitochondrial protein, transcript variant 1 | NM_001003703 | 0.01314 | -1.31 | -1.16 | -2.37 |
| ATF7IP | activating transcription factor 7 interacting protein (ATF7IP) | NM_018179 | 0.01603 | -1.31 | -1.25 | -2.02 |
| RAD50 | RAD50 homolog (S. cerevisiae) (RAD50), transcript variant 1 | NM_005732 | 0.00942 | -1.44 | -1.41 | -2.29 |
| DNTTIP2 | deoxynucleotidyltransferase, terminal, interacting protein 2 (DNTTIP2) | NM_014597 | 0.02217 | -1.20 | -1.02 | -1.86 |
| GATAD2A | GATA zinc finger domain containing 2A (GATAD2A) | NM_017660 | 0.00481 | -1.08 | -1.18 | -1.55 |
| HNRNPA1L2 | heterogeneous nuclear ribonucleoprotein A1-like (LOC144983), transcript variant 1 | NM_001011724 | 0.00731 | 1.29 | -1.06 | -13.88 |
| DDX1 | DEAD (Asp-Glu-Ala-Asp) box polypeptide 1 (DDX1) | NM_004939 | 0.03701 | -1.26 | 1.06 | -1.47 |
| EXOSC8 | exosome component 8 (EXOSC8) | NM_181503 | 0.00081 | -1.25 | 1.03 | -3.36 |
| GIN1 | zinc finger, H2C2 domain containing (ZH2C2) | NM_017676 | 0.01541 | -1.36 | 1.07 | -1.22 |
| XBP1 | X-box binding protein 1 (XBP1), transcript variant 1 | NM_005080 | 0.03074 | -1.17 | 1.06 | -1.56 |
| PRPF38A | PRP38 pre-mRNA processing factor 38 (yeast) domain containing A (PRPF38A), transcript variant 1 | NM_032284 | 0.02516 | -1.22 | 1.04 | -1.69 |
| SMARCB1 | SWI/SNF related, matrix associated, actin dependent regulator of chromatin, subfamily b, member 1 (SMARCB1), transcript variant 1 | NM_003073 | 0.02333 | -1.30 | 1.06 | -1.25 |
| ATP5C1 | ATP synthase, H+ transporting, mitochondrial F1 complex, gamma polypeptide 1 (ATP5C1), nuclear gene encoding mitochondrial protein, transcript variant 2 | NM_005174 | 0.00004 | -1.32 | 1.12 | -8.16 |
| SUV39H2 | suppressor of variegation 3-9 homolog 2 (Drosophila) (SUV39H2) | NM_024670 | 0.02553 | -1.81 | 1.06 | -1.15 |
| ATP6V1A | ATPase, H+ transporting, lysosomal 70kDa, V1 subunit A (ATP6V1A) | NM_001690 | 0.01291 | -1.40 | 1.03 | -1.36 |
| PBRM1 | polybromo 1 (PB1), transcript variant 2 | NM_018313 | 0.00058 | -1.33 | 1.04 | -2.40 |
| PAPD4 | PAP associated domain containing 4 (PAPD4) | NM_173797 | 0.04849 | -1.96 | 1.02 | -2.77 |
| ELP4 | elongation protein 4 homolog (S. cerevisiae) (ELP4) | NM_019040 | 0.01461 | -1.34 | 1.22 | -2.02 |
| ZNF669 | zinc finger protein 669 (ZNF669) | NM_024804 | 0.03033 | -1.35 | 1.24 | -3.41 |
| MSH6 | mutS homolog 6 (E. coli) (MSH6) | NM_000179 | 0.00006 | -1.23 | 1.05 | -4.24 |
| SNRPD1 | small nuclear ribonucleoprotein D1 polypeptide 16kDa (SNRPD1) | NM_006938 | 0.04360 | -1.28 | 1.15 | -1.79 |
| EXOSC9 | exosome component 9 (EXOSC9), transcript variant 2 | NM_005033 | 0.04327 | -1.27 | 1.04 | -1.56 |
| MIER3 | mesoderm induction early response 1, family member 3 (MIER3) | NM_152622 | 0.04153 | -2.54 | 1.04 | -2.00 |
| BAT1 | HLA-B associated transcript 1 (BAT1), transcript variant 1 | NM_004640 | 0.04305 | -1.40 | 1.18 | -2.05 |
| ZNF818P | zinc finger protein 818 (ZNF818) | NM_001001675 | 0.01072 | 1.77 | 1.15 | -6.33 |
| HIF1AN | hypoxia-inducible factor 1, alpha subunit inhibitor (HIF1AN) | NM_017902 | 0.03239 | 1.38 | 1.48 | -1.06 |
| SP7 | Sp7 transcription factor (SP7) | NM_152860 | 0.00031 | 2.60 | 1.62 | -1.09 |
| ATXN7 | ataxin 7 (ATXN7) | NM_000333 | 0.03204 | 1.74 | 1.22 | -1.00 |
| LBH | limb bud and heart development homolog (mouse) (LBH) | NM_030915 | 0.01253 | -1.41 | -1.83 | 1.21 |
| ARID1A | AT rich interactive domain 1A (SWI-like) (ARID1A), transcript variant 1 | NM_006015 | 0.04678 | -1.03 | -1.05 | 2.75 |
| ZNF628 | zinc finger protein 628 (ZNF628) | NM_033113 | 0.04951 | 1.50 | -1.17 | 1.68 |
| GATA2 | GATA binding protein 2 (GATA2) | NM_032638 | 0.04229 | 1.46 | -1.02 | 1.61 |
| ZNF282 | zinc finger protein 282 (ZNF282) | NM_003575 | 0.03366 | 1.46 | -1.03 | 1.52 |
| SALL1 | sal-like 1 (Drosophila) (SALL1) | NM_002968 | 0.01990 | 1.27 | -1.03 | 2.36 |
| KLF16 | Kruppel-like factor 16 (KLF16) | NM_031918 | 0.00608 | -1.05 | 1.01 | 1.86 |
| ZNF532 | zinc finger protein 532 (ZNF532) | NM_018181 | 0.03873 | -1.02 | 1.26 | 1.43 |
| RBM10 | RNA binding motif protein 10 (RBM10), transcript variant 1 | NM_005676 | 0.03404 | -1.06 | 1.25 | 1.43 |
| MAF1 | MAF1 homolog (S. cerevisiae) (MAF1) | NM_032272 | 0.04372 | -1.02 | 1.23 | 1.54 |
| TREX1 | three prime repair exonuclease 1 (TREX1), transcript variant 1 | NM_016381 | 0.02127 | -1.02 | 1.30 | 1.65 |
| PELP1 | proline, glutamic acid and leucine rich protein 1 (PELP1) | NM_014389 | 0.04002 | -1.02 | 1.12 | 1.50 |
| ATP13A1 | ATPase type 13A1 (ATP13A1) | NM_020410 | 0.04130 | -1.05 | 1.00 | 2.06 |
| SLC29A2 | solute carrier family 29 (nucleoside transporters), member 2 (SLC29A2) | NM_001532 | 0.02467 | -1.03 | 1.95 | 1.88 |
| GEMIN4 | gem (nuclear organelle) associated protein 4 (GEMIN4) | NM_015721 | 0.02022 | -1.04 | 1.08 | 1.61 |
| GPBP1L1 | GC-rich promoter binding protein 1-like 1 (GPBP1L1) | NM_021639 | 0.00634 | 1.48 | 1.21 | 1.35 |
| TOP3B | topoisomerase (DNA) III beta (TOP3B) | NM_003935 | 0.00771 | 1.44 | 1.23 | 1.43 |
| PAF1 | Paf1, RNA polymerase II associated factor, homolog (S. cerevisiae) (PAF1) | NM_019088 | 0.01051 | 1.27 | 1.17 | 1.09 |
| MZF1 | myeloid zinc finger 1 (MZF1), transcript variant 2 | NM_198055 | 0.00472 | 1.41 | 1.38 | 1.80 |
| RXRB | retinoid X receptor, beta (RXRB) | NM_021976 | 0.02635 | 1.15 | 1.15 | 1.12 |
| ZNF787 | zinc finger protein 787 (ZNF787) | NM_001002836 | 0.04737 | 1.00 | 1.26 | 1.74 |
| SETDB1 | SET domain, bifurcated 1 (SETDB1) | NM_012432 | 0.01145 | 1.12 | 1.07 | 1.40 |
| TERF2IP | telomeric repeat binding factor 2, interacting protein (TERF2IP) | NM_018975 | 0.00473 | 1.24 | 1.34 | 1.39 |
| RBM4B | RNA binding motif protein 4B (RBM4B) | NM_031492 | 0.01192 | 1.28 | 1.17 | 1.40 |
| PTOV1 | prostate tumor overexpressed gene 1 (PTOV1) | NM_017432 | 0.00137 | 1.36 | 1.30 | 1.47 |
| PABPN1 | poly(A) binding protein, nuclear 1 (PABPN1) | NM_004643 | 0.03715 | 1.22 | 1.39 | 1.27 |
| ATF6B | cAMP responsive element binding protein-like 1 (CREBL1) | NM_004381 | 0.04260 | 1.06 | 1.01 | 1.32 |
| TCF25 | transcription factor 25 (basic helix-loop-helix) (TCF25) | NM_014972 | 0.01568 | 1.05 | 1.11 | 1.58 |
| ZNF689 | zinc finger protein 689 (ZNF689) | NM_138447 | 0.00243 | 1.09 | 1.34 | 1.53 |
| ILF3 | interleukin enhancer binding factor 3, 90kDa (ILF3), transcript variant 1 | NM_012218 | 0.02209 | 1.31 | 1.20 | 1.38 |
| POLL | polymerase (DNA directed), lambda (POLL) | NM_013274 | 0.03751 | 1.30 | 1.15 | 1.37 |
| ZNF3 | zinc finger protein 3 (ZNF3), transcript variant 2 | NM_032924 | 0.02294 | 1.33 | 1.14 | 1.19 |
| ZIK1 | zinc finger protein interacting with K protein 1 homolog (mouse) (ZIK1) | NM_001010879 | 0.00940 | 1.32 | 1.22 | 1.13 |
| ELOF1 | elongation factor 1 homolog (S. cerevisiae) (ELOF1) | NM_032377 | 0.02289 | 1.04 | 1.24 | 1.31 |
| SMAD6 | SMAD family member 6 (SMAD6) | NM_005585 | 0.04927 | 1.62 | 1.11 | 1.52 |
| KCTD13 | potassium channel tetramerisation domain containing 13 (KCTD13) | NM_178863 | 0.00225 | 1.33 | 1.08 | 1.35 |
| ZNF524 | zinc finger protein 524 (ZNF524) | NM_153219 | 0.02297 | 1.19 | 1.11 | 1.59 |
| ZNF584 | zinc finger protein 584 (ZNF584) | NM_173548 | 0.00535 | 1.59 | 1.09 | 1.19 |
| ZNF454 | zinc finger protein 454 (ZNF454) | NM_182594 | 0.01111 | 1.25 | 1.26 | 1.39 |
| SUPT6H | suppressor of Ty 6 homolog (S. cerevisiae) (SUPT6H) | NM_003170 | 0.00198 | 1.16 | 1.31 | 1.58 |
| ZNF384 | zinc finger protein 384 (ZNF384), transcript variant 2 | NM_133476 | 0.02564 | 1.20 | 1.06 | 1.41 |
| RRP1B | KIAA0179 (KIAA0179) | NM_015056 | 0.01963 | 1.11 | 1.63 | 2.13 |
| C17orf70 | chromosome 17 open reading frame 70 (C17orf70) | NM_025161 | 0.03581 | 1.22 | 1.10 | 1.47 |
| SNRNP200 | activating signal cointegrator 1 complex subunit 3-like 1 (ASCC3L1) | NM_014014 | 0.01461 | 1.27 | 1.53 | 1.02 |
| ZNF408 | zinc finger protein 408 (ZNF408) | NM_024741 | 0.03998 | 1.05 | 1.13 | 1.35 |
| CRTC3 | CREB regulated transcription coactivator 3 (CRTC3), transcript variant 1 | NM_022769 | 0.00212 | 1.23 | 1.18 | 1.83 |
| CRTC3 | CREB regulated transcription coactivator 3 (CRTC3), transcript variant 1 | NM_022769 | 0.00250 | 1.31 | 1.19 | 1.51 |
| ZNF76 | zinc finger protein 76 (expressed in testis) (ZNF76) | NM_003427 | 0.03936 | 1.18 | 1.16 | 1.23 |
| ADARB1 | adenosine deaminase, RNA-specific, B1 (RED1 homolog rat) (ADARB1), transcript variant 2 | NM_015833 | 0.00879 | 1.56 | 1.13 | 1.58 |
| SFRS17A | chromosome X and Y open reading frame 3 (CXYorf3) | NM_005088 | 0.01276 | 1.27 | 1.13 | 1.93 |
| IKZF4 | IKAROS family zinc finger 4 (Eos) (IKZF4) | NM_022465 | 0.04518 | 1.08 | 1.28 | 1.36 |
| ZNF512B | zinc finger protein 512B (ZNF512B) | NM_020713 | 0.00947 | 1.47 | 1.21 | 1.37 |
| ZBTB48 | zinc finger and BTB domain containing 48 (ZBTB48) | NM_005341 | 0.00816 | 1.02 | 1.19 | 1.94 |
| AKNA | AT-hook transcription factor (AKNA) | NM_030767 | 0.03284 | 1.13 | 1.11 | 1.82 |
| RERE | arginine-glutamic acid dipeptide (RE) repeats (RERE), transcript variant 1 | NM_012102 | 0.03463 | 1.08 | 1.28 | 1.36 |
| DHX38 | DEAH (Asp-Glu-Ala-His) box polypeptide 38 (DHX38) | NM_014003 | 0.00564 | 1.12 | 1.06 | 1.67 |
| ZRSR2 | zinc finger (CCCH type), RNA-binding motif and serine/arginine rich 2 (ZRSR2) | NM_005089 | 0.00682 | 1.19 | 1.21 | 1.39 |
| RRP1 | DNA segment on chromosome 21 (unique) 2056 expressed sequence (D21S2056E) | NM_003683 | 0.01699 | 1.06 | 1.13 | 1.80 |
| MEN1 | multiple endocrine neoplasia I (MEN1), transcript variant e1E | NM_130803 | 0.02186 | 1.20 | 1.00 | 2.01 |
| IGHMBP2 | immunoglobulin mu binding protein 2 (IGHMBP2) | NM_002180 | 0.01602 | 1.52 | 1.71 | 2.28 |
| TFIP11 | tuftelin interacting protein 11 (TFIP11), transcript variant 1 | NM_001008697 | 0.00544 | 1.08 | 1.06 | 1.26 |
| ZNF687 | zinc finger protein 687 (ZNF687) | NM_020832 | 0.00303 | 1.25 | 1.11 | 1.49 |
| ZNF343 | zinc finger protein 343 (ZNF343) | NM_024325 | 0.02809 | 1.10 | 1.04 | 2.01 |
| ZSCAN21 | zinc finger and SCAN domain containing 21 (ZSCAN21) | NM_145914 | 0.01461 | 1.12 | 1.07 | 1.25 |
| NT5C3L | 5'-nucleotidase, cytosolic III-like (NT5C3L) | NM_052935 | 0.04996 | 1.16 | 1.08 | 1.49 |
| SRRM2 | serine/arginine repetitive matrix 2 (SRRM2) | NM_016333 | 0.04399 | 1.22 | 1.33 | 1.51 |
| FOXB1 | forkhead box B1 (FOXB1) | NM_012182 | 0.04351 | 1.58 | 1.22 | 1.76 |
| ZNF311 | zinc finger protein 311 (ZNF311) | NM_001010877 | 0.04832 | 1.13 | 1.21 | 1.52 |
| TXNL4B | thioredoxin-like 4B (TXNL4B) | NM_017853 | 0.00131 | 1.48 | 1.32 | 1.42 |
| CXXC1 | CXXC finger 1 (PHD domain) (CXXC1) | NM_014593 | 0.00108 | 1.15 | 1.13 | 1.55 |
| XRCC3 | X-ray repair complementing defective repair in Chinese hamster cells 3 (XRCC3) | NM_005432 | 0.00212 | 1.77 | 1.20 | 1.38 |
| PHF17 | PHD finger protein 17 (PHF17), transcript variant S | NM_024900 | 0.04757 | 1.13 | 1.26 | 1.39 |
| ZNF263 | zinc finger protein 263 (ZNF263) | NM_005741 | 0.00494 | 1.13 | 1.01 | 1.91 |
| CASZ1 | castor zinc finger 1 (CASZ1), transcript variant 2 | NM_017766 | 0.03555 | 1.19 | 1.18 | 1.93 |
| FOXJ2 | forkhead box J2 (FOXJ2) | NM_018416 | 0.00031 | 1.26 | 1.25 | 1.42 |
| EZH1 | enhancer of zeste homolog 1 (Drosophila) (EZH1) | NM_001991 | 0.00192 | 1.44 | 1.53 | 1.55 |
| ZNF509 | zinc finger protein 509 (ZNF509) | NM_145291 | 0.04395 | 1.02 | 1.08 | 1.38 |
| ZBTB4 | zinc finger and BTB domain containing 4 (ZBTB4) | NM_020899 | 0.04796 | 1.31 | 1.05 | 1.38 |
| ZNF671 | zinc finger protein 671 (ZNF671) | NM_024833 | 0.04853 | 1.18 | 1.17 | 1.35 |
| ZSCAN10 | zinc finger and SCAN domain containing 10 (ZSCAN10) | NM_032805 | 0.00754 | 1.55 | 1.39 | 1.19 |
| FANCG | Fanconi anemia, complementation group G (FANCG) | NM_004629 | 0.01159 | 1.19 | 1.14 | 1.28 |
| PRPF40B | PRP40 pre-mRNA processing factor 40 homolog B (S. cerevisiae) (PRPF40B), transcript variant 2 | NM_012272 | 0.01591 | 1.09 | 1.39 | 1.57 |
| NEIL1 | nei endonuclease VIII-like 1 (E. coli) (NEIL1) | NM_024608 | 0.00164 | 1.46 | 1.36 | 1.75 |
| TSEN34 | tRNA splicing endonuclease 34 homolog (S. cerevisiae) (TSEN34), transcript variant 1 | NM_024075 | 0.00423 | 1.25 | 1.21 | 1.26 |
| KLF7 | Kruppel-like factor 7 (ubiquitous) (KLF7) | NM_003709 | 0.00705 | 1.01 | 1.21 | 1.87 |
| ZNF276 | zinc finger protein 276 (ZNF276) | NM_152287 | 0.00007 | 1.22 | 1.14 | 1.42 |
| PRPF6 | PRP6 pre-mRNA processing factor 6 homolog (S. cerevisiae) (PRPF6) | NM_012469 | 0.00236 | 1.30 | 1.15 | 1.67 |
| ZBTB46 | zinc finger and BTB domain containing 46 (ZBTB46) | NM_025224 | 0.00246 | 1.63 | 1.14 | 1.84 |
| PUS1 | pseudouridylate synthase 1 (PUS1), transcript variant 1 | NM_025215 | 0.04541 | 1.07 | 1.29 | 1.49 |
| TSEN54 | tRNA splicing endonuclease 54 homolog (S. cerevisiae) (TSEN54) | NM_207346 | 0.00015 | 1.43 | 1.27 | 1.27 |
| SFRS16 | splicing factor, arginine/serine-rich 16 (SFRS16) | NM_007056 | 0.00558 | 1.22 | 1.14 | 2.13 |
| ZBTB22 | zinc finger and BTB domain containing 22 (ZBTB22) | NM_005453 | 0.00147 | 1.20 | 1.31 | 1.44 |
| ZNF224 | zinc finger protein 224 (ZNF224) | NM_013398 | 0.01066 | 1.23 | 1.50 | 1.92 |
| FTSJ3 | FtsJ homolog 3 (E. coli) (FTSJ3) | NM_017647 | 0.01848 | 1.05 | 1.26 | 1.82 |
| DGCR8 | DiGeorge syndrome critical region gene 8 (DGCR8) | NM_022720 | 0.00740 | 1.18 | 1.40 | 1.50 |
| C1orf83 | chromosome 1 open reading frame 83 (C1orf83) | NM_153035 | 0.01530 | 1.59 | 1.28 | 1.38 |
| RPA4 | replication protein A4, 34kDa (RPA4) | NM_013347 | 0.02932 | 1.23 | 1.32 | 1.50 |
| RBM6 | RNA binding motif protein 6 (RBM6) | NM_005777 | 0.00398 | 1.23 | 1.24 | 1.49 |
| RNASEN | ribonuclease III, nuclear (RNASEN) | NM_013235 | 0.00130 | 1.25 | 1.42 | 1.16 |
| ZMIZ1 | zinc finger, MIZ-type containing 1 (ZMIZ1) | NM_020338 | 0.03273 | 1.50 | 1.07 | 1.42 |
| ZNF16 | zinc finger protein 16 (ZNF16), transcript variant 1 | NM_006958 | 0.00244 | 1.32 | 1.32 | 1.72 |
| ZNF784 | zinc finger protein 784 (ZNF784) | NM_203374 | 0.00385 | 1.23 | 1.21 | 1.53 |
| DGCR14 | DiGeorge syndrome critical region gene 14 (DGCR14) | NM_022719 | 0.03413 | 1.13 | 1.02 | 1.49 |
| SMC1A | structural maintenance of chromosomes 1A (SMC1A) | NM_006306 | 0.00931 | 1.34 | 1.48 | 1.42 |
| ZNF434 | zinc finger protein 434 (ZNF434) | NM_017810 | 0.03639 | 1.24 | 1.17 | 1.23 |
| DDX24 | DEAD (Asp-Glu-Ala-Asp) box polypeptide 24 (DDX24) | NM_020414 | 0.01991 | 1.83 | 1.22 | 1.65 |
| NT5C2 | 5'-nucleotidase, cytosolic II (NT5C2) | NM_012229 | 0.01129 | 1.70 | 1.13 | 1.20 |
| SFRS5 | splicing factor, arginine/serine-rich 5 (SFRS5), transcript variant 2 | NM_006925 | 0.00667 | 1.36 | 1.62 | 1.58 |
| BAZ2A | bromodomain adjacent to zinc finger domain, 2A (BAZ2A) | NM_013449 | 0.02121 | 1.21 | 1.00 | 1.92 |
| TNRC4 | trinucleotide repeat containing 4 (TNRC4) | NM_007185 | 0.00593 | 1.21 | 2.93 | 1.40 |
| CBX6 | chromobox homolog 6 (CBX6) | NM_014292 | 0.02565 | 1.33 | 1.20 | 1.42 |
| IMP4 | IMP4, U3 small nucleolar ribonucleoprotein, homolog (yeast) (IMP4) | NM_033416 | 0.00858 | 1.27 | 1.18 | 1.45 |
| TEAD1 | TEA domain family member 1 (SV40 transcriptional enhancer factor) (TEAD1) | NM_021961 | 0.01767 | 1.21 | 1.19 | 1.53 |
| ZNF579 | zinc finger protein 579 (ZNF579) | NM_152600 | 0.02773 | 1.20 | 1.06 | 1.97 |
| DHX35 | DEAH (Asp-Glu-Ala-His) box polypeptide 35 (DHX35) | NM_021931 | 0.04162 | 1.09 | 1.22 | 1.21 |
| ADRA1D | adrenergic, alpha-1D-, receptor (ADRA1D) | NM_000678 | 0.03044 | 1.26 | 1.68 | 1.30 |
| ADCY5 | adenylate cyclase 5 (ADCY5) | NM_183357 | 0.03632 | 1.24 | 1.57 | 1.41 |
| NR1H3 | nuclear receptor subfamily 1, group H, member 3 (NR1H3) | NM_005693 | 0.00312 | 1.78 | 1.08 | 1.61 |
| HIP1 | huntingtin interacting protein 1 (HIP1) | NM_005338 | 0.00640 | 1.07 | 1.51 | 1.87 |
| ZNF653 | zinc finger protein 653 (ZNF653) | NM_138783 | 0.01951 | 1.08 | 1.19 | 1.68 |
| RNMTL1 | RNA methyltransferase like 1 (RNMTL1) | NM_018146 | 0.00085 | 1.48 | 1.26 | 1.15 |
| RFXANK | regulatory factor X-associated ankyrin-containing protein (RFXANK), transcript variant 1 | NM_003721 | 0.03702 | 1.22 | 1.23 | 1.14 |
| SIN3A | SIN3 homolog A, transcription regulator (yeast) (SIN3A) | NM_015477 | 0.00503 | 1.21 | 1.10 | 1.58 |
| ZNF335 | zinc finger protein 335 (ZNF335) | NM_022095 | 0.00059 | 1.18 | 1.26 | 2.37 |
| MLL4 | myeloid/lymphoid or mixed-lineage leukemia 4 (MLL4) | NM_014727 | 0.00030 | 1.36 | 1.08 | 2.18 |
| PSMC3IP | PSMC3 interacting protein (PSMC3IP), transcript variant 1 | NM_013290 | 0.00030 | 1.35 | 1.13 | 1.60 |
| SEC14L2 | SEC14-like 2 (S. cerevisiae) (SEC14L2) | NM_012429 | 0.03843 | 1.45 | 1.17 | 1.25 |
| MAML2 | mastermind-like 2 (Drosophila) (MAML2) | NM_032427 | 0.03930 | 2.26 | 1.75 | 1.34 |
| RBL1 | retinoblastoma-like 1 (p107) (RBL1), transcript variant 1 | NM_002895 | 0.01171 | 1.53 | 1.46 | 1.21 |
| A2BP1 | ataxin 2-binding protein 1 (A2BP1), transcript variant 3 | NM_145893 | 0.02019 | 1.08 | 1.78 | 1.62 |
| ZNF318 | zinc finger protein 318 (ZNF318) | NM_014345 | 0.02212 | 1.29 | 1.17 | 1.18 |
| TSHZ2 | teashirt family zinc finger 2 (TSHZ2) | NM_173485 | 0.01272 | 1.15 | 1.67 | 1.25 |
| ZNF10 | zinc finger protein 10 (ZNF10) | NM_015394 | 0.04661 | 1.03 | 1.44 | 1.21 |
| ZNF324 | zinc finger protein 324 (ZNF324) | NM_014347 | 0.02522 | 1.30 | 1.13 | 1.52 |
| IKBKG | inhibitor of kappa light polypeptide gene enhancer in B-cells, kinase gamma (IKBKG) | NM_003639 | 0.00194 | 1.11 | 1.24 | 2.19 |
| PHF1 | PHD finger protein 1 (PHF1), transcript variant 2 | NM_024165 | 0.00283 | 1.22 | 1.19 | 1.68 |
| NOL8 | nucleolar protein 8 (NOL8) | NM_017948 | 0.03718 | 1.23 | 1.17 | 1.10 |
| **Other Cellular Metabolic Process** | |  |  |  |  |  |
| NDUFB4 | NADH dehydrogenase (ubiquinone) 1 beta subcomplex, 4, 15kDa (NDUFB4), nuclear gene encoding mitochondrial protein | NM_004547 | 0.01330 | -1.17 | -1.05 | -1.53 |
| TOLLIP | toll interacting protein (TOLLIP) | NM_019009 | 0.00016 | -1.16 | -1.01 | -12.68 |
| STEAP1 | six transmembrane epithelial antigen of the prostate 1 (STEAP1) | NM_012449 | 0.03183 | -1.39 | -1.24 | -5.10 |
| CDKN3 | cyclin-dependent kinase inhibitor 3 (CDK2-associated dual specificity phosphatase) (CDKN3) | NM_005192 | 0.01178 | -1.46 | -1.23 | -2.21 |
| NDUFV2 | NADH dehydrogenase (ubiquinone) flavoprotein 2, 24kDa (NDUFV2) | NM_021074 | 0.00150 | -1.32 | -1.02 | -2.36 |
| ACYP2 | acylphosphatase 2, muscle type (ACYP2) | NM_138448 | 0.04188 | -1.27 | -1.99 | -1.21 |
| UGT1A6 | UDP glucuronosyltransferase 1 family, polypeptide A6 (UGT1A6), transcript variant 1 | NM_001072 | 0.04007 | -2.07 | -3.14 | -1.40 |
| GC | group-specific component (vitamin D binding protein) (GC) | NM_000583 | 0.01880 | 1.06 | -1.02 | -7.62 |
| ACMSD | aminocarboxymuconate semialdehyde decarboxylase (ACMSD) | NM_138326 | 0.00020 | 1.12 | -1.10 | -14.31 |
| NDUFV3 | NADH dehydrogenase (ubiquinone) flavoprotein 3, 10kDa (NDUFV3), nuclear gene encoding mitochondrial protein, transcript variant 1 | NM_021075 | 0.02105 | -1.25 | 1.03 | -1.58 |
| CYB5R4 | cytochrome b5 reductase 4 (CYB5R4) | NM_016230 | 0.04198 | -1.46 | 1.13 | -1.50 |
| ETFDH | electron-transferring-flavoprotein dehydrogenase (ETFDH), nuclear gene encoding mitochondrial protein | NM_004453 | 0.00160 | -1.42 | 1.09 | -6.04 |
| SLC25A4 | solute carrier family 25 (mitochondrial carrier; adenine nucleotide translocator), member 4 (SLC25A4), nuclear gene encoding mitochondrial protein | NM_001151 | 0.04586 | -1.38 | 1.00 | -1.84 |
| NDUFB1 | NADH dehydrogenase (ubiquinone) 1 beta subcomplex, 1, 7kDa (NDUFB1) | NM_004545 | 0.00021 | -1.40 | 1.28 | -11.29 |
| NDUFC1 | NADH dehydrogenase (ubiquinone) 1, subcomplex unknown, 1, 6kDa (NDUFC1) | NM_002494 | 0.00019 | -1.42 | 1.10 | -18.79 |
| CYB5A | cytochrome b5 type A (microsomal) (CYB5A), transcript variant 2 | NM_001914 | 0.01558 | -1.35 | 1.09 | -1.71 |
| GDE1 | membrane interacting protein of RGS16 (MIR16) | NM_016641 | 0.03918 | 1.19 | 1.20 | -11.54 |
| CA2 | carbonic anhydrase II (CA2) | NM_000067 | 0.03334 | -3.57 | -3.48 | 1.19 |
| AIFM3 | apoptosis-inducing factor, mitochondrion-associated, 3 (AIFM3), transcript variant 1 | NM_144704 | 0.03202 | 1.20 | 1.30 | 1.10 |
| GDPD5 | glycerophosphodiester phosphodiesterase domain containing 5 (GDPD5) | NM_030792 | 0.01446 | 1.31 | 1.07 | 1.51 |
| UCRC | ubiquinol-cytochrome c reductase complex (7.2 kD) (UCRC), transcript variant 2 | NM_001003684 | 0.03456 | 1.36 | 1.34 | 1.05 |
| ADH6 | alcohol dehydrogenase 6 (class V) (ADH6) | NM_000672 | 0.04882 | 1.32 | 1.26 | 1.16 |
| ITPK1 | inositol 1,3,4-triphosphate 5/6 kinase (ITPK1) | NM_014216 | 0.01254 | 1.05 | 1.26 | 2.31 |
| SFTPD | surfactant, pulmonary-associated protein D (SFTPD) | NM_003019 | 0.01652 | 1.87 | 1.30 | 1.41 |
| CSAD | cysteine sulfinic acid decarboxylase (CSAD) | NM_015989 | 0.01097 | 1.80 | 2.21 | 1.18 |
| CCS | copper chaperone for superoxide dismutase (CCS) | NM_005125 | 0.04297 | 1.13 | 1.19 | 1.51 |
| GDPD3 | glycerophosphodiester phosphodiesterase domain containing 3 (GDPD3), transcript variant 2 | NM_024307 | 0.01471 | 1.49 | 1.11 | 1.45 |
| ITPKA | inositol 1,4,5-trisphosphate 3-kinase A (ITPKA) | NM_002220 | 0.00090 | 1.84 | 1.22 | 1.22 |
| NM_015636 | eukaryotic translation initiation factor 2B, subunit 4 delta, 67kDa (EIF2B4), transcript variant 1 | NM_015636 | 0.049772 | 1.043 | 1.188 | 1.237 |
| **Other Metabolic Process** | |  |  |  |  |  |
| C8orf38 | chromosome 8 open reading frame 38 (C8orf38) | NM_152416 | 0.00000 | -2.50 | -1.04 | -6.08 |
| GPX7 | glutathione peroxidase 7 (GPX7) | NM_015696 | 0.01135 | -1.58 | -1.04 | -1.71 |
| ABHD5 | abhydrolase domain containing 5 (ABHD5) | NM_016006 | 0.04973 | -1.23 | -1.48 | -1.25 |
| CLN5 | ceroid-lipofuscinosis, neuronal 5 (CLN5) | NM_006493 | 0.00615 | -1.47 | -1.27 | -1.43 |
| ISOC1 | isochorismatase domain containing 1 (ISOC1) | NM_016048 | 0.04014 | -1.87 | -1.08 | -1.54 |
| DHRS7 | dehydrogenase/reductase (SDR family) member 7 (DHRS7) | NM_016029 | 0.00113 | -1.40 | -1.16 | -3.22 |
| AADACL1 | arylacetamide deacetylase-like 1 (AADACL1) | NM_020792 | 0.01792 | -2.62 | -1.12 | -1.47 |
| MAN1A1 | mannosidase, alpha, class 1A, member 1 (MAN1A1) | NM_005907 | 0.02009 | -1.19 | -1.40 | -1.28 |
| METTL13 | KIAA0859 (KIAA0859), transcript variant 1 | NM_015935 | 0.03794 | -1.30 | -1.00 | -1.37 |
| DDHD1 | DDHD domain containing 1 (DDHD1) | NM_030637 | 0.00692 | -1.90 | -1.38 | -1.51 |
| CP | ceruloplasmin (ferroxidase) (CP) | NM_000096 | 0.00014 | -1.12 | -1.27 | -13.29 |
| EGLN3 | egl nine homolog 3 (C. elegans) (EGLN3) | NM_022073 | 0.04535 | -1.66 | -1.22 | -1.50 |
| SLC2A3 | solute carrier family 2 (facilitated glucose transporter), member 3 (SLC2A3) | NM_006931 | 0.01525 | -1.42 | -1.60 | -1.46 |
| ENPP4 | ectonucleotide pyrophosphatase/phosphodiesterase 4 (putative function) (ENPP4) | NM_014936 | 0.02391 | -1.76 | -1.22 | -1.10 |
| P4HA1 | procollagen-proline, 2-oxoglutarate 4-dioxygenase (proline 4-hydroxylase), alpha polypeptide I (P4HA1), transcript variant 1 | NM_000917 | 0.04527 | -1.44 | -1.31 | -1.53 |
| NAT1 | N-acetyltransferase 1 (arylamine N-acetyltransferase) (NAT1) | NM_000662 | 0.01730 | 1.15 | -1.75 | -2.53 |
| NIT2 | nitrilase family, member 2 (NIT2) | NM_020202 | 0.00003 | -1.44 | 1.04 | -4.24 |
| UCKL1 | uridine-cytidine kinase 1-like 1 (UCKL1) | NM_017859 | 0.01129 | 1.27 | 1.14 | -3.66 |
| HEXDC | hexosaminidase (glycosyl hydrolase family 20, catalytic domain) containing (HEXDC) | NM_173620 | 0.01392 | -1.10 | 1.37 | 1.85 |
| HEPH | hephaestin (HEPH), transcript variant 2 | NM_014799 | 0.04977 | -1.04 | 1.40 | 1.38 |
| SDR39U1 | chromosome 14 open reading frame 124 (C14orf124) | NM_020195 | 0.04106 | -1.05 | 1.50 | 1.43 |
| RDH16 | retinol dehydrogenase 16 (all-trans) (RDH16) | NM_003708 | 0.04438 | 2.02 | 1.22 | 1.18 |
| SLC5A2 | solute carrier family 5 (sodium/glucose cotransporter), member 2 (SLC5A2) | NM_003041 | 0.00018 | 1.71 | 1.55 | 1.54 |
| CPNE1 | copine I (CPNE1), transcript variant 7 | NM_152930 | 0.01106 | 1.35 | 1.32 | 1.44 |
| PCCA | propionyl Coenzyme A carboxylase, alpha polypeptide (PCCA) | NM_000282 | 0.02004 | 1.10 | 1.49 | 1.08 |
| HYAL3 | hyaluronoglucosaminidase 3 (HYAL3) | NM_003549 | 0.03417 | 1.14 | 1.02 | 1.76 |
| LCN15 | MSFL2541 (UNQ2541) | NM_203347 | 0.00529 | 1.89 | 5.35 | 2.22 |
| SLC2A4 | solute carrier family 2 (facilitated glucose transporter), member 4 (SLC2A4) | NM_001042 | 0.01165 | 1.25 | 1.66 | 1.04 |
| TRMT2A | HpaII tiny fragments locus 9C (HTF9C), transcript variant 1 | NM_022727 | 0.00161 | 1.17 | 1.17 | 1.48 |
| UGT3A1 | UDP glycosyltransferase 3 family, polypeptide A1 (UGT3A1) | NM_152404 | 0.01232 | 1.69 | 1.26 | 5.83 |
| OGFOD2 | 2-oxoglutarate and iron-dependent oxygenase domain containing 2 (OGFOD2) | NM_024623 | 0.01947 | 1.29 | 1.48 | 1.30 |
| FOXRED2 | FAD-dependent oxidoreductase domain containing 2 (FOXRED2) | NM_024955 | 0.00012 | 1.48 | 1.28 | 1.58 |
| GPT2 | glutamic pyruvate transaminase (alanine aminotransferase) 2 (GPT2) | NM_133443 | 0.00826 | 1.63 | 1.54 | 1.71 |
| PLD2 | phospholipase D2 (PLD2) | NM_002663 | 0.02684 | 1.18 | 1.10 | 1.67 |
| SCLY | selenocysteine lyase (SCLY) | NM_016510 | 0.01393 | 1.15 | 1.32 | 1.44 |
| SHPK | carbohydrate kinase-like (CARKL) | NM_013276 | 0.01378 | 1.06 | 1.26 | 1.84 |
| **Transport** |  |  |  |  |  |  |
| SNX16 | sorting nexin 16 (SNX16), transcript variant 1 | NM_022133 | 0.03428 | -1.28 | -1.23 | -1.52 |
| FLJ11506 | hypothetical protein FLJ11506 (FLJ11506) | NM_024666 | 0.04792 | -1.66 | -1.04 | -1.14 |
| NUP54 | nucleoporin 54kDa (NUP54) | NM_017426 | 0.03089 | -1.04 | -1.07 | -1.44 |
| GOLT1B | golgi transport 1 homolog B (S. cerevisiae) (GOLT1B) | NM_016072 | 0.01639 | -1.68 | -1.03 | -1.45 |
| SLC36A1 | solute carrier family 36 (proton/amino acid symporter), member 1 (SLC36A1) | NM_078483 | 0.00748 | -1.91 | -1.30 | -1.23 |
| SLC31A2 | solute carrier family 31 (copper transporters), member 2 (SLC31A2) | NM_001860 | 0.04663 | -1.18 | -1.91 | -1.09 |
| XPO6 | exportin 6 (XPO6) | NM_015171 | 0.01063 | -1.44 | -1.19 | -2.42 |
| SEC62 | translocation protein 1 (TLOC1) | NM_003262 | 0.03384 | -1.33 | -1.09 | -1.15 |
| UNC50 | unc-50 homolog (C. elegans) (UNC50) | NM_014044 | 0.01600 | -1.07 | -1.02 | -1.94 |
| RINT1 | RAD50 interactor 1 (RINT1) | NM_021930 | 0.00079 | -1.45 | -1.17 | -1.84 |
| RAB4B | RAB4B, member RAS oncogene family (RAB4B) | NM_016154 | 0.04405 | -1.08 | -1.06 | -1.95 |
| SLC35B3 | solute carrier family 35, member B3 (SLC35B3) | NM_015948 | 0.01108 | -1.28 | -1.16 | -1.15 |
| GJA1 | gap junction protein, alpha 1, 43kDa (GJA1) | NM_000165 | 0.02539 | -1.49 | -1.31 | -1.49 |
| NRAS | neuroblastoma RAS viral (v-ras) oncogene homolog (NRAS) | NM_002524 | 0.00181 | -1.48 | -1.16 | -1.39 |
| YIPF5 | Yip1 domain family, member 5 (YIPF5), transcript variant 2 | NM_030799 | 0.04017 | -1.48 | -1.10 | -1.43 |
| DIRC2 | disrupted in renal carcinoma 2 (DIRC2) | NM_032839 | 0.04106 | -1.39 | -1.24 | -1.11 |
| SNX10 | sorting nexin 10 (SNX10) | NM_013322 | 0.01221 | -1.92 | -2.11 | -1.41 |
| CENPE | centromere protein E, 312kDa (CENPE) | NM_001813 | 0.00626 | -2.34 | -1.66 | -1.34 |
| YWHAB | tyrosine 3-monooxygenase/tryptophan 5-monooxygenase activation protein, beta polypeptide (YWHAB), transcript variant 1 | NM_003404 | 0.03741 | -1.30 | -1.08 | -1.17 |
| SLC39A6 | solute carrier family 39 (zinc transporter), member 6 (SLC39A6) | NM_012319 | 0.00974 | -1.46 | -1.36 | -1.65 |
| KIF11 | kinesin family member 11 (KIF11) | NM_004523 | 0.00998 | -1.80 | -1.80 | -1.42 |
| SFXN1 | sideroflexin 1 (SFXN1) | NM_022754 | 0.01696 | -1.53 | -1.74 | -1.08 |
| KIF2C | kinesin family member 2C (KIF2C) | NM_006845 | 0.04842 | -1.38 | -1.64 | -1.27 |
| TIMM13 | translocase of inner mitochondrial membrane 13 homolog (yeast) (TIMM13), nuclear gene encoding mitochondrial protein | NM_012458 | 0.03834 | -1.43 | -1.06 | -1.80 |
| RAB3IP | RAB3A interacting protein (rabin3) (RAB3IP), transcript variant alpha 2 | NM_175623 | 0.00479 | -1.84 | -1.31 | -1.34 |
| CLTC | clathrin, heavy chain (Hc) (CLTC) | NM_004859 | 0.01924 | -1.26 | -1.06 | -1.44 |
| VTI1B | vesicle transport through interaction with t-SNAREs homolog 1B (yeast) (VTI1B) | NM_006370 | 0.03066 | -1.39 | -1.11 | -1.16 |
| WAS | Wiskott-Aldrich syndrome (eczema-thrombocytopenia) (WAS) | NM_000377 | 0.01074 | -1.92 | -1.33 | -1.89 |
| RAB18 | RAB18, member RAS oncogene family (RAB18) | NM_021252 | 0.00307 | -1.43 | -1.07 | -1.28 |
| PDIA3 | protein disulfide isomerase family A, member 3 (PDIA3) | NM_005313 | 0.01053 | -1.40 | -1.10 | -1.45 |
| DOPEY2 | dopey family member 2 (DOPEY2) | NM_005128 | 0.03532 | -1.35 | -1.24 | -1.36 |
| NUP205 | nucleoporin 205kDa (NUP205) | NM_015135 | 0.03122 | -1.09 | -1.07 | -1.35 |
| SEC23A | Sec23 homolog A (S. cerevisiae) (SEC23A) | NM_006364 | 0.00493 | -1.61 | -1.05 | -1.99 |
| ABCB10 | ATP-binding cassette, sub-family B (MDR/TAP), member 10 (ABCB10), nuclear gene encoding mitochondrial protein | NM_012089 | 0.00860 | -1.36 | -1.18 | -1.18 |
| AP2A2 | adaptor-related protein complex 2, alpha 2 subunit (AP2A2) | NM_012305 | 0.00507 | -1.21 | -1.03 | -1.35 |
| CKLF | chemokine-like factor (CKLF), transcript variant 1 | NM_016951 | 0.00042 | -1.13 | -1.06 | -12.48 |
| ARL6IP1 | ADP-ribosylation factor-like 6 interacting protein 1 (ARL6IP1) | NM_015161 | 0.03225 | -1.21 | -1.32 | -1.44 |
| AGFG1 | HIV-1 Rev binding protein (HRB) | NM_004504 | 0.01958 | -1.09 | -1.12 | -1.36 |
| LIN7C | lin-7 homolog C (C. elegans) (LIN7C) | NM_018362 | 0.00063 | -1.43 | -1.01 | -15.93 |
| GNAI3 | guanine nucleotide binding protein (G protein), alpha inhibiting activity polypeptide 3 (GNAI3) | NM_006496 | 0.02941 | -1.19 | -1.08 | -1.39 |
| YWHAG | tyrosine 3-monooxygenase/tryptophan 5-monooxygenase activation protein, gamma polypeptide (YWHAG) | NM_012479 | 0.03047 | -1.17 | -1.09 | -1.09 |
| MRS2 | MRS2-like, magnesium homeostasis factor (S. cerevisiae) (MRS2L) | NM_020662 | 0.01196 | -1.56 | -1.28 | -1.12 |
| SLCO4C1 | solute carrier organic anion transporter family, member 4C1 (SLCO4C1) | NM_180991 | 0.02693 | -1.07 | -2.05 | -1.33 |
| SLC35F5 | solute carrier family 35, member F5 (SLC35F5) | NM_025181 | 0.00703 | -1.39 | -1.23 | -1.10 |
| CACNB1 | calcium channel, voltage-dependent, beta 1 subunit (CACNB1), transcript variant 3 | NM_199248 | 0.01793 | -1.27 | -1.13 | -1.71 |
| MYO5A | myosin VA (heavy chain 12, myoxin) (MYO5A) | NM_000259 | 0.01768 | -1.57 | -1.34 | -1.35 |
| G3BP1 | GTPase activating protein (SH3 domain) binding protein 1 (G3BP1), transcript variant 1 | NM_005754 | 0.03724 | -1.14 | -1.07 | -1.42 |
| RAB4A | RAB4A, member RAS oncogene family (RAB4A) | NM_004578 | 0.00019 | -1.53 | -1.00 | -5.51 |
| FNBP1L | formin binding protein 1-like (FNBP1L), transcript variant 2 | NM_017737 | 0.03091 | -1.16 | -1.53 | -1.49 |
| SLC36A4 | solute carrier family 36 (proton/amino acid symporter), member 4 (SLC36A4) | NM_152313 | 0.01046 | -1.13 | -1.58 | -2.22 |
| RACGAP1 | Rac GTPase activating protein 1 (RACGAP1) | NM_013277 | 0.03015 | -1.49 | -1.54 | -1.15 |
| SLC39A8 | solute carrier family 39 (zinc transporter), member 8 (SLC39A8) | NM_022154 | 0.04151 | -1.37 | -3.61 | -1.39 |
| TMCO3 | transmembrane and coiled-coil domains 3 (TMCO3) | NM_017905 | 0.00620 | 1.07 | -1.57 | -4.62 |
| RAB7L1 | RAB7, member RAS oncogene family-like 1 (RAB7L1) | NM_003929 | 0.02260 | 1.16 | -2.25 | -1.04 |
| NACA2 | nascent-polypeptide-associated complex alpha polypeptide-like (NACAL) | NM_199290 | 0.03744 | 1.02 | -1.02 | -2.58 |
| SRP72 | signal recognition particle 72kDa (SRP72) | NM_006947 | 0.02668 | -1.25 | 1.01 | -1.79 |
| TUBA1B | tubulin, alpha 1b (TUBA1B) | NM_006082 | 0.04541 | -1.43 | 1.03 | -1.57 |
| SNX14 | sorting nexin 14 (SNX14), transcript variant 1 | NM_153816 | 0.04302 | -1.17 | 1.04 | -1.66 |
| NUP88 | nucleoporin 88kDa (NUP88) | NM_002532 | 0.02830 | -1.21 | 1.02 | -1.53 |
| TIMM17A | translocase of inner mitochondrial membrane 17 homolog A (yeast) (TIMM17A) | NM_006335 | 0.03568 | -1.20 | 1.05 | -1.66 |
| SEC22C | SEC22 vesicle trafficking protein homolog C (S. cerevisiae) (SEC22C), transcript variant 1 | NM_032970 | 0.01435 | -1.52 | 1.05 | -1.53 |
| NUP155 | nucleoporin 155kDa (NUP155), transcript variant 1 | NM_153485 | 0.04460 | -1.13 | 1.10 | -1.47 |
| SEC24D | SEC24 related gene family, member D (S. cerevisiae) (SEC24D) | NM_014822 | 0.03516 | -1.56 | 1.08 | -1.59 |
| TRPC4AP | transient receptor potential cation channel, subfamily C, member 4 associated protein (TRPC4AP), transcript variant 1 | NM_015638 | 0.00010 | -1.34 | 1.07 | -9.45 |
| TIMM23 | translocase of inner mitochondrial membrane 23 homolog (yeast) (TIMM23) | NM_006327 | 0.03342 | -1.41 | 1.02 | -1.32 |
| SEC61B | Sec61 beta subunit (SEC61B) | NM_006808 | 0.02049 | -1.24 | 1.05 | -1.80 |
| KCND1 | potassium voltage-gated channel, Shal-related subfamily, member 1 (KCND1) | NM_004979 | 0.03143 | 1.77 | 1.32 | -1.34 |
| SEC14L3 | SEC14-like 3 (S. cerevisiae) (SEC14L3) | NM_174975 | 0.02553 | 3.04 | 1.79 | -1.55 |
| KCNK9 | potassium channel, subfamily K, member 9 (KCNK9) | NM_016601 | 0.02450 | 2.21 | 1.30 | -1.24 |
| CNGB1 | cyclic nucleotide gated channel beta 1 (CNGB1) | NM_001297 | 0.04435 | 1.81 | 1.68 | -1.26 |
| SLC41A2 | solute carrier family 41, member 2 (SLC41A2) | NM_032148 | 0.04211 | 2.32 | 1.24 | -1.05 |
| RIMS4 | regulating synaptic membrane exocytosis 4 (RIMS4) | NM_182970 | 0.04113 | 1.72 | 2.97 | -1.02 |
| KCNE2 | potassium voltage-gated channel, Isk-related family, member 2 (KCNE2) | NM_172201 | 0.00070 | 1.97 | 3.11 | -1.17 |
| ELMOD1 | ELMO/CED-12 domain containing 1 (ELMOD1) | NM_018712 | 0.01143 | -1.74 | -25.93 | 2.05 |
| KCNK6 | potassium channel, subfamily K, member 6 (KCNK6) | NM_004823 | 0.02003 | 1.58 | -1.04 | 1.53 |
| TRPM4 | transient receptor potential cation channel, subfamily M, member 4 (TRPM4) | NM_017636 | 0.01293 | 1.10 | -1.03 | 2.23 |
| ABCC5 | ATP-binding cassette, sub-family C (CFTR/MRP), member 5 (ABCC5), transcript variant 1 | NM_005688 | 0.02846 | 1.61 | -1.17 | 1.60 |
| CYTH3 | pleckstrin homology, Sec7 and coiled-coil domains 3 (PSCD3) | NM_004227 | 0.01517 | 1.20 | -1.01 | 1.38 |
| VPS16 | vacuolar protein sorting 16 homolog (S. cerevisiae) (VPS16), transcript variant 2 | NM_080414 | 0.00375 | 1.27 | -1.03 | 1.56 |
| SLC6A1 | solute carrier family 6 (neurotransmitter transporter, GABA), member 1 (SLC6A1) | NM_003042 | 0.04881 | 1.75 | -1.06 | 2.17 |
| CNNM2 | cyclin M2 (CNNM2), transcript variant 1 | NM_017649 | 0.00019 | 3.14 | -1.01 | 1.79 |
| SYTL4 | synaptotagmin-like 4 (granuphilin-a) (SYTL4) | NM_080737 | 0.00667 | 1.92 | -1.02 | 1.17 |
| SLC2A11 | solute carrier family 2 (facilitated glucose transporter), member 11 (SLC2A11), transcript variant 1 | NM_030807 | 0.02728 | 1.29 | -1.01 | 3.49 |
| UNC13B | unc-13 homolog B (C. elegans) (UNC13B) | NM_006377 | 0.01843 | 1.37 | -1.01 | 1.33 |
| SLC25A23 | solute carrier family 25 (mitochondrial carrier; phosphate carrier), member 23 (SLC25A23) | NM_024103 | 0.01926 | -1.01 | 1.14 | 2.09 |
| P2RX5 | purinergic receptor P2X, ligand-gated ion channel, 5 (P2RX5), transcript variant 1 | NM_002561 | 0.03147 | -1.51 | 1.78 | 3.10 |
| SLC26A9 | solute carrier family 26, member 9 (SLC26A9), transcript variant 1 | NM_052934 | 0.04210 | 3.09 | 2.14 | 1.78 |
| KCNB1 | potassium voltage-gated channel, Shab-related subfamily, member 1 (KCNB1) | NM_004975 | 0.03414 | 1.38 | 1.77 | 1.81 |
| SV2B | synaptic vesicle glycoprotein 2B (SV2B) | NM_014848 | 0.00683 | 2.05 | 1.50 | 1.30 |
| ABCC6 | ATP-binding cassette, sub-family C (CFTR/MRP), member 6 (ABCC6), transcript variant 1 | NM_001171 | 0.01170 | 2.08 | 1.41 | 2.28 |
| CDC37 | cell division cycle 37 homolog (S. cerevisiae) (CDC37) | NM_007065 | 0.00649 | 1.01 | 1.09 | 1.47 |
| RALBP1 | ralA binding protein 1 (RALBP1) | NM_006788 | 0.00315 | 1.46 | 1.35 | 1.31 |
| ARFGAP1 | ADP-ribosylation factor GTPase activating protein 1 (ARFGAP1), transcript variant 2 | NM_175609 | 0.03603 | 1.08 | 1.07 | 1.59 |
| C19orf28 | chromosome 19 open reading frame 28 (C19orf28), transcript variant 3 | NM_174983 | 0.00707 | 1.09 | 1.19 | 1.89 |
| SEC16B | leucine zipper transcription regulator 2 (LZTR2) | NM_033127 | 0.00283 | 1.49 | 1.30 | 2.84 |
| SCN3B | sodium channel, voltage-gated, type III, beta (SCN3B), transcript variant 1 | NM_018400 | 0.00199 | 2.73 | 1.57 | 1.27 |
| DYNC1H1 | dynein, cytoplasmic 1, heavy chain 1 (DYNC1H1) | NM_001376 | 0.01157 | 1.25 | 1.12 | 1.52 |
| GRIN3A | glutamate receptor, ionotropic, N-methyl-D-aspartate 3A (GRIN3A) | NM_133445 | 0.02003 | 1.99 | 2.04 | 1.52 |
| PEX16 | peroxisomal biogenesis factor 16 (PEX16), transcript variant 1 | NM_004813 | 0.00287 | 1.18 | 1.22 | 1.90 |
| EXOC3 | exocyst complex component 3 (EXOC3) | NM_007277 | 0.00015 | 1.51 | 1.39 | 1.63 |
| GGA3 | golgi associated, gamma adaptin ear containing, ARF binding protein 3 (GGA3), transcript variant long | NM_138619 | 0.04030 | 1.14 | 1.08 | 1.39 |
| TRIP10 | thyroid hormone receptor interactor 10 (TRIP10) | NM_004240 | 0.00144 | 1.37 | 1.23 | 1.31 |
| RHOT2 | ras homolog gene family, member T2 (RHOT2) | NM_138769 | 0.00002 | 1.21 | 1.21 | 1.57 |
| CACNG2 | calcium channel, voltage-dependent, gamma subunit 2 (CACNG2) | NM_006078 | 0.03868 | 1.60 | 1.51 | 1.08 |
| KCNQ2 | potassium voltage-gated channel, KQT-like subfamily, member 2 (KCNQ2), transcript variant 5 | NM_172109 | 0.03774 | 1.10 | 1.23 | 1.51 |
| SLC25A42 | solute carrier family 25, member 42 (SLC25A42) | NM_178526 | 0.00790 | 1.33 | 1.63 | 2.57 |
| GRID2 | glutamate receptor, ionotropic, delta 2 (GRID2) | NM_001510 | 0.01032 | 1.70 | 1.63 | 1.06 |
| TSC2 | tuberous sclerosis 2 (TSC2), transcript variant 1 | NM_000548 | 0.00071 | 1.14 | 1.20 | 1.74 |
| GOLGA3 | golgi autoantigen, golgin subfamily a, 3 (GOLGA3) | NM_005895 | 0.03059 | 1.22 | 1.42 | 1.13 |
| TRPM5 | transient receptor potential cation channel, subfamily M, member 5 (TRPM5) | NM_014555 | 0.03822 | 1.35 | 1.20 | 1.79 |
| C11orf2 | chromosome 11 open reading frame2 (C11orf2) | NM_013265 | 0.00010 | 1.16 | 1.14 | 1.96 |
| CXCL16 | chemokine (C-X-C motif) ligand 16 (CXCL16) | NM_022059 | 0.00123 | 2.31 | 1.38 | 1.74 |
| SLC25A28 | solute carrier family 25, member 28 (SLC25A28) | NM_031212 | 0.00085 | 1.11 | 1.16 | 1.64 |
| 05-Sep | septin 5 (SEPT5) | NM_002688 | 0.04681 | 1.06 | 1.28 | 1.39 |
| SLC24A2 | solute carrier family 24 (sodium/potassium/calcium exchanger), member 2 (SLC24A2) | NM_020344 | 0.01152 | 1.49 | 1.62 | 1.07 |
| SLC5A11 | solute carrier family 5 (sodium/glucose cotransporter), member 11 (SLC5A11) | NM_052944 | 0.00042 | 2.32 | 1.52 | 2.00 |
| SLC16A12 | solute carrier family 16, member 12 (monocarboxylic acid transporter 12) (SLC16A12) | NM_213606 | 0.04959 | 1.82 | 1.22 | 1.07 |
| ACCN3 | amiloride-sensitive cation channel 3 (ACCN3), transcript variant 3 | NM_020322 | 0.00216 | 1.41 | 1.87 | 2.03 |
| GJB1 | gap junction protein, beta 1, 32kDa (GJB1) | NM_000166 | 0.00391 | 1.57 | 1.26 | 1.34 |
| SPNS1 | spinster (SPIN1) | NM_032038 | 0.00837 | 1.17 | 1.02 | 1.73 |
| SEC24C | SEC24 related gene family, member C (S. cerevisiae) (SEC24C), transcript variant 1 | NM_004922 | 0.01904 | 1.02 | 1.08 | 1.29 |
| FAM3B | family with sequence similarity 3, member B (FAM3B), transcript variant 1 | NM_058186 | 0.02486 | 1.96 | 1.57 | 1.16 |
| P2RX1 | purinergic receptor P2X, ligand-gated ion channel, 1 (P2RX1) | NM_002558 | 0.01479 | 1.49 | 1.76 | 1.51 |
| DNM2 | dynamin 2 (DNM2), transcript variant 1 | NM_001005360 | 0.01342 | 1.29 | 1.10 | 1.36 |
| LIN7B | lin-7 homolog B (C. elegans) (LIN7B) | NM_022165 | 0.02022 | 1.22 | 1.47 | 1.07 |
| FNBP1 | formin binding protein 1 (FNBP1) | NM_015033 | 0.03536 | 1.16 | 1.24 | 1.28 |
| SCG2 | secretogranin II (chromogranin C) (SCG2) | NM_003469 | 0.01775 | 2.10 | 1.23 | 1.40 |
| VPS41 | vacuolar protein sorting 41 homolog (S. cerevisiae) (VPS41), transcript variant 2 | NM_080631 | 0.03720 | 1.15 | 1.02 | 1.51 |
| KCTD15 | potassium channel tetramerisation domain containing 15 (KCTD15) | NM_024076 | 0.02580 | 1.30 | 1.29 | 1.40 |
| AP4M1 | adaptor-related protein complex 4, mu 1 subunit (AP4M1) | NM_004722 | 0.01453 | 1.19 | 1.42 | 1.17 |
| SLC12A9 | solute carrier family 12 (potassium/chloride transporters), member 9 (SLC12A9) | NM_020246 | 0.00001 | 1.37 | 1.31 | 1.75 |
| RILP | Rab interacting lysosomal protein (RILP) | NM_031430 | 0.00486 | 1.52 | 1.52 | 1.71 |
| SLC38A3 | solute carrier family 38, member 3 (SLC38A3) | NM_006841 | 0.00582 | 1.36 | 2.10 | 1.73 |
| HGS | hepatocyte growth factor-regulated tyrosine kinase substrate (HGS) | NM_004712 | 0.00194 | 1.18 | 1.07 | 1.81 |
| MYH6 | myosin, heavy chain 6, cardiac muscle, alpha (cardiomyopathy, hypertrophic 1) (MYH6) | NM_002471 | 0.03264 | 2.24 | 1.59 | 1.36 |
| GLP1R | glucagon-like peptide 1 receptor (GLP1R) | NM_002062 | 0.04596 | 1.84 | 1.24 | 1.37 |
| DENND1A | DENN/MADD domain containing 1A (DENND1A), transcript variant 1 | NM_020946 | 0.00567 | 1.30 | 1.12 | 1.41 |
| VPS11 | vacuolar protein sorting 11 homolog (S. cerevisiae) (VPS11) | NM_021729 | 0.04091 | 1.11 | 1.09 | 1.24 |
| NM_005704 | protein tyrosine phosphatase, receptor type, U (PTPRU), transcript variant 3 | NM_005704 | 0.0186841 | 1.9626 | -1.05 | 2.2312 |
| SH3GLB2 | SH3-domain GRB2-like endophilin B2 (SH3GLB2) | NM_020145 | 0.0026808 | 1.2347 | 1.3143 | 1.708 |
| NM_000356 | Treacher Collins-Franceschetti syndrome 1 (TCOF1), transcript variant 2 | NM_000356 | 0.0016162 | 1.4952 | 1.3849 | 1.4567 |
| **Regulation of Molecular Functions** | |  |  |  |  |  |
| GABBR1 | gamma-aminobutyric acid (GABA) B receptor, 1 (GABBR1), transcript variant 1 | NM_001470 | 0.00344 | 1.20 | 1.39 | 2.41 |
| ARHGAP6 | Rho GTPase activating protein 6 (ARHGAP6), transcript variant 2 | NM_001174 | 0.00976 | 1.18 | 1.27 | 1.54 |
| DGKZ | diacylglycerol kinase, zeta 104kDa (DGKZ), transcript variant 2 | NM_003646 | 0.00695 | 1.11 | 1.15 | 1.63 |
| DOCK7 | dedicator of cytokinesis 7 (DOCK7) | NM_033407 | 0.01800 | 1.17 | 1.17 | -9.74 |
| HBXIP | hepatitis B virus x interacting protein (HBXIP) | NM_006402 | 0.01076 | -1.34 | 1.05 | -1.80 |
| SMAP1 | stromal membrane-associated protein 1 (SMAP1), transcript variant 2 | NM_021940 | 0.04998 | -1.46 | -1.32 | -1.65 |
| SGSM3 | RUN and TBC1 domain containing 3 (RUTBC3) | NM_015705 | 0.03274 | 1.02 | 1.13 | 1.75 |
| SIGIRR | single immunoglobulin and toll-interleukin 1 receptor (TIR) domain (SIGIRR) | NM_021805 | 0.02663 | 1.15 | -1.17 | 1.98 |
| TBC1D8B | TBC1 domain family, member 8B (with GRAM domain) (TBC1D8B), transcript variant 1 | NM_017752 | 0.00452 | -1.73 | -1.02 | -2.11 |
| TNNC1 | troponin C type 1 (slow) (TNNC1) | NM_003280 | 0.02856 | 1.66 | 1.78 | 1.26 |
| CALCA | calcitonin/calcitonin-related polypeptide, alpha (CALCA), transcript variant 1 | NM_001741 | 0.02911 | 2.41 | 1.28 | -1.03 |
| CAP2 | CAP, adenylate cyclase-associated protein, 2 (yeast) (CAP2) | NM_006366 | 0.03196 | 1.24 | 1.44 | 1.38 |
| SPRED1 | sprouty-related, EVH1 domain containing 1 (SPRED1) | NM_152594 | 0.00597 | -1.30 | -1.19 | -1.52 |
| ADAP2 | centaurin, alpha 2 (CENTA2) | NM_018404 | 0.01575 | 1.87 | 1.22 | 1.70 |
| GALR3 | galanin receptor 3 (GALR3) | NM_003614 | 0.04232 | 1.21 | 1.30 | 2.55 |
| SMO | smoothened homolog (Drosophila) (SMO) | NM_005631 | 0.02807 | 1.49 | 1.31 | 1.22 |
| CALM3 | calmodulin 3 (phosphorylase kinase, delta) (CALM3) | NM_005184 | 0.04605 | -1.10 | 1.12 | 2.26 |
| CKS2 | CDC28 protein kinase regulatory subunit 2 (CKS2) | NM_001827 | 0.01045 | -2.12 | -1.53 | -1.55 |
| ARHGAP1 | Rho GTPase activating protein 1 (ARHGAP1) | NM_004308 | 0.03831 | 1.44 | 1.39 | -1.03 |
| AIDA | chromosome 1 open reading frame 80 (C1orf80) | NM_022831 | 0.04437 | -1.29 | 1.01 | -1.10 |
| DLC1 | deleted in liver cancer 1 (DLC1), transcript variant 3 | NM_024767 | 0.02419 | 1.50 | 1.26 | -1.00 |
| ACAP3 | centaurin, beta 5 (CENTB5) | NM_030649 | 0.04630 | 1.06 | 1.04 | 2.43 |
| TBC1D14 | TBC1 domain family, member 14 (TBC1D14) | NM_020773 | 0.03100 | -1.22 | 1.01 | -1.72 |
| TBC1D22B | TBC1 domain family, member 22B (TBC1D22B) | NM_017772 | 0.00002 | -1.47 | 1.09 | -5.58 |
| TBC1D5 | TBC1 domain family, member 5 (TBC1D5) | NM_014744 | 0.01156 | 1.25 | 1.03 | 1.83 |
| TBC1D10A | TBC1 domain family, member 10A (TBC1D10A) | NM_031937 | 0.00682 | 1.34 | 1.20 | 1.92 |
| TBC1D13 | TBC1 domain family, member 13 (TBC1D13) | NM_018201 | 0.03626 | 1.32 | 1.34 | 1.37 |
| NARFL | nuclear prelamin A recognition factor-like (NARFL) | NM_022493 | 0.00691 | 1.00 | 1.24 | 1.57 |
| **Biological Adhesion** | |  |  |  |  |  |
| COL27A1 | collagen, type XXVII, alpha 1 (COL27A1) | NM_032888 | 0.00066 | 1.50 | 1.17 | 2.52 |
| PERP | PERP, TP53 apoptosis effector (PERP) | NM_022121 | 0.02754 | -1.50 | -2.61 | -2.22 |
| HSPB11 | chromosome 1 open reading frame 41 (C1orf41) | NM_016126 | 0.00007 | -1.46 | 1.05 | -8.48 |
| PCDHGB1 | protocadherin gamma subfamily B, 1 (PCDHGB1), transcript variant 2 | NM_032095 | 0.00704 | 1.35 | 1.72 | 1.78 |
| ITGBL1 | integrin, beta-like 1 (with EGF-like repeat domains) (ITGBL1) | NM_004791 | 0.02861 | -2.56 | -3.00 | -1.24 |
| ITGA10 | integrin, alpha 10 (ITGA10) | NM_003637 | 0.02458 | 1.29 | 1.60 | 1.59 |
| CDH23 | cadherin-like 23 (CDH23), transcript variant 1 | NM_022124 | 0.04614 | 1.47 | 1.18 | 1.53 |
| SDK2 | sidekick homolog 2 (chicken) (SDK2) | NM_019064 | 0.04453 | 1.66 | 1.31 | -1.12 |
| PCDH7 | protocadherin 7 (PCDH7), transcript variant a | NM_002589 | 0.03870 | -1.58 | -1.43 | -1.30 |
| CLSTN1 | calsyntenin 1 (CLSTN1), transcript variant 1 | NM_001009566 | 0.02955 | 1.30 | 1.60 | 1.13 |
| BCAR1 | breast cancer anti-estrogen resistance 1 (BCAR1) | NM_014567 | 0.04939 | 1.01 | -1.14 | 1.83 |
| VCAM1 | vascular cell adhesion molecule 1 (VCAM1), transcript variant 1 | NM_001078 | 0.00496 | -1.69 | -1.58 | -1.71 |
| PCDHGB2 | protocadherin gamma subfamily B, 2 (PCDHGB2), transcript variant 2 | NM_032096 | 0.02405 | 2.17 | 1.43 | -1.40 |
| VCAN | versican (VCAN) | NM_004385 | 0.01441 | -1.65 | 1.00 | -2.02 |
| LAMB2 | laminin, beta 2 (laminin S) (LAMB2) | NM_002292 | 0.03209 | 1.00 | 1.22 | 1.77 |
| MLLT4 | myeloid/lymphoid or mixed-lineage leukemia (trithorax homolog, Drosophila); translocated to, 4 (MLLT4), transcript variant 3 | NM_005936 | 0.02389 | 1.52 | 1.33 | -1.15 |
| CD9 | CD9 molecule (CD9) | NM_001769 | 0.04931 | -1.76 | 1.17 | -2.01 |
| DLG5 | discs, large homolog 5 (Drosophila) (DLG5) | NM_004747 | 0.01366 | 1.73 | 1.08 | 1.35 |
| SSPN | sarcospan (Kras oncogene-associated gene) (SSPN) | NM_005086 | 0.01552 | -1.55 | 1.03 | -1.74 |
| NRP1 | neuropilin 1 (NRP1), transcript variant 1 | NM_003873 | 0.03425 | -1.11 | -1.11 | -1.62 |
| FZD6 | frizzled homolog 6 (Drosophila) (FZD6) | NM_003506 | 0.00720 | 1.09 | -1.68 | -1.72 |
| GPR98 | G protein-coupled receptor 98 (GPR98), transcript variant 1 | NM_032119 | 0.02475 | -1.11 | -3.16 | -3.92 |
| MSLN | mesothelin (MSLN), transcript variant 1 | NM_005823 | 0.04642 | 1.19 | -1.22 | 2.87 |
| SYMPK | symplekin (SYMPK) | NM_004819 | 0.01259 | 1.12 | 1.10 | 1.50 |
| MCAM | melanoma cell adhesion molecule (MCAM) | NM_006500 | 0.00826 | 1.29 | 1.94 | 1.30 |
| COL8A2 | collagen, type VIII, alpha 2 (COL8A2) | NM_005202 | 0.02913 | -2.78 | -1.11 | -2.33 |
| NM_001003722 | GLE1 RNA export mediator-like (yeast) (GLE1L), transcript variant 1 | NM_001003722 | 0.0018716 | 1.6117 | 1.1237 | 1.4033 |
| **Developmental Process** | |  |  |  |  |  |
| SERPINF1 | serpin peptidase inhibitor, clade F (alpha-2 antiplasmin, pigment epithelium derived factor), member 1 (SERPINF1) | NM_002615 | 0.04328 | -1.45 | -1.69 | 1.35 |
| PAX8 | paired box gene 8 (PAX8), transcript variant PAX8A | NM_003466 | 0.03554 | 2.00 | 1.17 | -1.27 |
| DFNB31 | deafness, autosomal recessive 31 (DFNB31) | NM_015404 | 0.00900 | 1.26 | 1.38 | 1.21 |
| EIF2B5 | eukaryotic translation initiation factor 2B, subunit 5 epsilon, 82kDa (EIF2B5) | NM_003907 | 0.02008 | 1.08 | 1.15 | 1.24 |
| POGZ | pogo transposable element with ZNF domain (POGZ), transcript variant 1 | NM_015100 | 0.01172 | 1.19 | 1.25 | 1.89 |
| MOV10L1 | Mov10l1, Moloney leukemia virus 10-like 1, homolog (mouse) (MOV10L1) | NM_018995 | 0.01546 | -1.32 | -2.04 | -1.77 |
| PHLPP | PH domain and leucine rich repeat protein phosphatase (PHLPP) | NM_194449 | 0.01001 | 1.40 | 1.25 | 1.26 |
| SPRR3 | small proline-rich protein 3 (SPRR3) | NM_005416 | 0.01351 | 2.57 | 2.68 | 1.37 |
| STAT3 | signal transducer and activator of transcription 3 (acute-phase response factor) (STAT3), transcript variant 3 | NM_213662 | 0.03981 | 1.22 | 1.37 | 1.77 |
| RBM45 | developmentally regulated RNA-binding protein 1 (DRB1) | NM_152945 | 0.01600 | -1.28 | -1.06 | -1.51 |
| PHC2 | polyhomeotic homolog 2 (Drosophila) (PHC2), transcript variant 1 | NM_198040 | 0.02723 | 1.21 | 1.20 | 1.14 |
| PTH1R | parathyroid hormone receptor 1 (PTHR1) | NM_000316 | 0.01759 | 1.45 | 1.17 | 1.24 |
| MTSS1L | actin-bundling protein with BAIAP2 homology (ABBA-1) | NM_138383 | 0.02816 | 1.59 | 1.44 | 1.56 |
| TTC8 | tetratricopeptide repeat domain 8 (TTC8), transcript variant 1 | NM_144596 | 0.00870 | -1.46 | 1.10 | -1.76 |
| EFNB3 | ephrin-B3 (EFNB3) | NM_001406 | 0.01747 | 1.61 | 1.61 | 1.04 |
| NPAS2 | neuronal PAS domain protein 2 (NPAS2) | NM_002518 | 0.01191 | 1.58 | 1.07 | 1.11 |
| PPHLN1 | periphilin 1 (PPHLN1), transcript variant 2 | NM_201515 | 0.03330 | -1.23 | 1.03 | -2.16 |
| AAMP | angio-associated, migratory cell protein (AAMP) | NM_001087 | 0.00003 | -1.56 | -1.09 | -10.17 |
| EVL | Enah/Vasp-like (EVL) | NM_016337 | 0.02016 | 1.31 | 1.18 | 1.50 |
| PLXNB1 | plexin B1 (PLXNB1) | NM_002673 | 0.01815 | -1.05 | 1.35 | 2.06 |
| CXCL1 | chemokine (C-X-C motif) ligand 1 (melanoma growth stimulating activity, alpha) (CXCL1) | NM_001511 | 0.00696 | 1.06 | -3.39 | -4.61 |
| MEST | mesoderm specific transcript homolog (mouse) (MEST), transcript variant 1 | NM_002402 | 0.01177 | -1.55 | -1.28 | -1.05 |
| GYLTL1B | glycosyltransferase-like 1B (GYLTL1B) | NM_152312 | 0.00094 | 3.14 | 1.17 | 3.20 |
| GAS2 | growth arrest-specific 2 (GAS2), transcript variant 1 | NM_005256 | 0.03903 | -2.21 | -1.15 | -1.41 |
| ATOH8 | atonal homolog 8 (Drosophila) (ATOH8) | NM_032827 | 0.01557 | 1.43 | 1.43 | 1.71 |
| STIL | SCL/TAL1 interrupting locus (STIL), transcript variant 2 | NM_003035 | 0.01412 | -1.36 | -1.31 | -1.47 |
| FARP2 | FERM, RhoGEF and pleckstrin domain protein 2 (FARP2) | NM_014808 | 0.04624 | 1.65 | -1.08 | 1.57 |
| NTF3 | neurotrophin 3 (NTF3) | NM_002527 | 0.00995 | 1.15 | 2.24 | -1.06 |
| TCAP | titin-cap (telethonin) (TCAP) | NM_003673 | 0.03185 | 1.12 | 1.20 | 2.05 |
| GPC4 | glypican 4 (GPC4) | NM_001448 | 0.01549 | -1.34 | -1.13 | -1.29 |
| SLIT3 | slit homolog 3 (Drosophila) (SLIT3) | NM_003062 | 0.04962 | -1.62 | -1.24 | -1.75 |
| EN1 | engrailed homeobox 1 (EN1) | NM_001426 | 0.03473 | 1.38 | 2.21 | 1.02 |
| PBXIP1 | pre-B-cell leukemia homeobox interacting protein 1 (PBXIP1) | NM_020524 | 0.00250 | 1.13 | 1.48 | 1.49 |
| PDCD10 | programmed cell death 10 (PDCD10), transcript variant 1 | NM_007217 | 0.03567 | -1.14 | -1.09 | -1.28 |
| AXIN1 | axin 1 (AXIN1), transcript variant 1 | NM_003502 | 0.00770 | 1.32 | 1.05 | 1.43 |
| NAB2 | NGFI-A binding protein 2 (EGR1 binding protein 2) (NAB2) | NM_005967 | 0.00001 | -1.71 | -1.03 | -4.43 |
| EMD | emerin (Emery-Dreifuss muscular dystrophy) (EMD) | NM_000117 | 0.02067 | 1.15 | 1.10 | 1.20 |
| TCTN3 | chromosome 10 open reading frame 61 (C10orf61), transcript variant 2 | NM_015631 | 0.04343 | 1.15 | 1.37 | 1.09 |
| LCE3E | late cornified envelope 3E (LCE3E) | NM_178435 | 0.00737 | 2.19 | 1.49 | 1.02 |
| RTN4 | reticulon 4 (RTN4), transcript variant 1 | NM_020532 | 0.03957 | -1.30 | -1.14 | -1.41 |
| TBX19 | T-box 19 (TBX19) | NM_005149 | 0.00404 | 1.21 | 1.21 | 1.54 |
| DLX2 | distal-less homeobox 2 (DLX2) | NM_004405 | 0.03938 | 1.88 | 1.02 | 1.22 |
| OSTF1 | osteoclast stimulating factor 1 (OSTF1) | NM_012383 | 0.00804 | -1.64 | -1.18 | -1.80 |
| SNAI1 | snail homolog 1 (Drosophila) (SNAI1) | NM_005985 | 0.00504 | -1.53 | -4.58 | -1.85 |
| SIX1 | sine oculis homeobox homolog 1 (Drosophila) (SIX1) | NM_005982 | 0.04573 | 1.13 | 1.23 | 2.41 |
| RIC8A | resistance to inhibitors of cholinesterase 8 homolog A (C. elegans) (RIC8A) | NM_021932 | 0.04967 | 1.22 | 1.21 | 1.04 |
| TLE1 | transducin-like enhancer of split 1 (E(sp1) homolog, Drosophila) (TLE1) | NM_005077 | 0.04854 | 1.25 | 1.24 | 1.71 |
| MAPT | microtubule-associated protein tau (MAPT), transcript variant 1 | NM_016835 | 0.02192 | 1.02 | 1.55 | 2.00 |
| COL9A2 | collagen, type IX, alpha 2 (COL9A2) | NM_001852 | 0.00460 | 1.56 | 1.15 | 2.28 |
| DVL3 | dishevelled, dsh homolog 3 (Drosophila) (DVL3) | NM_004423 | 0.00853 | 1.53 | 1.22 | 1.40 |
| ODF2 | outer dense fiber of sperm tails 2 (ODF2), transcript variant 1 | NM_002540 | 0.02295 | 1.22 | 1.22 | 1.16 |
| WNT3 | wingless-type MMTV integration site family, member 3 (WNT3) | NM_030753 | 0.02186 | 1.59 | 1.11 | 1.42 |
| PCNT | pericentrin (kendrin) (PCNT) | NM_006031 | 0.01150 | 1.28 | 1.22 | 1.37 |
| OFD1 | oral-facial-digital syndrome 1 (OFD1) | NM_003611 | 0.03408 | 1.20 | 1.07 | 1.46 |
| OLFML3 | olfactomedin-like 3 (OLFML3) | NM_020190 | 0.03547 | -1.97 | -1.26 | -1.24 |
| SHROOM1 | shroom family member 1 (SHROOM1) | NM_133456 | 0.01305 | 1.81 | -1.29 | 1.55 |
| HOXB13 | homeobox B13 (HOXB13) | NM_006361 | 0.00840 | 2.82 | 1.53 | -1.06 |
| SEMA3B | sema domain, immunoglobulin domain (Ig), short basic domain, secreted, (semaphorin) 3B (SEMA3B), transcript variant 1 | NM_004636 | 0.00196 | -1.04 | 1.48 | 1.93 |
| POU4F3 | POU domain, class 4, transcription factor 3 (POU4F3) | NM_002700 | 0.02460 | 3.86 | 1.62 | -1.99 |
| LIMS1 | LIM and senescent cell antigen-like domains 1 (LIMS1) | NM_004987 | 0.02434 | -1.33 | -1.14 | -1.69 |
| FHL1 | four and a half LIM domains 1 (FHL1) | NM_001449 | 0.03805 | -1.05 | 1.36 | 1.58 |
| UNC45A | unc-45 homolog A (C. elegans) (UNC45A), transcript variant 2 | NM_018671 | 0.00008 | 1.09 | 1.25 | 1.42 |
| CIDEB | cell death-inducing DFFA-like effector b (CIDEB) | NM_014430 | 0.03171 | 1.73 | 1.01 | 2.81 |
| ACIN1 | apoptotic chromatin condensation inducer 1 (ACIN1) | NM_014977 | 0.04121 | 1.58 | 1.07 | 1.10 |
| EGFL7 | EGF-like-domain, multiple 7 (EGFL7), transcript variant 2 | NM_201446 | 0.00794 | 1.38 | -1.03 | 2.19 |
| ACP2 | acid phosphatase 2, lysosomal (ACP2) | NM_001610 | 0.02528 | -1.31 | -1.13 | -1.53 |
| HEYL | hairy/enhancer-of-split related with YRPW motif-like (HEYL) | NM_014571 | 0.01960 | 1.20 | 1.22 | 1.21 |
| OTOP1 | otopetrin 1 (OTOP1) | NM_177998 | 0.04308 | 2.13 | 1.48 | -1.16 |
| TSNAXIP1 | translin-associated factor X interacting protein 1 (TSNAXIP1) | NM_018430 | 0.03730 | 1.32 | 1.48 | 1.02 |
| PHC1 | polyhomeotic homolog 1 (Drosophila) (PHC1) | NM_004426 | 0.00436 | 1.23 | 1.29 | 1.73 |
| ECT2 | epithelial cell transforming sequence 2 oncogene (ECT2) | NM_018098 | 0.00083 | -1.74 | -1.29 | -2.97 |
| DNM1L | dynamin 1-like (DNM1L), transcript variant 1 | NM_012062 | 0.01310 | -1.56 | -1.10 | -1.93 |
| S100A4 | S100 calcium binding protein A4 (S100A4), transcript variant 1 | NM_002961 | 0.01836 | -2.66 | -1.05 | -2.01 |
| TNS4 | tensin 4 (TNS4) | NM_032865 | 0.04163 | 2.72 | 1.32 | -1.30 |
| **Other Biological Process** | |  |  |  |  |  |
| DOK1 | docking protein 1, 62kDa (downstream of tyrosine kinase 1) (DOK1) | NM_001381 | 0.03668 | 1.80 | 1.11 | 1.08 |
| APBB1IP | amyloid beta (A4) precursor protein-binding, family B, member 1 interacting protein (APBB1IP) | NM_019043 | 0.03476 | -1.49 | -1.51 | -1.60 |
| GPRC5D | G protein-coupled receptor, family C, group 5, member D (GPRC5D) | NM_018654 | 0.00652 | 1.74 | 1.58 | -1.16 |
| SHOC2 | soc-2 suppressor of clear homolog (C. elegans) (SHOC2) | NM_007373 | 0.04324 | -1.23 | 1.05 | -1.45 |
| COBRA1 | cofactor of BRCA1 (COBRA1) | NM_015456 | 0.03556 | 1.30 | 1.11 | 1.48 |
| PIK3R2 | phosphoinositide-3-kinase, regulatory subunit 2 (p85 beta) (PIK3R2) | NM_005027 | 0.03729 | 1.11 | 1.14 | 1.60 |
| RALB | v-ral simian leukemia viral oncogene homolog B (ras related; GTP binding protein) (RALB) | NM_002881 | 0.04724 | -1.41 | -1.36 | -1.12 |
| RXFP1 | relaxin/insulin-like family peptide receptor 1 (RXFP1) | NM_021634 | 0.00210 | 1.93 | 3.04 | 1.28 |
| PDE1C | phosphodiesterase 1C, calmodulin-dependent 70kDa (PDE1C) | NM_005020 | 0.02690 | 2.67 | 1.60 | -1.15 |
| ZNF207 | zinc finger protein 207 (ZNF207), transcript variant 1 | NM_003457 | 0.02032 | -1.23 | 1.02 | -1.08 |
| RAP1B | RAP1B, member of RAS oncogene family (RAP1B), transcript variant 1 | NM_015646 | 0.04988 | -1.19 | 1.10 | -1.59 |
| DEF8 | hypothetical protein FLJ20186 (FLJ20186), transcript variant 1 | NM_207514 | 0.00034 | 1.27 | 1.16 | 2.04 |
| CDC123 | cell division cycle 123 homolog (S. cerevisiae) (CDC123) | NM_006023 | 0.00001 | -1.58 | 1.01 | -5.01 |
| FLCN | folliculin (FLCN), transcript variant 1 | NM_144997 | 0.04126 | 1.40 | -1.01 | 1.23 |
| TXNDC11 | thioredoxin domain containing 11 (TXNDC11) | NM_015914 | 0.00004 | -1.53 | -1.01 | -12.38 |
| PDE2A | phosphodiesterase 2A, cGMP-stimulated (PDE2A) | NM_002599 | 0.02142 | 2.46 | 2.08 | 1.57 |
| FRS3 | fibroblast growth factor receptor substrate 3 (FRS3) | NM_006653 | 0.01648 | 1.32 | 1.17 | 1.54 |
| BCL7A | B-cell CLL/lymphoma 7A (BCL7A), transcript variant 1 | NM_020993 | 0.03693 | 1.13 | 1.38 | 1.78 |
| TIGD5 | tigger transposable element derived 5 (TIGD5) | NM_032862 | 0.04105 | 1.23 | 1.19 | 1.37 |
| ARHGEF16 | Rho guanine exchange factor (GEF) 16 (ARHGEF16) | NM_014448 | 0.02514 | 1.95 | 1.23 | 1.05 |
| VIPR1 | vasoactive intestinal peptide receptor 1 (VIPR1) | NM_004624 | 0.00691 | 1.48 | 1.83 | 1.69 |
| APLN | apelin, AGTRL1 ligand (APLN) | NM_017413 | 0.01016 | -2.52 | -1.36 | -1.50 |
| EFEMP2 | EGF-containing fibulin-like extracellular matrix protein 2 (EFEMP2) | NM_016938 | 0.00369 | 1.24 | -1.02 | 1.72 |
| MAD1L1 | MAD1 mitotic arrest deficient-like 1 (yeast) (MAD1L1), transcript variant 1 | NM_003550 | 0.01107 | 1.30 | 1.09 | 1.30 |
| BCAR3 | breast cancer anti-estrogen resistance 3 (BCAR3) | NM_003567 | 0.02416 | -1.05 | -1.88 | -1.32 |
| PRKRIR | protein-kinase, interferon-inducible double stranded RNA dependent inhibitor, repressor of (P58 repressor) (PRKRIR) | NM_004705 | 0.01404 | -1.22 | -1.07 | -1.25 |
| CHD5 | chromodomain helicase DNA binding protein 5 (CHD5) | NM_015557 | 0.01641 | 2.12 | 1.58 | 1.37 |
| ANLN | anillin, actin binding protein (ANLN) | NM_018685 | 0.02693 | -2.81 | -1.21 | -1.21 |
| RHEB | Ras homolog enriched in brain (RHEB) | NM_005614 | 0.00003 | -1.80 | 1.05 | -6.55 |
| USF2 | upstream transcription factor 2, c-fos interacting (USF2), transcript variant 1 | NM_003367 | 0.01058 | 1.14 | 1.09 | 1.48 |
| RALA | v-ral simian leukemia viral oncogene homolog A (ras related) (RALA) | NM_005402 | 0.03195 | -1.08 | 1.08 | -1.44 |
| ZDHHC13 | zinc finger, DHHC-type containing 13 (ZDHHC13), transcript variant 1 | NM_019028 | 0.01449 | -1.03 | -1.25 | -2.37 |
| MDN1 | MDN1, midasin homolog (yeast) (MDN1) | NM_014611 | 0.02112 | -1.11 | 1.16 | 2.34 |
| SPAG5 | sperm associated antigen 5 (SPAG5) | NM_006461 | 0.00864 | -1.17 | -1.41 | -2.01 |
| MRGPRF | MAS-related GPR, member F (MRGPRF) | NM_145015 | 0.04491 | -1.12 | 1.87 | 1.74 |
| GAS8 | growth arrest-specific 8 (GAS8) | NM_001481 | 0.00013 | 1.24 | 1.34 | 1.45 |
| SHANK2 | SH3 and multiple ankyrin repeat domains 2 (SHANK2), transcript variant 1 | NM_012309 | 0.02328 | 1.26 | 3.35 | -1.19 |
| DLG3 | discs, large homolog 3 (neuroendocrine-dlg, Drosophila) (DLG3), transcript variant 1 | NM_021120 | 0.04086 | 1.70 | 1.29 | 1.55 |
| FLT3LG | fms-related tyrosine kinase 3 ligand (FLT3LG) | NM_001459 | 0.02979 | -1.04 | 2.89 | 1.53 |
| GIGYF1 | PERQ amino acid rich, with GYF domain 1 (PERQ1) | NM_022574 | 0.03116 | 1.26 | 1.25 | 1.40 |
| KLRC2 | killer cell lectin-like receptor subfamily C, member 2 (KLRC2) | NM_002260 | 0.03617 | -1.58 | -1.68 | -1.71 |
| RICH2 | KIAA0672 gene product (KIAA0672) | NM_014859 | 0.01838 | 1.02 | 1.32 | 1.63 |
| MTA1 | metastasis associated 1 (MTA1) | NM_004689 | 0.00807 | 1.18 | 1.08 | 2.02 |
| ARHGEF17 | Rho guanine nucleotide exchange factor (GEF) 17 (ARHGEF17) | NM_014786 | 0.00020 | 1.04 | 1.37 | 1.71 |
| DEPDC7 | DEP domain containing 7 (DEPDC7), transcript variant 2 | NM_139160 | 0.02394 | -1.13 | -1.46 | -1.70 |
| ZNF217 | zinc finger protein 217 (ZNF217) | NM_006526 | 0.02904 | -1.28 | -1.28 | -1.15 |
| VGLL3 | vestigial like 3 (Drosophila) (VGLL3) | NM_016206 | 0.01763 | -1.07 | 1.49 | 2.14 |
| BNIP3L | BCL2/adenovirus E1B 19kDa interacting protein 3-like (BNIP3L) | NM_004331 | 0.00004 | -2.52 | -1.12 | -18.47 |
| NCOA3 | nuclear receptor coactivator 3 (NCOA3), transcript variant 1 | NM_181659 | 0.02327 | 1.20 | -1.05 | 1.50 |
| SPSB3 | splA/ryanodine receptor domain and SOCS box containing 3 (SPSB3) | NM_080861 | 0.04311 | 1.46 | -1.04 | 1.49 |
| TIGD7 | tigger transposable element derived 7 (TIGD7) | NM_033208 | 0.02412 | 1.23 | 1.35 | 1.45 |
| GNG5 | guanine nucleotide binding protein (G protein), gamma 5 (GNG5) | NM_005274 | 0.03641 | 1.01 | -1.06 | -1.80 |
| DEFB1 | defensin, beta 1 (DEFB1) | NM_005218 | 0.00495 | 1.07 | -2.98 | -2.81 |
| GTPBP1 | GTP binding protein 1 (GTPBP1) | NM_004286 | 0.03939 | 1.04 | 1.16 | 1.57 |
| CHD4 | chromodomain helicase DNA binding protein 4 (CHD4) | NM_001273 | 0.00178 | 1.25 | 1.15 | 1.41 |
| COL4A4 | collagen, type IV, alpha 4 (COL4A4) | NM_000092 | 0.01571 | -1.55 | -4.90 | -1.06 |
| RAP2C | RAP2C, member of RAS oncogene family (RAP2C) | NM_021183 | 0.03119 | -1.41 | -1.23 | -1.24 |
| TANK | TRAF family member-associated NFKB activator (TANK), transcript variant 2 | NM_133484 | 0.01346 | -1.50 | -1.21 | -1.44 |
| GPR182 | adrenomedullin receptor (ADMR) | NM_007264 | 0.02346 | 1.78 | 1.11 | 1.60 |
| MKL1 | megakaryoblastic leukemia (translocation) 1 (MKL1) | NM_020831 | 0.04668 | 1.05 | 1.31 | 1.38 |
| ARHGAP18 | Rho GTPase activating protein 18 (ARHGAP18) | NM_033515 | 0.00077 | -2.30 | -1.22 | -1.61 |
| OXER1 | oxoeicosanoid (OXE) receptor 1 (OXER1) | NM_148962 | 0.00356 | 1.43 | 1.42 | 2.39 |
| SEPT 9 | septin 9 (SEPT9) | NM_006640 | 0.01894 | 1.04 | 1.31 | 1.66 |
| PPP2R5E | protein phosphatase 2, regulatory subunit B', epsilon isoform (PPP2R5E) | NM_006246 | 0.00326 | -1.63 | 1.03 | -4.62 |
| CGRRF1 | cell growth regulator with ring finger domain 1 (CGRRF1) | NM_006568 | 0.01117 | -1.70 | -1.22 | -1.30 |
| SNIP1 | Smad nuclear interacting protein 1 (SNIP1) | NM_024700 | 0.02820 | 1.03 | -1.14 | -1.33 |
| ARHGAP10 | Rho GTPase activating protein 10 (ARHGAP10) | NM_024605 | 0.04044 | 1.07 | 1.15 | 1.82 |
| IL1RAP | interleukin 1 receptor accessory protein (IL1RAP), transcript variant 2 | NM_134470 | 0.01122 | -1.76 | -1.53 | -2.18 |
| USH1C | Usher syndrome 1C (autosomal recessive, severe) (USH1C), transcript variant 1 | NM_005709 | 0.00506 | 1.60 | 4.33 | 1.47 |
| DRG2 | developmentally regulated GTP binding protein 2 (DRG2) | NM_001388 | 0.00521 | 1.27 | 1.13 | 1.70 |
| ZWILCH | Zwilch, kinetochore associated, homolog (Drosophila) (ZWILCH), transcript variant 1 | NM_017975 | 0.03538 | -1.45 | -1.35 | -1.11 |
| IL1RAP | interleukin 1 receptor accessory protein (IL1RAP), transcript variant 1 | NM_002182 | 0.00390 | -1.33 | -2.25 | -1.45 |
| NFKBIL1 | nuclear factor of kappa light polypeptide gene enhancer in B-cells inhibitor-like 1 (NFKBIL1) | NM_005007 | 0.00019 | 1.39 | 1.12 | 1.53 |
| F13B | coagulation factor XIII, B polypeptide (F13B) | NM_001994 | 0.00001 | -1.05 | -1.03 | -13.95 |
| CNKSR3 | CNKSR family member 3 (CNKSR3) | NM_173515 | 0.00228 | 1.31 | 1.24 | 1.51 |
| MAP4 | microtubule-associated protein 4 (MAP4), transcript variant 1 | NM_002375 | 0.04837 | 1.18 | 1.42 | 1.13 |
| FLCN | folliculin (FLCN), transcript variant 2 | NM_144606 | 0.02743 | 2.09 | 1.47 | 1.04 |
| RASL12 | RAS-like, family 12 (RASL12) | NM_016563 | 0.00240 | 1.88 | 1.82 | -1.10 |
| PRKCSH | protein kinase C substrate 80K-H (PRKCSH), transcript variant 1 | NM_002743 | 0.04396 | 1.23 | -1.08 | 1.65 |
| OSMR | oncostatin M receptor (OSMR) | NM_003999 | 0.04850 | -1.25 | -1.22 | -1.47 |
| RHOC | ras homolog gene family, member C (RHOC), transcript variant 1 | NM_175744 | 0.03328 | 1.07 | 1.01 | 1.21 |
| GNG8 | guanine nucleotide binding protein (G protein), gamma 8 (GNG8) | NM_033258 | 0.00010 | 1.59 | 1.77 | 1.41 |
| TH1L | TH1-like (Drosophila) (TH1L), transcript variant 1 | NM_198976 | 0.02673 | 1.19 | 1.13 | 1.14 |
| ARL1 | ADP-ribosylation factor-like 1 (ARL1) | NM_001177 | 0.01165 | -1.37 | -1.04 | -1.46 |
| FAM13B | chromosome 5 open reading frame 5 (C5orf5) | NM_016603 | 0.03425 | 1.18 | 1.20 | 1.43 |
| NTSR1 | neurotensin receptor 1 (high affinity) (NTSR1) | NM_002531 | 0.01127 | 2.15 | 1.71 | -1.03 |
| BRMS1 | breast cancer metastasis suppressor 1 (BRMS1), transcript variant 1 | NM_015399 | 0.03607 | 1.02 | 1.14 | 1.49 |
| C11orf9 | chromosome 11 open reading frame 9 (C11orf9) | NM_013279 | 0.00197 | 1.91 | 1.11 | 1.74 |
| ASCC1 | activating signal cointegrator 1 complex subunit 1 (ASCC1) | NM_015947 | 0.04672 | -1.14 | -1.08 | -1.14 |
| ARL3 | ADP-ribosylation factor-like 3 (ARL3) | NM_004311 | 0.02168 | 1.91 | 1.25 | 1.11 |
| PTGIR | prostaglandin I2 (prostacyclin) receptor (IP) (PTGIR) | NM_000960 | 0.01420 | 1.72 | 1.34 | 1.14 |
| GNB3 | guanine nucleotide binding protein (G protein), beta polypeptide 3 (GNB3) | NM_002075 | 0.02475 | 1.19 | 1.58 | 1.60 |
| WDR6 | WD repeat domain 6 (WDR6) | NM_018031 | 0.02480 | 1.12 | 1.05 | 1.60 |
| SORCS3 | sortilin-related VPS10 domain containing receptor 3 (SORCS3) | NM_014978 | 0.00329 | 2.27 | 5.00 | 2.92 |
| DEDD | death effector domain containing (DEDD), transcript variant 1 | NM_032998 | 0.01186 | 1.52 | 1.12 | 1.21 |
| DEF8 | hypothetical protein FLJ20186 (FLJ20186), transcript variant 2 | NM_017702 | 0.00990 | 1.21 | 1.14 | 1.55 |
| HRSP12 | heat-responsive protein 12 (HRSP12) | NM_005836 | 0.01064 | 1.12 | 1.01 | -19.35 |
| ARPC2 | actin related protein 2/3 complex, subunit 2, 34kDa (ARPC2), transcript variant 1 | NM_152862 | 0.02486 | -1.34 | -1.21 | -1.38 |
| IL15RA | interleukin 15 receptor, alpha (IL15RA), transcript variant 2 | NM_172200 | 0.00004 | -2.77 | -2.54 | -5.78 |
| NDFIP2 | Nedd4 family interacting protein 2 (NDFIP2) | NM_019080 | 0.00917 | -1.27 | -1.83 | -1.11 |
| TMEM115 | transmembrane protein 115 (TMEM115) | NM_007024 | 0.00591 | 1.08 | 1.15 | 1.47 |
| MCC | mutated in colorectal cancers (MCC) | NM_002387 | 0.03694 | -1.34 | -1.38 | -1.41 |
| CHRM4 | cholinergic receptor, muscarinic 4 (CHRM4) | NM_000741 | 0.00920 | 1.52 | 1.29 | 1.23 |
| ADAMTSL4 | ADAMTS-like 4 (ADAMTSL4), transcript variant 1 | NM_019032 | 0.00056 | 1.52 | 1.26 | 2.01 |
| DTX3 | deltex 3 homolog (Drosophila) (DTX3) | NM_178502 | 0.03879 | -1.08 | 1.27 | 1.88 |
| ZC3H15 | zinc finger CCCH-type containing 15 (ZC3H15) | NM_018471 | 0.01236 | -1.31 | 1.04 | -1.25 |
| ACTR3 | ARP3 actin-related protein 3 homolog (yeast) (ACTR3) | NM_005721 | 0.02527 | -1.73 | -1.11 | -1.28 |
| ARHGEF18 | rho/rac guanine nucleotide exchange factor (GEF) 18 (ARHGEF18) | NM_015318 | 0.00806 | 1.31 | 1.24 | 1.59 |
| TFG | TRK-fused gene (TFG), transcript variant 1 | NM_006070 | 0.04734 | -1.14 | 1.01 | -1.31 |
| MXD4 | MAX dimerization protein 4 (MXD4) | NM_006454 | 0.00062 | 1.43 | 1.14 | 2.08 |
| NPFF | neuropeptide FF-amide peptide precursor (NPFF) | NM_003717 | 0.00644 | 1.34 | 1.10 | 1.75 |
| CHN1 | chimerin (chimaerin) 1 (CHN1), transcript variant 1 | NM_001822 | 0.00442 | -1.14 | 1.13 | -8.04 |
| C9orf86 | chromosome 9 open reading frame 86 (C9orf86) | NM_024718 | 0.01210 | 1.03 | 1.20 | 2.02 |
| PLEKHG6 | pleckstrin homology domain containing, family G (with RhoGef domain) member 6 (PLEKHG6) | NM_018173 | 0.04100 | 1.52 | -1.03 | 1.50 |
| ANXA7 | annexin A7 (ANXA7), transcript variant 2 | NM_004034 | 0.02005 | -1.24 | -1.02 | -1.33 |
| RASA2 | RAS p21 protein activator 2 (RASA2) | NM_006506 | 0.02541 | -1.37 | -1.21 | -1.15 |
| CREB3L4 | cAMP responsive element binding protein 3-like 4 (CREB3L4) | NM_130898 | 0.04420 | -1.40 | 1.03 | -1.62 |
| RBPJL | recombination signal binding protein for immunoglobulin kappa J region-like (RBPJL) | NM_014276 | 0.04816 | 1.53 | 1.30 | 1.15 |
| ARHGAP11A | Rho GTPase activating protein 11A (ARHGAP11A), transcript variant 1 | NM_014783 | 0.02111 | -2.34 | -1.34 | -1.43 |
| STAT6 | signal transducer and activator of transcription 6, interleukin-4 induced (STAT6) | NM_003153 | 0.04003 | -1.05 | -1.05 | 2.31 |
| GNL2 | guanine nucleotide binding protein-like 2 (nucleolar) (GNL2) | NM_013285 | 0.00885 | -1.39 | 1.35 | -19.52 |
| BAT5 | HLA-B associated transcript 5 (BAT5) | NM_021160 | 0.00121 | 1.32 | 1.12 | 1.36 |
| RNF6 | ring finger protein (C3H2C3 type) 6 (RNF6), transcript variant 1 | NM_005977 | 0.03611 | -1.21 | -1.16 | -1.42 |
| CYTSA | SPECC1-like (SPECC1L) | NM_015330 | 0.00896 | 1.07 | 1.21 | 1.38 |
| CHAC2 | ChaC, cation transport regulator homolog 2 (E. coli) (CHAC2) | NM_001008708 | 0.04202 | -1.69 | -1.02 | -1.19 |
| TUB | tubby homolog (mouse) (TUB), transcript variant 1 | NM_003320 | 0.00213 | 1.22 | 1.42 | 1.41 |
| H1FOO | H1 histone family, member O, oocyte-specific (H1FOO) | NM_153833 | 0.01111 | 3.78 | 1.40 | -1.45 |
| FAM50A | family with sequence similarity 50, member A (FAM50A) | NM_004699 | 0.03709 | 1.32 | 1.30 | 1.27 |
| IL1R2 | interleukin 1 receptor, type II (IL1R2), transcript variant 1 | NM_004633 | 0.00435 | -1.87 | -4.78 | -1.69 |
| CUTA | cutA divalent cation tolerance homolog (E. coli) (CUTA), transcript variant 2 | NM_015921 | 0.02186 | 1.24 | 1.11 | 1.12 |
| LIME1 | Lck interacting transmembrane adaptor 1 (LIME1) | NM_017806 | 0.02825 | 1.54 | 1.25 | 1.25 |
| IK | IK cytokine, down-regulator of HLA II (IK) | NM_006083 | 0.03459 | 1.26 | 1.23 | 1.24 |
| CEP110 | centrosomal protein 110kDa (CEP110) | NM_007018 | 0.01043 | 1.28 | 1.14 | 1.80 |
| YTHDF2 | YTH domain family, member 2 (YTHDF2) | NM_016258 | 0.00773 | -1.19 | -1.20 | -1.59 |
| FAM96B | family with sequence similarity 96, member B (FAM96B) | NM_016062 | 0.00588 | -1.22 | 1.18 | -2.43 |
| SH3PXD2A | SH3 and PX domains 2A (SH3PXD2A) | NM_014631 | 0.01884 | 1.58 | 1.11 | 1.49 |
| MAK10 | MAK10 homolog, amino-acid N-acetyltransferase subunit, (S. cerevisiae) (MAK10) | NM_024635 | 0.00001 | -1.42 | -1.12 | -3.08 |
| MEGF8 | chromosome 19 open reading frame 49 (C19orf49) | NM_178121 | 0.04621 | 1.05 | 1.16 | 2.21 |
| GLTSCR2 | glioma tumor suppressor candidate region gene 2 (GLTSCR2) | NM_015710 | 0.01246 | 1.15 | 1.15 | 1.37 |
| CHORDC1 | cysteine and histidine-rich domain (CHORD)-containing 1 (CHORDC1) | NM_012124 | 0.00227 | -1.68 | -1.11 | -1.78 |
| BRD3 | bromodomain containing 3 (BRD3) | NM_007371 | 0.02679 | 1.18 | 1.05 | 1.50 |
| LENG1 | leukocyte receptor cluster (LRC) member 1 (LENG1) | NM_024316 | 0.00143 | 1.32 | 1.50 | 1.62 |
| TMEM117 | transmembrane protein 117 (TMEM117) | NM_032256 | 0.03228 | -1.25 | -1.11 | -1.24 |
| BSCL2 | Bernardinelli-Seip congenital lipodystrophy 2 (seipin) (BSCL2) | NM_032667 | 0.01681 | 1.10 | 1.29 | 1.34 |
| LXN | latexin (LXN) | NM_020169 | 0.03543 | -1.66 | -1.21 | -1.30 |
| BCL7B | B-cell CLL/lymphoma 7B (BCL7B), transcript variant 2 | NM_138707 | 0.00001 | -1.43 | 1.13 | -8.23 |
| KPTN | kaptin (actin binding protein) (KPTN) | NM_007059 | 0.04078 | 1.22 | 1.18 | 1.35 |
| NCAPH2 | non-SMC condensin II complex, subunit H2 (NCAPH2), transcript variant 2 | NM_152299 | 0.00453 | 1.13 | 1.16 | 1.38 |
| ATRN | attractin (ATRN), transcript variant 1 | NM_139321 | 0.00057 | 1.49 | 1.14 | 1.76 |
| CASKIN2 | CASK interacting protein 2 (CASKIN2) | NM_020753 | 0.00253 | 1.24 | 1.12 | 1.37 |
| H2AFZ | H2A histone family, member Z (H2AFZ) | NM_002106 | 0.00223 | -1.17 | -1.32 | -1.78 |
| C9orf114 | chromosome 9 open reading frame 114 (C9orf114) | NM_016390 | 0.01153 | 1.20 | 1.28 | 1.27 |
| EPB41L1 | erythrocyte membrane protein band 4.1-like 1 (EPB41L1), transcript variant 1 | NM_012156 | 0.03217 | 1.11 | 1.42 | 1.16 |
| WASF3 | WAS protein family, member 3 (WASF3) | NM_006646 | 0.00270 | -1.13 | 1.57 | -14.21 |
| RCC2 | regulator of chromosome condensation 2 (RCC2) | NM_018715 | 0.02158 | 1.28 | 1.04 | 1.70 |
| SRRT | ARS2 protein (ARS2), transcript variant 2 | NM_182800 | 0.00599 | 1.20 | 1.21 | 1.72 |
| SRRT | ARS2 protein (ARS2), transcript variant 2 | NM_182800 | 0.01144 | 1.14 | 1.21 | 1.53 |
| KBTBD7 | kelch repeat and BTB (POZ) domain containing 7 (KBTBD7) | NM_032138 | 0.04196 | -1.35 | 1.03 | -1.91 |
| STRN | striatin, calmodulin binding protein (STRN) | NM_003162 | 0.03316 | -1.62 | -1.71 | 1.14 |
| TEKT5 | tektin 5 (TEKT5) | NM_144674 | 0.04026 | 1.44 | 1.73 | 1.12 |
| TUBGCP2 | tubulin, gamma complex associated protein 2 (TUBGCP2) | NM_006659 | 0.01039 | 1.18 | 1.05 | 1.38 |
| HIST1H2BH | histone cluster 1, H2bh (HIST1H2BH) | NM_003524 | 0.00814 | 1.00 | -1.48 | -2.15 |
| AVPI1 | arginine vasopressin-induced 1 (AVPI1) | NM_021732 | 0.02581 | -1.01 | 1.34 | 1.58 |
| PLS3 | plastin 3 (T isoform) (PLS3) | NM_005032 | 0.04937 | -1.15 | -1.17 | -1.40 |
| KIAA1199 | KIAA1199 (KIAA1199) | NM_018689 | 0.01453 | 1.25 | 1.60 | 2.87 |
| HIST1H2BO | histone cluster 1, H2bo (HIST1H2BO) | NM_003527 | 0.01265 | 1.20 | 1.16 | 2.58 |
| C12orf10 | chromosome 12 open reading frame 10 (C12orf10) | NM_021640 | 0.01072 | 1.13 | 1.26 | 1.25 |
| MFAP1 | microfibrillar-associated protein 1 (MFAP1) | NM_005926 | 0.04063 | -1.27 | -1.13 | -1.53 |
| NUDCD1 | NudC domain containing 1 (NUDCD1) | NM_032869 | 0.00261 | -1.33 | -1.13 | -1.39 |
| CDCA5 | cell division cycle associated 5 (CDCA5) | NM_080668 | 0.04400 | -2.36 | -1.55 | -1.18 |
| HIST1H2BL | histone cluster 1, H2bl (HIST1H2BL) | NM_003519 | 0.03123 | 1.30 | -1.01 | -6.28 |
| HIST1H1E | histone cluster 1, H1e (HIST1H1E) | NM_005321 | 0.04213 | -2.00 | 1.03 | -2.52 |
| FAM96A | family with sequence similarity 96, member A (FAM96A), transcript variant 1 | NM_032231 | 0.00015 | -1.24 | -1.34 | -1.44 |
| EFNA4 | ephrin-A4 (EFNA4), transcript variant 1 | NM_005227 | 0.00709 | 1.08 | -2.84 | -2.77 |
| ASPSCR1 | alveolar soft part sarcoma chromosome region, candidate 1 (ASPSCR1) | NM_024083 | 0.02753 | 1.19 | 1.21 | 1.40 |
| NUBP1 | nucleotide binding protein 1 (MinD homolog, E. coli) (NUBP1) | NM_002484 | 0.02962 | -1.50 | 1.05 | -1.41 |
| C19orf33 | chromosome 19 open reading frame 33 (C19orf33) | NM_033520 | 0.00606 | 1.92 | -1.23 | 2.05 |
| PALLD | palladin, cytoskeletal associated protein (PALLD) | NM_016081 | 0.04381 | -1.59 | 1.08 | -1.34 |
| C9orf61 | chromosome 9 open reading frame 61 (C9orf61) | NM_004816 | 0.02247 | 1.09 | 1.26 | 1.78 |
| FAM50A | family with sequence similarity 50, member A (FAM50A) | NM_004699 | 0.03427 | 1.38 | 1.26 | 1.30 |
| C17orf75 | chromosome 17 open reading frame 75 (C17orf75) | NM_022344 | 0.00136 | -3.76 | -3.45 | -1.44 |
| CCDC130 | coiled-coil domain containing 130 (CCDC130) | NM_030818 | 0.00032 | 1.20 | 1.21 | 1.77 |
| C6orf145 | chromosome 6 open reading frame 145 (C6orf145) | NM_183373 | 0.00011 | -2.14 | -1.05 | -4.62 |
| CXCL3 | chemokine (C-X-C motif) ligand 3 (CXCL3) | NM_002090 | 0.04904 | -1.10 | -3.91 | 1.17 |
| EDC4 | enhancer of mRNA decapping 4 (EDC4) | NM_014329 | 0.01587 | 1.19 | 1.18 | 1.04 |
| LUM | lumican (LUM) | NM_002345 | 0.04282 | -2.08 | -1.18 | -1.03 |
| CCDC5 | coiled-coil domain containing 5 (spindle associated) (CCDC5) | NM_138443 | 0.02641 | -1.30 | -1.12 | -1.22 |
| FAM3A | family with sequence similarity 3, member A (FAM3A) | NM_021806 | 0.04625 | 1.07 | 1.26 | 1.34 |
| IL17D | interleukin 17D (IL17D) | NM_138284 | 0.04335 | 2.15 | 1.48 | 1.16 |
| BOLA3 | bolA homolog 3 (E. coli) (BOLA3), transcript variant 1 | NM_212552 | 0.00176 | -1.33 | -1.07 | -3.72 |
| TMEM106C | transmembrane protein 106C (TMEM106C) | NM_024056 | 0.03222 | -1.08 | -1.22 | -1.18 |
| IFNA6 | interferon, alpha 6 (IFNA6) | NM_021002 | 0.04820 | 1.45 | 1.29 | 1.03 |
| EDEM3 | ER degradation enhancer, mannosidase alpha-like 3 (EDEM3) | NM_025191 | 0.00013 | -1.73 | -1.12 | -3.70 |
| SPC24 | SPC24, NDC80 kinetochore complex component, homolog (S. cerevisiae) (SPC24) | NM_182513 | 0.02811 | -2.44 | -2.30 | -1.85 |
| C9orf7 | chromosome 9 open reading frame 7 (C9orf7) | NM_017586 | 0.01977 | 1.59 | 1.85 | 1.35 |
| NUB1 | negative regulator of ubiquitin-like proteins 1 (NUB1) | NM_016118 | 0.02582 | 1.18 | 1.20 | 1.41 |
| CD200R1 | CD200 receptor 1 (CD200R1), transcript variant 1 | NM_138806 | 0.04967 | 1.05 | -4.72 | -1.32 |
| TMEM107 | transmembrane protein 107 (TMEM107), transcript variant 1 | NM_032354 | 0.01918 | -1.26 | -1.14 | -1.59 |
| TULP1 | tubby like protein 1 (TULP1) | NM_003322 | 0.02748 | 2.15 | 1.61 | -1.27 |
| DAAM1 | dishevelled associated activator of morphogenesis 1 (DAAM1) | NM_014992 | 0.04826 | -1.65 | -1.32 | -1.15 |
| MINA | MYC induced nuclear antigen (MINA), transcript variant 3 | NM_032778 | 0.02927 | 1.21 | 1.09 | 1.80 |
| BRD1 | bromodomain containing 1 (BRD1) | NM_014577 | 0.04710 | 1.25 | -1.01 | 1.71 |
| MNS1 | meiosis-specific nuclear structural 1 (MNS1) | NM_018365 | 0.01008 | -1.53 | -1.18 | -2.31 |
| LYPLAL1 | lysophospholipase-like 1 (LYPLAL1) | NM_138794 | 0.00279 | -1.69 | -1.10 | -1.47 |
| MFSD11 | major facilitator superfamily domain containing 11 (MFSD11) | NM_024311 | 0.03992 | -1.24 | -1.15 | -1.26 |
| ACTR2 | ARP2 actin-related protein 2 homolog (yeast) (ACTR2), transcript variant 1 | NM_001005386 | 0.03420 | -1.36 | -1.14 | -1.19 |
| CCDC22 | coiled-coil domain containing 22 (CCDC22) | NM_014008 | 0.00156 | 1.03 | 1.04 | 2.09 |
| CDCA8 | cell division cycle associated 8 (CDCA8) | NM_018101 | 0.04813 | -1.42 | -1.44 | -1.45 |
| PDGFRL | platelet-derived growth factor receptor-like (PDGFRL) | NM_006207 | 0.01140 | -1.21 | -2.94 | -1.55 |
| GLI4 | GLI-Kruppel family member GLI4 (GLI4) | NM_138465 | 0.00645 | 1.18 | 1.31 | 1.39 |
| LEAP2 | liver expressed antimicrobial peptide 2 (LEAP2) | NM_052971 | 0.00607 | -1.85 | 1.23 | -2.91 |
| TRIM39 | tripartite motif-containing 39 (TRIM39), transcript variant 1 | NM_021253 | 0.00736 | 1.21 | 1.24 | 1.11 |
| PSMG2 | tumor necrosis factor superfamily, member 5-induced protein 1 (TNFSF5IP1) | NM_020232 | 0.01199 | -1.12 | -1.09 | -1.78 |
| NAP1L4 | nucleosome assembly protein 1-like 4 (NAP1L4) | NM_005969 | 0.00877 | -1.17 | 1.04 | -2.23 |
| ADCK4 | aarF domain containing kinase 4 (ADCK4) | NM_024876 | 0.0355793 | 1.1735 | 1.0802 | 1.496 |
| ANP32E | acidic (leucine-rich) nuclear phosphoprotein 32 family, member E (ANP32E) | NM_030920 | 0.0234312 | -1.582 | -1.19 | -1.637 |
| ANKMY1 | ankyrin repeat and MYND domain containing 1 (ANKMY1), transcript variant 1 | NM_016552 | 0.0475667 | 1.8567 | 1.3478 | -1.1 |
| ANTXR1 | anthrax toxin receptor 1 (ANTXR1), transcript variant 1 | NM_032208 | 0.0116547 | -1.364 | -1.188 | -1.066 |
| ARRDC4 | arrestin domain containing 4 (ARRDC4) | NM_183376 | 0.0386787 | -1.798 | -1.262 | -1.452 |
| CASD1 | CAS1 domain containing 1 (CASD1) | NM_022900 | 0.0076723 | -1.429 | -1.032 | -1.611 |
| COBLL1 | COBL-like 1 (COBLL1) | NM_014900 | 0.025135 | -1.359 | 1.1861 | -4.791 |
| CCDC9 | coiled-coil domain containing 9 (CCDC9) | NM_015603 | 0.0236989 | 1.0593 | 1.1103 | 1.5661 |
| CHCHD1 | coiled-coil-helix-coiled-coil-helix domain containing 1 (CHCHD1) | NM_203298 | 0.0135115 | -1.037 | 1.0325 | -1.792 |
| COMMD10 | COMM domain containing 10 (COMMD10) | NM_016144 | 0.0049017 | -1.162 | -1.196 | -1.755 |
| COMMD8 | COMM domain containing 8 (COMMD8) | NM_017845 | 0.0311983 | -1.127 | -1.131 | -2.003 |
| COPS7B | COP9 constitutive photomorphogenic homolog subunit 7B (Arabidopsis) (COPS7B) | NM_022730 | 0.0103264 | 1.1195 | 1.5379 | 2.1014 |
| CPNE2 | copine II (CPNE2) | NM_152727 | 0.0065479 | -1.292 | -1.104 | -3.159 |
| CTDSP2 | CTD (carboxy-terminal domain, RNA polymerase II, polypeptide A) small phosphatase 2 (CTDSP2) | NM_005730 | 0.0460098 | 1.5339 | -1.009 | 1.2795 |
| CTDSPL2 | CTD (carboxy-terminal domain, RNA polymerase II, polypeptide A) small phosphatase like 2 (CTDSPL2) | NM_016396 | 0.0294279 | -1.077 | -1.018 | -1.414 |
| LRRC8A | leucine rich repeat containing 8 family, member A (LRRC8A) | NM_019594 | 0.0091482 | 1.2205 | 1.0711 | 1.5137 |
| LIMS2 | LIM and senescent cell antigen-like domains 2 (LIMS2) | NM_017980 | 0.0368221 | 1.0083 | 1.1745 | 2.0589 |
| LMBR1 | limb region 1 homolog (mouse) (LMBR1) | NM_022458 | 0.0136665 | -1.211 | 1.0233 | -1.327 |
| OCIAD1 | OCIA domain containing 1 (OCIAD1), transcript variant 1 | NM_017830 | 0.0461919 | -1.361 | -1.208 | -2.168 |
| PACRG | PARK2 co-regulated (PACRG), transcript variant 1 | NM_152410 | 0.0338442 | 1.5738 | 1.2106 | 1.6604 |
| PPP2R1B | protein phosphatase 2 (formerly 2A), regulatory subunit A, beta isoform (PPP2R1B), transcript variant 1 | NM_002716 | 0.003326 | 2.1499 | 1.925 | 1.6938 |
| RBBP9 | retinoblastoma binding protein 9 (RBBP9) | NM_006606 | 0.026729 | -1.474 | -11.01 | -5.131 |
| CNPY2 | transmembrane protein 4 (TMEM4) | NM_014255 | 6.12E-05 | -2.034 | -1.346 | -24.28 |
| TRIM37 | tripartite motif-containing 37 (TRIM37), transcript variant 2 | NM_001005207 | 0.0033857 | -1.079 | -1.293 | -3.818 |
| VSIG2 | V-set and immunoglobulin domain containing 2 (VSIG2) | NM_014312 | 3.37E-04 | 2.0946 | 1.1448 | 1.7906 |
| NM_031295 | Williams Beuren syndrome chromosome region 21 (WBSCR21), transcript variant 4 | NM_031295 | 0.0127052 | 1.1439 | 1.204 | 1.3341 |
| WBP5 | WW domain binding protein 5 (WBP5), transcript variant 1 | NM_016303 | 0.0026197 | -1.466 | -1.234 | -4.25 |
| ZBED3 | zinc finger, BED-type containing 3 (ZBED3) | NM_032367 | 0.0257097 | 1.4268 | 1.1769 | 2.1253 |
| ZNHIT2 | zinc finger, HIT type 2 (ZNHIT2) | NM_014205 | 0.0368779 | 1.1197 | 1.1824 | 1.2172 |
| ZMYND10 | zinc finger, MYND-type containing 10 (ZMYND10) | NM_015896 | 0.0064684 | 1.2424 | 1.5326 | 1.3561 |
| NM_014057 | osteoglycin (osteoinductive factor, mimecan) (OGN), transcript variant 3 | NM_014057 | 0.0423393 | -1.539 | -1.188 | -1.243 |
| NM_000383 | autoimmune regulator (autoimmune polyendocrinopathy candidiasis ectodermal dystrophy) (AIRE), transcript variant AIRE-1 | NM_000383 | 0.0016904 | 2.2316 | 1.7385 | 5.0058 |
| NM_001009811 | 5-azacytidine induced 1 (AZI1), transcript variant 2 | NM_001009811 | 0.0243037 | 1.1623 | 1.124 | 1.769 |
| NM_080794 | mitochondrial ribosomal protein L39 (MRPL39), nuclear gene encoding mitochondrial protein, transcript variant 2 | NM_080794 | 0.0025726 | -1.278 | -1.006 | -1.961 |
| NM_012230 | POM (POM121 homolog, rat) and ZP3 fusion (POMZP3), transcript variant 1 | NM_012230 | 0.0495495 | 1.7243 | 1.2275 | -1.114 |
| NM_152856 | RNA binding motif protein 10 (RBM10), transcript variant 2 | NM_152856 | 0.0454596 | -1.04 | 1.2445 | 1.3284 |
| NM_007222 | zinc fingers and homeoboxes 1 (ZHX1) | NM_007222 | 0.0363114 | -1.274 | 1.0178 | -1.165 |
| D86962 | mRNA for KIAA0207 gene, partial cds [D86962] | D86962 | 0.0336714 | 1.5502 | -1.097 | 1.5758 |

| **Non Classified Genes** | |  |  |  |  |  |
| --- | --- | --- | --- | --- | --- | --- |
| OPLAH | 5-oxoprolinase (ATP-hydrolysing) (OPLAH) | NM_017570 | 0.0134769 | 1.4797 | -1.075 | 2.0609 |
| NM_003815 | a disintegrin and metalloproteinase domain 15 (metargidin) (ADAM15), transcript variant 2 | NM_003815 | 0.0266863 | 1.0489 | 1.0939 | 1.8613 |
| NM_021779 | a disintegrin and metalloproteinase domain 29 (ADAM29), transcript variant 3 | NM_021779 | 0.0467442 | 1.6992 | 1.4254 | 1.1832 |
| ADCK5 | aarF domain containing kinase 5 (ADCK5) | NM_174922 | 0.0220769 | 1.2358 | 1.0851 | 1.4387 |
| ABHD10 | abhydrolase domain containing 10 (ABHD10) | NM_018394 | 0.0369155 | -1.857 | 1.1825 | -2.021 |
| NM_031295 | abhydrolase domain containing 11 (ABHD11), transcript variant 4 | NM_031295 | 0.0152716 | 1.2027 | 1.2111 | 1.3386 |
| ABHD13 | abhydrolase domain containing 13 (ABHD13) | NM_032859 | 1.11E-04 | -1.32 | 1.0055 | -3.541 |
| NM_015831 | acetylcholinesterase (YT blood group) (ACHE), transcript variant E4-E5 | NM_015831 | 0.0353457 | -1.653 | 1.0023 | -1.643 |
| NM_000664 | acetyl-Coenzyme A carboxylase alpha (ACACA), transcript variant 6 | NM_000664 | 0.0420291 | 1.1232 | 1.4534 | 1.2888 |
| ADD3 | adducin 3 (gamma) (ADD3), transcript variant 1 | NM_016824 | 0.0085665 | -1.074 | -1.574 | -1.627 |
| NM_001132 | AFG3 ATPase family gene 3-like 1 (yeast) (AFG3L1) | NM_001132 | 6.21E-05 | 2.4404 | 1.4493 | 2.115 |
| CB229015 | AGENCOURT_11499445 NICHD_Rh_Ov1 Macaca mulatta cDNA clone IMAGE:6884874 5' | CB229015 | 0.0165922 | -1.661 | 1.156 | -1.716 |
| CB309156 | AGENCOURT_11823313 NICHD_Rh_Ov1 Macaca mulatta cDNA clone IMAGE:6917650 5' | CB309156 | 0.0200228 | -1.91 | -1.183 | -1.619 |
| CB312177 | AGENCOURT_11828698 NICHD_Rh_Ov1 Macaca mulatta cDNA clone IMAGE:6913202 5' | CB312177 | 0.0408117 | -1.222 | -1.19 | -1.302 |
| CB310061 | AGENCOURT_11876913 NICHD_Rh_Ov1 Macaca mulatta cDNA clone IMAGE:6913844 5' | CB310061 | 0.0427633 | -1.231 | -1.055 | -1.675 |
| BM918631 | AGENCOURT_6635016 NIH_MGC_120 cDNA clone IMAGE:5747645 5' | BM918631 | 0.0187019 | -1.202 | 1.064 | -1.643 |
| NM_031436 | aldo-keto reductase family 1, member C-like 2 (AKR1CL2) | NM_031436 | 0.0357839 | 1.7406 | 2.6904 | 1.6987 |
| NM_012103 | ancient ubiquitous protein 1 (AUP1), transcript variant 1 | NM_012103 | 0.0409366 | 1.1646 | 1.1096 | 1.0897 |
| ANKHD1 | ankyrin repeat and KH domain containing 1 (ANKHD1), transcript variant 3 | NM_024668 | 7.35E-05 | -1.649 | -1.074 | -3.353 |
| ANKS6 | ankyrin repeat and sterile alpha motif domain containing 6 (ANKS6) | NM_173551 | 0.0137324 | 1.0936 | 1.0993 | 1.5804 |
| ANKZF1 | ankyrin repeat and zinc finger domain containing 1 (ANKZF1), transcript variant 1 | NM_018089 | 0.0031965 | 1.4676 | 1.29 | 2.0378 |
| ANKRD13D | ankyrin repeat domain 13 family, member D (ANKRD13D) | NM_207354 | 0.032946 | -1.035 | -1.029 | 1.9804 |
| ANKRD23 | ankyrin repeat domain 23 (ANKRD23) | NM_144994 | 0.0203696 | 1.1803 | 1.3863 | 2.0456 |
| NM_005876 | aortic preferentially expressed protein 1 (APEG1) | NM_005876 | 0.0050657 | -1.039 | 1.7527 | 1.6762 |
| APOOL | apolipoprotein O-like (APOOL) | NM_198450 | 0.0388166 | -1.218 | -1.121 | -2.051 |
| NM_031920 | ARG99 protein (ARG99) | NM_031920 | 0.0080453 | 2.0774 | 1.4688 | 1.2971 |
| ARMC5 | armadillo repeat containing 5 (ARMC5) | NM_024742 | 0.0476537 | 1.1312 | 1.1508 | 1.4531 |
| ARMCX3 | armadillo repeat containing, X-linked 3 (ARMCX3), transcript variant 1 | NM_016607 | 1.09E-04 | -1.981 | -1.004 | -4.819 |
| NM_000046 | arylsulfatase B (ARSB), transcript variant 1 | NM_000046 | 0.030381 | 1.2644 | 1.5414 | 1.1706 |
| ALG14 | asparagine-linked glycosylation 14 homolog (S. cerevisiae) (ALG14) | NM_144988 | 0.0020183 | -1.717 | -1.22 | -1.238 |
| GPN3 | ATP binding domain 1 family, member C (ATPBD1C) | NM_016301 | 0.0257114 | -1.156 | 1.0052 | -1.441 |
| NM_001001486 | ATPase, Ca++ transporting, type 2C, member 1 (ATP2C1), transcript variant 4 | NM_001001486 | 0.0125325 | 1.2287 | 1.0126 | 1.2347 |
| AV657608 | AV657608 GLC cDNA clone GLCFDF10 3' | AV657608 | 0.0079894 | -1.205 | -1.431 | -2.203 |
| B4GALNT4 | beta-1,4-N-acetyl-galactosaminyl transferase 4 (B4GALNT4) | NM_178537 | 0.0115372 | 1.6941 | 2.2814 | 1.143 |
| BRI3BP | BRI3 binding protein (BRI3BP) | NM_080626 | 0.021461 | -1.711 | 1.0911 | -2.035 |
| NM_001009877 | bromodomain containing 9 (BRD9), transcript variant 2 | NM_001009877 | 0.0044369 | 1.6329 | 1.2531 | 1.5533 |
| BSDC1 | BSD domain containing 1 (BSDC1) | NM_018045 | 0.0158289 | 1.4369 | 1.0302 | 1.3926 |
| CAMK2N1 | calcium/calmodulin-dependent protein kinase II inhibitor 1 (CAMK2N1) | NM_018584 | 0.010772 | -1.319 | 1.1082 | -2.478 |
| CAST | calpastatin (CAST), transcript variant 2 | NM_173060 | 0.0168764 | -1.589 | -1.122 | -1.963 |
| NM_201377 | cancer susceptibility candidate 2 (CASC2) | NM_201377 | 0.0280449 | 1.9771 | 1.7162 | -1.728 |
| CTAG1A | cancer/testis antigen 1A (CTAG1A) | NM_139250 | 0.0014902 | 1.2862 | 1.6596 | 1.5002 |
| CTAG1A | cancer/testis antigen 1A (CTAG1A) | NM_139250 | 2.21E-04 | 1.498 | 1.7852 | 1.4087 |
| NM_152247 | carnitine palmitoyltransferase 1B (muscle) (CPT1B), nuclear gene encoding mitochondrial protein, transcript variant 4 | NM_152247 | 0.0353671 | 1.2539 | -1.092 | 2.0044 |
| NM_001223 | caspase 1, apoptosis-related cysteine protease (interleukin 1, beta, convertase) (CASP1), transcript variant beta | NM_001223 | 0.0389773 | -1.689 | -1.394 | -1.1 |
| NM_054020 | cation channel, sperm associated 2 (CATSPER2), transcript variant 1 | NM_054020 | 0.0330084 | -1.129 | -1.147 | -5.916 |
| CD163L1 | CD163 molecule-like 1 (CD163L1) | NM_174941 | 0.004596 | -1.605 | -2.454 | -3.184 |
| NM_207397 | CD164 sialomucin-like 2 (CD164L2) | NM_207397 | 0.0120353 | 1.8732 | 1.3845 | 1.0548 |
| NM_001291 | CDC-like kinase 2 (CLK2), transcript variant 2 | NM_001291 | 0.039457 | 1.1178 | 1.1058 | 1.4215 |
| NM_025197 | CDK5 regulatory subunit associated protein 3 (CDK5RAP3), transcript variant 2 | NM_025197 | 0.0340461 | 1.2567 | 1.2689 | 1.3841 |
| BC017041 | cDNA clone IMAGE:3846805, partial cds [BC017041] | BC017041 | 0.0279464 | 1.8158 | 1.325 | -1.179 |
| BC110642 | cDNA clone MGC:110972 IMAGE:6144610, complete cds [BC110642] | BC110642 | 0.0454145 | 1.1919 | 1.392 | 1.0294 |
| AK131251 | cDNA FLJ16178 fis, clone BRHIP3000017 [AK131251] | AK131251 | 8.26E-04 | 2.2227 | 1.282 | 1.3235 |
| AK055375 | cDNA FLJ30813 fis, clone FEBRA2001523 [AK055375] | AK055375 | 0.0371374 | -1.163 | -1.046 | -1.14 |
| AK056473 | cDNA FLJ31911 fis, clone NT2RP7004751 [AK056473] | AK056473 | 0.0067221 | -1.21 | -1.166 | -1.89 |
| AK094929 | cDNA FLJ37610 fis, clone BRCOC2011398. [AK094929] | AK094929 | 0.0011182 | 1.6909 | 5.2621 | 1.1106 |
| AK127395 | cDNA FLJ45486 fis, clone BRTHA2002726 [AK127395] | AK127395 | 0.0278247 | 1.0793 | -1.465 | -1.758 |
| AK127770 | cDNA FLJ45871 fis, clone OCBBF3005597, highly similar to High-affinity cGMP-specific 3',5'-cyclic phosphodiesterase 9A (EC 3.1.4.17) [AK127770] | AK127770 | 0.0176348 | 1.0952 | 1.603 | -5.442 |
| CENPQ | centromere protein Q (CENPQ) | NM_018132 | 7.47E-04 | -1.406 | -1.293 | -2.244 |
| CEP164 | centrosomal protein 164kDa (CEP164) | NM_014956 | 0.0263423 | 1.2313 | 1.063 | 1.6472 |
| CEP27 | centrosomal protein 27kDa (CEP27) | NM_018097 | 0.0385485 | -1.254 | -1.135 | -1.59 |
| NM_148888 | chemokine (C-C motif) ligand 25 (CCL25), transcript variant 2 | NM_148888 | 0.0411149 | 1.3747 | 1.1364 | 1.2067 |
| NM_006090 | choline/ethanolaminephosphotransferase (CEPT1) | NM_006090 | 0.0490345 | 1.086 | 1.124 | 1.1818 |
| CHD1L | chromodomain helicase DNA binding protein 1-like (CHD1L) | NM_004284 | 0.0107275 | 1.0517 | 1.4299 | 1.5348 |
| C1orf103 | chromosome 1 open reading frame 103 (C1orf103), transcript variant 1 | NM_018372 | 0.0420578 | -1.213 | -1.012 | -1.383 |
| C1orf112 | chromosome 1 open reading frame 112 (C1orf112) | NM_018186 | 0.00783 | -1.197 | -1.054 | -2.28 |
| NM_001002292 | chromosome 1 open reading frame 139 (C1orf139), transcript variant 2 | NM_001002292 | 3.92E-04 | 1.0164 | -1.22 | -9.259 |
| C1orf27 | chromosome 1 open reading frame 27 (C1orf27) | NM_017847 | 0.0460511 | -1.242 | -1.094 | -1.46 |
| C1orf35 | chromosome 1 open reading frame 35 (C1orf35) | NM_024319 | 0.0019609 | 1.3554 | 1.1721 | 1.45 |
| C1orf74 | chromosome 1 open reading frame 74 (C1orf74) | NM_152485 | 0.0486447 | -1.389 | -1.088 | -1.514 |
| C1orf91 | chromosome 1 open reading frame 91 (C1orf91) | NM_019118 | 0.0012038 | 1.6597 | 1.5811 | 1.4265 |
| C1orf93 | chromosome 1 open reading frame 93 (C1orf93) | NM_152371 | 0.0075632 | 1.7728 | 1.0981 | 1.4138 |
| C10orf107 | chromosome 10 open reading frame 107 (C10orf107) | NM_173554 | 0.0038743 | 1.7875 | 1.4467 | 1.1247 |
| C10orf28 | chromosome 10 open reading frame 28 (C10orf28) | NM_014472 | 0.0028488 | 1.2924 | 1.2208 | 1.1163 |
| C10orf32 | chromosome 10 open reading frame 32 (C10orf32) | NM_144591 | 0.0409073 | -1.979 | -1.113 | -1.508 |
| ZCCHC24 | chromosome 10 open reading frame 56 (C10orf56) | NM_153367 | 0.0497498 | 1.4218 | 1.1354 | 1.0917 |
| C11orf35 | chromosome 11 open reading frame 35 (C11orf35) | NM_173573 | 0.0088362 | 1.1267 | 1.2345 | 2.4753 |
| C11orf49 | chromosome 11 open reading frame 49 (C11orf49), transcript variant 1 | NM_001003676 | 0.0121236 | 1.2932 | 1.2163 | 1.3312 |
| C11orf49 | chromosome 11 open reading frame 49 (C11orf49), transcript variant 1 | NM_001003676 | 0.015001 | 1.3749 | 1.1973 | 1.3029 |
| C11orf49 | chromosome 11 open reading frame 49 (C11orf49), transcript variant 1 | NM_001003676 | 0.0072069 | 1.3367 | 1.1789 | 1.3575 |
| C12orf29 | chromosome 12 open reading frame 29 (C12orf29) | NM_001009894 | 0.0453557 | -1.348 | -1.069 | -1.58 |
| C12orf35 | chromosome 12 open reading frame 35 (C12orf35) | NM_018169 | 0.0364355 | -1.512 | -1.151 | -1.995 |
| TCTN2 | chromosome 12 open reading frame 38 (C12orf38) | NM_024809 | 0.0149482 | -1.008 | 1.0783 | -5.037 |
| NM_018185 | chromosome 13 open reading frame 17 (C13orf17), transcript variant 1 | NM_018185 | 0.046721 | 1.2409 | 1.2153 | 1.8818 |
| C13orf33 | chromosome 13 open reading frame 33 (C13orf33) | NM_032849 | 0.006215 | -3.587 | -2.664 | 1.062 |
| C14orf101 | chromosome 14 open reading frame 101 (C14orf101) | NM_017799 | 0.0435868 | 1.1714 | 1.177 | 1.2726 |
| C14orf159 | chromosome 14 open reading frame 159 (C14orf159) | NM_024952 | 5.13E-04 | 1.5838 | 1.4077 | 1.8959 |
| C14orf45 | chromosome 14 open reading frame 45 (C14orf45) | NM_025057 | 0.0483064 | -2.087 | -1.268 | -1.655 |
| C15orf27 | chromosome 15 open reading frame 27 (C15orf27) | NM_152335 | 0.0159274 | -2.03 | -1.119 | -1.589 |
| C16orf42 | chromosome 16 open reading frame 42 (C16orf42) | NM_001001410 | 0.0096714 | 1.2123 | 1.1431 | 1.3773 |
| C16orf48 | chromosome 16 open reading frame 48 (C16orf48) | NM_032140 | 0.031655 | 1.1009 | 1.4216 | 1.4427 |
| C16orf63 | chromosome 16 open reading frame 63 (C16orf63) | NM_144600 | 0.0299304 | -1.214 | -1.263 | -1.215 |
| C17orf42 | chromosome 17 open reading frame 42 (C17orf42) | NM_024683 | 0.00283 | 1.1257 | 1.3225 | 1.3132 |
| C17orf56 | chromosome 17 open reading frame 56 (C17orf56) | NM_144679 | 0.0038449 | 1.2102 | 1.1031 | 2.3433 |
| C18orf8 | chromosome 18 open reading frame 8 (C18orf8) | NM_013326 | 0.049593 | 1.1707 | 1.1442 | 1.253 |
| NM_021185 | chromosome 19 open reading frame 15 (C19orf15) | NM_021185 | 0.0189242 | 1.0542 | 1.6454 | 1.1118 |
| C19orf23 | chromosome 19 open reading frame 23 (C19orf23) | NM_152480 | 0.0206942 | 1.7395 | 1.2527 | 1.0453 |
| C19orf44 | chromosome 19 open reading frame 44 (C19orf44) | NM_032207 | 1.10E-05 | 1.2998 | 1.3966 | 2.476 |
| C19orf47 | chromosome 19 open reading frame 47 (C19orf47) | NM_178830 | 0.0413656 | 1.0955 | 1.083 | 1.332 |
| C2orf16 | chromosome 2 open reading frame 16 (C2orf16) | NM_032266 | 3.01E-05 | 1.8212 | 1.261 | 4.5575 |
| NM_006462 | chromosome 20 open reading frame 18 (C20orf18), transcript variant 1 | NM_006462 | 0.0024919 | 1.0375 | 1.2273 | 2.2144 |
| C20orf26 | chromosome 20 open reading frame 26 (C20orf26) | NM_015585 | 0.0232108 | 3.1169 | 1.3539 | 1.4147 |
| NM_018478 | chromosome 20 open reading frame 35 (C20orf35) | NM_018478 | 0.0322676 | 1.1634 | 1.3003 | 1.4755 |
| NM_194309 | chromosome 21 open reading frame 125 (C21orf125) | NM_194309 | 0.028822 | 1.7545 | 1.3561 | 2.1976 |
| C22orf13 | chromosome 22 open reading frame 13 (C22orf13) | NM_031444 | 0.0292426 | 1.2017 | 1.1264 | 1.1895 |
| C3orf25 | chromosome 3 open reading frame 25 (C3orf25) | NM_207307 | 0.0153013 | 1.5016 | 1.3118 | 2.0294 |
| C4orf34 | chromosome 4 open reading frame 34 (C4orf34) | NM_174921 | 0.0308506 | -1.246 | -1.18 | -1.767 |
| C4orf8 | chromosome 4 open reading frame 8 (C4orf8) | NM_003704 | 0.001713 | 1.243 | 1.2887 | 1.5179 |
| C5orf15 | chromosome 5 open reading frame 15 (C5orf15) | NM_020199 | 0.0097178 | -1.393 | -1.375 | -1.687 |
| C5orf23 | chromosome 5 open reading frame 23 (C5orf23) | NM_024563 | 0.0147533 | 6.0809 | 1.0726 | 1.3014 |
| C5orf24 | chromosome 5 open reading frame 24 (C5orf24) | NM_152409 | 0.0124578 | -1.374 | -1.006 | -1.461 |
| C5orf25 | chromosome 5 open reading frame 25 (C5orf25) | NM_198567 | 0.0287508 | 1.1729 | 1.1334 | 1.2892 |
| C5orf35 | chromosome 5 open reading frame 35 (C5orf35) | NM_153706 | 0.004563 | -1.109 | -1.237 | -1.774 |
| C5orf38 | chromosome 5 open reading frame 38 (C5orf38) | NM_178569 | 0.0301383 | -1.313 | -1.29 | -1.833 |
| C6orf136 | chromosome 6 open reading frame 136 (C6orf136) | NM_145029 | 0.0484778 | 1.0328 | 1.1513 | 1.449 |
| C6orf173 | chromosome 6 open reading frame 173 (C6orf173) | NM_001012507 | 0.0103241 | -1.169 | -1.169 | -4.92 |
| C6orf35 | chromosome 6 open reading frame 35 (C6orf35) | NM_018452 | 0.0249419 | -1.402 | 1.2521 | -3.081 |
| C7orf36 | chromosome 7 open reading frame 36 (C7orf36) | NM_020192 | 0.0014424 | -1.365 | -1.01 | -1.407 |
| C7orf41 | chromosome 7 open reading frame 41 (C7orf41) | NM_152793 | 0.0154729 | 2.5621 | 1.4246 | 1.566 |
| C8orf22 | chromosome 8 open reading frame 22 (C8orf22) | NM_001007176 | 0.0421192 | 1.7217 | -1.146 | 3.1708 |
| C8orf31 | chromosome 8 open reading frame 31 (C8orf31) | NM_173687 | 0.032501 | 1.7721 | 1.2059 | -1.132 |
| C8orf40 | chromosome 8 open reading frame 40 (C8orf40) | NM_138436 | 0.0160068 | -1.12 | -1.065 | -2.047 |
| FAM164A | chromosome 8 open reading frame 70 (C8orf70) | NM_016010 | 0.0109117 | 1.1062 | 1.0695 | 1.4792 |
| C9orf164 | chromosome 9 open reading frame 164 (C9orf164) | NM_182635 | 0.0188401 | 1.6712 | -1.253 | 2.5019 |
| C9orf40 | chromosome 9 open reading frame 40 (C9orf40) | NM_017998 | 0.0394643 | 1.0343 | -1.351 | -1.218 |
| C9orf43 | chromosome 9 open reading frame 43 (C9orf43) | NM_152786 | 0.0014494 | 2.1659 | 1.6569 | 2.4094 |
| C9orf6 | chromosome 9 open reading frame 6 (C9orf6) | NM_017832 | 0.0090476 | 1.3107 | 1.2751 | 1.2766 |
| NM_025091 | chromosome X and Y open reading frame 2 (CXYorf2) | NM_025091 | 0.0102943 | 1.9161 | 1.492 | 1.2578 |
| NM_032179 | cleavage and polyadenylation specific factor 3-like (CPSF3L) | NM_032179 | 0.0101633 | 1.1191 | 1.2466 | 1.4902 |
| CCDC107 | coiled-coil domain containing 107 (CCDC107) | NM_174923 | 0.0303512 | 1.3761 | 1.4887 | 1.3388 |
| CCDC114 | coiled-coil domain containing 114 (CCDC114) | NM_144577 | 0.0343117 | 1.5265 | 1.8432 | 1.3785 |
| CCDC124 | coiled-coil domain containing 124 (CCDC124) | NM_138442 | 0.0190848 | 1.0378 | 1.047 | 1.549 |
| CCDC24 | coiled-coil domain containing 24 (CCDC24) | NM_152499 | 0.0430239 | 1.4634 | 1.22 | 1.0666 |
| CCDC46 | coiled-coil domain containing 46 (CCDC46), transcript variant 1 | NM_145036 | 9.33E-04 | -1.747 | -1.365 | -1.964 |
| CCDC81 | coiled-coil domain containing 81 (CCDC81) | NM_021827 | 0.0049824 | -1.534 | -3.272 | -4.913 |
| INO80E | coiled-coil domain containing 95 (CCDC95) | NM_173618 | 0.0269783 | 1.0479 | 1.1135 | 1.2589 |
| CCDC97 | coiled-coil domain containing 97 (CCDC97) | NM_052848 | 0.0127462 | 1.2507 | 1.0457 | 1.2396 |
| NM_000088 | collagen, type I, alpha 1 (COL1A1) | NM_000088 | 0.0114976 | -2.074 | -1.087 | -1.824 |
| NM_005677 | collagen-like tail subunit (single strand of homotrimer) of asymmetric acetylcholinesterase (COLQ), transcript variant I | NM_005677 | 0.0385859 | 1.5693 | 1.3942 | 1.0291 |
| CFHR2 | complement factor H-related 2 (CFHR2) | NM_005666 | 2.07E-04 | -1.209 | -1.032 | -11.38 |
| CFHR3 | complement factor H-related 3 (CFHR3) | NM_021023 | 0.0463442 | 2.0313 | 1.9346 | -1.015 |
| COPS4 | COP9 constitutive photomorphogenic homolog subunit 4 (Arabidopsis) (COPS4) | NM_016129 | 0.0109311 | -1.251 | 1.0182 | -1.641 |
| CORO7 | coronin 7 (CORO7) | NM_024535 | 0.0450485 | 1.0582 | -1.014 | 1.6067 |
| NM_001328 | C-terminal binding protein 1 (CTBP1) | NM_001328 | 0.0015415 | 1.2039 | 1.2655 | 1.3602 |
| CTTNBP2NL | CTTNBP2 N-terminal like (CTTNBP2NL) | NM_018704 | 0.0440268 | -1.552 | -1.147 | -1.385 |
| NM_198943 | CXYorf1-related protein (MGC52000) | NM_198943 | 0.0310809 | 1.1123 | 1.1144 | 1.6009 |
| CCNY | cyclin Y (CCNY), transcript variant 1 | NM_145012 | 0.0080144 | 1.1069 | 1.1303 | 1.4344 |
| NM_003674 | cyclin-dependent kinase (CDC2-like) 10 (CDK10), transcript variant 1 | NM_003674 | 0.0052882 | 1.2898 | 1.2223 | 2.3096 |
| NM_003674 | cyclin-dependent kinase (CDC2-like) 10 (CDK10), transcript variant 1 | NM_003674 | 0.0070012 | 1.247 | 1.2259 | 2.2033 |
| CST9L | cystatin 9-like (mouse) (CST9L) | NM_080610 | 0.0085729 | 2.9286 | 1.4646 | -1.695 |
| CYHR1 | cysteine/histidine-rich 1 (CYHR1) | NM_032687 | 0.0030687 | 1.3611 | 1.184 | 1.5787 |
| COX7B | cytochrome c oxidase subunit VIIb (COX7B), nuclear gene encoding mitochondrial protein | NM_001866 | 8.03E-06 | -1.283 | 1.1389 | -15.91 |
| CRLF3 | cytokine receptor-like factor 3 (CRLF3) | NM_015986 | 0.0278721 | -1.424 | -1.427 | -1.002 |
| DB566244 | DB566244 RIKEN full-length enriched human cDNA library, hypothalamus cDNA clone H033057I09 3' | DB566244 | 0.0227847 | -1.132 | -1.055 | -1.391 |
| OSTC | DC2 protein (DC2) | NM_021227 | 0.0064319 | -1.228 | -1.127 | -1.843 |
| DDX3Y | DEAD (Asp-Glu-Ala-Asp) box polypeptide 3, Y-linked (DDX3Y) | NM_004660 | 4.78E-06 | -1.356 | -1.134 | -9.688 |
| DDX42 | DEAD (Asp-Glu-Ala-Asp) box polypeptide 42 (DDX42), transcript variant 1 | NM_007372 | 0.0157816 | 1.1781 | 1.13 | 1.4129 |
| DIS3L2 | DIS3 mitotic control homolog (S. cerevisiae)-like 2 (DIS3L2) | NM_152383 | 0.0049341 | 1.0212 | 1.2183 | 1.3187 |
| NM_015492 | DKFZP434H132 protein (DKFZP434H132) | NM_015492 | 0.0018922 | 1.2535 | 1.3188 | 1.5401 |
| NM_005690 | dynamin 1-like (DNM1L), transcript variant 3 | NM_005690 | 0.0383864 | -1.344 | -1.01 | -1.408 |
| NM_006874 | E74-like factor 2 (ets domain transcription factor) (ELF2), transcript variant 2 | NM_006874 | 0.037584 | -1.1 | -1.058 | -1.417 |
| EFCAB4A | EF-hand calcium binding domain 4A (EFCAB4A) | NM_173584 | 0.007527 | 1.5356 | 1.0987 | 1.6157 |
| NM_022759 | endo-beta-N-acetylglucosaminidase (FLJ21865) | NM_022759 | 0.0296228 | 1.0535 | 1.2825 | 1.6522 |
| NM_003792 | endothelial differentiation-related factor 1 (EDF1), transcript variant alpha | NM_003792 | 0.0224626 | 1.1673 | 1.1776 | 1.1483 |
| EDC3 | enhancer of mRNA decapping 3 homolog (S. cerevisiae) (EDC3) | NM_025083 | 3.37E-04 | 1.0873 | 1.1872 | 1.8513 |
| ESPNL | espin-like (ESPNL) | NM_194312 | 0.018221 | 1.8906 | 1.3664 | 1.2711 |
| ETAA1 | Ewing's tumor-associated antigen 1 (ETAA1) | NM_019002 | 0.0311013 | 1.3288 | 1.312 | -1.017 |
| NM_130398 | exonuclease 1 (EXO1), transcript variant 2 | NM_130398 | 0.012798 | -1.559 | -2.34 | -1.845 |
| NM_130398 | exonuclease 1 (EXO1), transcript variant 2 | NM_130398 | 0.0223119 | -1.348 | -2.435 | -1.946 |
| FAM105B | family with sequence similarity 105, member B (FAM105B) | NM_138348 | 0.0128291 | 1.2635 | 1.1276 | 1.4891 |
| FAM107B | family with sequence similarity 107, member B (FAM107B) | NM_031453 | 0.0203987 | -1.227 | 1.1223 | -1.828 |
| FAM109A | family with sequence similarity 109, member A (FAM109A) | NM_144671 | 0.0142326 | 2.0235 | 1.2516 | 1.0126 |
| GTSF1 | family with sequence similarity 112, member B (FAM112B) | NM_144594 | 0.0170403 | 3.6633 | 4.654 | 2.4872 |
| FAM123B | family with sequence similarity 123B (FAM123B) | NM_152424 | 0.0340243 | 1.1266 | 1.1594 | 1.5802 |
| FAM38A | family with sequence similarity 38, member A (FAM38A) | NM_014745 | 0.0255899 | 1.3971 | 1.1654 | 1.5371 |
| FAM45B | family with sequence similarity 45, member B (FAM45B) | NM_018472 | 0.0219179 | -1.282 | -1.085 | -2.777 |
| FAM62B | family with sequence similarity 62 (C2 domain containing) member B (FAM62B) | NM_020728 | 0.0327857 | 1.0532 | 1.2112 | 1.6225 |
| FAM62A | family with sequence similarity 62 (C2 domain containing), member A (FAM62A) | NM_015292 | 0.0140307 | 1.3098 | 1.0719 | 1.1284 |
| FAM64A | family with sequence similarity 64, member A (FAM64A) | NM_019013 | 0.0393133 | -2.219 | -1.779 | -1.264 |
| FAM73B | family with sequence similarity 73, member B (FAM73B) | NM_032809 | 0.029224 | 1.2442 | 1.2393 | 1.9195 |
| FAM82B | family with sequence similarity 82, member B (FAM82B) | NM_016033 | 0.0059907 | -1.15 | -1.444 | -1.492 |
| FAM98C | family with sequence similarity 98, member C (FAM98C) | NM_174905 | 0.0229354 | 1.3756 | 1.0189 | 1.3117 |
| NM_178226 | F-box and WD-40 domain protein 5 (FBXW5), transcript variant 3 | NM_178226 | 0.0023623 | 1.1276 | 1.3051 | 1.6505 |
| NM_032807 | F-box protein, helicase, 18 (FBXO18), transcript variant 1 | NM_032807 | 2.89E-04 | -1.075 | -1.015 | -11.24 |
| NM_032807 | F-box protein, helicase, 18 (FBXO18), transcript variant 1 | NM_032807 | 0.0011729 | -1.523 | -1.14 | -10.34 |
| FRMD1 | FERM domain containing 1 (FRMD1) | NM_024919 | 6.85E-04 | 2.1257 | 1.0651 | 1.5248 |
| FRMD6 | FERM domain containing 6 (FRMD6), transcript variant 2 | NM_152330 | 0.0412183 | -1.532 | -1.249 | -1.125 |
| FBRS | fibrosin 1 (FBS1) | NM_022452 | 0.0094513 | -1.016 | 1.0945 | 2.2026 |
| FSIP2 | fibrous sheath interacting protein 2 (FSIP2) | NM_173651 | 0.0271153 | -1.257 | -1.008 | -2.643 |
| NM_015839 | ficolin (collagen/fibrinogen domain containing lectin) 2 (hucolin) (FCN2), transcript variant SV3 | NM_015839 | 0.0314214 | 1.5486 | 1.1698 | 1.6509 |
| PATL1 | FLJ36874 protein (FLJ36874) | NM_152716 | 0.0100752 | 1.2147 | 1.1001 | 1.4173 |
| NM_207488 | FLJ42393 protein (FLJ42393) | NM_207488 | 0.0342924 | 1.2142 | 1.5089 | 1.2214 |
| C15orf52 | FLJ43339 protein (FLJ43339) | NM_207380 | 0.0057803 | 1.4562 | 1.2702 | 2.0944 |
| FXR2 | fragile X mental retardation, autosomal homolog 2 (FXR2) | NM_004860 | 0.0154828 | 1.1223 | 1.0976 | 1.3522 |
| CR601067 | full-length cDNA clone CS0DC005YL10 of Neuroblastoma Cot 25-normalized of (human) [CR601067] | CR601067 | 0.0447735 | -1.624 | -1.993 | 1.2142 |
| CR603105 | full-length cDNA clone CS0DF006YN22 of Fetal brain of (human) [CR603105] | CR603105 | 9.80E-04 | -1.284 | -1.155 | -12.21 |
| FUNDC2 | FUN14 domain containing 2 (FUNDC2) | NM_023934 | 0.0010691 | -1.439 | 1.0629 | -4.284 |
| NM_001010850 | fusion (involved in t(12;16) in malignant liposarcoma) (FUS), transcript variant 2 | NM_001010850 | 0.0167284 | 1.0075 | 1.3004 | 1.3558 |
| NM_198827 | G protein-coupled receptor 133 (GPR133) | NM_198827 | 0.0045968 | 1.4352 | 1.1178 | 2.0818 |
| GPR172A | G protein-coupled receptor 172A (GPR172A) | NM_024531 | 0.0336571 | 1.1214 | 1.1172 | 1.2855 |
| GDAP2 | ganglioside induced differentiation associated protein 2 (GDAP2) | NM_017686 | 0.0047203 | -1.741 | 1.1302 | -2.044 |
| QRICH2 | glutamine rich 2 (QRICH2) | NM_032134 | 0.0056823 | 1.6698 | 1.7535 | 2.6173 |
| GSTK1 | glutathione S-transferase kappa 1 (GSTK1) | NM_015917 | 0.0460953 | 1.0339 | 1.3629 | 1.3202 |
| NM_000850 | glutathione S-transferase M4 (GSTM4), transcript variant 1 | NM_000850 | 0.0013888 | 1.225 | 1.3351 | 2.7746 |
| GLYATL1 | glycine-N-acyltransferase-like 1 (GLYATL1) | NM_080661 | 0.0461521 | 1.4822 | 1.0365 | 1.9802 |
| NM_013365 | golgi associated, gamma adaptin ear containing, ARF binding protein 1 (GGA1) | NM_013365 | 0.0272223 | -1.032 | 1.1502 | 1.8555 |
| GOLGA1 | golgi autoantigen, golgin subfamily a, 1 (GOLGA1) | NM_002077 | 0.0268689 | 1.1982 | 1.1313 | 1.1899 |
| CAPRIN1 | GPI-anchored membrane protein 1 (GPIAP1), transcript variant 1 | NM_005898 | 0.0324768 | -1.273 | -1.118 | -1.179 |
| GTPBP2 | GTP binding protein 2 (GTPBP2) | NM_019096 | 0.0408473 | -1.085 | -1.131 | 2.4611 |
| NM_005272 | guanine nucleotide binding protein (G protein), alpha transducing activity polypeptide 2 (GNAT2) | NM_005272 | 0.0041503 | 1.0384 | 1.0507 | -6.505 |
| H3F3A | H3 histone, family 3A (H3F3A) | NM_002107 | 0.006486 | -1.03 | 1.0735 | -1.731 |
| NM_001001520 | hepatoma-derived growth factor-related protein 2 (HDGF2), transcript variant 1 | NM_001001520 | 9.32E-04 | 1.3401 | 1.2478 | 1.7609 |
| HPCAL1 | hippocalcin-like 1 (HPCAL1), transcript variant 2 | NM_134421 | 0.0429776 | 1.2353 | 1.6289 | -1.055 |
| HIST1H3I | histone cluster 1, H3i (HIST1H3I) | NM_003533 | 0.0467806 | -1.699 | -1.643 | -2.181 |
| NM_001535 | HMT1 hnRNP methyltransferase-like 1 (S. cerevisiae) (HRMT1L1), transcript variant 2 | NM_001535 | 0.0461123 | 1.0514 | 1.1225 | 1.38 |
| WDR91 | HSPC049 protein (HSPC049) | NM_014149 | 0.0136528 | 1.3512 | 1.1526 | 1.378 |
| NM_207112 | hydroxyacylglutathione hydrolase-like (HAGHL), transcript variant 1 | NM_207112 | 0.0258072 | -1.075 | 1.2271 | 2.3865 |
| NM_207112 | hydroxyacylglutathione hydrolase-like (HAGHL), transcript variant 1 | NM_207112 | 0.0214048 | 1.0097 | 1.216 | 2.4635 |
| NM_139016 | hypothetical gene LOC128439 (LOC128439) | NM_139016 | 0.0433933 | 1.4472 | 1.2388 | 1.2194 |
| FLYWCH2 | hypothetical protein BC014089 (LOC114984) | NM_138439 | 1.07E-04 | -2.246 | 1.0823 | -7.99 |
| C7orf64 | hypothetical protein DKFZp564O0523 (DKFZP564O0523) | NM_032120 | 0.012343 | -1.691 | 1.0638 | -5.179 |
| KIAA1310 | hypothetical protein FLJ10081 (FLJ10081) | NM_017991 | 0.0392555 | 1.1672 | 1.0486 | 1.6215 |
| ARGLU1 | hypothetical protein FLJ10154 (FLJ10154) | NM_018011 | 0.0152516 | 1.2591 | 1.2042 | 1.4374 |
| NM_019057 | hypothetical protein FLJ10404 (FLJ10404) | NM_019057 | 0.0010911 | 1.4206 | 1.1855 | 2.7476 |
| CPPED1 | hypothetical protein FLJ11151 (FLJ11151) | NM_018340 | 0.0169207 | -1.62 | -1.217 | -1.105 |
| CCDC142 | hypothetical protein FLJ14397 (FLJ14397) | NM_032779 | 0.0033793 | 1.5477 | 1.2599 | 1.0254 |
| NM_017751 | hypothetical protein FLJ20297 (FLJ20297) | NM_017751 | 0.0033909 | 1.326 | 1.1867 | 1.5362 |
| GOLSYN | hypothetical protein FLJ20366 (FLJ20366) | NM_017786 | 2.29E-05 | 1.0557 | 6.1311 | 1.4584 |
| TTC38 | hypothetical protein FLJ20699 (FLJ20699) | NM_017931 | 0.0070897 | 1.6618 | 1.1091 | 1.5455 |
| NM_017939 | hypothetical protein FLJ20718 (FLJ20718) | NM_017939 | 1.23E-04 | -1.465 | 1.0246 | -4.447 |
| RPAP3 | hypothetical protein FLJ21908 (FLJ21908) | NM_024604 | 0.0439941 | -1.294 | -1.25 | -1.397 |
| NM_152675 | hypothetical protein FLJ23754 (FLJ23754) | NM_152675 | 0.0282486 | 1.3723 | 1.4095 | 1.3226 |
| NM_144719 | hypothetical protein FLJ25467 (FLJ25467) | NM_144719 | 0.0115283 | 2.2933 | 2.0999 | -1.282 |
| NM_173795 | hypothetical protein FLJ32096 (FLJ32096) | NM_173795 | 0.0029434 | 2.0701 | 1.2723 | 1.6279 |
| MFSD6L | hypothetical protein FLJ35773 (FLJ35773) | NM_152599 | 0.0457192 | 1.8403 | 1.4544 | 1.1093 |
| NM_153233 | hypothetical protein FLJ36445 (FLJ36445) | NM_153233 | 0.0225703 | 1.5956 | 1.366 | 1.1587 |
| CES8 | hypothetical protein FLJ37464 (FLJ37464) | NM_173815 | 0.0499467 | 1.2972 | 1.4514 | 1.535 |
| NM_194299 | hypothetical protein LOC221711 (LOC221711) | NM_194299 | 0.0240512 | 2.392 | 1.0789 | 1.6755 |
| IFFO1 | hypothetical protein LOC25900 (HOM-TES-103), transcript variant 3 | NM_080731 | 0.0067578 | 1.0045 | 1.288 | 2.0801 |
| C19orf68 | hypothetical protein LOC374920 (LOC374920) | NM_199341 | 0.029367 | 1.255 | 1.1846 | 1.4068 |
| NM_032921 | hypothetical protein MGC15875 (MGC15875) | NM_032921 | 0.0115964 | 1.316 | -1.001 | 1.5291 |
| NM_032921 | hypothetical protein MGC15875 (MGC15875) | NM_032921 | 0.0124861 | 1.2325 | 1.0186 | 1.5131 |
| C7orf62 | hypothetical protein MGC26647 (MGC26647) | NM_152706 | 0.0337328 | 2.8282 | 1.4664 | -1.326 |
| NM_031298 | hypothetical protein MGC2963 (MGC2963) | NM_031298 | 0.0076692 | -1.065 | 1.0487 | -1.636 |
| TCTEX1D2 | hypothetical protein MGC33212 (MGC33212) | NM_152773 | 0.0448497 | 1.0325 | 1.2651 | -7.783 |
| C12orf72 | hypothetical protein MGC50559 (MGC50559) | NM_173802 | 7.82E-04 | -1.88 | -1.62 | -1.939 |
| CMC1 | hypothetical protein MGC61571 (MGC61571) | NM_182523 | 0.0124847 | -1.086 | -1.021 | -1.857 |
| RILPL2 | hypothetical protein MGC7036 (MGC7036) | NM_145058 | 0.0245925 | 1.184 | 1.1573 | 1.416 |
| CN644731 | ILLUMIGEN_MCQ_10781 Katze_MMPL2 Macaca mulatta cDNA clone IBIUW:9547 5' similar to Bases 5 to 628 highly similar to human NDUFB9 (Hs.15977) | CN644731 | 0.0491065 | -1.083 | 1.1377 | -1.639 |
| CK230740 | ILLUMIGEN_MCQ_1100 Katze_MMPL2 Macaca mulatta cDNA 5' similar to human PSG5 (Hs.251850) | CK230740 | 0.0073279 | -1.114 | -1.124 | -1.381 |
| CK231052 | ILLUMIGEN_MCQ_1448 Katze_MMPL2 Macaca mulatta cDNA 5' similar to human Unigene Hs.500464 | CK231052 | 0.0428543 | -1.027 | -1.285 | -1.638 |
| CK231384 | ILLUMIGEN_MCQ_2133 Katze_MMLG Macaca mulatta cDNA 5' similar to human TFPI2 (Hs.438231) | CK231384 | 0.0394968 | -1.144 | -1.002 | -1.649 |
| CN806006 | ILLUMIGEN_MCQ_23806 Katze_MMPL1 Macaca mulatta cDNA clone IBIUW:15932 5' similar to Bases 13 to 893 highly similar to human RNP24 (Hs.75914) | CN806006 | 0.0224136 | -1.181 | -1.107 | -1.397 |
| DR771832 | ILLUMIGEN_MCQ_24026 Katze_MMSP Macaca mulatta cDNA clone IBIUW:30296 5' similar to Bases 1 to 694 highly similar to human RSBN1 (Hs.486285) | DR771832 | 0.0060273 | -1.207 | -1.155 | -1.393 |
| CK231549 | ILLUMIGEN_MCQ_2449 Katze_MMLG Macaca mulatta cDNA 5' similar to human TFPI2 (Hs.438231) | CK231549 | 4.63E-05 | -1.126 | -1.157 | -5.174 |
| CK231551 | ILLUMIGEN_MCQ_2453 Katze_MMLG Macaca mulatta cDNA 5' similar to human TFPI2 (Hs.438231) | CK231551 | 0.0146216 | -1.36 | 1.2746 | -2.193 |
| CN802660 | ILLUMIGEN_MCQ_25016 Katze_MMBR Macaca mulatta cDNA clone IBIUW:11517 5' similar to Bases 1 to 791 highly similar to human SPRED1 (Hs.132804) | CN802660 | 0.0099857 | -1.4 | -1.063 | -1.483 |
| CN802660 | ILLUMIGEN_MCQ_25016 Katze_MMBR Macaca mulatta cDNA clone IBIUW:11517 5' similar to Bases 1 to 791 highly similar to human SPRED1 (Hs.132804) | CN802660 | 0.0305566 | -1.277 | -1.147 | -1.442 |
| CN645943 | ILLUMIGEN_MCQ_25163 Katze_MMBR Macaca mulatta cDNA clone IBIUW:10759 5' similar to Bases 18 to 487 highly similar to human ZHX3 (Hs.380133) | CN645943 | 0.0086764 | 1.2031 | 1.1394 | 1.76 |
| CN802671 | ILLUMIGEN_MCQ_25191 Katze_MMBR Macaca mulatta cDNA clone IBIUW:12556 5' similar to Bases 1 to 720 highly similar to human DKFZp667B1218 (Hs.151293) | CN802671 | 0.021401 | -1.546 | -1.201 | -1.178 |
| CN646039 | ILLUMIGEN_MCQ_25388 Katze_MMBR Macaca mulatta cDNA clone IBIUW:10855 5' similar to Bases 1 to 1036 highly similar to human ATP6V1G1 (Hs.90336) | CN646039 | 0.0275955 | -1.196 | 1.0092 | -1.342 |
| CN646048 | ILLUMIGEN_MCQ_25404 Katze_MMBR Macaca mulatta cDNA clone IBIUW:10864 5' similar to Bases 1 to 1047 highly similar to human FLJ10097 (Hs.184736) | CN646048 | 0.0460276 | 1.314 | 1.1657 | 1.3757 |
| CN646438 | ILLUMIGEN_MCQ_26202 Katze_MMBR Macaca mulatta cDNA clone IBIUW:8578 5' similar to Bases 1 to 503 highly similar to human APBB1 (Hs.378063) | CN646438 | 0.0066523 | 1.14 | 1.3253 | 1.4987 |
| CN646662 | ILLUMIGEN_MCQ_26776 Katze_MMBR Macaca mulatta cDNA clone IBIUW:8375 5' similar to Bases 1 to 808 highly similar to human RRP41 (Hs.343589) | CN646662 | 0.0184637 | 1.085 | 1.267 | 1.2388 |
| CN646671 | ILLUMIGEN_MCQ_26813 Katze_MMBR Macaca mulatta cDNA clone IBIUW:8352 5' similar to Bases 516 to 877 highly similar to human Unigene Hs.289044 | CN646671 | 0.0423004 | -1.734 | -1.117 | -1.35 |
| CN646819 | ILLUMIGEN_MCQ_27178 Katze_MMBR Macaca mulatta cDNA clone IBIUW:8212 5' similar to Bases 1 to 1032 highly similar to human PSMA4 (Hs.251531) | CN646819 | 0.0254488 | -1.362 | 1.0339 | -1.598 |
| CN647507 | ILLUMIGEN_MCQ_28626 Katze_MMPB Macaca mulatta cDNA clone IBIUW:7547 5' similar to Bases 62 to 959 highly similar to human NXT2 (Hs.25010) | CN647507 | 0.0391185 | -1.183 | -1.027 | -1.489 |
| CN802789 | ILLUMIGEN_MCQ_28768 Katze_MMPL2 Macaca mulatta cDNA clone IBIUW:13171 5' similar to Bases 1 to 526 highly similar to human ALS2CR17 (Hs.471145) | CN802789 | 0.0259581 | -1.594 | -1.149 | -2.531 |
| CN802789 | ILLUMIGEN_MCQ_28768 Katze_MMPL2 Macaca mulatta cDNA clone IBIUW:13171 5' similar to Bases 1 to 526 highly similar to human ALS2CR17 (Hs.471145) | CN802789 | 0.0057728 | -1.342 | -1.13 | -3.152 |
| CN647646 | ILLUMIGEN_MCQ_28879 Katze_MMPB Macaca mulatta cDNA clone IBIUW:7398 5' similar to Bases 1 to 917 highly similar to human ARHGEF11 (Hs.371602) | CN647646 | 0.0050926 | 1.1102 | 1.3377 | 1.721 |
| CN802796 | ILLUMIGEN_MCQ_29125 Katze_MMPL2 Macaca mulatta cDNA clone IBIUW:11348 5' similar to Bases 1 to 797 highly similar to human C14orf138 (Hs.390504) | CN802796 | 0.0320462 | -1.269 | -1.001 | -1.359 |
| CO645773 | ILLUMIGEN_MCQ_30118 Katze_MMPB Macaca mulatta cDNA clone IBIUW:22572 5' similar to Bases 1 to 42 highly similar to human RARRES3 (Hs.17466) | CO645773 | 0.0370491 | -3.538 | -2.022 | -1.475 |
| CN648422 | ILLUMIGEN_MCQ_30476 Katze_MMPB Macaca mulatta cDNA clone IBIUW:6624 5' similar to Bases 1 to 611 highly similar to human Unigene Hs.294035 | CN648422 | 0.0160543 | 1.4496 | 1.8946 | 1.7261 |
| CN648422 | ILLUMIGEN_MCQ_30476 Katze_MMPB Macaca mulatta cDNA clone IBIUW:6624 5' similar to Bases 1 to 611 highly similar to human Unigene Hs.294035 | CN648422 | 0.0302787 | 1.4787 | 1.6329 | 1.6017 |
| CN648648 | ILLUMIGEN_MCQ_31033 Katze_MMPB Macaca mulatta cDNA clone IBIUW:6402 5' similar to Bases 332 to 402 highly similar to human KIAA0676 (Hs.155829) | CN648648 | 3.85E-04 | 1.3101 | 1.3408 | 1.4925 |
| DV768384 | ILLUMIGEN_MCQ_32283 Katze_MMBR Macaca mulatta cDNA clone IBIUW:38694 5' similar to Bases 2 to 489 highly similar to human PAG (Hs.266175) | DV768384 | 0.0493748 | -1.228 | -1.901 | 1.1813 |
| CK232048 | ILLUMIGEN_MCQ_3236 Katze_MMPL2 Macaca mulatta cDNA 5' similar to human PSG5 (Hs.251850) | CK232048 | 0.0196901 | -1.673 | -1.145 | -3.927 |
| CN803327 | ILLUMIGEN_MCQ_32753 Katze_MMBR Macaca mulatta cDNA clone IBIUW:11351 5' similar to Bases 118 to 739 highly similar to human UBXD1 (Hs.435255) | CN803327 | 0.0063234 | 1.8372 | -1.03 | 1.4781 |
| CK232102 | ILLUMIGEN_MCQ_3292 Katze_MMPL2 Macaca mulatta cDNA 5' | CK232102 | 0.0158245 | -1.73 | 1.0277 | -1.276 |
| CK232102 | ILLUMIGEN_MCQ_3292 Katze_MMPL2 Macaca mulatta cDNA 5' | CK232102 | 0.0033816 | -1.599 | 1.0096 | -1.645 |
| CN804242 | ILLUMIGEN_MCQ_34318 Katze_MMPL1 Macaca mulatta cDNA clone IBIUW:11673 5' similar to Bases 174 to 470 highly similar to human FLJ42280 (Hs.333495) | CN804242 | 0.0164383 | -1.127 | -1.059 | -1.617 |
| CN804253 | ILLUMIGEN_MCQ_34333 Katze_MMPL1 Macaca mulatta cDNA clone IBIUW:13916 5' similar to Bases 1 to 292 highly similar to human PRO0659 (Hs.6451) | CN804253 | 0.0103916 | 1.1502 | 1.3481 | 1.7704 |
| CK232222 | ILLUMIGEN_MCQ_3441 Katze_MMPL2 Macaca mulatta cDNA 5' similar to human TFPI2 (Hs.438231) | CK232222 | 5.68E-04 | -1.486 | -2.102 | -2.899 |
| CK232222 | ILLUMIGEN_MCQ_3441 Katze_MMPL2 Macaca mulatta cDNA 5' similar to human TFPI2 (Hs.438231) | CK232222 | 0.0389478 | -1.334 | -2.34 | 1.1144 |
| CN804946 | ILLUMIGEN_MCQ_35249 Katze_MMPL1 Macaca mulatta cDNA clone IBIUW:11690 5' similar to Bases 180 to 614 highly similar to human Unigene Hs.5250 | CN804946 | 0.0114918 | -1.607 | 1.0265 | -1.332 |
| CO725491 | ILLUMIGEN_MCQ_35277 Katze_MMPL Macaca mulatta cDNA clone IBIUW:28183 5' similar to Bases 1 to 267 highly similar to human KNS2 (Hs.20107) | CO725491 | 0.0212246 | 1.0883 | 1.0849 | 1.3541 |
| CK232335 | ILLUMIGEN_MCQ_3574 Katze_MMPL2 Macaca mulatta cDNA 5' similar to human DKFZp761B1514 (Hs.277533) | CK232335 | 0.0099483 | -1.451 | -1.095 | -1.363 |
| CN801659 | ILLUMIGEN_MCQ_35872 Katze_MMPL1 Macaca mulatta cDNA clone IBIUW:14717 5' similar to Bases 417 to 900 highly similar to human CGI-115 (Hs.56043) | CN801659 | 0.0102564 | 1.2239 | 1.3151 | 1.4132 |
| CN805444 | ILLUMIGEN_MCQ_36605 Katze_MMBR Macaca mulatta cDNA clone IBIUW:13910 5' similar to Bases 14 to 989 highly similar to human Unigene Hs.162601 | CN805444 | 0.0226156 | -1.547 | -1.298 | -3.044 |
| CN802172 | ILLUMIGEN_MCQ_37772 Katze_MMBR Macaca mulatta cDNA clone IBIUW:15508 5' similar to Bases 2 to 934 highly similar to human DKFZp313A2432 (Hs.349096) | CN802172 | 0.0063905 | -1.708 | -1.585 | -1.225 |
| CO048985 | ILLUMIGEN_MCQ_38319 Katze_MMBR Macaca mulatta cDNA clone IBIUW:16091 5' similar to Bases 1 to 765 highly similar to human Unigene Hs.171939 | CO048985 | 0.0121788 | 1.0135 | 1.531 | 2.0307 |
| CO725791 | ILLUMIGEN_MCQ_38543 Katze_MMLV Macaca mulatta cDNA clone IBIUW:23402 5' similar to Bases 5 to 222 highly similar to human PXMP2 (Hs.430299) | CO725791 | 0.040376 | 1.1989 | 1.2596 | 1.05 |
| CO646479 | ILLUMIGEN_MCQ_39220 Katze_MMPB2 Macaca mulatta cDNA clone IBIUW:21594 5' similar to Bases 5 to 919 highly similar to human ABC1 (Hs.17230) | CO646479 | 0.0432475 | 1.0487 | 1.1027 | 1.7265 |
| CO647386 | ILLUMIGEN_MCQ_40418 Katze_MMPB2 Macaca mulatta cDNA clone IBIUW:21432 5' similar to Bases 185 to 778 highly similar to human CXCL2 (Hs.75765) | CO647386 | 0.0207766 | -1.03 | -3.165 | -1.632 |
| CO647411 | ILLUMIGEN_MCQ_40453 Katze_MMPB2 Macaca mulatta cDNA clone IBIUW:23989 5' similar to Bases 99 to 916 highly similar to human SPEC2 (Hs.5985) | CO647411 | 0.006682 | -1.45 | -1.277 | -1.072 |
| CO647467 | ILLUMIGEN_MCQ_40520 Katze_MMPB2 Macaca mulatta cDNA clone IBIUW:23282 5' similar to Bases 2 to 944 highly similar to human PAG (Hs.266175) | CO647467 | 0.0201326 | -1.399 | -2.072 | 1.1974 |
| CO647725 | ILLUMIGEN_MCQ_40874 Katze_MMPB2 Macaca mulatta cDNA clone IBIUW:22705 5' similar to Bases 4 to 962 highly similar to human RPE (Hs.282260) | CO647725 | 5.16E-04 | -1.969 | -1.176 | -1.345 |
| CO647725 | ILLUMIGEN_MCQ_40874 Katze_MMPB2 Macaca mulatta cDNA clone IBIUW:22705 5' similar to Bases 4 to 962 highly similar to human RPE (Hs.282260) | CO647725 | 1.96E-05 | -2.131 | -1.258 | -1.578 |
| CO647944 | ILLUMIGEN_MCQ_41166 Katze_MMPB2 Macaca mulatta cDNA clone IBIUW:21926 5' similar to Bases 5 to 810 highly similar to human PITRM1 (Hs.528300) | CO647944 | 0.0022032 | 1.0842 | 1.2351 | 1.6846 |
| CO648435 | ILLUMIGEN_MCQ_41807 Katze_MMPB2 Macaca mulatta cDNA clone IBIUW:23814 5' similar to Bases 5 to 938 highly similar to human SCOC (Hs.286013) | CO648435 | 0.0149903 | -1.288 | 1.0307 | -1.591 |
| CK230160 | ILLUMIGEN_MCQ_458 Katze_MMPL2 Macaca mulatta cDNA 5' similar to human PSG5 (Hs.251850) | CK230160 | 0.0375474 | -1.599 | -1.328 | -1.177 |
| CO581492 | ILLUMIGEN_MCQ_47149 Katze_MMJJ Macaca mulatta cDNA clone IBIUW:20898 5' similar to Bases 10 to 541 highly similar to human DDT (Hs.433902) | CO581492 | 0.0211263 | 1.7659 | 1.1484 | 1.3335 |
| CO581217 | ILLUMIGEN_MCQ_47520 Katze_MMJJ Macaca mulatta cDNA clone IBIUW:21011 5' similar to Bases 118 to 566 highly similar to human SDBCAG84 (Hs.169992) | CO581217 | 0.0382712 | 1.4159 | 1.111 | 1.1326 |
| CN641426 | ILLUMIGEN_MCQ_4806 Katze_MMPL2 Macaca mulatta cDNA clone IBIUW:5667 5' similar to Bases 271 to 753 highly similar to human MESDC2 (Hs.78871) | CN641426 | 0.0467052 | 1.0195 | -1.287 | -1.237 |
| CN641451 | ILLUMIGEN_MCQ_4838 Katze_MMPL2 Macaca mulatta cDNA clone IBIUW:5698 5' similar to Bases 1 to 1025 highly similar to human Unigene Hs.529772 | CN641451 | 0.0391728 | -1.562 | -1.152 | -1.875 |
| CO580464 | ILLUMIGEN_MCQ_48633 Katze_MMTE Macaca mulatta cDNA clone IBIUW:16662 5' similar to Bases 400 to 718 highly similar to human NDUFAB1 (Hs.5556) | CO580464 | 0.0347989 | -1.366 | 1.0586 | -1.69 |
| CO578964 | ILLUMIGEN_MCQ_50856 Katze_MMIL Macaca mulatta cDNA clone IBIUW:18015 5' similar to Bases 5 to 879 highly similar to human C9orf10OS (Hs.350364) | CO578964 | 0.0160808 | 1.2813 | 1.2915 | 1.3203 |
| CN641666 | ILLUMIGEN_MCQ_5148 Katze_MMBR Macaca mulatta cDNA clone IBIUW:5963 5' similar to Bases 345 to 909 highly similar to human Unigene Hs.530633 | CN641666 | 7.52E-04 | -2.317 | -1.066 | -6.44 |
| CN641686 | ILLUMIGEN_MCQ_5182 Katze_MMBR Macaca mulatta cDNA clone IBIUW:5989 5' similar to Bases 11 to 612 highly similar to human Unigene Hs.445038 | CN641686 | 0.013021 | 1.2137 | 1.489 | 1.311 |
| CN641686 | ILLUMIGEN_MCQ_5182 Katze_MMBR Macaca mulatta cDNA clone IBIUW:5989 5' similar to Bases 11 to 612 highly similar to human Unigene Hs.445038 | CN641686 | 0.0114205 | 1.2569 | 1.4975 | 1.1879 |
| CO774990 | ILLUMIGEN_MCQ_52168 Katze_MMIL Macaca mulatta cDNA clone IBIUW:29185 5' similar to Bases 42 to 707 highly similar to human MGC23909 (Hs.355606) | CO774990 | 0.0087958 | -1.341 | -1.076 | -1.487 |
| CN641710 | ILLUMIGEN_MCQ_5231 Katze_MMBR Macaca mulatta cDNA clone IBIUW:6025 5' similar to Bases 11 to 158 highly similar to human RPN2 (Hs.406532) | CN641710 | 0.0085742 | -1.567 | -1.065 | -1.832 |
| DR773623 | ILLUMIGEN_MCQ_56220 Katze_MMOV Macaca mulatta cDNA clone IBIUW:37350 5' similar to Bases 329 to 712 highly similar to human PIAS3 (Hs.435761) | DR773623 | 0.0165815 | 1.1462 | 1.0962 | 1.3811 |
| DR767858 | ILLUMIGEN_MCQ_60585 Katze_MMOV Macaca mulatta cDNA clone IBIUW:35758 5' similar to Bases 6 to 537 highly similar to human LZTS2 (Hs.523221) | DR767858 | 0.0330334 | 1.0597 | 1.136 | 1.9474 |
| CN642258 | ILLUMIGEN_MCQ_6183 Katze_MMBR Macaca mulatta cDNA clone IBIUW:4969 5' similar to Bases 1 to 1119 highly similar to human RNF11 (Hs.96334) | CN642258 | 0.0491212 | -1.24 | -1.124 | -1.364 |
| CN642598 | ILLUMIGEN_MCQ_6611 Katze_MMPL2 Macaca mulatta cDNA clone IBIUW:4616 5' similar to Bases 1 to 553 highly similar to human MRPS28 (Hs.55097) | CN642598 | 0.0176974 | -1.432 | -1.15 | -1.403 |
| CN642739 | ILLUMIGEN_MCQ_6790 Katze_MMPL2 Macaca mulatta cDNA clone IBIUW:4457 5' similar to Bases 354 to 806 highly similar to human Unigene Hs.291319 | CN642739 | 0.0271758 | -1.537 | -1.279 | -1.53 |
| DV769745 | ILLUMIGEN_MCQ_68759 Katze_MMTE Macaca mulatta cDNA clone IBIUW:40266 5' similar to Bases 5 to 572 highly similar to human POLR2J (Hs.533383) | DV769745 | 0.0310642 | -1.06 | -1.062 | -1.399 |
| CN802479 | ILLUMIGEN_MCQ_7198 Katze_MMPL2 Macaca mulatta cDNA clone IBIUW:4073 5' similar to Bases 1 to 816 highly similar to human NEK7 (Hs.24119) | CN802479 | 0.0095709 | -1.012 | -1.008 | -2.159 |
| CK230400 | ILLUMIGEN_MCQ_722 Katze_MMPL2 Macaca mulatta cDNA 5' | CK230400 | 0.0377208 | -1.562 | -1.419 | 1.0145 |
| CN643185 | ILLUMIGEN_MCQ_7373 Katze_MMBR Macaca mulatta cDNA clone IBIUW:3944 5' similar to Bases 475 to 877 highly similar to human Unigene Hs.117979 | CN643185 | 0.0165309 | -1.759 | -1.139 | -2.592 |
| CN643185 | ILLUMIGEN_MCQ_7373 Katze_MMBR Macaca mulatta cDNA clone IBIUW:3944 5' similar to Bases 475 to 877 highly similar to human Unigene Hs.117979 | CN643185 | 0.0038791 | -1.668 | -1.248 | -2.691 |
| CN643290 | ILLUMIGEN_MCQ_7610 Katze_MMBR Macaca mulatta cDNA clone IBIUW:3798 5' similar to Bases 1 to 954 highly similar to human LOC90799 (Hs.528712) | CN643290 | 0.0103956 | 1.1473 | 1.1345 | 1.2124 |
| CN643312 | ILLUMIGEN_MCQ_7714 Katze_MMBR Macaca mulatta cDNA clone IBIUW:3737 5' similar to Bases 1 to 1023 highly similar to human MGC29463 (Hs.4845) | CN643312 | 0.0431973 | 1.2685 | 1.0631 | 1.8117 |
| CN643612 | ILLUMIGEN_MCQ_8162 Katze_MMBR Macaca mulatta cDNA clone IBIUW:3364 5' similar to Bases 463 to 963 highly similar to human Unigene Hs.4241 | CN643612 | 0.0428528 | 1.7215 | 1.0825 | 1.7802 |
| CN643639 | ILLUMIGEN_MCQ_8235 Katze_MMBR Macaca mulatta cDNA clone IBIUW:3333 5' similar to Bases 1 to 682 highly similar to human Unigene Hs.513885 | CN643639 | 2.57E-05 | -1.711 | 1.1324 | -19.18 |
| CN643667 | ILLUMIGEN_MCQ_8320 Katze_MMBR Macaca mulatta cDNA clone IBIUW:3308 5' similar to Bases 1 to 1042 highly similar to human C5orf13 (Hs.508741) | CN643667 | 0.0453147 | -2.481 | -1.038 | -1.393 |
| CN643945 | ILLUMIGEN_MCQ_8982 Katze_MMBR Macaca mulatta cDNA clone IBIUW:2913 5' similar to Bases 1 to 922 highly similar to human KIAA0436 (Hs.110) | CN643945 | 0.0301482 | -1.842 | 1.1943 | -1.379 |
| CN644012 | ILLUMIGEN_MCQ_9292 Katze_MMBR Macaca mulatta cDNA clone IBIUW:8819 5' similar to Bases 1 to 744 highly similar to human TNPO3 (Hs.412527) | CN644012 | 0.0121125 | -1.074 | -1.026 | -1.423 |
| CN644224 | ILLUMIGEN_MCQ_9905 Katze_MMPL2 Macaca mulatta cDNA clone IBIUW:9031 5' similar to Bases 151 to 767 highly similar to human CGI-32 (Hs.16606) | CN644224 | 0.0359085 | -1.569 | -1.226 | -1.244 |
| CN644249 | ILLUMIGEN_MCQ_9972 Katze_MMPL2 Macaca mulatta cDNA clone IBIUW:9056 5' similar to Bases 1 to 253 highly similar to human FLJ12886 (Hs.10116) | CN644249 | 0.0330444 | -1.002 | 1.0826 | 1.8378 |
| CN644249 | ILLUMIGEN_MCQ_9972 Katze_MMPL2 Macaca mulatta cDNA clone IBIUW:9056 5' similar to Bases 1 to 253 highly similar to human FLJ12886 (Hs.10116) | CN644249 | 0.0227015 | -1.044 | 1.1628 | 1.8893 |
| NM_003669 | inactivation escape 1 (INE1) | NM_003669 | 0.0150247 | 1.5018 | 1.3866 | 1.8389 |
| INHBC | inhibin, beta C (INHBC) | NM_005538 | 0.044017 | 1.8205 | 1.2259 | 1.4998 |
| IMPAD1 | inositol monophosphatase domain containing 1 (IMPAD1) | NM_017813 | 0.0312103 | -1.414 | -1.166 | -1.496 |
| ITFG2 | integrin alpha FG-GAP repeat containing 2 (ITFG2) | NM_018463 | 0.0254437 | -1.002 | 1.315 | 2.0005 |
| NM_004517 | integrin-linked kinase (ILK) | NM_004517 | 0.005506 | 1.12 | 1.2808 | 1.3132 |
| IFRG15 | interferon responsive gene 15 (IFRG15) | NM_022347 | 0.0425541 | -1.064 | -1.205 | -1.96 |
| IL11RA | interleukin 11 receptor, alpha (IL11RA), transcript variant 2 | NM_147162 | 0.0411768 | 2.1904 | 1.1286 | -1.245 |
| IL22RA1 | interleukin 22 receptor, alpha 1 (IL22RA1) | NM_021258 | 0.0033508 | 2.227 | -1.125 | 3.7357 |
| IFT122 | intraflagellar transport 122 homolog (Chlamydomonas) (IFT122), transcript variant 3 | NM_018262 | 0.0262107 | 1.0005 | 1.3125 | 1.1943 |
| IFT140 | intraflagellar transport 140 homolog (Chlamydomonas) (IFT140) | NM_014714 | 5.96E-04 | 1.0179 | 1.2173 | 2.6028 |
| NM_005354 | jun D proto-oncogene (JUND) | NM_005354 | 0.0487768 | 1.124 | 1.1845 | 1.6439 |
| NM_006855 | KDEL (Lys-Asp-Glu-Leu) endoplasmic reticulum protein retention receptor 3 (KDELR3), transcript variant 1 | NM_006855 | 0.0397502 | -1.641 | 1.0109 | -1.359 |
| KLHDC8B | kelch domain containing 8B (KLHDC8B) | NM_173546 | 0.0238237 | 1.1402 | -1.001 | 1.837 |
| KLHL17 | kelch-like 17 (Drosophila) (KLHL17) | NM_198317 | 0.0285051 | 1.4397 | 1.2308 | 1.2829 |
| KLHL30 | kelch-like 30 (Drosophila) (KLHL30) | NM_198582 | 0.0458518 | 1.3719 | 1.3926 | 1.6705 |
| KRT33A | keratin 33A (KRT33A) | NM_004138 | 0.0355757 | 1.8124 | 1.1581 | -1.01 |
| KRT37 | keratin 37 (KRT37) | NM_003770 | 0.0358268 | 1.7717 | 1.3119 | -1.035 |
| NM_198691 | keratin associated protein 10-1 (KRTAP10-1) | NM_198691 | 0.0196965 | 1.6671 | 1.5203 | -1.143 |
| KRTAP10-10 | keratin associated protein 10-10 (KRTAP10-10) | NM_181688 | 0.0337096 | 1.6735 | 1.2613 | -1.048 |
| NM_198692 | keratin associated protein 10-11 (KRTAP10-11) | NM_198692 | 0.0319435 | 1.3438 | -3.361 | -1.655 |
| NM_198698 | keratin associated protein 12-4 (KRTAP12-4) | NM_198698 | 0.0395041 | -1.092 | -1.188 | -3.152 |
| NM_181612 | keratin associated protein 19-6 (KRTAP19-6) | NM_181612 | 9.88E-04 | 1.3831 | 1.0986 | 2.2526 |
| URB2 | KIAA0133 (KIAA0133) | NM_014777 | 0.0145741 | 1.3204 | 1.2094 | 1.9499 |
| KIAA0182 | KIAA0182 (KIAA0182) | NM_014615 | 0.0074288 | 1.3925 | 1.2726 | 1.7205 |
| KIAA0319L | KIAA0319-like (KIAA0319L), transcript variant 2 | NM_182686 | 0.042602 | 1.5465 | 1.1225 | 1.7713 |
| TECPR2 | KIAA0329 (KIAA0329) | NM_014844 | 0.0049225 | 1.2661 | 1.3102 | 2.2766 |
| KIAA0564 | KIAA0564 protein (KIAA0564), transcript variant 2 | NM_001009814 | 0.0428524 | -1.691 | 1.1629 | -1.398 |
| KIAA0652 | KIAA0652 (KIAA0652) | NM_014741 | 0.0424156 | 1.265 | 1.5899 | -1.1 |
| KIAA0776 | KIAA0776 (KIAA0776) | NM_015323 | 4.58E-05 | -1.969 | -1.536 | -4.121 |
| KIAA0913 | KIAA0913 (KIAA0913) | NM_015037 | 0.0125254 | 1.2202 | 1.125 | 1.6921 |
| KIAA1128 | KIAA1128 (KIAA1128) | NM_018999 | 0.0455322 | -1.133 | 1.0187 | -1.533 |
| CCDC146 | KIAA1505 protein (KIAA1505) | NM_020879 | 0.0306615 | 1.0666 | 5.2815 | 1.3606 |
| NEURL4 | KIAA1787 protein (KIAA1787), transcript variant 1 | NM_032442 | 0.0295133 | 1.1001 | 1.2202 | 2.0274 |
| KIAA1853 | KIAA1853 (KIAA1853) | NM_194286 | 0.0497938 | 1.807 | 1.87 | 1.1417 |
| KLC2 | kinesin light chain 2 (KLC2) | NM_022822 | 0.0019457 | 1.0209 | 1.1668 | 1.965 |
| KLC4 | kinesin light chain 4 (KLC4), transcript variant 3 | NM_201523 | 0.0093248 | 1.1806 | 1.3744 | 1.2507 |
| KRBA1 | KRAB-A domain containing 1 (KRBA1) | NM_032534 | 0.0116077 | 1.2548 | 1.7013 | 1.4431 |
| NM_032857 | lactamase, beta (LACTB), nuclear gene encoding mitochondrial protein, transcript variant 1 | NM_032857 | 4.39E-04 | -1.34 | -1.327 | -1.503 |
| LAS1L | LAS1-like (S. cerevisiae) (LAS1L) | NM_031206 | 0.0209205 | 1.2235 | 1.245 | 1.2933 |
| LTBP3 | latent transforming growth factor beta binding protein 3 (LTBP3) | NM_021070 | 0.0137505 | 1.0731 | 1.1233 | 1.869 |
| LMAN1L | lectin, mannose-binding, 1 like (LMAN1L) | NM_021819 | 0.0391857 | 2.0026 | 1.1952 | -1.086 |
| LEPROTL1 | leptin receptor overlapping transcript-like 1 (LEPROTL1) | NM_015344 | 0.0377367 | -1.031 | -1.053 | -1.675 |
| NM_001004055 | leucine rich repeat containing 29 (LRRC29), transcript variant 2 | NM_001004055 | 0.0205494 | 1.1613 | 1.3386 | 1.469 |
| LRRC40 | leucine rich repeat containing 40 (LRRC40) | NM_017768 | 0.0349841 | -1.253 | -1.183 | -1.528 |
| LRRC42 | leucine rich repeat containing 42 (LRRC42) | NM_052940 | 0.0429174 | -1.039 | -1.381 | -1.366 |
| LRRC56 | leucine rich repeat containing 56 (LRRC56) | NM_198075 | 3.12E-04 | 2.0021 | 1.1576 | 1.3877 |
| NM_002319 | leucine-rich repeats and calponin homology (CH) domain containing 4 (LRCH4) | NM_002319 | 0.0475144 | 1.2714 | -1.03 | 2.9507 |
| MBOAT7 | leukocyte receptor cluster (LRC) member 4 (LENG4) | NM_024298 | 0.0053191 | 1.3153 | 1.0242 | 1.2535 |
| LENG8 | leukocyte receptor cluster (LRC) member 8 (LENG8) | NM_052925 | 0.0061913 | 1.0509 | 1.3705 | 1.5691 |
| LNX2 | ligand of numb-protein X 2 (LNX2) | NM_153371 | 0.0454688 | -1.331 | -1.018 | -1.408 |
| BEGAIN | likely ortholog of rat brain-enriched guanylate kinase-associated protein (KIAA1446) | NM_020836 | 0.0241397 | -1 | 1.3865 | 3.9732 |
| LRP5L | low density lipoprotein receptor-related protein 5-like (LRP5L) | NM_182492 | 0.0043124 | 2.0563 | 1.4083 | -1.037 |
| NM_018032 | LUC7-like (S. cerevisiae) (LUC7L), transcript variant 1 | NM_018032 | 0.037524 | 1.0072 | 1.1681 | 1.8077 |
| LUC7L2 | LUC7-like 2 (S. cerevisiae) (LUC7L2) | NM_016019 | 0.0139692 | 1.1281 | 1.3348 | 1.6423 |
| NM_024123 | lymphocyte antigen 6 complex, locus G6E (LY6G6E) | NM_024123 | 0.0410068 | 2.3184 | 1.3394 | -1.237 |
| LYRM5 | LYR motif containing 5 (LYRM5) | NM_001001660 | 0.0041064 | 1.1354 | -1.003 | -5.758 |
| LAMP1 | lysosomal-associated membrane protein 1 (LAMP1) | NM_005561 | 0.0168624 | 1.1125 | 1.0553 | 1.4513 |
| AY937248 | Macaca mulatta placental protein 14 mRNA, complete cds [AY937248] | AY937248 | 0.002621 | 2.7558 | -1.074 | 2.0461 |
| LOC729991-MEF2B | MADS box transcription enhancer factor 2, polypeptide B (myocyte enhancer factor 2B) (MEF2B) | NM_005919 | 0.0222132 | 1.2479 | 1.3235 | 1.3849 |
| NM_206892 | malate dehydrogenase 1B, NAD (soluble) (MDH1B) | NM_206892 | 0.0208784 | 1.6257 | 1.5764 | -1.03 |
| ANKHD1-EIF4EBP3 | MASK-4E-BP3 alternate reading frame gene (MASK-BP3) | NM_020690 | 0.0452658 | 1.2011 | 1.0906 | 1.7012 |
| MDM1 | Mdm4, transformed 3T3 cell double minute 1, p53 binding protein (mouse) (MDM1), transcript variant 1 | NM_017440 | 5.22E-05 | -1.126 | 1.2474 | -7.443 |
| NM_052877 | mediator of RNA polymerase II transcription, subunit 8 homolog (yeast) (MED8), transcript variant 2 | NM_052877 | 0.0307482 | 1.0679 | 1.3173 | 1.0776 |
| NM_000902 | membrane metallo-endopeptidase (neutral endopeptidase, enkephalinase, CALLA, CD10) (MME), transcript variant 1 | NM_000902 | 0.0409735 | 1.0079 | -1.35 | -2.881 |
| MPP7 | membrane protein, palmitoylated 7 (MAGUK p55 subfamily member 7) (MPP7) | NM_173496 | 0.0419458 | -2.048 | -2.347 | -1.105 |
| NM_021126 | mercaptopyruvate sulfurtransferase (MPST) | NM_021126 | 0.0163564 | 1.6326 | 1.4033 | 1.2231 |
| METT5D1 | methyltransferase 5 domain containing 1 (METT5D1) | NM_152636 | 0.0325403 | -1.15 | -1.358 | -1.756 |
| NM_024723 | MICAL-like 2 (MICAL-L2), transcript variant 2 | NM_024723 | 0.0075832 | -1.036 | 1.2386 | 2.6367 |
| MAPKBP1 | mitogen activated protein kinase binding protein 1 (MAPKBP1) | NM_014994 | 0.0232871 | 1.1795 | 1.1016 | 1.8302 |
| CB549543 | MMPL0003_F04 MMPL Macaca mulatta cDNA | CB549543 | 0.0437947 | -1.07 | -1.166 | -1.783 |
| CB549491 | MMPL0007_H01 MMPL Macaca mulatta cDNA | CB549491 | 0.0202714 | 1.0093 | -1.056 | -1.91 |
| CB548968 | MMPL0018_H05 MMPL Macaca mulatta cDNA | CB548968 | 0.017164 | 1.0553 | 1.1035 | 1.6778 |
| CB549630 | MMPL0019_G07 MMPL Macaca mulatta cDNA | CB549630 | 0.0246387 | 1.0681 | 1.3143 | 1.147 |
| CB549220 | MMPL0027_F12 MMPL Macaca mulatta cDNA | CB549220 | 0.0144041 | -1.313 | -1.171 | -2.032 |
| CB550176 | MMPL0028_H02 MMPL Macaca mulatta cDNA | CB550176 | 0.0367465 | 1.0616 | 1.0675 | 1.356 |
| MORC2 | MORC family CW-type zinc finger 2 (MORC2) | NM_014941 | 3.72E-05 | 1.2277 | 1.2753 | 1.5942 |
| MORF4 | mortality factor 4 (MORF4) | NM_006792 | 2.75E-04 | -1.362 | -1.127 | -1.667 |
| AB208892 | mRNA for hepatoma-derived growth factor-related protein 2 isoform 1 variant protein [AB208892] | AB208892 | 1.05E-04 | 1.6151 | 1.3045 | 2.0984 |
| AB209633 | mRNA for trithorax homolog protein [AB209633] | AB209633 | 0.0086029 | 1.2991 | 1.0488 | 2.0042 |
| AL512727 | mRNA; cDNA DKFZp547P042 (from clone DKFZp547P042) [AL512727] | AL512727 | 0.0338209 | 2.4575 | 1.9646 | 1.3301 |
| BX648585 | mRNA; cDNA DKFZp686E22185 (from clone DKFZp686E22185) [BX648585] | BX648585 | 0.0079966 | -1.982 | -1.27 | -3.084 |
| BX538347 | mRNA; cDNA DKFZp686H1983 (from clone DKFZp686H1983) [BX538347] | BX538347 | 0.0035823 | -1.305 | -1.076 | -2.434 |
| BX537731 | mRNA; cDNA DKFZp686O0762 (from clone DKFZp686O0762) [BX537731] | BX537731 | 0.0408859 | 1.7268 | 1.1767 | 1.1938 |
| MTERFD1 | MTERF domain containing 1 (MTERFD1) | NM_015942 | 7.46E-04 | -1.359 | -1.068 | -1.478 |
| BC044226 | myosin binding protein H | BC044226 | 0.0467789 | -4.4 | -1.967 | -1.502 |
| NM_002493 | NADH dehydrogenase (ubiquinone) 1 beta subcomplex, 6, 17kDa (NDUFB6), nuclear gene encoding mitochondrial protein, transcript variant 1 | NM_002493 | 0.003055 | -1.211 | -1.048 | -1.966 |
| NM_172167 | NADPH oxidase organizer 1 (NOXO1), transcript variant b | NM_172167 | 0.0021704 | 1.6737 | 1.328 | 1.5348 |
| NM_001007156 | neurotrophic tyrosine kinase, receptor, type 3 (NTRK3), transcript variant 3 | NM_001007156 | 0.0091964 | 1.1692 | 2.6189 | 1.6653 |
| NOS1AP | nitric oxide synthase 1 (neuronal) adaptor protein (NOS1AP) | NM_014697 | 0.0410832 | -1.001 | 1.3656 | 2.1503 |
| NSFL1C | NSFL1 (p97) cofactor (p47) (NSFL1C), transcript variant 1 | NM_016143 | 0.0373946 | 1.1391 | 1.4088 | -1.112 |
| NM_033334 | nuclear receptor subfamily 6, group A, member 1 (NR6A1), transcript variant 1 | NM_033334 | 0.0482471 | 1.276 | -2.285 | -1.338 |
| NOC4L | nucleolar complex associated 4 homolog (S. cerevisiae) (NOC4L) | NM_024078 | 0.0250483 | 1.2083 | 1.3082 | 1.1365 |
| NOL10 | nucleolar protein 10 (NOL10) | NM_024894 | 0.0352313 | 1.2799 | 1.4613 | 1.105 |
| NOL7 | nucleolar protein 7, 27kDa (NOL7) | NM_016167 | 0.0028892 | -1.448 | -1.112 | -3.076 |
| NUDT22 | nudix (nucleoside diphosphate linked moiety X)-type motif 22 (NUDT22) | NM_032344 | 0.0187306 | 1.1808 | 1.3317 | 1.2061 |
| NM_001005239 | olfactory receptor, family 11, subfamily H, member 1 (OR11H1) | NM_001005239 | 0.0121426 | 1.4715 | 2.0223 | -1.297 |
| NM_001005276 | olfactory receptor, family 2, subfamily AE, member 1 (OR2AE1) | NM_001005276 | 0.0359078 | 2.4427 | 1.1364 | -1.093 |
| NM_001004685 | olfactory receptor, family 2, subfamily F, member 2 (OR2F2) | NM_001004685 | 0.0434429 | 3.1748 | 1.3538 | -1.407 |
| NM_001005499 | olfactory receptor, family 6, subfamily C, member 70 (OR6C70) | NM_001005499 | 0.041527 | 1.5044 | 1.4397 | 1.1184 |
| OPTC | opticin (OPTC) | NM_014359 | 0.0132904 | 2.1587 | 1.3986 | 1.0021 |
| OTOP2 | otopetrin 2 (OTOP2) | NM_178160 | 0.0382212 | 2.2553 | 1.3505 | -1.171 |
| NM_001003712 | oxysterol binding protein-like 8 (OSBPL8), transcript variant 2 | NM_001003712 | 1.07E-04 | -1.397 | -1.129 | -8.052 |
| PNMA3 | paraneoplastic antigen MA3 (PNMA3) | NM_013364 | 0.0165137 | 1.4043 | 1.4791 | 1.1506 |
| NM_014451 | parathyroid hormone-responsive B1 gene (B1), transcript variant 1 | NM_014451 | 0.0318103 | 1.1096 | 1.1894 | 1.7256 |
| NM_080415 | peanut-like 2 (Drosophila) (PNUTL2), transcript variant 2 | NM_080415 | 0.0234299 | -1.023 | 1.7159 | 1.2251 |
| PELI3 | pellino homolog 3 (Drosophila) (PELI3) | NM_145065 | 0.0436012 | 1.3208 | 1.0546 | 1.6495 |
| NM_001001928 | peroxisome proliferative activated receptor, alpha (PPARA), transcript variant 3 | NM_001001928 | 0.0291109 | -1.028 | 1.2189 | 2.5108 |
| PHF15 | PHD finger protein 15 (PHF15) | NM_015288 | 0.0493893 | 1.4784 | 1.0039 | 1.748 |
| NM_024517 | PHD finger protein 2 (PHF2), transcript variant 2 | NM_024517 | 0.0014676 | 1.2453 | 1.3173 | 1.2232 |
| PHF8 | PHD finger protein 8 (PHF8) | NM_015107 | 4.98E-04 | 1.4116 | 1.0633 | 2.4289 |
| PPAPDC3 | phosphatidic acid phosphatase type 2 domain containing 3 (PPAPDC3) | NM_032728 | 0.0208358 | 1.1076 | 1.2637 | 1.5282 |
| JMJD7-PLA2G4B | phospholipase A2, group IVB (cytosolic) (PLA2G4B) | NM_005090 | 0.034079 | 1.2856 | 1.1574 | 2.6254 |
| BM423036 | PLATE1_C10 Rhesus Macaca mulatta cDNA | BM423036 | 0.0154015 | 1.2116 | 1.0483 | 1.959 |
| BM423201 | PLATE3_E12 Rhesus Macaca mulatta cDNA | BM423201 | 0.0051404 | 2.5152 | 1.0632 | 1.302 |
| PLEKHF2 | pleckstrin homology domain containing, family F (with FYVE domain) member 2 (PLEKHF2) | NM_024613 | 0.0372043 | -1.107 | -1.019 | -1.369 |
| PODNL1 | podocan-like 1 (PODNL1) | NM_024825 | 0.0192152 | 2.5247 | 1.0174 | -1.09 |
| PCBP4 | poly(rC) binding protein 4 (PCBP4), transcript variant 4 | NM_033010 | 3.36E-04 | 1.732 | 1.1343 | 1.772 |
| PCBP4 | poly(rC) binding protein 4 (PCBP4), transcript variant 4 | NM_033010 | 4.80E-04 | 1.6471 | 1.1191 | 1.8649 |
| NM_144494 | polyglutamine binding protein 1 (PQBP1) | NM_144494 | 4.56E-04 | 1.1045 | 1.1977 | 1.4648 |
| NM_144494 | polyglutamine binding protein 1 (PQBP1) | NM_144494 | 0.0129857 | 1.3067 | 1.5156 | 1.8674 |
| PRAMEF1 | PRAME family member 1 (PRAMEF1) | NM_023013 | 0.0387379 | 1.7016 | 1.689 | -1.149 |
| XM_371279 | PREDICTED: amphoterin-induced gene (KIAA1163) | XM_371279 | 0.0194817 | 1.2465 | 1.6639 | 1.1818 |
| XM_208213 | PREDICTED: cytochrome P450, family 4, subfamily A, polypeptide 22 (CYP4A22) | XM_208213 | 0.0186021 | 1.3195 | 1.2134 | 1.8811 |
| XM_374578 | PREDICTED: enhancer of zeste homolog 2 (Drosophila) (EZH2) | XM_374578 | 0.0040744 | -1.396 | -1.166 | -1.493 |
| XM_378195 | PREDICTED: general transcription factor IIH, polypeptide 4, 52kDa (GTF2H4) | XM_378195 | 0.0249791 | 1.0662 | 1.1757 | 1.4083 |
| XM_166529 | PREDICTED: glucocorticoid induced transcript 1 (GLCCI1) | XM_166529 | 0.0099819 | 1.1422 | 1.1298 | 2.1103 |
| XM_056254 | PREDICTED: heparan sulfate (glucosamine) 3-O-sulfotransferase 4 (HS3ST4) | XM_056254 | 0.0218958 | 9.5692 | -2.092 | 1.3919 |
| XM_495934 | PREDICTED: hypothetical protein 15E1.2 (15E1.2) | XM_495934 | 1.95E-04 | -1.507 | -1.444 | -1.323 |
| XM_371897 | PREDICTED: hypothetical protein DKFZp761I2123 (DKFZp761I2123) | XM_371897 | 0.0289136 | -1.031 | 1.1771 | 1.7773 |
| XM_113796 | PREDICTED: hypothetical protein LOC196996 (LOC196996) | XM_113796 | 0.0138695 | -1.832 | -1.648 | -1.336 |
| XM_290517 | PREDICTED: KIAA0404 protein (KIAA0404) | XM_290517 | 0.0451385 | 1.0094 | 1.2982 | 1.7189 |
| XM_051699 | PREDICTED: KIAA1344 (KIAA1344) | XM_051699 | 0.0464029 | 1.1768 | 1.1327 | 1.6647 |
| XM_371832 | PREDICTED: KIAA1411 (KIAA1411) | XM_371832 | 0.0281144 | -1.169 | -1.469 | -1.591 |
| XM_498527 | PREDICTED: LOC440068 (LOC440068) | XM_498527 | 0.0110539 | -1.51 | -1.447 | -1.418 |
| XR_013427 | PREDICTED: Macaca mulatta alanyl-tRNA synthetase (AARS) | XR_013427 | 0.0026736 | 1.0568 | 1.29 | 1.5238 |
| XR_010902 | PREDICTED: Macaca mulatta ash2 (absent, small, or homeotic)-like (ASH2L) | XR_010902 | 0.0337732 | -1.504 | -1.226 | -1.489 |
| XR_010913 | PREDICTED: Macaca mulatta calcineurin binding protein 1 (CABIN1) | XR_010913 | 0.0010167 | 1.0866 | 1.4571 | 1.7902 |
| XR_014749 | PREDICTED: Macaca mulatta calcium channel, voltage-dependent, alpha 1H subunit (CACNA1H) | XR_014749 | 3.06E-05 | 1.4723 | 1.8584 | 2.608 |
| XR_012927 | PREDICTED: Macaca mulatta CLN3 protein (CLN3) | XR_012927 | 0.0208974 | 1.1088 | 1.0098 | 1.5393 |
| XR_014487 | PREDICTED: Macaca mulatta glutamate receptor, metabotropic 4 (GRM4) | XR_014487 | 0.0229395 | 1.7316 | 1.2943 | 1.3236 |
| XR_009789 | PREDICTED: Macaca mulatta hypothetical protein LOC693798 (LOC693798) | XR_009789 | 0.0437924 | -1.014 | -1.219 | -1.505 |
| XR_009982 | PREDICTED: Macaca mulatta hypothetical protein LOC695780 (LOC695780) | XR_009982 | 0.0365608 | -1.575 | 1.0097 | -1.204 |
| XR_011615 | PREDICTED: Macaca mulatta hypothetical protein LOC699195 (LOC699195) | XR_011615 | 0.0163905 | -1.498 | -1.022 | -2.445 |
| XR_011417 | PREDICTED: Macaca mulatta hypothetical protein LOC700304 (LOC700304) | XR_011417 | 0.0049613 | -1.166 | -1.006 | -1.647 |
| XR_011967 | PREDICTED: Macaca mulatta hypothetical protein LOC703409 (LOC703409) | XR_011967 | 0.0028011 | 1.1253 | 1.1185 | 1.3871 |
| XR_011305 | PREDICTED: Macaca mulatta hypothetical protein LOC704599 (LOC704599) | XR_011305 | 0.0383957 | 1.4962 | 1.1345 | 1.1298 |
| XR_012376 | PREDICTED: Macaca mulatta hypothetical protein LOC710335 (LOC710335) | XR_012376 | 0.0225543 | -1.93 | -3.07 | -1.439 |
| XR_012295 | PREDICTED: Macaca mulatta hypothetical protein LOC710818 (LOC710818) | XR_012295 | 0.0010697 | -1.443 | -1.512 | -6.319 |
| XR_013014 | PREDICTED: Macaca mulatta hypothetical protein LOC711927 (LOC711927) | XR_013014 | 0.0201115 | 1.0803 | 1.2125 | 1.4825 |
| XR_014265 | PREDICTED: Macaca mulatta hypothetical protein LOC716045 (LOC716045) | XR_014265 | 0.0013825 | -3.232 | -1.27 | -2.169 |
| XR_014077 | PREDICTED: Macaca mulatta hypothetical protein LOC719082 (LOC719082) | XR_014077 | 0.0248046 | 1.2412 | 1.0697 | 1.2906 |
| XR_014077 | PREDICTED: Macaca mulatta hypothetical protein LOC719082 (LOC719082) | XR_014077 | 0.0308466 | 1.2398 | 1.0854 | 1.2932 |
| XR_014204 | PREDICTED: Macaca mulatta hypothetical protein LOC719546 (LOC719546) | XR_014204 | 0.0292259 | -3.167 | -3.849 | 1.0848 |
| XR_014590 | PREDICTED: Macaca mulatta hypothetical protein LOC721251 (LOC721251) | XR_014590 | 0.0012247 | 1.2217 | 1.1452 | 1.562 |
| XR_014848 | PREDICTED: Macaca mulatta hypothetical protein LOC723278 (LOC723278) | XR_014848 | 0.049131 | 1.3468 | 1.2944 | 1.0787 |
| XR_012553 | PREDICTED: Macaca mulatta IQ motif containing GTPase activating protein 2 (IQGAP2) | XR_012553 | 0.0061201 | -1.843 | -1.764 | 1.1649 |
| XR_012427 | PREDICTED: Macaca mulatta PTPRF interacting protein alpha 1 (PPFIA1) | XR_012427 | 0.0028461 | 1.2172 | 1.1885 | 1.5468 |
| XR_011731 | PREDICTED: Macaca mulatta similar to acidic (leucine-rich) nuclear phosphoprotein 32 family, member A (LOC707419) | XR_011731 | 0.0489562 | 1.5562 | 1.1159 | 1.2822 |
| XR_011326 | PREDICTED: Macaca mulatta similar to adipocyte enhancer binding protein 1 precursor (LOC699977) | XR_011326 | 0.0420843 | 1.0912 | 1.5212 | 1.3646 |
| XR_011510 | PREDICTED: Macaca mulatta similar to anaphase-promoting complex subunit 5 (LOC699821) | XR_011510 | 0.0065167 | 1.1098 | 1.2398 | 1.0111 |
| XR_012049 | PREDICTED: Macaca mulatta similar to ankyrin repeat and sterile alpha motif domain containing 3 (LOC706182) | XR_012049 | 0.0079454 | 1.1394 | 1.1817 | 1.9438 |
| XR_010844 | PREDICTED: Macaca mulatta similar to Annexin A2 (Annexin II) (Lipocortin II) (Calpactin I heavy chain) (Chromobindin-8) (p36) (Protein I) (Placental anticoagulant protein IV) (PAP-IV) (LOC697019) | XR_010844 | 0.0255438 | -1.609 | -1.233 | -1.76 |
| XR_013392 | PREDICTED: Macaca mulatta similar to arginyl aminopeptidase (aminopeptidase B)-like 1 (LOC716318) | XR_013392 | 0.0227833 | 1.2556 | 1.1331 | 1.5093 |
| XR_012211 | PREDICTED: Macaca mulatta similar to autoantigen La (LOC710344) | XR_012211 | 7.92E-05 | -1.44 | 1.0099 | -3.358 |
| XR_010556 | PREDICTED: Macaca mulatta similar to BCL2-associated athanogene 5 isoform a (LOC699170) | XR_010556 | 0.0462962 | 1.1579 | 1.1361 | 1.4438 |
| XR_013227 | PREDICTED: Macaca mulatta similar to beta isoform of regulatory subunit A, protein phosphatase 2 isoform b (LOC710387) | XR_013227 | 0.0167933 | 1.4543 | 1.3873 | 2.3292 |
| XR_013102 | PREDICTED: Macaca mulatta similar to bromodomain and WD repeat domain containing 2 (LOC714990) | XR_013102 | 9.65E-05 | -1.443 | -1.098 | -6.994 |
| XR_011163 | PREDICTED: Macaca mulatta similar to carbamoylphosphate synthetase 2 [XR_011163] | XR_011163 | 0.0199721 | -1.016 | 1.2784 | 1.6309 |
| XR_010753 | PREDICTED: Macaca mulatta similar to CBF1 interacting corepressor (LOC698068) | XR_010753 | 0.0213686 | 1.2576 | 1.2994 | -1.011 |
| XR_012933 | PREDICTED: Macaca mulatta similar to centaurin, gamma 3 (LOC714140) | XR_012933 | 0.002971 | 1.3569 | 1.0991 | 2.0302 |
| XR_010248 | PREDICTED: Macaca mulatta similar to CG17065-PA (LOC697051) | XR_010248 | 0.0127606 | 1.1054 | 1.4033 | 1.4817 |
| XR_012999 | PREDICTED: Macaca mulatta similar to CG31643-PA (LOC706154) | XR_012999 | 0.0165358 | -1.626 | -1.07 | -1.75 |
| XR_010395 | PREDICTED: Macaca mulatta similar to CG3184-PA (LOC703764) | XR_010395 | 0.0157396 | 1.8677 | 1.4008 | 2.099 |
| XR_014425 | PREDICTED: Macaca mulatta similar to CG4203-PA (LOC720347) | XR_014425 | 0.0082955 | 1.341 | 1.221 | 1.5064 |
| XR_012668 | PREDICTED: Macaca mulatta similar to CG5645-PA (LOC713047) | XR_012668 | 0.0121404 | 1.2052 | -1.019 | 2.0078 |
| XR_012773 | PREDICTED: Macaca mulatta similar to CG9581-PA (LOC705778) | XR_012773 | 0.023391 | 1.0952 | 1.3176 | 1.3943 |
| XR_013229 | PREDICTED: Macaca mulatta similar to Charged multivesicular body protein 3 (Chromatin-modifying protein 3) (Vacuolar protein sorting 24) (hVps24) (Neuroendocrine differentiation factor) (VPS24) | XR_013229 | 0.0236717 | 1.0916 | 1.2517 | 1.3584 |
| XR_013120 | PREDICTED: Macaca mulatta similar to CHMP family, member 7 (LOC710050) | XR_013120 | 0.0208052 | 1.1817 | 1.4755 | 1.2526 |
| XR_010573 | PREDICTED: Macaca mulatta similar to citron (LOC703199) | XR_010573 | 0.0140857 | 1.5799 | 1.329 | 1.3453 |
| XR_012647 | PREDICTED: Macaca mulatta similar to CKLF-like MARVEL transmembrane domain-containing protein 6 (Chemokine-like factor superfamily member 6) (CMTM6) | XR_012647 | 0.0040428 | -1.497 | -1.223 | -1.346 |
| XR_011312 | PREDICTED: Macaca mulatta similar to Coatomer subunit beta (Beta-coat protein) (Beta-COP) (LOC699327) | XR_011312 | 0.0254395 | -1.031 | 1.1213 | -2.15 |
| XR_010402 | PREDICTED: Macaca mulatta similar to coiled-coil domain containing 64 (LOC698147) | XR_010402 | 0.0314706 | 1.6588 | 1.2974 | -1.037 |
| XR_014262 | PREDICTED: Macaca mulatta similar to collagen-like protein (LOC719698) | XR_014262 | 0.016396 | 2.2109 | 1.5463 | -1.347 |
| XR_014707 | PREDICTED: Macaca mulatta similar to Complement C1r subcomponent precursor (Complement component 1, r subcomponent) (LOC722131) | XR_014707 | 0.0338382 | -1.821 | -1.557 | -1.383 |
| XR_014757 | PREDICTED: Macaca mulatta similar to Crm, cramped-like (LOC722473) | XR_014757 | 0.0307287 | 1.3179 | 1.1069 | 1.4611 |
| XR_014633 | PREDICTED: Macaca mulatta similar to Dipeptidyl-peptidase 2 precursor (Dipeptidyl-peptidase II) (DPP II) (Dipeptidyl aminopeptidase II) (Quiescent cell proline dipeptidase) (Dipeptidyl peptidase 7) (LOC721595) | XR_014633 | 0.0466924 | 1.524 | 1.0698 | 1.6658 |
| XR_010309 | PREDICTED: Macaca mulatta similar to eukaryotic translation elongation factor 1 delta isoform 1 (LOC697625) | XR_010309 | 0.0024312 | 1.2775 | 1.1439 | 1.3686 |
| XR_011196 | PREDICTED: Macaca mulatta similar to G protein-coupled receptor 108 (LOC703831) | XR_011196 | 0.0027036 | 1.1836 | 1.0006 | 1.4547 |
| XR_010365 | PREDICTED: Macaca mulatta similar to glucocorticoid induced transcript 1 (LOC696004) | XR_010365 | 0.010635 | 1.0859 | 1.1555 | 2.2402 |
| XR_009800 | PREDICTED: Macaca mulatta similar to Golgi autoantigen, golgin subfamily a, 2 (LOC694113) | XR_009800 | 0.0269485 | 1.2108 | 1.6349 | 1.7813 |
| XR_014443 | PREDICTED: Macaca mulatta similar to hydroxysteroid (17-beta) dehydrogenase 7 (LOC720399) | XR_014443 | 0.0196467 | -1.525 | -1.042 | -1.039 |
| XR_014443 | PREDICTED: Macaca mulatta similar to hydroxysteroid (17-beta) dehydrogenase 7 (LOC720399) | XR_014443 | 0.025165 | -1.438 | 1.159 | -1.702 |
| XR_010973 | PREDICTED: Macaca mulatta similar to inter-alpha (globulin) inhibitor H5 (LOC701602) | XR_010973 | 0.0284034 | 1.3237 | -1.356 | -2.431 |
| XR_011036 | PREDICTED: Macaca mulatta similar to IQ motif and Sec7 domain 2 (LOC702562) | XR_011036 | 0.0037608 | 1.0801 | 1.1353 | 1.5214 |
| XR_010590 | PREDICTED: Macaca mulatta similar to leucine rich repeat and death domain containing protein isoform 1 (LOC700580) | XR_010590 | 7.55E-04 | 1.2179 | 1.0078 | 2.6598 |
| XR_010288 | PREDICTED: Macaca mulatta similar to Leydig cell tumor 10 kDa protein homolog (LOC697972) | XR_010288 | 0.0062127 | 1.3678 | 1.0637 | 1.3414 |
| XR_014613 | PREDICTED: Macaca mulatta similar to Low molecular weight phosphotyrosine protein phosphatase (LMW-PTP) (Low molecular weight cytosolic acid phosphatase) (Red cell acid phosphatase 1) (PTPase) (Adipocyte acid phosphatase) (LOC721397) | XR_014613 | 0.0494272 | -1.352 | -1.187 | -1.385 |
| XR_010672 | PREDICTED: Macaca mulatta similar to Molybdenum cofactor synthesis protein 2 large subunit (Molybdopterin synthase large subunit) (MPT synthase large subunit) (MOCS2B) (MOCO1-B) (LOC703049) | XR_010672 | 5.09E-05 | -1.602 | 1.0594 | -11.63 |
| XR_013651 | PREDICTED: Macaca mulatta similar to Muellerian-inhibiting factor precursor (MIS) (Anti-Muellerian hormone) (AMH) (Mullerian-inhibiting substance) (LOC717539) | XR_013651 | 0.0158431 | -1.185 | -1.512 | -1.541 |
| XR_012588 | PREDICTED: Macaca mulatta similar to myosin, heavy polypeptide 7B, cardiac muscle, beta (LOC712230) | XR_012588 | 0.0142064 | 2.1402 | 1.3958 | 2.1562 |
| XR_010838 | PREDICTED: Macaca mulatta similar to NADPH dependent diflavin oxidoreductase 1 (LOC706056) | XR_010838 | 0.0260292 | 1.0748 | 1.2008 | 2.1114 |
| XR_014490 | PREDICTED: Macaca mulatta similar to nephroretinin (LOC720707) | XR_014490 | 0.0034691 | 1.2346 | 1.6783 | 1.907 |
| XR_011345 | PREDICTED: Macaca mulatta similar to otoancorin isoform 1 (LOC699600) | XR_011345 | 0.0292551 | 2.6165 | 1.4501 | -1.493 |
| XR_013556 | PREDICTED: Macaca mulatta similar to PCTAIRE protein kinase 2 (LOC717183) | XR_013556 | 0.0226923 | -1.342 | -1.083 | -1.604 |
| XR_010681 | PREDICTED: Macaca mulatta similar to peroxisomal biogenesis factor 14 (LOC699373) | XR_010681 | 0.0234451 | -1.075 | 1.4241 | 1.4262 |
| XR_013413 | PREDICTED: Macaca mulatta similar to PGC-1-related estrogen receptor alpha coactivator (LOC711027) | XR_013413 | 0.0214568 | -1.214 | 1.6666 | 2.6185 |
| XR_013194 | PREDICTED: Macaca mulatta similar to Phosphomannomutase 1 (PMM 1) (PMMH-22) (PMM1) | XR_013194 | 0.0340103 | 1.5501 | 1.2152 | 1.2219 |
| XR_012775 | PREDICTED: Macaca mulatta similar to pleckstrin homology domain containing, family G, member 3 (LOC708025) | XR_012775 | 8.51E-05 | 1.3277 | 1.2434 | 1.8819 |
| XR_011794 | PREDICTED: Macaca mulatta similar to poly(A)-specific ribonuclease (PARN)-like domain containing 1 (LOC707835) | XR_011794 | 0.0105199 | 3.7855 | 3.6371 | 2.7676 |
| XR_010175 | PREDICTED: Macaca mulatta similar to potassium channel tetramerisation domain containing 9 (LOC696565) | XR_010175 | 0.0025111 | -1.516 | -1.26 | -1.721 |
| XR_013376 | PREDICTED: Macaca mulatta similar to Pre-B-cell leukemia transcription factor 2 (Homeobox protein PBX2) (Protein G17) (LOC716239) | XR_013376 | 0.0170318 | 1.9277 | 2.0932 | -1.093 |
| XR_014116 | PREDICTED: Macaca mulatta similar to Probable U3 small nucleolar RNA-associated protein 11 (U3 snoRNA-associated protein 11) (UTP11-like protein) (UTP11L) | XR_014116 | 0.0050538 | -1.551 | -1.088 | -1.332 |
| XR_013098 | PREDICTED: Macaca mulatta similar to Protein KIAA0323 (LOC714937) | XR_013098 | 0.0425286 | 1.2722 | 1.0005 | 1.5308 |
| XR_011100 | PREDICTED: Macaca mulatta similar to purity of essence CG14472-PA (LOC702449) | XR_011100 | 0.0211108 | 1.1406 | 1.0133 | 1.7879 |
| XR_010591 | PREDICTED: Macaca mulatta similar to Putative eukaryotic translation initiation factor 3 subunit (eIF-3) (LOC704219) | XR_010591 | 0.0170343 | -1.013 | 1.3799 | 1.6331 |
| XR_014152 | PREDICTED: Macaca mulatta similar to radical fringe homolog (LOC719330) | XR_014152 | 0.0231141 | 1.4095 | 1.1129 | 1.2101 |
| XR_013445 | PREDICTED: Macaca mulatta similar to Rho GTPase activating protein 5 isoform b (LOC716644) | XR_013445 | 0.0395951 | -1.542 | 1.0216 | -1.262 |
| XR_011614 | PREDICTED: Macaca mulatta similar to ribonucleic acid binding protein S1 (predicted) (LOC702134) | XR_011614 | 0.0156043 | 1.0865 | 1.3282 | 1.5792 |
| XR_010511 | PREDICTED: Macaca mulatta similar to RUN and TBC1 domain containing 1 (LOC703543) | XR_010511 | 9.90E-05 | 1.5488 | 1.3944 | 1.4847 |
| XR_010998 | PREDICTED: Macaca mulatta similar to Sec23 (S. cerevisiae) homolog B (LOC698440) | XR_010998 | 2.08E-05 | -1.476 | -1.044 | -3.431 |
| XR_013517 | PREDICTED: Macaca mulatta similar to selenoprotein T (LOC710572) | XR_013517 | 0.003465 | -2.044 | -1.137 | -1.345 |
| XR_013975 | PREDICTED: Macaca mulatta similar to SET domain containing 1A (LOC712416) | XR_013975 | 0.0020217 | 1.3039 | 1.1439 | 1.9831 |
| XR_014103 | PREDICTED: Macaca mulatta similar to SIN3 homolog B, transcription regulator (LOC718813) | XR_014103 | 0.0067467 | 1.3259 | -1.038 | 1.901 |
| XR_012915 | PREDICTED: Macaca mulatta similar to solute carrier family 13 (sodium-dependent citrate transporter), member 5 (LOC713292) | XR_012915 | 0.0417732 | 1.8661 | 1.0266 | 1.6338 |
| XR_012251 | PREDICTED: Macaca mulatta similar to superkiller viralicidic activity 2-like 2 (LOC706419) | XR_012251 | 0.0481489 | -1.137 | 1.0182 | -1.188 |
| XR_013291 | PREDICTED: Macaca mulatta similar to Survival of motor neuron-related splicing factor 30 (SMN-related protein) (30 kDa splicing factor SMNrp) (Survival motor neuron domain-containing protein 1) (SMNDC1) | XR_013291 | 0.0271996 | -1.274 | -1.093 | -1.629 |
| XR_014641 | PREDICTED: Macaca mulatta similar to SWI [XR_014641] | XR_014641 | 0.0182879 | -1.103 | 1.2086 | 1.634 |
| XR_012592 | PREDICTED: Macaca mulatta similar to tau tubulin kinase 2 (LOC712249) | XR_012592 | 0.0120737 | 1.1521 | 1.162 | 1.6015 |
| XR_011624 | PREDICTED: Macaca mulatta similar to tetratricopeptide repeat domain 16 (LOC706784) | XR_011624 | 0.0327079 | 2.4683 | 1.3417 | 1.8936 |
| XR_010760 | PREDICTED: Macaca mulatta similar to tetratricopeptide repeat domain 19 (LOC700653) | XR_010760 | 0.0218717 | 1.141 | 1.1059 | 1.9341 |
| XR_011738 | PREDICTED: Macaca mulatta similar to tight junction protein 4 (peripheral) (LOC700496) | XR_011738 | 8.59E-04 | 1.2317 | 1.3086 | 1.8281 |
| XR_009827 | PREDICTED: Macaca mulatta similar to titin isoform N2-A (LOC694413) | XR_009827 | 0.014185 | 1.3079 | 1.4976 | 1.4138 |
| XR_011737 | PREDICTED: Macaca mulatta similar to transcription factor B2, mitochondrial (LOC710669) | XR_011737 | 0.0019403 | -1.737 | 1.168 | -13.59 |
| XR_012463 | PREDICTED: Macaca mulatta similar to Transmembrane anchor protein 1 (LOC711598) | XR_012463 | 0.0180131 | 1.3597 | 1.3543 | 1.343 |
| XR_012340 | PREDICTED: Macaca mulatta similar to tubulin tyrosine ligase-like family, member 5 (LOC703116) | XR_012340 | 0.0260267 | 1.3459 | -1.02 | 1.2549 |
| XR_013652 | PREDICTED: Macaca mulatta similar to Ubiquitin carboxyl-terminal hydrolase 4 (Ubiquitin thioesterase 4) (Ubiquitin-specific-processing protease 4) (Deubiquitinating enzyme 4) (Ubiquitous nuclear protein homolog) (USP4) | XR_013652 | 0.0063059 | 1.3338 | 1.1562 | 1.3079 |
| XR_014393 | PREDICTED: Macaca mulatta similar to ubiquitin-activating enzyme E1-domain containing 1 isoform 1 (LOC717647) | XR_014393 | 0.0346095 | -1.199 | 1.0182 | -1.571 |
| XR_011803 | PREDICTED: Macaca mulatta similar to uncharacterized protein family UPF0227 member RGD1359682 (LOC709119) | XR_011803 | 0.0104021 | 1.1743 | 1.1729 | 1.481 |
| XR_014803 | PREDICTED: Macaca mulatta similar to vacuolar protein sorting 13D isoform 1 (LOC722775) | XR_014803 | 0.0421847 | 1.401 | 1.5848 | 1.0194 |
| XR_009904 | PREDICTED: Macaca mulatta similar to Vitamin K-dependent protein S precursor (LOC694845) | XR_009904 | 0.0103608 | -1.22 | -1.65 | -1.225 |
| XR_011876 | PREDICTED: Macaca mulatta similar to VprBP protein (LOC700596) | XR_011876 | 0.0017091 | 1.862 | 1.345 | -1.288 |
| XR_014300 | PREDICTED: Macaca mulatta similar to WD-repeat protein 12 (YTM1 homolog) (WDR12) | XR_014300 | 1.81E-05 | -1.356 | -1.059 | -2.413 |
| XR_013163 | PREDICTED: Macaca mulatta similar to Y45F10A.6b (LOC715258) | XR_013163 | 0.0455999 | 1.1404 | 1.124 | 1.2133 |
| XR_014207 | PREDICTED: Macaca mulatta similar to Yip1 interacting factor homolog B isoform 2 (LOC719551) | XR_014207 | 0.0355511 | 1.5607 | -1.117 | 1.3101 |
| XR_009814 | PREDICTED: Macaca mulatta similar to YTH domain containing 2 (LOC694612) | XR_009814 | 0.0065862 | -1.478 | -1.027 | -2.694 |
| XR_012771 | PREDICTED: Macaca mulatta similar to zinc finger protein 317 (LOC713200) | XR_012771 | 0.0451661 | 1.1396 | 1.0704 | 1.2902 |
| XR_012226 | PREDICTED: Macaca mulatta similar to zinc finger protein 749 (LOC710445) | XR_012226 | 0.0194094 | 1.3853 | 1.1485 | 1.4854 |
| XR_014683 | PREDICTED: Macaca mulatta similar to Zinc-finger protein ubi-d4 (Requiem) (Apoptosis response zinc finger protein) (D4, zinc and double PHD fingers family 2) (LOC721967) | XR_014683 | 0.0219258 | 1.1202 | 1.2344 | 1.8094 |
| XR_012677 | PREDICTED: Macaca mulatta solute carrier family 13 member 3 (SLC13A3) | XR_012677 | 0.0133917 | 1.4579 | 1.3945 | 1.32 |
| XR_014317 | PREDICTED: Macaca mulatta splicing factor 1 (SF1) | XR_014317 | 0.0067192 | 1.2162 | 1.137 | 1.3973 |
| XR_014317 | PREDICTED: Macaca mulatta splicing factor 1 (SF1) | XR_014317 | 0.0047463 | 1.2115 | 1.1208 | 1.5109 |
| XR_013847 | PREDICTED: Macaca mulatta TRM1 tRNA methyltransferase 1 (TRMT1) | XR_013847 | 0.0484157 | 1.1139 | 1.172 | 1.3773 |
| XR_014480 | PREDICTED: Macaca mulatta tubulin, gamma complex associated protein 6 (TUBGCP6) | XR_014480 | 0.0032817 | 1.0876 | 1.3088 | 2.2718 |
| XR_014323 | PREDICTED: Macaca mulatta ubiquitin protein ligase E3C (UBE3C) | XR_014323 | 0.0462205 | -1.053 | 1.2811 | 1.3081 |
| XR_011745 | PREDICTED: Macaca mulatta ubiquitin specific protease 19 (USP19) | XR_011745 | 0.0099555 | 1.0938 | 1.2525 | 1.4462 |
| XR_011954 | PREDICTED: Macaca mulatta zinc finger protein 446 (ZNF446) | XR_011954 | 1.57E-04 | 1.4381 | 1.2444 | 1.5929 |
| XM_038567 | PREDICTED: metastasis associated family, member 3 (MTA3) | XM_038567 | 0.0238812 | 1.0262 | 1.0268 | -1.904 |
| XM_041162 | PREDICTED: Nedd4 family interacting protein 2 (NDFIP2) | XM_041162 | 0.0138542 | -1.525 | -1.195 | -1.052 |
| XM_496526 | PREDICTED: phosphatidylinositol (4,5) bisphosphate 5-phosphatase, A (PIB5PA) | XM_496526 | 0.0355917 | 1.4932 | 1.0232 | 2.8393 |
| XM_496244 | PREDICTED: potassium channel tetramerisation domain containing 2 (KCTD2) | XM_496244 | 0.0326542 | 1.083 | 1.0639 | 1.5419 |
| XM_370663 | PREDICTED: roundabout, axon guidance receptor, homolog 3 (Drosophila) (ROBO3) | XM_370663 | 0.0257898 | 1.2475 | 1.2814 | 1.2614 |
| XM_048462 | PREDICTED: RUN and SH3 domain containing 2 (RUSC2) | XM_048462 | 0.008264 | 1.5948 | 1.2803 | 1.6781 |
| XM_375559 | PREDICTED: scaffold attachment factor B2 (SAFB2) | XM_375559 | 3.34E-04 | 1.348 | 1.1481 | 2.6913 |
| XM_371151 | PREDICTED: similar to 40S ribosomal protein S16 (LOC388519) | XM_371151 | 0.0272234 | -1.362 | -1.419 | -1.343 |
| XM_058581 | PREDICTED: similar to hypothetical protein 9530023G02 (MGC90512) | XM_058581 | 0.0127403 | 1.0893 | 1.3327 | 1.2094 |
| XM_291334 | PREDICTED: similar to hypothetical protein MGC15737 (LOC340543) | XM_291334 | 0.0094866 | -1.382 | 1.019 | -2.122 |
| XM_370715 | PREDICTED: similar to hypothetical protein MGC48915 (LOC387911) | XM_370715 | 0.0016141 | 1.5613 | 2.7566 | 2.3829 |
| XM_375284 | PREDICTED: similar to interleukin 9 receptor (LOC400481) | XM_375284 | 0.0450653 | 2.0935 | 1.8704 | -1.64 |
| XM_371837 | PREDICTED: similar to oxidoreductase UCPA (LOC389416) | XM_371837 | 2.06E-06 | -1.762 | -1.154 | -18.25 |
| XM_370763 | PREDICTED: similar to ribosomal protein L31 (LOC387991) | XM_370763 | 1.05E-05 | -1.526 | -1.41 | -8.804 |
| XM_495885 | PREDICTED: similar to ribosomal protein S12 (LOC440055) | XM_495885 | 0.002429 | -1.165 | -1.157 | -11.32 |
| XM_376819 | PREDICTED: similar to RIKEN 4933428I03 (LOC401494) | XM_376819 | 0.0203041 | -1.532 | -2.056 | -1.713 |
| XM_116936 | PREDICTED: similar to RIKEN cDNA 4832428D23 gene (LOC196541) | XM_116936 | 0.0179133 | 10.054 | 1.0804 | 2.1853 |
| XM_928905 | PREDICTED: similar to zinc finger protein 598 (LOC645937) | XM_928905 | 0.0427768 | 1.2371 | 1.1484 | 1.196 |
| XM_371581 | PREDICTED: Sp5 transcription factor (SP5) | XM_371581 | 0.0031109 | -1.747 | -2.592 | -2.944 |
| XR_000287 | PREDICTED: Tmp21-II , transcribed pseudogene (Tmp21-II), misc RNA [XR_000287] | XR_000287 | 0.0299629 | -1.173 | -1.129 | -1.398 |
| XM_496297 | PREDICTED: transmembrane 6 superfamily member 2 (TM6SF2) | XM_496297 | 0.0332004 | 1.5659 | 1.0892 | 1.3878 |
| XM_499565 | PREDICTED: twist homolog 2 (Drosophila) (TWIST2) | XM_499565 | 0.0248009 | 1.3012 | -1.155 | -2.944 |
| XM_035863 | PREDICTED: zinc finger protein 37a (KOX 21) (ZNF37A) | XM_035863 | 0.0398977 | -1.248 | -1.214 | -1.271 |
| XM_375247 | PREDICTED: zinc finger protein 592 (ZNF592) | XM_375247 | 0.0189476 | 1.0377 | 1.1412 | 1.4378 |
| XM_171060 | PREDICTED: zinc finger protein 620 (ZNF620) | XM_171060 | 0.0188457 | 1.465 | 1.5672 | 1.3175 |
| PCOLCE2 | procollagen C-endopeptidase enhancer 2 (PCOLCE2) | NM_013363 | 0.0457129 | -2.263 | -1.792 | -1.083 |
| PAQR6 | progestin and adipoQ receptor family member VI (PAQR6), transcript variant 1 | NM_024897 | 0.0066689 | 1.3165 | 1.55 | 1.2146 |
| PAQR6 | progestin and adipoQ receptor family member VI (PAQR6), transcript variant 1 | NM_024897 | 0.0039839 | 1.3421 | 1.5921 | 1.2172 |
| PDCD2L | programmed cell death 2-like (PDCD2L) | NM_032346 | 0.0461201 | -1.242 | 1.0935 | -1.628 |
| PRR3 | proline rich 3 (PRR3), transcript variant 1 | NM_025263 | 0.0062324 | 1.366 | 1.2572 | 1.3074 |
| NM_032104 | protein phosphatase 1, regulatory (inhibitor) subunit 12B (PPP1R12B), transcript variant 4 | NM_032104 | 0.0197282 | -1.099 | 1.5398 | 1.7282 |
| PPP1R12C | protein phosphatase 1, regulatory (inhibitor) subunit 12C (PPP1R12C) | NM_017607 | 0.0482932 | 1.0886 | 1.154 | 1.7695 |
| PPP1R3F | protein phosphatase 1, regulatory (inhibitor) subunit 3F (PPP1R3F) | NM_033215 | 0.0241363 | 1.349 | 1.5988 | -1.045 |
| PPFIA3 | protein tyrosine phosphatase, receptor type, f polypeptide (PTPRF), interacting protein (liprin), alpha 3 (PPFIA3) | NM_003660 | 0.025911 | 1.2445 | 1.4273 | 1.617 |
| NM_032086 | protocadherin gamma subfamily A, 6 (PCDHGA6), transcript variant 2 | NM_032086 | 0.01741 | 1.272 | 1.1853 | 1.7406 |
| NM_181334 | PRR5-ARHGAP8 fusion (LOC553158) | NM_181334 | 0.043141 | 2.5492 | 1.0823 | -1.353 |
| NM_198974 | PTK9 protein tyrosine kinase 9 (PTK9), transcript variant 2 | NM_198974 | 0.0138398 | -1.352 | -1.099 | -1.71 |
| PWWP2B | PWWP domain containing 2 (PWWP2) | NM_138499 | 0.022824 | 1.2644 | -1.106 | 1.9297 |
| NM_002853 | RAD1 homolog (S. pombe) (RAD1), transcript variant 1 | NM_002853 | 0.037651 | 1.5274 | 1.1966 | 1.1036 |
| NM_134423 | RAD52 homolog (S. cerevisiae) (RAD52), transcript variant gamma | NM_134423 | 0.0399198 | 1.2637 | 1.2947 | 1.548 |
| RSPH3 | radial spokehead-like 2 (RSHL2) | NM_031924 | 0.0499348 | 1.1828 | 1.2297 | 1.2758 |
| RANBP10 | RAN binding protein 10 (RANBP10) | NM_020850 | 0.0433726 | 1.5753 | 1.1835 | 1.0613 |
| NM_203365 | Ras association (RalGDS/AF-6) and pleckstrin homology domains 1 (RAPH1), transcript variant 3 | NM_203365 | 0.0054793 | 2.5006 | 1.5162 | 1.2306 |
| NM_007182 | Ras association (RalGDS/AF-6) domain family 1 (RASSF1), transcript variant A | NM_007182 | 0.0124592 | 1.3024 | 1.1479 | 1.4288 |
| RMND5B | required for meiotic nuclear division 5 homolog B (S. cerevisiae) (RMND5B) | NM_022762 | 0.0154856 | 1.5348 | 1.1615 | 1.2921 |
| RIC3 | resistance to inhibitors of cholinesterase 3 homolog (C. elegans) (RIC3) | NM_024557 | 0.0343069 | -2.436 | -2.165 | -2.26 |
| RCN2 | reticulocalbin 2, EF-hand calcium binding domain (RCN2) | NM_002902 | 0.0283052 | -1.159 | -1.132 | -1.46 |
| L20442 | Rhesus monkey p53 mRNA, complete cds [L20442] | L20442 | 0.0170867 | 1.2836 | 1.2648 | 1.4983 |
| NM_018054 | Rho GTPase activating protein 17 (ARHGAP17) | NM_018054 | 0.003048 | 1.2652 | 1.2937 | 1.5405 |
| NM_001011722 | Rho guanine nucleotide exchange factor (GEF) 10-like (ARHGEF10L), transcript variant 2 | NM_001011722 | 0.0189936 | 1.1788 | 1.5144 | 1.6448 |
| RHBDF1 | rhomboid 5 homolog 1 (Drosophila) (RHBDF1) | NM_022450 | 0.0150078 | 1.2484 | -1.016 | 1.803 |
| NM_001005498 | rhomboid, veinlet-like 6 (Drosophila) (RHBDL6), transcript variant 2 | NM_001005498 | 0.0435768 | 1.6119 | -1.016 | 2.133 |
| NM_000977 | ribosomal protein L13 (RPL13), transcript variant 1 | NM_000977 | 0.008562 | 3.2655 | 2.1384 | 2.0148 |
| RNF126 | ring finger protein 126 (RNF126), transcript variant 2 | NM_194460 | 4.43E-04 | -1.165 | -1.084 | -15.15 |
| RNF13 | ring finger protein 13 (RNF13), transcript variant 1 | NM_007282 | 0.007378 | -1.334 | -1.155 | -1.192 |
| RNF38 | ring finger protein 38 (RNF38), transcript variant 2 | NM_194328 | 0.0063375 | 1.3024 | 1.0953 | 1.3101 |
| MAK16 | RNA binding motif protein 13 (RBM13) | NM_032509 | 0.0112431 | 1.0619 | 1.2557 | 1.4038 |
| NM_001008408 | RNA binding motif protein 33 (RBM33) | NM_001008408 | 0.012448 | 1.7486 | 1.35 | 1.4156 |
| NM_001008710 | RNA binding protein with multiple splicing (RBPMS), transcript variant 1 | NM_001008710 | 2.88E-04 | 1.2022 | 1.7427 | 1.3039 |
| NM_184244 | RNA-binding region (RNP1, RRM) containing 2 (RNPC2), transcript variant 5 | NM_184244 | 0.0393745 | 1.1915 | 1.1238 | 1.2795 |
| SAPS2 | SAPS domain family, member 2 (SAPS2) | NM_014678 | 0.0080958 | 1.1498 | 1.1932 | 1.2135 |
| SRCRB4D | scavenger receptor cysteine rich domain containing, group B (4 domains) (SRCRB4D) | NM_080744 | 0.028798 | 1.2061 | 1.6506 | 1.0629 |
| SCRN3 | secernin 3 (SCRN3) | NM_024583 | 1.23E-04 | -2.081 | -1.014 | -3.483 |
| RP3-402G11.5 | selenoprotein O (SELO) | NM_031454 | 0.0065774 | 1.5716 | 1.0144 | 2.3801 |
| SH3YL1 | SH3 domain containing, Ysc84-like 1 (S. cerevisiae) (SH3YL1) | NM_015677 | 0.0445655 | -1.405 | -2.659 | -1.03 |
| SHCBP1 | SHC SH2-domain binding protein 1 (SHCBP1) | NM_024745 | 0.0382276 | -1.834 | -1.853 | -1.233 |
| NM_015996 | SID1 transmembrane family, member 2 (SIDT2) | NM_015996 | 0.0361974 | 1.4583 | 1.0432 | 1.277 |
| C5orf46 | similar to AVLV472 (MGC23985) | NM_206966 | 0.0477624 | 1.2346 | -10.76 | 1.0969 |
| OSTCL | similar to RIKEN cDNA 2310008M10 (LOC202459) | NM_145303 | 9.90E-05 | -1.174 | -1.076 | -17.57 |
| SLMO2 | slowmo homolog 2 (Drosophila) (SLMO2) | NM_016045 | 0.0257932 | -1.7 | -1.163 | -1.697 |
| SERF1B | small EDRK-rich factor 1B (centromeric) (SERF1B) | NM_022978 | 0.0026327 | -1.158 | -1.074 | -2.189 |
| NM_001009820 | small nuclear ribonucleoprotein 70kDa polypeptide (RNP antigen) (SNRP70), transcript variant 2 | NM_001009820 | 6.45E-04 | 1.43 | 1.4597 | 2.442 |
| NM_003056 | solute carrier family 19 (folate transporter), member 1 (SLC19A1), transcript variant 1 | NM_003056 | 0.0219214 | 1.7768 | 1.306 | 1.6618 |
| NM_014096 | solute carrier family 43, member 3 (SLC43A3) | NM_014096 | 4.21E-04 | -1.555 | -1.352 | -6.473 |
| NM_019003 | spindlin family, member 2 (SPIN2) | NM_019003 | 0.0013769 | 1.1075 | 1.3468 | 1.3152 |
| NM_001006682 | spindlin-like protein 2 (SPIN-2), transcript variant 2 | NM_001006682 | 0.0093755 | 1.2912 | 1.3466 | 1.0848 |
| NM_001006682 | spindlin-like protein 2 (SPIN-2), transcript variant 2 | NM_001006682 | 0.0028745 | 1.1478 | 1.3861 | 1.1452 |
| SPTY2D1 | SPT2, Suppressor of Ty, domain containing 1 (S. cerevisiae) (SPTY2D1) | NM_194285 | 0.0479728 | 1.0273 | 1.0549 | -2.096 |
| STARD3NL | STARD3 N-terminal like (STARD3NL) | NM_032016 | 0.0216416 | -1.302 | -1.65 | -1.357 |
| NM_001008410 | STEAP family member 3 (STEAP3), transcript variant 3 | NM_001008410 | 0.0187307 | -1.085 | -2.633 | -1.653 |
| SAMD1 | sterile alpha motif domain containing 1 (SAMD1) | NM_138352 | 0.0239787 | -1.06 | 1.0767 | 2.0112 |
| SAMD10 | sterile alpha motif domain containing 10 (SAMD10) | NM_080621 | 0.0020128 | 1.3128 | 1.0472 | 2.3011 |
| HSPA13 | stress 70 protein chaperone, microsome-associated, 60kDa (STCH) | NM_006948 | 0.0018644 | -1.509 | -1.055 | -2.359 |
| SDF4 | stromal cell derived factor 4 (SDF4) | NM_016176 | 0.0174905 | -1.221 | 1.0233 | -3.142 |
| SKIV2L | superkiller viralicidic activity 2-like (S. cerevisiae) (SKIV2L) | NM_006929 | 0.0273454 | 1.0173 | 1.1101 | 1.6274 |
| ST5 | suppression of tumorigenicity 5 (ST5), transcript variant 1 | NM_005418 | 0.0043675 | 1.012 | 1.5006 | 1.4931 |
| NM_198328 | suppression of tumorigenicity 7 like (ST7L), transcript variant 6 | NM_198328 | 0.0169334 | 1.2182 | 1.235 | 1.5179 |
| BC028095 | survival of motor neuron protein interacting protein 1 | BC028095 | 0.0138547 | -1.058 | -1.034 | -2.019 |
| SUSD4 | sushi domain containing 4 (SUSD4), transcript variant 1 | NM_017982 | 0.0498275 | 1.0717 | 1.7553 | 1.6347 |
| SYNGR2 | synaptogyrin 2 (SYNGR2) | NM_004710 | 0.0250393 | 1.1711 | 1.1764 | 1.2416 |
| BC043391 | TAF7-like RNA polymerase II, TATA box binding protein (TBP)-associated factor, 50kDa | BC043391 | 0.024515 | -1.892 | -1.598 | -2.027 |
| TELO2 | TEL2, telomere maintenance 2, homolog (S. cerevisiae) (TELO2) | NM_016111 | 0.0182485 | 1.2201 | 1.3395 | 1.4063 |
| TSPAN3 | tetraspanin 3 (TSPAN3), transcript variant 1 | NM_005724 | 0.008296 | -1.049 | 1.1827 | -4.4 |
| TTC17 | tetratricopeptide repeat domain 17 (TTC17) | NM_018259 | 0.0193896 | 1.1123 | 1.1344 | 1.811 |
| TTC21A | tetratricopeptide repeat domain 21A (TTC21A) | NM_145755 | 5.33E-04 | 1.0711 | 1.4858 | 2.0577 |
| TTC7A | tetratricopeptide repeat domain 7A (TTC7A) | NM_020458 | 0.0290389 | 1.3144 | -1.01 | 1.6921 |
| THAP1 | THAP domain containing, apoptosis associated protein 1 (THAP1), transcript variant 1 | NM_018105 | 0.0014763 | -1.657 | 1.0186 | -2.084 |
| THOC7 | THO complex 7 homolog (Drosophila) (THOC7) | NM_025075 | 0.0060426 | -1.195 | 1.0046 | -1.849 |
| THADA | thyroid adenoma associated (THADA), transcript variant 1 | NM_022065 | 0.0049217 | 1.3649 | 1.1458 | 1.2067 |
| NM_022333 | TIA1 cytotoxic granule-associated RNA binding protein-like 1 (TIAL1), transcript variant 2 | NM_022333 | 0.0025985 | 1.1306 | -1.015 | -10.95 |
| TM2D2 | TM2 domain containing 2 (TM2D2), transcript variant 1 | NM_078473 | 0.043884 | -1.189 | -1.044 | -1.46 |
| TOX4 | TOX high mobility group box family member 4 (TOX4) | NM_014828 | 0.0010564 | 1.1633 | 1.2726 | 1.1972 |
| TRABD | TraB domain containing (TRABD) | NM_025204 | 0.0403395 | 1.1533 | 1.1681 | 1.4654 |
| TM4SF19 | transmembrane 4 L six family member 19 (TM4SF19) | NM_138461 | 0.0112138 | -1.26 | 1.2676 | -3.866 |
| TM7SF3 | transmembrane 7 superfamily member 3 (TM7SF3) | NM_016551 | 0.0451341 | -1.624 | -1.359 | -1.197 |
| TMCC1 | transmembrane and coiled-coil domain family 1 (TMCC1), transcript variant 2 | NM_015008 | 0.0335203 | 1.1915 | 1.2129 | 1.2704 |
| TMTC3 | transmembrane and tetratricopeptide repeat containing 3 (TMTC3) | NM_181783 | 0.0221732 | -1.15 | -1.032 | -1.579 |
| RNFT2 | transmembrane protein 118 (TMEM118) | NM_032814 | 0.0177155 | -1.401 | -1.929 | -2.754 |
| ORAI3 | transmembrane protein 142C (TMEM142C) | NM_152288 | 0.0063331 | 1.6854 | 1.1915 | 1.3638 |
| TMEM14A | transmembrane protein 14A (TMEM14A) | NM_014051 | 0.0074929 | -1.221 | -1.063 | -1.848 |
| TMEM14B | transmembrane protein 14B (TMEM14B) | NM_030969 | 0.0388346 | -1.381 | -1.101 | -1.757 |
| FAM174A | transmembrane protein 157 (TMEM157) | NM_198507 | 4.49E-05 | -1.6 | 1.0353 | -2.285 |
| TMEM161B | transmembrane protein 161B (TMEM161B) | NM_153354 | 0.0325386 | -1.163 | -1.112 | -1.546 |
| TMEM2 | transmembrane protein 2 (TMEM2) | NM_013390 | 0.0051788 | -1.603 | -1.119 | -3.324 |
| TMEM20 | transmembrane protein 20 (TMEM20) | NM_153226 | 0.0010558 | -2.935 | -1.822 | -1.558 |
| FAM155B | transmembrane protein 28 (TMEM28) | NM_015686 | 0.0218961 | 1.8056 | 1.0721 | 1.155 |
| TMEM45B | transmembrane protein 45B (TMEM45B) | NM_138788 | 0.0283695 | 2.7895 | 1.1081 | 1.4345 |
| TMEM5 | transmembrane protein 5 (TMEM5) | NM_014254 | 0.0383844 | -1.542 | -1.182 | -1.908 |
| TMEM55B | transmembrane protein 55B (TMEM55B) | NM_144568 | 0.0105423 | 1.1759 | 1.0347 | 1.4746 |
| TMEM59L | transmembrane protein 59-like (TMEM59L) | NM_012109 | 5.34E-04 | 1.5736 | 3.137 | 1.408 |
| TMEM65 | transmembrane protein 65 (TMEM65) | NM_194291 | 0.0326446 | -1.727 | -1.1 | -1.552 |
| TMEM85 | transmembrane protein 85 (TMEM85) | NM_016454 | 0.0139164 | -1.469 | -1.198 | -1.8 |
| TRIM41 | tripartite motif-containing 41 (TRIM41), transcript variant 1 | NM_033549 | 0.0113574 | 1.3406 | 1.2983 | 1.7145 |
| TRIM50 | tripartite motif-containing 50 (TRIM50) | NM_178125 | 0.0473643 | 1.3955 | 1.318 | 2.5929 |
| TRIM6 | tripartite motif-containing 6 (TRIM6), transcript variant 1 | NM_001003818 | 0.0054246 | 2.7182 | 1.4518 | 1.9601 |
| WRB | tryptophan rich basic protein (WRB) | NM_004627 | 0.0258696 | -1.174 | -1.254 | -1.333 |
| NM_003790 | tumor necrosis factor receptor superfamily, member 25 (TNFRSF25), transcript variant 2 | NM_003790 | 0.0145071 | 1.0425 | 1.3631 | 1.877 |
| TNFAIP8L3 | tumor necrosis factor, alpha-induced protein 8-like 3 (TNFAIP8L3) | NM_207381 | 0.0246953 | -1.566 | -1.983 | -1.664 |
| TPD52L3 | tumor protein D52-like 3 (TPD52L3), transcript variant 1 | NM_033516 | 0.0160602 | 2.1602 | 1.7564 | -1.279 |
| NM_001008744 | tyrosyl-DNA phosphodiesterase 1 (TDP1), transcript variant 2 | NM_001008744 | 0.0354432 | -1.436 | -1.424 | -1.11 |
| UBAP2L | ubiquitin associated protein 2-like (UBAP2L) | NM_014847 | 0.0370175 | 1.423 | 1.4116 | 2.6679 |
| TMEM189-UBE2V1 | ubiquitin-conjugating enzyme E2 variant 1 (Kua-UEV), transcript variant 1 | NM_199203 | 0.0097578 | -1.302 | -1.169 | -1.231 |
| TMEM189-UBE2V1 | ubiquitin-conjugating enzyme E2 variant 1 (Kua-UEV), transcript variant 1 | NM_199203 | 0.0135521 | -1.319 | -1.093 | -1.328 |
| NM_003343 | ubiquitin-conjugating enzyme E2G 2 (UBC7 homolog, yeast) (UBE2G2), transcript variant 1 | NM_003343 | 0.03791 | -1.434 | 1.0265 | -1.366 |
| UBXN2A | UBX domain containing 4 (UBXD4) | NM_181713 | 0.0396301 | -1.273 | -1.347 | -1.212 |
| FAF2 | UBX domain containing 8 (UBXD8) | NM_014613 | 0.0012772 | 1.3183 | 1.065 | 1.4822 |
| CN479040 | UI-CF-FN0-afv-g-11-0-UI.s1 UI-CF-FN0 cDNA clone UI-CF-FN0-afv-g-11-0-UI 3' | CN479040 | 0.0209939 | -1.161 | -1.021 | -1.34 |
| BM991059 | UI-H-DI0-atp-f-13-0-UI.s1 NCI_CGAP_DI0 cDNA clone IMAGE:5862540 3' | BM991059 | 0.006156 | -1.215 | -1.23 | -1.846 |
| BQ017694 | UI-H-ED0-awy-k-04-0-UI.s1 NCI_CGAP_ED0 cDNA clone IMAGE:5825403 3' | BQ017694 | 3.93E-04 | 1.3141 | 1.0186 | 2.0529 |
| NM_054035 | unc-119 homolog (C. elegans) (UNC119), transcript variant 2 | NM_054035 | 0.0397224 | 1.7953 | 1.2236 | -1.068 |
| NM_153676 | Usher syndrome 1C (autosomal recessive, severe) (USH1C), transcript variant b3 | NM_153676 | 0.0013536 | 2.4309 | 2.0038 | -1.031 |
| VWCE | von Willebrand factor C and EGF domains (VWCE) | NM_152718 | 0.0078819 | 1.3275 | 1.9014 | 1.7557 |
| WFIKKN1 | WAP, follistatin/kazal, immunoglobulin, kunitz and netrin domain containing 1 (WFIKKN1) | NM_053284 | 0.0370106 | 1.3722 | 1.2584 | 1.4095 |
| WDFY2 | WD repeat and FYVE domain containing 2 (WDFY2) | NM_052950 | 0.0377534 | -1.111 | -1.037 | 2.4792 |
| WDR22 | WD repeat domain 22 (WDR22) | NM_003861 | 0.0411861 | 1.1801 | 1.1496 | 1.2378 |
| WDR24 | WD repeat domain 24 (WDR24) | NM_032259 | 6.05E-04 | 1.2511 | 1.2026 | 1.5621 |
| WDR34 | WD repeat domain 34 (WDR34) | NM_052844 | 0.0017708 | 1.2661 | 1.1176 | 1.7789 |
| WDR51B | WD repeat domain 51B (WDR51B) | NM_172240 | 0.0230189 | -1.469 | -1.391 | -1.666 |
| WDR61 | WD repeat domain 61 (WDR61) | NM_025234 | 0.0477684 | -1.173 | 1.061 | -1.177 |
| WDR68 | WD repeat domain 68 (WDR68) | NM_005828 | 0.0358818 | 1.0591 | 1.1995 | 1.0653 |
| WDR75 | WD repeat domain 75 (WDR75) | NM_032168 | 7.83E-05 | -2.353 | -1.037 | -17.03 |
| WRAP53 | WD repeat domain 79 (WDR79) | NM_018081 | 0.0128129 | -1.04 | 1.154 | 1.5264 |
| WDR8 | WD repeat domain 8 (WDR8) | NM_017818 | 0.0046812 | -5.68 | -2.978 | -8.061 |
| WDR81 | WD repeat domain 81 (WDR81) | NM_152348 | 0.0033325 | 1.2773 | -1.168 | 1.8939 |
| NM_014919 | Wolf-Hirschhorn syndrome candidate 1 (WHSC1), transcript variant 4 | NM_014919 | 0.011045 | 1.3859 | 1.07 | 1.334 |
| GPN1 | XPA binding protein 1, GTPase (XAB1) | NM_007266 | 0.0184459 | -1.114 | -1.048 | -1.272 |
| YIPF6 | Yip1 domain family, member 6 (YIPF6) | NM_173834 | 0.0082897 | -1.359 | -1.197 | -1.277 |
| NM_194320 | zinc finger protein 169 (ZNF169) | NM_194320 | 3.18E-04 | 1.4186 | -1.019 | 2.0724 |
| RNF112 | zinc finger protein 179 (ZNF179) | NM_007148 | 0.0355366 | 1.2877 | 1.3913 | 1.4519 |
| NM_006385 | zinc finger protein 211 (ZNF211), transcript variant 1 | NM_006385 | 0.0109645 | 1.3037 | 1.1746 | 1.4461 |
| NM_152283 | zinc finger protein 62 homolog (mouse) (ZFP62) | NM_152283 | 0.0296514 | 1.285 | 1.2005 | 1.3366 |
| ZFAND3 | zinc finger, AN1-type domain 3 (ZFAND3) | NM_021943 | 0.0046086 | -1.686 | -1.122 | -3.182 |
| ZDHHC8 | zinc finger, DHHC-type containing 8 (ZDHHC8) | NM_013373 | 0.0164508 | 1.224 | 1.0878 | 1.3916 |
| ZFYVE1 | zinc finger, FYVE domain containing 1 (ZFYVE1), transcript variant 1 | NM_021260 | 0.0273629 | 1.1995 | 1.0585 | 1.4753 |
| NM_032850 | zinc finger, FYVE domain containing 19 (ZFYVE19) | NM_032850 | 0.0100409 | 1.1407 | 1.0691 | 2.0565 |
| NM_032997 | ZW10 interactor (ZWINT), transcript variant 2 | NM_032997 | 0.0470782 | -1.917 | -1.504 | -1.517 |
| A_01_P000558 | GCAGTCATTAATTGCAGTAAAATAAAATACGATCCCATTAGGGAATCTTGAATTCTGACC |  | 0.0058613 | -1.168 | -1.277 | -1.934 |
| A_01_P000705 | AGCCTCCAGCTACTATTACAATGTGAAAATTCCAATGATCCATTTACCAGAGCAGTTAGA |  | 0.0132548 | 1.0129 | -1.317 | -1.644 |
| A_01_P000953 | ATTTGTTAACTTTTGACAGAGAAAATAGTGTTCTCTGTTCTAGCAGGTAAATCCTCTACG |  | 0.0043681 | -1.253 | -1.369 | -1.7 |
| A_01_P000977 | ATTAAAGGTGCAGTTGGTACAAGAATCAGAAGAGAAGAAAAGTTAATACTACATTGAGTT |  | 0.0123337 | -1.099 | -1.051 | -2.676 |
| A_01_P002411 | TCACTGGAATAGTTTATCTTGTTTTAAAATATTATTGGCGCTGGTACAACAGCATCCAAC |  | 0.0080307 | -1.579 | -1.787 | -1.279 |
| A_01_P002494 | CTGATGGGGTTGAAGTCAAAAGACCAAAATACTAATCACCAGTTACAACCGGAGACCCTC |  | 0.0295851 | 1.1554 | 1.192 | 1.1355 |
| A_01_P003040 | TAGGTGTGAGCTGCATGCTATTGTGGGGATTTGGCACTGAAGTCTAGGTGCTTTTGTTTT |  | 0.0499774 | 1.1867 | 1.1377 | 1.3644 |
| A_01_P003241 | TGTAAGGAGCTCATTTCTGAACAAAAAGGTTTGCTCTGTGGAAAAATCAATCACTGCCAG |  | 0.0056265 | -1.447 | -1.211 | -1.353 |
| A_01_P003242 | TTGTATAACTGAATTTGTTCACTTCTCTCACACCAGCAAGTGTTTTACAGGTGCCTTGGA |  | 0.0057904 | -1.278 | -1.184 | -1.515 |
| A_01_P003244 | TGTGGAGTGTGACAATGGTGTTTGTGTAACGATCCGTTATCGTTCGGTATCGAACGTAAC |  | 0.0129278 | 1.012 | -1.023 | -2.615 |
| A_01_P003292 | TCCAGTGAAACTGCAGGGAAATGCAGGTAGAATCTTGGGAGGTAATAATGATGTGAAACA |  | 0.0057737 | -1.179 | -1.003 | -1.538 |
| A_01_P004730 | GCTACCTTAATGTATTGCTACAAAAATCTCTTGTTAGGGGTAATGCAGGTAATAAAGTAG |  | 0.0020621 | -1.541 | -1.612 | -1.305 |
| A_01_P005323 | GGAAAAATCTTTGTTTAGATATTGTGTCATATCTGGATATATCTGAATCCTAAAGGATTA |  | 0.0247731 | -1.428 | -1.029 | -1.343 |
| A_01_P005403 | GTATTGTATCATGTACATTCCTGATTTAATATTTTACAGAACATTTTATTCAGATATCAA |  | 0.0099933 | -2.415 | -1.289 | -3.79 |
| A_01_P005676 | CCCACTCCTTGCTTTGTCTCCCTGGATATGGATTTCAGTTTTTTGTAACCCGTTACACTG |  | 0.0056694 | 1.2472 | 1.3236 | 1.3139 |
| A_01_P005911 | GTCAGATATTATGACAAAATTTGACATTAATTGTTTTTTAAAGTATAGATTTCATTTGAA |  | 7.32E-06 | -1.888 | -1.174 | -8.813 |
| A_01_P007044 | TGCTTAAGCAGAAGCATTAACTTCTCTGGAAAGGGGGAAGCTGGGGAAACTCAAACTCTT |  | 0.0315541 | 1.1515 | 1.0761 | 1.5152 |
| A_01_P008101 | AGCGCCACTGCAACATATAGACCCGAGTGCTATTGTATTTTGGCTTGGTGTGTATGCTTT |  | 0.0237233 | 1.2669 | 1.1647 | 1.3406 |
| A_01_P009731 | TGCGGTTTCCAAGGCCTCCGGAAGCACCAACCCAGTTCTCAGTTACCTCCTCTGGCCGGA |  | 0.004496 | 1.349 | 1.1183 | 2.375 |
| A_01_P010016 | TAAGGTTGGTTTGCAGTGTGTAAGTGGCACATTGAACTGGAAGTTTTCTTGAAAGCGGCT |  | 0.0366487 | -1.33 | -1.043 | -1.519 |
| A_01_P010048 | CTCGATCTAGAAGTTTTCTCTTTGAATCCTTCCATCTCTTTCTCCTGATACCCCAGATGA |  | 0.0114191 | 1.5532 | 1.8083 | 1.3047 |
| A_01_P010968 | GCAATGATACAAAGATAACTGATAAAATATATTACATTCAATGAGGTTTTCTTTTACAAA |  | 2.91E-04 | -1.772 | -1.723 | -6.516 |
| A_01_P010998 | TATATTGCATATTGATCATTGGCTTTTTCTAGAAAAACTGCCTCTGATTCTGGACAAAGC |  | 0.0460227 | -1.271 | -1.01 | -1.397 |
| A_01_P011256 | GAGAGAAGCCTGGTGAAAGCCATCTGAAGATGATCCCTGAAGAGGTGTGGTGGATGTGCT |  | 0.0122165 | 1.3166 | 1.258 | 2.4209 |
| A_01_P011698 | TTCTCTGGACAAGTCATTATGCTCGTACTTTGGGCTCACAGGCTCCACTTCCCTGGACAC |  | 0.0215608 | 1.6561 | 1.1859 | 1.0557 |
| A_01_P012427 | AGCATTCCTGGAAATTCTAACTAACAGATGTTCTTGCACATTGACATAAGCCACCTGCAG |  | 0.0437574 | 1.5462 | 1.3294 | 1.6004 |
| A_01_P013390 | AAAGACTAAAAACGTTTTGATATTATACAATGTATTTGTTTCAGATAAGGTCGTTTTCAT |  | 0.0298691 | -4.625 | -1.099 | -1.527 |
| A_01_P014141 | CGCCCAAGTCCCCTTGTACTGCCATCTAGTCGAATAGAAGTCCTCCCTGTTGACGTTGGT |  | 0.0127006 | 2.2149 | 1.1558 | 1.0935 |
| A_01_P014575 | GCAAATGAAAGAAATATTTCCTGAACAGGATGATCAACCCTATGTAGTAGACGACTTGAC |  | 0.0476683 | -1.733 | -1.139 | -1.615 |
| A_01_P015150 | ACTTATCTGGGACGATCTGCGGAGCCTGTGCCTCTTCAGCTACCACCGCTTGAGAGACTT |  | 0.0156501 | 2.0042 | 1.3322 | -1.172 |
| A_01_P015364 | TCAATTGGGAAGATATTTTAGAGTCTAGATAATTATGTTTGTATATTGAAAAAATGGTGG |  | 7.30E-04 | -2.148 | -1.376 | -3.354 |
| A_01_P015849 | AAATGGGCTACATTTTCTACCTCAGAAAATTCCACGAAAACCCTTATGAAATTTAAGGGT |  | 0.0080151 | -1.605 | -1.095 | -6.224 |
| A_01_P015993 | GTTTCGGCATGGAGAGGGACTCCTGTCTAGTCTAGAAATGTGTGTTACTTACATGTCTCT |  | 0.0376075 | 1.49 | -1.026 | 2.1869 |
| A_01_P015995 | GTTTGGCTTTAAACACTACCCAGGCAGGAAGGTGGCTTTGGTAAGAATTTTGAAGTGGAT |  | 0.0249369 | 1.4441 | 1.1181 | 2.0235 |
| A_01_P017592 | TCTTCTAGAGTTACAAATTGAATTTTTAAATCTGAGCGCCTTTGTTGTGGTGTGGAGAAA |  | 1.32E-04 | -1.112 | -1.495 | -1.839 |
| A_01_P017935 | ACAGACACCCTTGATGTGGGTTTGTTAAGAACATAGAAGAACAGGAAAAGTTGCCAGGTT |  | 0.0237347 | -1.556 | -1.164 | -1.161 |
| A_01_P018169 | TTTGACATCCCAGTTTGCGTCAGTGACAGAACTTACTGCTTGGTCTTTGTAGTTTTAAAA |  | 0.0390885 | -1.202 | -1.022 | -1.221 |
| A_01_P018321 | GAAAACTGTAAAAGCAAAGACTGTAGTAATGACACAAATATGACAAACAGATGTTTTCTT |  | 0.0426833 | -1.555 | -1.112 | -2.497 |
| A_01_P018380 | ATTACCTCAAAACCCTGGGAAGGTAAAGAATGTGGTATATCCTTTAAATATTTCACATGC |  | 0.0493524 | -1.117 | 1.0348 | -1.638 |
| A_01_P018736 | CCTAGGAAAATGTGATTGTTAACTAGGATTCTGTTTTACATGTTGACATTTCCAACACAC |  | 0.0361774 | -1.386 | -2.103 | -1.328 |
| A_01_P018737 | TACGAATCCAATCACCAAGGAAACAGGAAGAGGAAAACATCCAAAACTACAATGACGATA |  | 0.0143345 | -1.543 | -2.128 | -1.575 |
| A_01_P019355 | CTGCAAAATGGCCCCTACCAAGCTGCAGAAAAGCATTTCATCTTTTTGGAGGTTTCCACA |  | 0.0206523 | 1.562 | 1.4188 | 1.167 |
